# Computational analysis of variability and uncertainty in the clinical reference on magnetic resonance imaging radiomics: modelling and performance

**DOI:** 10.1186/s42492-024-00180-9

**Published:** 2024-11-19

**Authors:** Cindy Xue, Jing Yuan, Gladys G. Lo, Darren M.C. Poon, Winnie CW Chu

**Affiliations:** 1 Research Department, Hong Kong Sanatorium and Hospital, Hong Kong, China; 2 Department of Imaging and Interventional Radiology, The Chinese University of Hong Kong, Hong Kong, China; 3 Department of Diagnostic and Interventional Radiology, Hong Kong Sanatorium and Hospital, Hong Kong, China; 4 Comprehensive Oncology Centre, Hong Kong Sanatorium and Hospital, Hong Kong, China

**Keywords:** Prostate cancer, Magnetic resonance imaging, Radiomics, Reliability, Clinical reference

## Abstract

To conduct a computational investigation to explore the influence of clinical reference uncertainty on magnetic resonance imaging (MRI) radiomics feature selection, modelling, and performance. This study used two sets of publicly available prostate cancer MRI = radiomics data (Dataset 1: *n* = 260; Dataset 2: *n* = 100) with Gleason score clinical references. Each dataset was divided into training and holdout testing datasets at a ratio of 7:3 and analysed independently. The clinical references of the training set were permuted at different levels (increments of 5%) and repeated 20 times. Four feature selection algorithms and two classifiers were used to construct the models. Cross-validation was employed for training, while a separate hold-out testing set was used for evaluation. The Jaccard similarity coefficient was used to evaluate feature selection, while the area under the curve (AUC) and accuracy were used to assess model performance. An analysis of variance test with Bonferroni correction was conducted to compare the metrics of each model. The consistency of the feature selection performance decreased substantially with the clinical reference permutation. AUCs of the trained models with permutation particularly after 20% were significantly lower (Dataset 1 (with ≥ 20% permutation): 0.67, and Dataset 2 (≥ 20% permutation): 0.74), compared to the AUC of models without permutation (Dataset 1: 0.94, Dataset 2: 0.97). The performances of the models were also associated with larger uncertainties and an increasing number of permuted clinical references. Clinical reference uncertainty can substantially influence MRI radiomic feature selection and modelling. The high accuracy of clinical references should be helpful in building reliable and robust radiomic models. Careful interpretation of the model performance is necessary, particularly for high-dimensional data.

**Supplementary Information:**

The online version contains supplementary material available at 10.1186/s42492-024-00180-9.

## Introduction

Radiomics is a technique that extracts quantitative features from medical images and utilizes machine learning methods to aid personalized clinical decisions in diagnosis, prognosis, and clinical decision support [1, 2]. By leveraging the advancements in quantitative imaging methods and computational analysis, such as machine learning, the application of radiomics in medicine has increased considerably [3]. Machine learning techniques commonly applied to radiomics include feature selection and data modelling. There are various feature selection techniques such as recursive feature elimination (RFE), minimum redundancy maximum relevance (mRMR) feature selection, and least absolute shrinkage and selection operator (LASSO) as well as data modelling techniques such as random forest (RF) and LASSO modelling [4]. Feature selection aims to reduce the high dimensionality of radiomics by selecting the relevant features and removing the irrelevant and redundant features [4, 5]. Data modelling is employed to interpret selected radiomic features to execute tasks such as diagnosing diseases or predicting treatment outcomes [4, 5]. Radiomics also has numerous clinical oncology applications, including classifying the aggressiveness of prostate cancer [6], aiding in the diagnosis and prognosis of head and neck cancer [7], and grading gliomas [8].

Despite the potential of radiomics, its reliability and generalizability in clinical applications, particularly with diverse clinical data, remain significant concerns requiring further investigation [9, 10]. The reliability of radiomics can arise at every step of a complicated radiomics workflow. The influence of many factors, including imaging scanner hardware/software configurations, patient setup, image acquisition, image reconstruction, image post-processing (e.g., filtering, segmentation, registration), radiomics feature quantification (e.g., feature definition, feature calculation, image discretization), and machine learning algorithms, and their influence on radiomics feature selection, modelling, and performance have been extensively investigated and demonstrated [4, 11–15].

For all clinical studies and applications, a high standard of clinical reference (or ground truth or endpoint) and accurate annotation are essential. However, in almost all radiomics studies, clinical references are commonly overlooked and presumed to be perfect, without bias or error. However, many uncertainties and variabilities are associated with clinical references. The variabilities could be due to low reference standards, different criteria/definitions, human-induced bias, disagreement, or errors in data sampling, collection, labelling, and annotation [16]. For example, biopsy samples used for clinical reference might provide suboptimal results owing to potential subsampling or missampling issues compared to pathological samples, necessitating caution in interpretation. Different criteria can be used for cancer staging or risk stratification, such as the D’Amico Risk Classification, N2ncertainty and variability on MRI radiomics modelling and performance. This analysis will involve clinical reference annotation permutations at different levels using publicly available prostate cancer radiomics datasets.

## Methods

This study used publicly available radiomics feature datasets to ensure reproducibility and external validation. The requirement for Institutional Review Board approval was waived for this retrospective study.

### Datasets

To enhance external validation and promote the reproducibility of results, an increasing number of datasets have become publicly available. Accordingly, this study used public MRI radiomics datasets. Currently, there are a few publicly available MRI radiomics feature datasets that mainly focus on the prostate, brain, and breasts [4, 17, 18]. Considering the limited availability of relevant datasets, we selected and utilized two publicly available MRI radiomics datasets for prostate cancer after evaluating the nature and number of subjects in each dataset. Both datasets have different characteristics that could potentially have different impacts on radiomics modelling. The first dataset had a relatively larger sample size with fewer features, whereas the second had a relatively smaller sample size with many features (Table 1).

**Table 1:** Overview of the prostate cancer datasets used for this study

Dataset	Sample size	No. of features	Outcome	Outcome balance	Modality	DOI
Dataset 1 [18]	260 lesions	265	Clinically significant vs non-clinically significant prostate cancer	49%	Multiparametric MRI (T2W, diffusion-weighted imaging and apparent diffusion coefficient maps)	10.1371/journal.pone.0237587
Dataset 2 [17]	100 lesions	7106	Clinically significant vs non-clinically significant prostate cancer	80%	T2-TSE, diffusion-weighted imaging and T2 mapping	10.1371/journal.pone.0217702

The clinical outcomes of both datasets were divided based on the Gleason score (GS) of the patients. GS is a pathological grading system used to evaluate the aggressiveness of prostate cancer based on the microscopic appearance of cancerous tissue samples obtained via either prostatectomy or biopsy [17–19]. This can help guide treatment decisions and predict the prognosis of patients with prostate cancer [19]. In this study, the scores could be classified into clinically significant prostate cancer (GS ≥ 3 + 3) and non-clinically significant prostate cancer (GS = 3 + 3). These outcomes were lesion-based, with one derived from whole-mount prostatectomy [17] and another from biopsy [18]. Both methods have been previously reported [17, 18]. These two datasets are high-dimensional, indicating that they contain more features than the samples. Only the radiomics feature values provided in the original studies were used in this study, encompassing various features such as first-order radiomics features, including max features signifying the highest intensity level or the brightest pixel, min features showing the darkest pixel or the lowest intensity in the image, grey-level co-occurrence matrix contrast features measuring the intensity discrepancies between neighbouring pixels, and many other features. The complete list of feature names used in this study is presented in Supplementary Tables 1 and 2.

### Training dataset and testing dataset

**Fig. 1:**
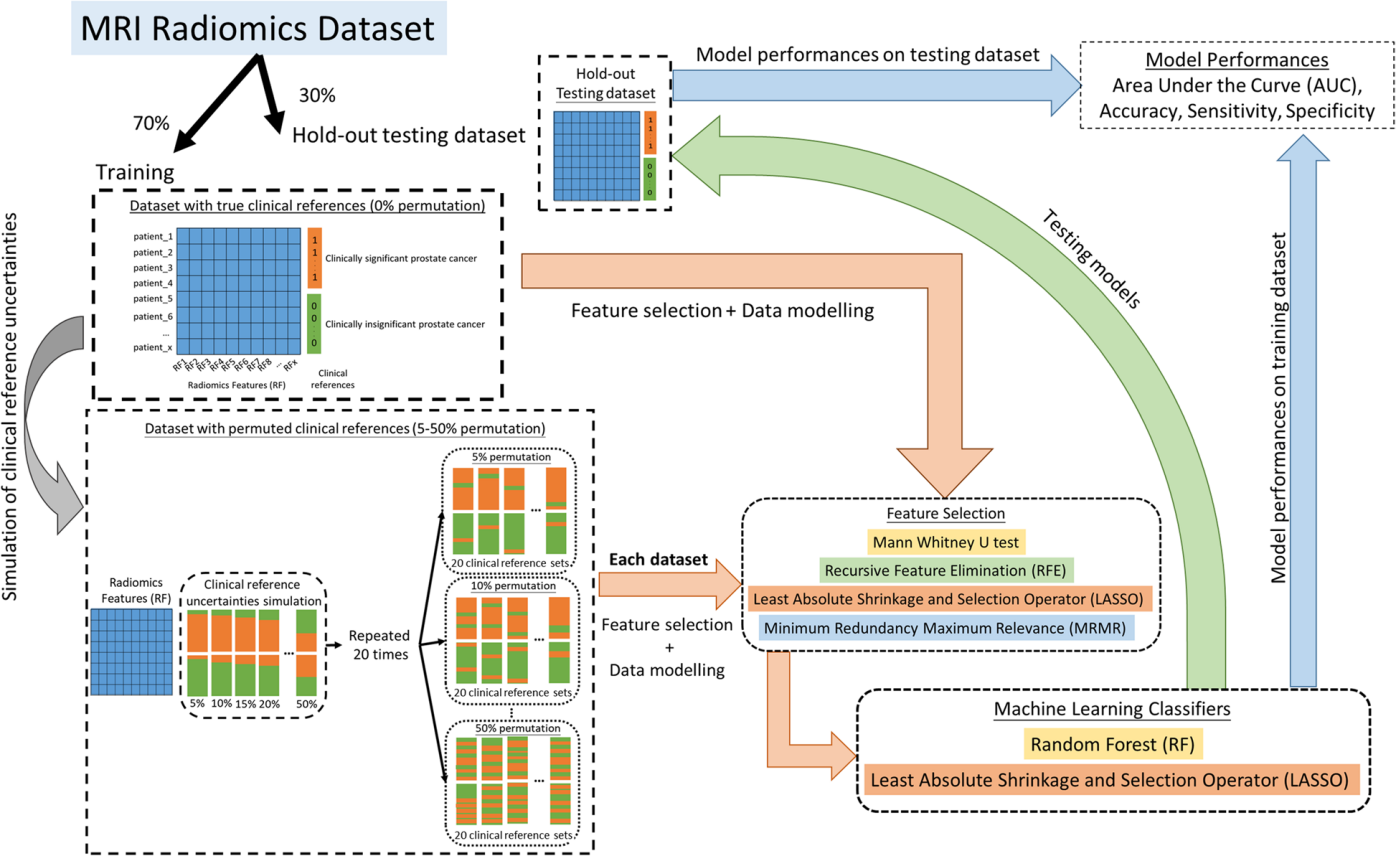
The overall workflow of this study, from obtaining the MRI radiomics dataset to measuring the performance of the radiomics model

The data from each dataset were separated into two subsets: training and holdout testing datasets, at a ratio of 7:3. This partitioning strategy ensures that a substantial portion of the data is dedicated to training the model, while retaining a separate set for unbiased evaluation. In addition, the ratio of reference cases with clinical significance to those considered clinically insignificant was carefully maintained as consistently as possible in the training and holdout testing sets. The overall workflow is illustrated in Fig. 1.

### Clinical reference uncertainties simulation

Clinical reference uncertainties were simulated by randomly inverting the clinical references between the clinically significant and clinically insignificant prostate tumours. Clinical reference permutations were conducted only in the training dataset and not in the hold-out data for testing. The number of samples was permuted at 11 levels with 5% increments (0%, 5%, 10%, 15%, 20%, 25%, 30%, 35%, 40%, 45%, and 50%) and repeated 20 times (with different random sample selections) at each level. The reference balance (clinical significance vs non-significance) ratio was maintained as consistently as possible at each permutation level. The details of this workflow are presented in Fig. 1 and Supplementary Table 3.

### Radiomics feature selection methods

Feature selection was utilized to choose a subset of relevant features from the datasets to enhance the model performance by retaining the most informative parameters while reducing complexity and computational cost. Four different feature selection methods are commonly used in radiomics studies [4, 8, 17, 18], which are Mann-Whitney U test (with a threshold of 0.05), RFE, LASSO, and mRMR. These techniques were applied to identify and preserve the most relevant features for our analysis with the aim of improving model accuracy, mitigating overfitting, and enhancing interpretability. The parameter settings for feature selection are listed in Supplementary Table 4.

### Machine learning classifiers

After feature selection, two classifiers were utilized to build the models: RF classifiers and LASSO. RF and LASSO were selected as classifiers because of their established effectiveness in radiomic analyses [4]. Finally, eight radiomics models (four feature selection algorithms × two classifiers) were constructed for each reference permutation. These were the Whitney U test + RF, LASSO + RF, RFE + RF, mRMR + RF, Whitney U test + LASSO, RFE + LASSO, mRMR + LASSO, and LASSO + LASSO. The parameter settings of the classifiers are listed in Supplementary Table 5.

### Data training, validation, and testing

Dataset 1 was trained using a ten-fold stratified cross-validation, whereas Dataset 2 was trained using a five-fold stratified cross-validation because of the relatively smaller sample size. The performances of the radiomics models trained based on different permutation levels of clinical references were first assessed against the permuted clinical references in the training dataset. Subsequently, the true performance of these radiomics models was tested and evaluated against true clinical references without permutation using the training and hold-out testing datasets.

### Statistics

JSC (range: 0-1; 0: no commonly selected features; 1: completely the same selected features) was calculated to assess feature selection consistency for each method (Formula 1) [20]. Because there were 20 repetitions at each permutation level, the JSC was calculated by the intersection of the features selected from all 20 permuted datasets over the union of those datasets.

1 $$JSC=\frac{A\cap B}{A\cup B}$$

A = Dataset A.

B = Dataset B.

The area under the curve (AUC) of the receiver operating characteristic curve, sensitivity, specificity, and accuracy of each model were computed and compared using ANOVA with Bonferroni correction [21]. Statistical significance was set at *P* value < 0.05. Statistical analyses were performed using the R Studio software.

## Results

### Feature selection

**Fig. 2:**
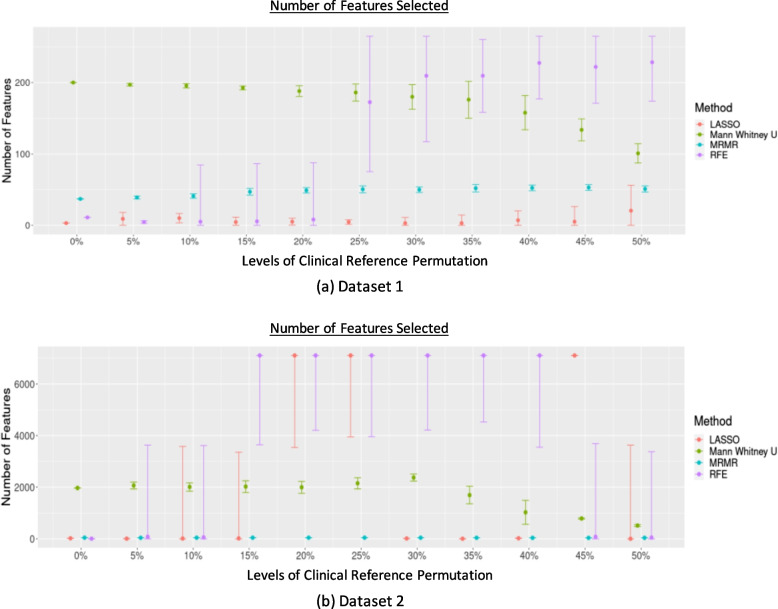
The proportion of features selected by the different feature selection methods at different levels of clinical reference permutations for (**a**) Dataset 1 and (**b**) Dataset 2

The number of features selected by each selection varied across the different permutation levels of the clinical references. The results of the four feature selection methods at different permutation levels are shown in Fig. 2. Overall, the most stable number of features across different permutation levels was selected using mRMR, followed by LASSO.

**Fig. 3:**
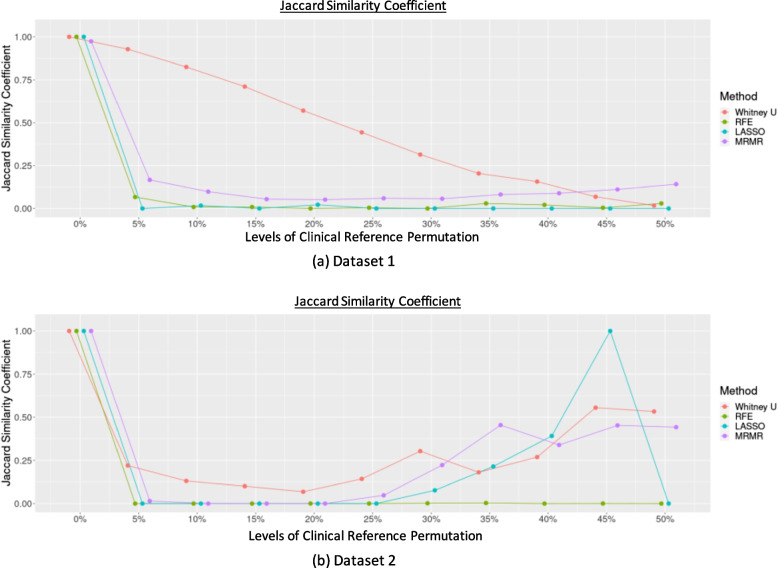
The JSC across different levels of permutation for (**a**) Dataset 1 and (**b**) Dataset 2

The most stable features selected using different feature selection methods for each dataset are listed in Supplementary Table 6. The JSC calculated to compare the selected features in each repetition by each feature selection method across different levels of clinical reference permutations is shown in Fig. 3. The JSC for different thresholds set to limit the maximum number of features selected by RFE, mRMR, and LASSO, showing the same trend as the overall feature selection performance, is shown in Supplementary Fig. 1.

JSC dropped substantially even when the permutation level was low (≥ 5%). The least decrease was observed when the Mann-Whitney U test was used. There is an increase in JSC at the permutation level ≥ 30% when using Mann-Whitney U test, LASSO, and mRMR in Dataset 2, while the JSC remained stably low in Dataset 1 across different permutation levels.

### Model performances

#### AUC

- Radiomics model performance in the training datasets with permuted clinical references

**Fig. 4:**
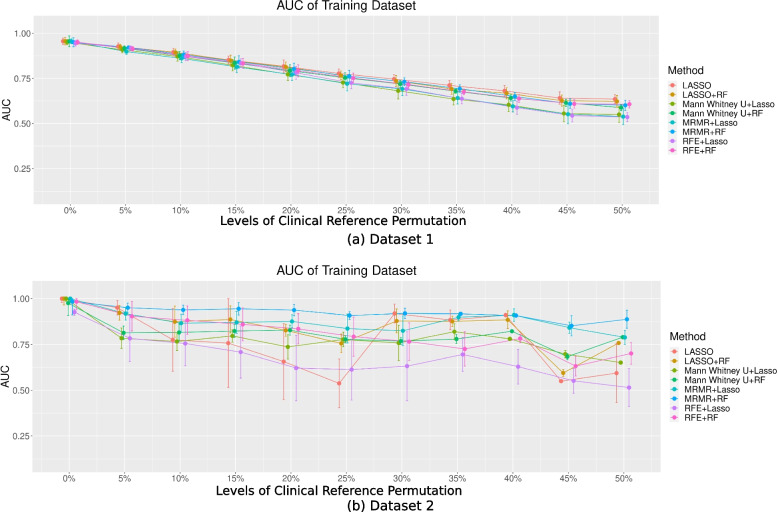
The AUC of the eight models in two different datasets with different levels of permutation toward the clinical references for (**a**) Dataset 1 and (**b**) Dataset 2

The AUCs for the eight models trained based on the permuted clinical references and tested against the permuted references in the training datasets are shown in Fig. 4.

Different feature selection and classification combinations did not lead to a significantly different model performance (*P* > 0.05). In Dataset 1, the mean AUCs showed a noticeable decreasing trend with increasing permutation levels. The mean AUCs with permuted clinical references (even only 5%) [5%: 0.91; 10%: 0.87; 15%: 0.83; 20%: 0.79; 25%: 0.74; 30%: 0.71; 35%: 0.67; 40%: 0.63; 45%: 0.59; 50%: 0.58] were significantly different from those without permutation [0%: 0.94] (*P* < 0.05). In Dataset 2, the AUCs varied significantly at different permutation levels, with no apparent decreasing trends. They were also associated with significantly larger uncertainties across the permutation repetitions, as indicated by the wide error bars. The AUCs were significantly lower for ≥ 20% permutation levels [averaged AUC: 0.74] compared to those of no permutation [averaged AUC: 0.97], except for mRMR + RF and mRMR + LASSO (*P* < 0.05). The performance of the models without permutations was comparable to that of previous studies [17, 18].

- Radiomics model (true) performance in the training datasets with true clinical references

**Fig. 5:**
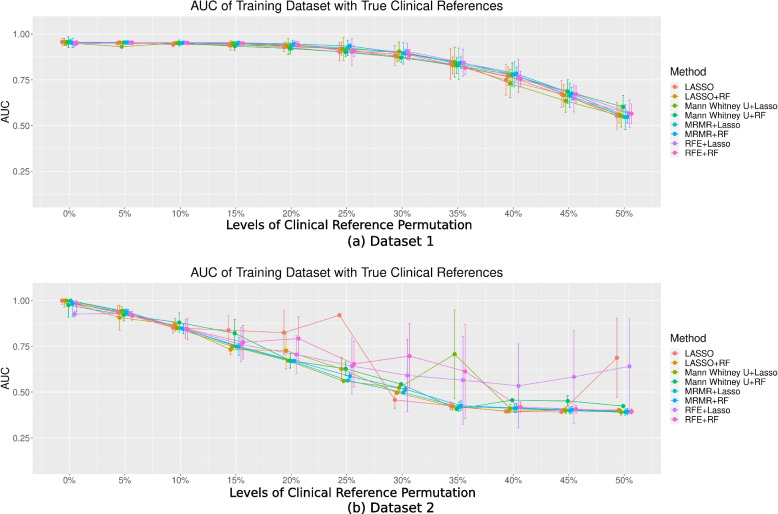
The AUC of the eight models in two different datasets with different permutation levels toward the clinical reference for (**a**) Dataset 1 and (**b**) Dataset 2 in the training dataset with true clinical references

The AUC for the eight models trained based on the permuted clinical references and tested against the true references in the training datasets are shown in Fig. 5. In Dataset 1, the AUCs were mostly constant without significant differences (*P* > 0.05) when the permutation levels were ≤ 15%. However, in Dataset 2, the AUCs were significantly different (*P* < 0.05) when the model was trained with ≥ 10% permutations [AUC: 10%: 0.85 vs 0%: 0.97]. For higher permutation levels, the AUC for each model generally decreased in both datasets, but with larger variabilities in Dataset 2. Different feature selection and classification combinations did not lead to significantly different model performance (*P* > 0.05) for each permutation.

- Radiomics model performance in the hold-out testing dataset

**Fig. 6:**
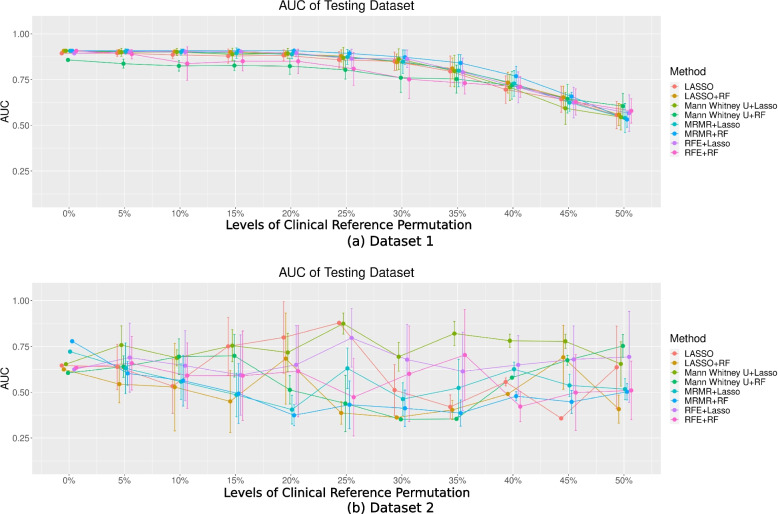
The AUC of the eight models in two different datasets with different levels of permutation toward the clinical reference for (**a**) Dataset 1 and (**b**) Dataset 2 in the testing dataset

The AUC for the eight models trained based on the permuted clinical references and tested against the true references in the hold-out testing dataset are shown in Fig. 6. The performance of the model remained stable and then decreased with permutation levels in Dataset 1 (Fig. 6). The AUCs of the testing datasets in Dataset 2 fluctuated across different levels of clinical reference permutation. AUCs of the models trained with ≥ 20% reference permutations using mRMR + LASSO and mRMR + RF were found to be significantly lower (*P* < 0.05) from that trained without permutation [0%: 0.72 (mRMR + LASSO), and 0.78 (mRMR + RF)], except for mRMR + LASSO at 25% levels of permutation. In addition, in Dataset 2, AUCs were generally poor (≤ 75%) regardless of the permutation levels based on which the radiomics models were trained.

#### Modelaccuracy

**Fig. 7:**
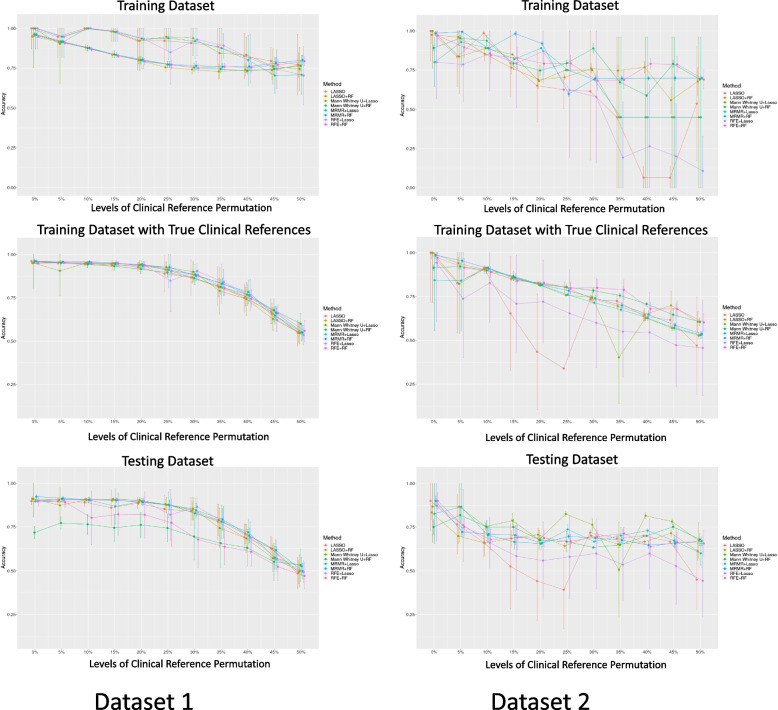
The accuracy of the eight models in two different datasets with different permutation levels toward the clinical reference for Datasets 1 and 2

The accuracy of the eight models trained based on permuted clinical references and tested against the training dataset with both permuted and true references is shown in Fig. 7. The accuracy of the model generally decreases for both datasets. In Dataset 1, the accuracy was stable without significant differences (*P* > 0.05) when the model was trained with ≥ 20% permutation, especially when tested against datasets with true references. In Dataset 2, the accuracy exhibited a steeper decreasing trend with larger variability, as reflected by the wider error bars. The accuracy of the models trained with ≥ 20% permutations (mean accuracy: 0.63-0.78) was significantly lower (*P* < 0.05) than those trained without permutations [mean accuracy: 0.86-0.93]. The sensitivity and specificity of the models with the same trend as the accuracy of the models for both Datasets 1 and 2, showing agreement with the accuracy of the models, are shown in Supplementary Figs. 2 and 3.

## Discussion

Uncertainties and variabilities in clinical references can be caused by many factors, some of which are unavoidable. Understanding these uncertainties and variabilities in clinical references and their impact on radiomics is crucial for designing and conducting high-quality radiomics studies and correctly interpreting the results. Generally, accurate clinical references are vital for studies utilizing machine learning methods in clinical settings [22]. Radiomics models trained based on high-quality clinical references should be robust and reliable.

From our computational analysis, the model performance during training showed decreasing trends with permuted references in Dataset 1 (Fig. 3a). However, the true performance of the model, when tested with true clinical references, remained satisfactory overall and even better than the training model performance (Figs. 4a and 5a) when the permutation levels were within 15%. When the permutation levels were above 15%, the performance of the model had wider uncertainties (wider error bars), which decreased with a further increase in the permutation levels. This finding implied that the built radiomics models were robust to reference permutations of up to 15% and genuinely revealed the true tumour properties, supporting the application of radiomics in clinical settings [1, 2, 23]. However, in Dataset 2, although the model’s AUCs during training appeared satisfactory across different levels of permuted clinical references (Fig. 3b), the true model performance decreased substantially when tested against the true references in the training data, with deeper drops when the level of clinical reference permutation increased (Fig. 4b). This suggests that the model fit the training dataset extremely well, including the permuted clinical references, which hindered the model from learning the true information; hence, the decrease in the performance of the models reflects the uncertainties of the clinical references. When applied to the hold-out testing data, the true model performances were poor overall and associated with large uncertainties (Fig. 5b), regardless of how the true clinical references were permuted. Overall, the results from both datasets show a decreasing trend in the performance of the models with increasing levels of clinical reference permutations. This decrease in performance with an increase in the permutation levels was also reflected in the decreasing trend of the accuracy of the model (Fig. 6) associated with larger uncertainties and variabilities in both datasets. This suggests that the quality of the clinical reference can affect the model performance.

In comparison to Dataset 1, where the performance of the models remained satisfactory when tested against true clinical references, the performance of the models in Dataset 2 showed a notable drop when tested with true clinical references despite a better fit to the training dataset. Hence, this might suggest a risk of overfitting in Dataset 2. The differences in the results for the two datasets can be attributed to several factors that are intrinsic to the characteristics of each dataset. First is the “high dimensionality” Dataset 2, where there might be overly many features relative to the sample size, resulting in more complex but highly variable models that lack generalizability to external data [24, 25]. Second, Dataset 2 had more imbalanced references (clinical significance vs non-clinical significance) (80%) than Dataset 1 (49%). This imbalance may cause the models to be biased toward the majority class [26]. This imbalance can introduce challenges, causing the models to perform poorly in accurately representing the less prevalent classes, resulting in wider error bars for the performance of the models in Dataset 2. These differences underscore the influence of dataset intricacies on the model outcomes, highlighting the complex relationship between the dataset used and the model performance.

The impact of clinical reference variability and uncertainty on radiomics modelling and performance could also depend on different combinations of feature selection and classifiers. Across different permutation levels, mRMR and LASSO exhibited better stability in selecting a stable number of features than RFE or the Mann-Whitney U test. This could be owing to the intrinsic methodology of the feature selection algorithm. The emphasis of mRMR on maximizing relevance while minimizing redundancy ensures a robust selection of features that remain pertinent despite variations in clinical references. LASSO’s sparsity-inducing property, however, enables the selection of a compact set of highly informative features, contributing to its stability in the face of changing reference data. However, with the increasing number of permutated clinical references, statistical tests such as the Mann-Whitney U test could find encounter difficulties in selecting the relevant features to differentiate both groups, and RFE, which iteratively removes features based on their importance, could be sensitive to changes in the clinical reference data, leading to fluctuations in the final feature selection. This variability and uncertainty also affect the feature selection consistency, as shown by a relatively low JSC. This shows that the instability of feature selection can be found even with a low permutation level (5%), except for the Mann-Whitney U test. JSC fluctuated across different levels of clinical reference permutations and datasets. This can be attributed to the behaviour of the algorithms in response to the permutation impact. At the 45% permutation level of the clinical reference in Dataset 2, LASSO might encounter a case where it fails to select the features that could differentiate either clinical reference group owing to the high permutation level, consequently resorting to the selection of all available features. This unique circumstance led to a JSC of 1 for all repetitions at this permutation level, indicating a complete overlap in feature selection consistency across iterations, which decreased later at the 50% permutation level. This distinctive behaviour highlights sensitivity of LASSO to different permutation levels. However, despite the variabilities in feature selection, the performance of the models trained based on the permuted datasets, particularly when the permutation levels were low, appeared to be satisfactory and stable for some combinations, which might suggest the superiority of some combinations over others [8, 20], while the comparison of different feature selection and classification methods was beyond the scope of this study.

Currently, the integration of machine learning techniques, including the use of radiomics, into the medical field has become more popular, particularly for predicting treatment outcomes and improving diagnosis [4–8]. This study showed that while some uncertainties and variabilities in clinical references might be inevitable, the performance of the radiomics model demonstrated a moderate level of reliability, with around 15%-20% error rate. Machine learning classifiers rely on accurate labelling, and any uncertainties in the labelling process can impede the learning process and subsequently affect the model performance. The extent of these uncertainties varies based on the data. Ideally, minimizing these uncertainties would enhance the reliability and performance of the radiomic models. However, when uncertainties are unavoidable, acknowledging or describing the potential factors affecting clinical reference uncertainties could help in better understanding the true performance of radiomics models.

Nevertheless, this computational study had some limitations. First, it was conducted using a retrospective and simulated study design, which may introduce certain biases and limitations in terms of real-world applicability. However, the use of publicly available radiomics feature data can be helpful for reproducibility and external validation. Second, the clinical references of both datasets used in this study were binary (clinically significant vs non-clinically significant prostate tumours with a GS cutoff of seven) obtained from cross-sectional clinical data. Although binary classification is widely used in machine learning, it often oversimplifies the complexity of real-world data. The impact of clinical references on radiomics models for multiple classifications has yet to be investigated. Another important type of clinical reference is longitudinal outcomes, such as survival data in oncological studies, for which radiomics is frequently applied. Although this longitudinal outcome can be binary, its variability and uncertainty are time-dependent and may be caused by substantially different factors from those in cross-sectional studies. Hence, its impact on longitudinal radiomics modelling and performance can be individually investigated. Third, this study only selected and utilized two MRI prostate cancer datasets. Having only two datasets could limit the statistical power and generalizability of the study. Hence, a larger number of datasets, potentially accessible in the future, could provide a more robust and generalized assessment. Furthermore, the results focused on prostate cancer; thus, the results may not be straightforwardly extended to other organs, diseases, and imaging modalities. Further research is required in this area. Finally, using only four feature selection methods and two classifiers may not represent the broad range of available machine-learning algorithms used in radiomics. However, we believe that using these commonly used methods in radiomics is sufficient to provide insights into the study focus, that is, the influence of clinical reference uncertainties and variabilities on radiomics modelling and performance. Incorporating alternative robust classifiers and emerging self-supervised learning methods into MRI radiomics research represents a promising avenue for advancing analysis techniques. Other robust classifiers, such as support vector machines, gradient boosting machines, and neural networks, offer diverse modelling approaches that can capture intricate relationships within radiomics data, potentially exhibiting different performances compared with the RF and LASSO classifiers used in this study. However, self-supervised learning methods like autoencoders and variational autoencoders introduce opportunities for unsupervised feature learning and representation. By leveraging the intrinsic structure of the data, these methods can uncover patterns and relationships that may not be noticed through manual or more conventional analyses. Integrating these advanced techniques with conventional approaches and their optimization could lead to improved feature extraction, model generalization, and a deeper understanding of the underlying data characteristics in MRI radiomics studies, which exceed the scope of the current study, suggesting their potential value in future research.

## Conclusions

The impact of clinical reference uncertainties and variabilities can be substantial in MRI radiomics feature selection, modelling, and performance. The high accuracy of clinical references should be helpful in building reliable and robust radiomics models, whereas low-quality references could lead to highly variable models with high uncertainty in performance. Given the impact of clinical reference variability on model performance, it is crucial to maintain the same quality and methods for clinical references across both training and validation phases, whether internal or external.

## Supplementary Information

Supplementary Material 1.

## Data Availability

The datasets generated and/or analysed during the current study are available from previous studies [17, 18].

## Abbreviations

MRI: Magnetic resonance imaging
AUC: Area under the curve
RFE: Recursive feature elimination
mRMR: Minimum redundancy maximum relevance
LASSO: Least absolute shrinkage and selection operator
RF: Random forest
GS: Gleason score
