# Supplementary material for: Computational analysis of variability and uncertainty in the clinical reference on magnetic resonance imaging radiomics: modelling and performance
Source: Vis Comput Ind Biomed Art. 2024 Nov 19;7:28. doi: 10.1186/s42492-024-00180-9 (PMC11573982; doi:10.1186/s42492-024-00180-9)
Supplement: Supplementary file 1 — Supplementary Material 1. [file 42492_2024_180_MOESM1_ESM.docx]

Supporting Table 1 shows the feature index with the feature name in Dataset 1

| index | Feature Name (Song Dataset) |
| --- | --- |
| 1 | label |
| 2 | ADC_general_info_VolumeNum |
| 3 | ADC_general_info_VoxelNum |
| 4 | ADC_original_firstorder_10Percentile |
| 5 | ADC_original_firstorder_90Percentile |
| 6 | ADC_original_firstorder_Energy |
| 7 | ADC_original_firstorder_Entropy |
| 8 | ADC_original_firstorder_InterquartileRange |
| 9 | ADC_original_firstorder_Kurtosis |
| 10 | ADC_original_firstorder_Maximum |
| 11 | ADC_original_firstorder_Mean |
| 12 | ADC_original_firstorder_MeanAbsoluteDeviation |
| 13 | ADC_original_firstorder_Median |
| 14 | ADC_original_firstorder_Minimum |
| 15 | ADC_original_firstorder_Range |
| 16 | ADC_original_firstorder_RobustMeanAbsoluteDeviation |
| 17 | ADC_original_firstorder_RootMeanSquared |
| 18 | ADC_original_firstorder_Skewness |
| 19 | ADC_original_firstorder_TotalEnergy |
| 20 | ADC_original_firstorder_Uniformity |
| 21 | ADC_original_firstorder_Variance |
| 22 | ADC_original_glcm_Autocorrelation |
| 23 | ADC_original_glcm_ClusterProminence |
| 24 | ADC_original_glcm_ClusterShade |
| 25 | ADC_original_glcm_ClusterTendency |
| 26 | ADC_original_glcm_Contrast |
| 27 | ADC_original_glcm_Correlation |
| 28 | ADC_original_glcm_DifferenceAverage |
| 29 | ADC_original_glcm_DifferenceEntropy |
| 30 | ADC_original_glcm_DifferenceVariance |
| 31 | ADC_original_glcm_Id |
| 32 | ADC_original_glcm_Idm |
| 33 | ADC_original_glcm_Idmn |
| 34 | ADC_original_glcm_Idn |
| 35 | ADC_original_glcm_Imc1 |
| 36 | ADC_original_glcm_Imc2 |
| 37 | ADC_original_glcm_InverseVariance |
| 38 | ADC_original_glcm_JointAverage |
| 39 | ADC_original_glcm_JointEnergy |
| 40 | ADC_original_glcm_JointEntropy |
| 41 | ADC_original_glcm_MaximumProbability |
| 42 | ADC_original_glcm_SumAverage |
| 43 | ADC_original_glcm_SumEntropy |
| 44 | ADC_original_glcm_SumSquares |
| 45 | ADC_original_glrlm_GrayLevelNonUniformity |
| 46 | ADC_original_glrlm_GrayLevelNonUniformityNormalized |
| 47 | ADC_original_glrlm_GrayLevelVariance |
| 48 | ADC_original_glrlm_HighGrayLevelRunEmphasis |
| 49 | ADC_original_glrlm_LongRunEmphasis |
| 50 | ADC_original_glrlm_LongRunHighGrayLevelEmphasis |
| 51 | ADC_original_glrlm_LongRunLowGrayLevelEmphasis |
| 52 | ADC_original_glrlm_LowGrayLevelRunEmphasis |
| 53 | ADC_original_glrlm_RunEntropy |
| 54 | ADC_original_glrlm_RunLengthNonUniformity |
| 55 | ADC_original_glrlm_RunLengthNonUniformityNormalized |
| 56 | ADC_original_glrlm_RunPercentage |
| 57 | ADC_original_glrlm_RunVariance |
| 58 | ADC_original_glrlm_ShortRunEmphasis |
| 59 | ADC_original_glrlm_ShortRunHighGrayLevelEmphasis |
| 60 | ADC_original_glrlm_ShortRunLowGrayLevelEmphasis |
| 61 | ADC_original_glszm_GrayLevelNonUniformity |
| 62 | ADC_original_glszm_GrayLevelNonUniformityNormalized |
| 63 | ADC_original_glszm_GrayLevelVariance |
| 64 | ADC_original_glszm_HighGrayLevelZoneEmphasis |
| 65 | ADC_original_glszm_LargeAreaEmphasis |
| 66 | ADC_original_glszm_LargeAreaHighGrayLevelEmphasis |
| 67 | ADC_original_glszm_LargeAreaLowGrayLevelEmphasis |
| 68 | ADC_original_glszm_LowGrayLevelZoneEmphasis |
| 69 | ADC_original_glszm_SizeZoneNonUniformity |
| 70 | ADC_original_glszm_SizeZoneNonUniformityNormalized |
| 71 | ADC_original_glszm_SmallAreaEmphasis |
| 72 | ADC_original_glszm_SmallAreaHighGrayLevelEmphasis |
| 73 | ADC_original_glszm_SmallAreaLowGrayLevelEmphasis |
| 74 | ADC_original_glszm_ZoneEntropy |
| 75 | ADC_original_glszm_ZonePercentage |
| 76 | ADC_original_glszm_ZoneVariance |
| 77 | ADC_original_shape_Elongation |
| 78 | ADC_original_shape_Flatness |
| 79 | ADC_original_shape_LeastAxis |
| 80 | ADC_original_shape_MajorAxis |
| 81 | ADC_original_shape_Maximum2DDiameterColumn |
| 82 | ADC_original_shape_Maximum2DDiameterRow |
| 83 | ADC_original_shape_Maximum2DDiameterSlice |
| 84 | ADC_original_shape_Maximum3DDiameter |
| 85 | ADC_original_shape_MinorAxis |
| 86 | ADC_original_shape_Sphericity |
| 87 | ADC_original_shape_SurfaceArea |
| 88 | ADC_original_shape_SurfaceVolumeRatio |
| 89 | ADC_original_shape_Volume |
| 90 | DWI_general_info_VolumeNum |
| 91 | DWI_general_info_VoxelNum |
| 92 | DWI_original_firstorder_10Percentile |
| 93 | DWI_original_firstorder_90Percentile |
| 94 | DWI_original_firstorder_Energy |
| 95 | DWI_original_firstorder_Entropy |
| 96 | DWI_original_firstorder_InterquartileRange |
| 97 | DWI_original_firstorder_Kurtosis |
| 98 | DWI_original_firstorder_Maximum |
| 99 | DWI_original_firstorder_Mean |
| 100 | DWI_original_firstorder_MeanAbsoluteDeviation |
| 101 | DWI_original_firstorder_Median |
| 102 | DWI_original_firstorder_Minimum |
| 103 | DWI_original_firstorder_Range |
| 104 | DWI_original_firstorder_RobustMeanAbsoluteDeviation |
| 105 | DWI_original_firstorder_RootMeanSquared |
| 106 | DWI_original_firstorder_Skewness |
| 107 | DWI_original_firstorder_TotalEnergy |
| 108 | DWI_original_firstorder_Uniformity |
| 109 | DWI_original_firstorder_Variance |
| 110 | DWI_original_glcm_Autocorrelation |
| 111 | DWI_original_glcm_ClusterProminence |
| 112 | DWI_original_glcm_ClusterShade |
| 113 | DWI_original_glcm_ClusterTendency |
| 114 | DWI_original_glcm_Contrast |
| 115 | DWI_original_glcm_Correlation |
| 116 | DWI_original_glcm_DifferenceAverage |
| 117 | DWI_original_glcm_DifferenceEntropy |
| 118 | DWI_original_glcm_DifferenceVariance |
| 119 | DWI_original_glcm_Id |
| 120 | DWI_original_glcm_Idm |
| 121 | DWI_original_glcm_Idmn |
| 122 | DWI_original_glcm_Idn |
| 123 | DWI_original_glcm_Imc1 |
| 124 | DWI_original_glcm_Imc2 |
| 125 | DWI_original_glcm_InverseVariance |
| 126 | DWI_original_glcm_JointAverage |
| 127 | DWI_original_glcm_JointEnergy |
| 128 | DWI_original_glcm_JointEntropy |
| 129 | DWI_original_glcm_MaximumProbability |
| 130 | DWI_original_glcm_SumAverage |
| 131 | DWI_original_glcm_SumEntropy |
| 132 | DWI_original_glcm_SumSquares |
| 133 | DWI_original_glrlm_GrayLevelNonUniformity |
| 134 | DWI_original_glrlm_GrayLevelNonUniformityNormalized |
| 135 | DWI_original_glrlm_GrayLevelVariance |
| 136 | DWI_original_glrlm_HighGrayLevelRunEmphasis |
| 137 | DWI_original_glrlm_LongRunEmphasis |
| 138 | DWI_original_glrlm_LongRunHighGrayLevelEmphasis |
| 139 | DWI_original_glrlm_LongRunLowGrayLevelEmphasis |
| 140 | DWI_original_glrlm_LowGrayLevelRunEmphasis |
| 141 | DWI_original_glrlm_RunEntropy |
| 142 | DWI_original_glrlm_RunLengthNonUniformity |
| 143 | DWI_original_glrlm_RunLengthNonUniformityNormalized |
| 144 | DWI_original_glrlm_RunPercentage |
| 145 | DWI_original_glrlm_RunVariance |
| 146 | DWI_original_glrlm_ShortRunEmphasis |
| 147 | DWI_original_glrlm_ShortRunHighGrayLevelEmphasis |
| 148 | DWI_original_glrlm_ShortRunLowGrayLevelEmphasis |
| 149 | DWI_original_glszm_GrayLevelNonUniformity |
| 150 | DWI_original_glszm_GrayLevelNonUniformityNormalized |
| 151 | DWI_original_glszm_GrayLevelVariance |
| 152 | DWI_original_glszm_HighGrayLevelZoneEmphasis |
| 153 | DWI_original_glszm_LargeAreaEmphasis |
| 154 | DWI_original_glszm_LargeAreaHighGrayLevelEmphasis |
| 155 | DWI_original_glszm_LargeAreaLowGrayLevelEmphasis |
| 156 | DWI_original_glszm_LowGrayLevelZoneEmphasis |
| 157 | DWI_original_glszm_SizeZoneNonUniformity |
| 158 | DWI_original_glszm_SizeZoneNonUniformityNormalized |
| 159 | DWI_original_glszm_SmallAreaEmphasis |
| 160 | DWI_original_glszm_SmallAreaHighGrayLevelEmphasis |
| 161 | DWI_original_glszm_SmallAreaLowGrayLevelEmphasis |
| 162 | DWI_original_glszm_ZoneEntropy |
| 163 | DWI_original_glszm_ZonePercentage |
| 164 | DWI_original_glszm_ZoneVariance |
| 165 | DWI_original_shape_Elongation |
| 166 | DWI_original_shape_Flatness |
| 167 | DWI_original_shape_LeastAxis |
| 168 | DWI_original_shape_MajorAxis |
| 169 | DWI_original_shape_Maximum2DDiameterColumn |
| 170 | DWI_original_shape_Maximum2DDiameterRow |
| 171 | DWI_original_shape_Maximum2DDiameterSlice |
| 172 | DWI_original_shape_Maximum3DDiameter |
| 173 | DWI_original_shape_MinorAxis |
| 174 | DWI_original_shape_Sphericity |
| 175 | DWI_original_shape_SurfaceArea |
| 176 | DWI_original_shape_SurfaceVolumeRatio |
| 177 | DWI_original_shape_Volume |
| 178 | T2_general_info_VolumeNum |
| 179 | T2_general_info_VoxelNum |
| 180 | T2_original_firstorder_10Percentile |
| 181 | T2_original_firstorder_90Percentile |
| 182 | T2_original_firstorder_Energy |
| 183 | T2_original_firstorder_Entropy |
| 184 | T2_original_firstorder_InterquartileRange |
| 185 | T2_original_firstorder_Kurtosis |
| 186 | T2_original_firstorder_Maximum |
| 187 | T2_original_firstorder_Mean |
| 188 | T2_original_firstorder_MeanAbsoluteDeviation |
| 189 | T2_original_firstorder_Median |
| 190 | T2_original_firstorder_Minimum |
| 191 | T2_original_firstorder_Range |
| 192 | T2_original_firstorder_RobustMeanAbsoluteDeviation |
| 193 | T2_original_firstorder_RootMeanSquared |
| 194 | T2_original_firstorder_Skewness |
| 195 | T2_original_firstorder_TotalEnergy |
| 196 | T2_original_firstorder_Uniformity |
| 197 | T2_original_firstorder_Variance |
| 198 | T2_original_glcm_Autocorrelation |
| 199 | T2_original_glcm_ClusterProminence |
| 200 | T2_original_glcm_ClusterShade |
| 201 | T2_original_glcm_ClusterTendency |
| 202 | T2_original_glcm_Contrast |
| 203 | T2_original_glcm_Correlation |
| 204 | T2_original_glcm_DifferenceAverage |
| 205 | T2_original_glcm_DifferenceEntropy |
| 206 | T2_original_glcm_DifferenceVariance |
| 207 | T2_original_glcm_Id |
| 208 | T2_original_glcm_Idm |
| 209 | T2_original_glcm_Idmn |
| 210 | T2_original_glcm_Idn |
| 211 | T2_original_glcm_Imc1 |
| 212 | T2_original_glcm_Imc2 |
| 213 | T2_original_glcm_InverseVariance |
| 214 | T2_original_glcm_JointAverage |
| 215 | T2_original_glcm_JointEnergy |
| 216 | T2_original_glcm_JointEntropy |
| 217 | T2_original_glcm_MaximumProbability |
| 218 | T2_original_glcm_SumAverage |
| 219 | T2_original_glcm_SumEntropy |
| 220 | T2_original_glcm_SumSquares |
| 221 | T2_original_glrlm_GrayLevelNonUniformity |
| 222 | T2_original_glrlm_GrayLevelNonUniformityNormalized |
| 223 | T2_original_glrlm_GrayLevelVariance |
| 224 | T2_original_glrlm_HighGrayLevelRunEmphasis |
| 225 | T2_original_glrlm_LongRunEmphasis |
| 226 | T2_original_glrlm_LongRunHighGrayLevelEmphasis |
| 227 | T2_original_glrlm_LongRunLowGrayLevelEmphasis |
| 228 | T2_original_glrlm_LowGrayLevelRunEmphasis |
| 229 | T2_original_glrlm_RunEntropy |
| 230 | T2_original_glrlm_RunLengthNonUniformity |
| 231 | T2_original_glrlm_RunLengthNonUniformityNormalized |
| 232 | T2_original_glrlm_RunPercentage |
| 233 | T2_original_glrlm_RunVariance |
| 234 | T2_original_glrlm_ShortRunEmphasis |
| 235 | T2_original_glrlm_ShortRunHighGrayLevelEmphasis |
| 236 | T2_original_glrlm_ShortRunLowGrayLevelEmphasis |
| 237 | T2_original_glszm_GrayLevelNonUniformity |
| 238 | T2_original_glszm_GrayLevelNonUniformityNormalized |
| 239 | T2_original_glszm_GrayLevelVariance |
| 240 | T2_original_glszm_HighGrayLevelZoneEmphasis |
| 241 | T2_original_glszm_LargeAreaEmphasis |
| 242 | T2_original_glszm_LargeAreaHighGrayLevelEmphasis |
| 243 | T2_original_glszm_LargeAreaLowGrayLevelEmphasis |
| 244 | T2_original_glszm_LowGrayLevelZoneEmphasis |
| 245 | T2_original_glszm_SizeZoneNonUniformity |
| 246 | T2_original_glszm_SizeZoneNonUniformityNormalized |
| 247 | T2_original_glszm_SmallAreaEmphasis |
| 248 | T2_original_glszm_SmallAreaHighGrayLevelEmphasis |
| 249 | T2_original_glszm_SmallAreaLowGrayLevelEmphasis |
| 250 | T2_original_glszm_ZoneEntropy |
| 251 | T2_original_glszm_ZonePercentage |
| 252 | T2_original_glszm_ZoneVariance |
| 253 | T2_original_shape_Elongation |
| 254 | T2_original_shape_Flatness |
| 255 | T2_original_shape_LeastAxis |
| 256 | T2_original_shape_MajorAxis |
| 257 | T2_original_shape_Maximum2DDiameterColumn |
| 258 | T2_original_shape_Maximum2DDiameterRow |
| 259 | T2_original_shape_Maximum2DDiameterSlice |
| 260 | T2_original_shape_Maximum3DDiameter |
| 261 | T2_original_shape_MinorAxis |
| 262 | T2_original_shape_Sphericity |
| 263 | T2_original_shape_SurfaceArea |
| 264 | T2_original_shape_SurfaceVolumeRatio |
| 265 | T2_original_shape_Volume |

Supporting Table 2 shows the feature index with the feature name in Dataset 2

| index | label |
| --- | --- |
| 1 | ADCm.3.glcm.contrast.1.mean. |
| 2 | ADCm.3.glcm.contrast.1.range. |
| 3 | ADCm.3.glcm.contrast.2.mean. |
| 4 | ADCm.3.glcm.contrast.2.range. |
| 5 | ADCm.3.glcm.contrast.3.mean. |
| 6 | ADCm.3.glcm.contrast.3.range. |
| 7 | ADCm.3.glcm.dissimilarity.1.mean. |
| 8 | ADCm.3.glcm.dissimilarity.1.range. |
| 9 | ADCm.3.glcm.dissimilarity.2.mean. |
| 10 | ADCm.3.glcm.dissimilarity.2.range. |
| 11 | ADCm.3.glcm.dissimilarity.3.mean. |
| 12 | ADCm.3.glcm.dissimilarity.3.range. |
| 13 | ADCm.3.glcm.homogeneity.1.mean. |
| 14 | ADCm.3.glcm.homogeneity.1.range. |
| 15 | ADCm.3.glcm.homogeneity.2.mean. |
| 16 | ADCm.3.glcm.homogeneity.2.range. |
| 17 | ADCm.3.glcm.homogeneity.3.mean. |
| 18 | ADCm.3.glcm.homogeneity.3.range. |
| 19 | ADCm.3.glcm.energy.1.mean. |
| 20 | ADCm.3.glcm.energy.1.range. |
| 21 | ADCm.3.glcm.energy.2.mean. |
| 22 | ADCm.3.glcm.energy.2.range. |
| 23 | ADCm.3.glcm.energy.3.mean. |
| 24 | ADCm.3.glcm.energy.3.range. |
| 25 | ADCm.3.glcm.correlation.1.mean. |
| 26 | ADCm.3.glcm.correlation.1.range. |
| 27 | ADCm.3.glcm.correlation.2.mean. |
| 28 | ADCm.3.glcm.correlation.2.range. |
| 29 | ADCm.3.glcm.correlation.3.mean. |
| 30 | ADCm.3.glcm.correlation.3.range. |
| 31 | ADCm.3.glcm.ASM.1.mean. |
| 32 | ADCm.3.glcm.ASM.1.range. |
| 33 | ADCm.3.glcm.ASM.2.mean. |
| 34 | ADCm.3.glcm.ASM.2.range. |
| 35 | ADCm.3.glcm.ASM.3.mean. |
| 36 | ADCm.3.glcm.ASM.3.range. |
| 37 | ADCm.5.glcm.contrast.1.mean. |
| 38 | ADCm.5.glcm.contrast.1.range. |
| 39 | ADCm.5.glcm.contrast.2.mean. |
| 40 | ADCm.5.glcm.contrast.2.range. |
| 41 | ADCm.5.glcm.contrast.3.mean. |
| 42 | ADCm.5.glcm.contrast.3.range. |
| 43 | ADCm.5.glcm.contrast.4.mean. |
| 44 | ADCm.5.glcm.contrast.4.range. |
| 45 | ADCm.5.glcm.dissimilarity.1.mean. |
| 46 | ADCm.5.glcm.dissimilarity.1.range. |
| 47 | ADCm.5.glcm.dissimilarity.2.mean. |
| 48 | ADCm.5.glcm.dissimilarity.2.range. |
| 49 | ADCm.5.glcm.dissimilarity.3.mean. |
| 50 | ADCm.5.glcm.dissimilarity.3.range. |
| 51 | ADCm.5.glcm.dissimilarity.4.mean. |
| 52 | ADCm.5.glcm.dissimilarity.4.range. |
| 53 | ADCm.5.glcm.homogeneity.1.mean. |
| 54 | ADCm.5.glcm.homogeneity.1.range. |
| 55 | ADCm.5.glcm.homogeneity.2.mean. |
| 56 | ADCm.5.glcm.homogeneity.2.range. |
| 57 | ADCm.5.glcm.homogeneity.3.mean. |
| 58 | ADCm.5.glcm.homogeneity.3.range. |
| 59 | ADCm.5.glcm.homogeneity.4.mean. |
| 60 | ADCm.5.glcm.homogeneity.4.range. |
| 61 | ADCm.5.glcm.energy.1.mean. |
| 62 | ADCm.5.glcm.energy.1.range. |
| 63 | ADCm.5.glcm.energy.2.mean. |
| 64 | ADCm.5.glcm.energy.2.range. |
| 65 | ADCm.5.glcm.energy.3.mean. |
| 66 | ADCm.5.glcm.energy.3.range. |
| 67 | ADCm.5.glcm.energy.4.mean. |
| 68 | ADCm.5.glcm.energy.4.range. |
| 69 | ADCm.5.glcm.correlation.1.mean. |
| 70 | ADCm.5.glcm.correlation.1.range. |
| 71 | ADCm.5.glcm.correlation.2.mean. |
| 72 | ADCm.5.glcm.correlation.2.range. |
| 73 | ADCm.5.glcm.correlation.3.mean. |
| 74 | ADCm.5.glcm.correlation.3.range. |
| 75 | ADCm.5.glcm.correlation.4.mean. |
| 76 | ADCm.5.glcm.correlation.4.range. |
| 77 | ADCm.5.glcm.ASM.1.mean. |
| 78 | ADCm.5.glcm.ASM.1.range. |
| 79 | ADCm.5.glcm.ASM.2.mean. |
| 80 | ADCm.5.glcm.ASM.2.range. |
| 81 | ADCm.5.glcm.ASM.3.mean. |
| 82 | ADCm.5.glcm.ASM.3.range. |
| 83 | ADCm.5.glcm.ASM.4.mean. |
| 84 | ADCm.5.glcm.ASM.4.range. |
| 85 | ADCm.7.glcm.contrast.1.mean. |
| 86 | ADCm.7.glcm.contrast.1.range. |
| 87 | ADCm.7.glcm.contrast.2.mean. |
| 88 | ADCm.7.glcm.contrast.2.range. |
| 89 | ADCm.7.glcm.contrast.3.mean. |
| 90 | ADCm.7.glcm.contrast.3.range. |
| 91 | ADCm.7.glcm.contrast.4.mean. |
| 92 | ADCm.7.glcm.contrast.4.range. |
| 93 | ADCm.7.glcm.dissimilarity.1.mean. |
| 94 | ADCm.7.glcm.dissimilarity.1.range. |
| 95 | ADCm.7.glcm.dissimilarity.2.mean. |
| 96 | ADCm.7.glcm.dissimilarity.2.range. |
| 97 | ADCm.7.glcm.dissimilarity.3.mean. |
| 98 | ADCm.7.glcm.dissimilarity.3.range. |
| 99 | ADCm.7.glcm.dissimilarity.4.mean. |
| 100 | ADCm.7.glcm.dissimilarity.4.range. |
| 101 | ADCm.7.glcm.homogeneity.1.mean. |
| 102 | ADCm.7.glcm.homogeneity.1.range. |
| 103 | ADCm.7.glcm.homogeneity.2.mean. |
| 104 | ADCm.7.glcm.homogeneity.2.range. |
| 105 | ADCm.7.glcm.homogeneity.3.mean. |
| 106 | ADCm.7.glcm.homogeneity.3.range. |
| 107 | ADCm.7.glcm.homogeneity.4.mean. |
| 108 | ADCm.7.glcm.homogeneity.4.range. |
| 109 | ADCm.7.glcm.energy.1.mean. |
| 110 | ADCm.7.glcm.energy.1.range. |
| 111 | ADCm.7.glcm.energy.2.mean. |
| 112 | ADCm.7.glcm.energy.2.range. |
| 113 | ADCm.7.glcm.energy.3.mean. |
| 114 | ADCm.7.glcm.energy.3.range. |
| 115 | ADCm.7.glcm.energy.4.mean. |
| 116 | ADCm.7.glcm.energy.4.range. |
| 117 | ADCm.7.glcm.correlation.1.mean. |
| 118 | ADCm.7.glcm.correlation.1.range. |
| 119 | ADCm.7.glcm.correlation.2.mean. |
| 120 | ADCm.7.glcm.correlation.2.range. |
| 121 | ADCm.7.glcm.correlation.3.mean. |
| 122 | ADCm.7.glcm.correlation.3.range. |
| 123 | ADCm.7.glcm.correlation.4.mean. |
| 124 | ADCm.7.glcm.correlation.4.range. |
| 125 | ADCm.7.glcm.ASM.1.mean. |
| 126 | ADCm.7.glcm.ASM.1.range. |
| 127 | ADCm.7.glcm.ASM.2.mean. |
| 128 | ADCm.7.glcm.ASM.2.range. |
| 129 | ADCm.7.glcm.ASM.3.mean. |
| 130 | ADCm.7.glcm.ASM.3.range. |
| 131 | ADCm.7.glcm.ASM.4.mean. |
| 132 | ADCm.7.glcm.ASM.4.range. |
| 133 | ADCm.9.glcm.contrast.1.mean. |
| 134 | ADCm.9.glcm.contrast.1.range. |
| 135 | ADCm.9.glcm.contrast.2.mean. |
| 136 | ADCm.9.glcm.contrast.2.range. |
| 137 | ADCm.9.glcm.contrast.3.mean. |
| 138 | ADCm.9.glcm.contrast.3.range. |
| 139 | ADCm.9.glcm.contrast.4.mean. |
| 140 | ADCm.9.glcm.contrast.4.range. |
| 141 | ADCm.9.glcm.dissimilarity.1.mean. |
| 142 | ADCm.9.glcm.dissimilarity.1.range. |
| 143 | ADCm.9.glcm.dissimilarity.2.mean. |
| 144 | ADCm.9.glcm.dissimilarity.2.range. |
| 145 | ADCm.9.glcm.dissimilarity.3.mean. |
| 146 | ADCm.9.glcm.dissimilarity.3.range. |
| 147 | ADCm.9.glcm.dissimilarity.4.mean. |
| 148 | ADCm.9.glcm.dissimilarity.4.range. |
| 149 | ADCm.9.glcm.homogeneity.1.mean. |
| 150 | ADCm.9.glcm.homogeneity.1.range. |
| 151 | ADCm.9.glcm.homogeneity.2.mean. |
| 152 | ADCm.9.glcm.homogeneity.2.range. |
| 153 | ADCm.9.glcm.homogeneity.3.mean. |
| 154 | ADCm.9.glcm.homogeneity.3.range. |
| 155 | ADCm.9.glcm.homogeneity.4.mean. |
| 156 | ADCm.9.glcm.homogeneity.4.range. |
| 157 | ADCm.9.glcm.energy.1.mean. |
| 158 | ADCm.9.glcm.energy.1.range. |
| 159 | ADCm.9.glcm.energy.2.mean. |
| 160 | ADCm.9.glcm.energy.2.range. |
| 161 | ADCm.9.glcm.energy.3.mean. |
| 162 | ADCm.9.glcm.energy.3.range. |
| 163 | ADCm.9.glcm.energy.4.mean. |
| 164 | ADCm.9.glcm.energy.4.range. |
| 165 | ADCm.9.glcm.correlation.1.mean. |
| 166 | ADCm.9.glcm.correlation.1.range. |
| 167 | ADCm.9.glcm.correlation.2.mean. |
| 168 | ADCm.9.glcm.correlation.2.range. |
| 169 | ADCm.9.glcm.correlation.3.mean. |
| 170 | ADCm.9.glcm.correlation.3.range. |
| 171 | ADCm.9.glcm.correlation.4.mean. |
| 172 | ADCm.9.glcm.correlation.4.range. |
| 173 | ADCm.9.glcm.ASM.1.mean. |
| 174 | ADCm.9.glcm.ASM.1.range. |
| 175 | ADCm.9.glcm.ASM.2.mean. |
| 176 | ADCm.9.glcm.ASM.2.range. |
| 177 | ADCm.9.glcm.ASM.3.mean. |
| 178 | ADCm.9.glcm.ASM.3.range. |
| 179 | ADCm.9.glcm.ASM.4.mean. |
| 180 | ADCm.9.glcm.ASM.4.range. |
| 181 | ADCm.11.glcm.contrast.1.mean. |
| 182 | ADCm.11.glcm.contrast.1.range. |
| 183 | ADCm.11.glcm.contrast.2.mean. |
| 184 | ADCm.11.glcm.contrast.2.range. |
| 185 | ADCm.11.glcm.contrast.3.mean. |
| 186 | ADCm.11.glcm.contrast.3.range. |
| 187 | ADCm.11.glcm.contrast.4.mean. |
| 188 | ADCm.11.glcm.contrast.4.range. |
| 189 | ADCm.11.glcm.dissimilarity.1.mean. |
| 190 | ADCm.11.glcm.dissimilarity.1.range. |
| 191 | ADCm.11.glcm.dissimilarity.2.mean. |
| 192 | ADCm.11.glcm.dissimilarity.2.range. |
| 193 | ADCm.11.glcm.dissimilarity.3.mean. |
| 194 | ADCm.11.glcm.dissimilarity.3.range. |
| 195 | ADCm.11.glcm.dissimilarity.4.mean. |
| 196 | ADCm.11.glcm.dissimilarity.4.range. |
| 197 | ADCm.11.glcm.homogeneity.1.mean. |
| 198 | ADCm.11.glcm.homogeneity.1.range. |
| 199 | ADCm.11.glcm.homogeneity.2.mean. |
| 200 | ADCm.11.glcm.homogeneity.2.range. |
| 201 | ADCm.11.glcm.homogeneity.3.mean. |
| 202 | ADCm.11.glcm.homogeneity.3.range. |
| 203 | ADCm.11.glcm.homogeneity.4.mean. |
| 204 | ADCm.11.glcm.homogeneity.4.range. |
| 205 | ADCm.11.glcm.energy.1.mean. |
| 206 | ADCm.11.glcm.energy.1.range. |
| 207 | ADCm.11.glcm.energy.2.mean. |
| 208 | ADCm.11.glcm.energy.2.range. |
| 209 | ADCm.11.glcm.energy.3.mean. |
| 210 | ADCm.11.glcm.energy.3.range. |
| 211 | ADCm.11.glcm.energy.4.mean. |
| 212 | ADCm.11.glcm.energy.4.range. |
| 213 | ADCm.11.glcm.correlation.1.mean. |
| 214 | ADCm.11.glcm.correlation.1.range. |
| 215 | ADCm.11.glcm.correlation.2.mean. |
| 216 | ADCm.11.glcm.correlation.2.range. |
| 217 | ADCm.11.glcm.correlation.3.mean. |
| 218 | ADCm.11.glcm.correlation.3.range. |
| 219 | ADCm.11.glcm.correlation.4.mean. |
| 220 | ADCm.11.glcm.correlation.4.range. |
| 221 | ADCm.11.glcm.ASM.1.mean. |
| 222 | ADCm.11.glcm.ASM.1.range. |
| 223 | ADCm.11.glcm.ASM.2.mean. |
| 224 | ADCm.11.glcm.ASM.2.range. |
| 225 | ADCm.11.glcm.ASM.3.mean. |
| 226 | ADCm.11.glcm.ASM.3.range. |
| 227 | ADCm.11.glcm.ASM.4.mean. |
| 228 | ADCm.11.glcm.ASM.4.range. |
| 229 | ADCm.13.glcm.contrast.1.mean. |
| 230 | ADCm.13.glcm.contrast.1.range. |
| 231 | ADCm.13.glcm.contrast.2.mean. |
| 232 | ADCm.13.glcm.contrast.2.range. |
| 233 | ADCm.13.glcm.contrast.3.mean. |
| 234 | ADCm.13.glcm.contrast.3.range. |
| 235 | ADCm.13.glcm.contrast.4.mean. |
| 236 | ADCm.13.glcm.contrast.4.range. |
| 237 | ADCm.13.glcm.dissimilarity.1.mean. |
| 238 | ADCm.13.glcm.dissimilarity.1.range. |
| 239 | ADCm.13.glcm.dissimilarity.2.mean. |
| 240 | ADCm.13.glcm.dissimilarity.2.range. |
| 241 | ADCm.13.glcm.dissimilarity.3.mean. |
| 242 | ADCm.13.glcm.dissimilarity.3.range. |
| 243 | ADCm.13.glcm.dissimilarity.4.mean. |
| 244 | ADCm.13.glcm.dissimilarity.4.range. |
| 245 | ADCm.13.glcm.homogeneity.1.mean. |
| 246 | ADCm.13.glcm.homogeneity.1.range. |
| 247 | ADCm.13.glcm.homogeneity.2.mean. |
| 248 | ADCm.13.glcm.homogeneity.2.range. |
| 249 | ADCm.13.glcm.homogeneity.3.mean. |
| 250 | ADCm.13.glcm.homogeneity.3.range. |
| 251 | ADCm.13.glcm.homogeneity.4.mean. |
| 252 | ADCm.13.glcm.homogeneity.4.range. |
| 253 | ADCm.13.glcm.energy.1.mean. |
| 254 | ADCm.13.glcm.energy.1.range. |
| 255 | ADCm.13.glcm.energy.2.mean. |
| 256 | ADCm.13.glcm.energy.2.range. |
| 257 | ADCm.13.glcm.energy.3.mean. |
| 258 | ADCm.13.glcm.energy.3.range. |
| 259 | ADCm.13.glcm.energy.4.mean. |
| 260 | ADCm.13.glcm.energy.4.range. |
| 261 | ADCm.13.glcm.correlation.1.mean. |
| 262 | ADCm.13.glcm.correlation.1.range. |
| 263 | ADCm.13.glcm.correlation.2.mean. |
| 264 | ADCm.13.glcm.correlation.2.range. |
| 265 | ADCm.13.glcm.correlation.3.mean. |
| 266 | ADCm.13.glcm.correlation.3.range. |
| 267 | ADCm.13.glcm.correlation.4.mean. |
| 268 | ADCm.13.glcm.correlation.4.range. |
| 269 | ADCm.13.glcm.ASM.1.mean. |
| 270 | ADCm.13.glcm.ASM.1.range. |
| 271 | ADCm.13.glcm.ASM.2.mean. |
| 272 | ADCm.13.glcm.ASM.2.range. |
| 273 | ADCm.13.glcm.ASM.3.mean. |
| 274 | ADCm.13.glcm.ASM.3.range. |
| 275 | ADCm.13.glcm.ASM.4.mean. |
| 276 | ADCm.13.glcm.ASM.4.range. |
| 277 | ADCm.15.glcm.contrast.1.mean. |
| 278 | ADCm.15.glcm.contrast.1.range. |
| 279 | ADCm.15.glcm.contrast.2.mean. |
| 280 | ADCm.15.glcm.contrast.2.range. |
| 281 | ADCm.15.glcm.contrast.3.mean. |
| 282 | ADCm.15.glcm.contrast.3.range. |
| 283 | ADCm.15.glcm.contrast.4.mean. |
| 284 | ADCm.15.glcm.contrast.4.range. |
| 285 | ADCm.15.glcm.dissimilarity.1.mean. |
| 286 | ADCm.15.glcm.dissimilarity.1.range. |
| 287 | ADCm.15.glcm.dissimilarity.2.mean. |
| 288 | ADCm.15.glcm.dissimilarity.2.range. |
| 289 | ADCm.15.glcm.dissimilarity.3.mean. |
| 290 | ADCm.15.glcm.dissimilarity.3.range. |
| 291 | ADCm.15.glcm.dissimilarity.4.mean. |
| 292 | ADCm.15.glcm.dissimilarity.4.range. |
| 293 | ADCm.15.glcm.homogeneity.1.mean. |
| 294 | ADCm.15.glcm.homogeneity.1.range. |
| 295 | ADCm.15.glcm.homogeneity.2.mean. |
| 296 | ADCm.15.glcm.homogeneity.2.range. |
| 297 | ADCm.15.glcm.homogeneity.3.mean. |
| 298 | ADCm.15.glcm.homogeneity.3.range. |
| 299 | ADCm.15.glcm.homogeneity.4.mean. |
| 300 | ADCm.15.glcm.homogeneity.4.range. |
| 301 | ADCm.15.glcm.energy.1.mean. |
| 302 | ADCm.15.glcm.energy.1.range. |
| 303 | ADCm.15.glcm.energy.2.mean. |
| 304 | ADCm.15.glcm.energy.2.range. |
| 305 | ADCm.15.glcm.energy.3.mean. |
| 306 | ADCm.15.glcm.energy.3.range. |
| 307 | ADCm.15.glcm.energy.4.mean. |
| 308 | ADCm.15.glcm.energy.4.range. |
| 309 | ADCm.15.glcm.correlation.1.mean. |
| 310 | ADCm.15.glcm.correlation.1.range. |
| 311 | ADCm.15.glcm.correlation.2.mean. |
| 312 | ADCm.15.glcm.correlation.2.range. |
| 313 | ADCm.15.glcm.correlation.3.mean. |
| 314 | ADCm.15.glcm.correlation.3.range. |
| 315 | ADCm.15.glcm.correlation.4.mean. |
| 316 | ADCm.15.glcm.correlation.4.range. |
| 317 | ADCm.15.glcm.ASM.1.mean. |
| 318 | ADCm.15.glcm.ASM.1.range. |
| 319 | ADCm.15.glcm.ASM.2.mean. |
| 320 | ADCm.15.glcm.ASM.2.range. |
| 321 | ADCm.15.glcm.ASM.3.mean. |
| 322 | ADCm.15.glcm.ASM.3.range. |
| 323 | ADCm.15.glcm.ASM.4.mean. |
| 324 | ADCm.15.glcm.ASM.4.range. |
| 325 | ADCm.mbb.glcm.contrast.1.mean. |
| 326 | ADCm.mbb.glcm.contrast.1.range. |
| 327 | ADCm.mbb.glcm.contrast.2.mean. |
| 328 | ADCm.mbb.glcm.contrast.2.range. |
| 329 | ADCm.mbb.glcm.contrast.3.mean. |
| 330 | ADCm.mbb.glcm.contrast.3.range. |
| 331 | ADCm.mbb.glcm.contrast.4.mean. |
| 332 | ADCm.mbb.glcm.contrast.4.range. |
| 333 | ADCm.mbb.glcm.dissimilarity.1.mean. |
| 334 | ADCm.mbb.glcm.dissimilarity.1.range. |
| 335 | ADCm.mbb.glcm.dissimilarity.2.mean. |
| 336 | ADCm.mbb.glcm.dissimilarity.2.range. |
| 337 | ADCm.mbb.glcm.dissimilarity.3.mean. |
| 338 | ADCm.mbb.glcm.dissimilarity.3.range. |
| 339 | ADCm.mbb.glcm.dissimilarity.4.mean. |
| 340 | ADCm.mbb.glcm.dissimilarity.4.range. |
| 341 | ADCm.mbb.glcm.homogeneity.1.mean. |
| 342 | ADCm.mbb.glcm.homogeneity.1.range. |
| 343 | ADCm.mbb.glcm.homogeneity.2.mean. |
| 344 | ADCm.mbb.glcm.homogeneity.2.range. |
| 345 | ADCm.mbb.glcm.homogeneity.3.mean. |
| 346 | ADCm.mbb.glcm.homogeneity.3.range. |
| 347 | ADCm.mbb.glcm.homogeneity.4.mean. |
| 348 | ADCm.mbb.glcm.homogeneity.4.range. |
| 349 | ADCm.mbb.glcm.energy.1.mean. |
| 350 | ADCm.mbb.glcm.energy.1.range. |
| 351 | ADCm.mbb.glcm.energy.2.mean. |
| 352 | ADCm.mbb.glcm.energy.2.range. |
| 353 | ADCm.mbb.glcm.energy.3.mean. |
| 354 | ADCm.mbb.glcm.energy.3.range. |
| 355 | ADCm.mbb.glcm.energy.4.mean. |
| 356 | ADCm.mbb.glcm.energy.4.range. |
| 357 | ADCm.mbb.glcm.correlation.1.mean. |
| 358 | ADCm.mbb.glcm.correlation.1.range. |
| 359 | ADCm.mbb.glcm.correlation.2.mean. |
| 360 | ADCm.mbb.glcm.correlation.2.range. |
| 361 | ADCm.mbb.glcm.correlation.3.mean. |
| 362 | ADCm.mbb.glcm.correlation.3.range. |
| 363 | ADCm.mbb.glcm.correlation.4.mean. |
| 364 | ADCm.mbb.glcm.correlation.4.range. |
| 365 | ADCm.mbb.glcm.ASM.1.mean. |
| 366 | ADCm.mbb.glcm.ASM.1.range. |
| 367 | ADCm.mbb.glcm.ASM.2.mean. |
| 368 | ADCm.mbb.glcm.ASM.2.range. |
| 369 | ADCm.mbb.glcm.ASM.3.mean. |
| 370 | ADCm.mbb.glcm.ASM.3.range. |
| 371 | ADCm.mbb.glcm.ASM.4.mean. |
| 372 | ADCm.mbb.glcm.ASM.4.range. |
| 373 | ADCm.3.lbp.1.0. |
| 374 | ADCm.3.lbp.1.1. |
| 375 | ADCm.3.lbp.1.2. |
| 376 | ADCm.3.lbp.1.3. |
| 377 | ADCm.3.lbp.1.4. |
| 378 | ADCm.3.lbp.1.5. |
| 379 | ADCm.3.lbp.1.6. |
| 380 | ADCm.3.lbp.1.7. |
| 381 | ADCm.3.lbp.1.8. |
| 382 | ADCm.3.lbp.1.9. |
| 383 | ADCm.5.lbp.2.0. |
| 384 | ADCm.5.lbp.2.1. |
| 385 | ADCm.5.lbp.2.2. |
| 386 | ADCm.5.lbp.2.3. |
| 387 | ADCm.5.lbp.2.4. |
| 388 | ADCm.5.lbp.2.5. |
| 389 | ADCm.5.lbp.2.6. |
| 390 | ADCm.5.lbp.2.7. |
| 391 | ADCm.5.lbp.2.8. |
| 392 | ADCm.5.lbp.2.9. |
| 393 | ADCm.7.lbp.3.0. |
| 394 | ADCm.7.lbp.3.1. |
| 395 | ADCm.7.lbp.3.2. |
| 396 | ADCm.7.lbp.3.3. |
| 397 | ADCm.7.lbp.3.4. |
| 398 | ADCm.7.lbp.3.5. |
| 399 | ADCm.7.lbp.3.6. |
| 400 | ADCm.7.lbp.3.7. |
| 401 | ADCm.7.lbp.3.8. |
| 402 | ADCm.7.lbp.3.9. |
| 403 | ADCm.9.lbp.4.0. |
| 404 | ADCm.9.lbp.4.1. |
| 405 | ADCm.9.lbp.4.2. |
| 406 | ADCm.9.lbp.4.3. |
| 407 | ADCm.9.lbp.4.4. |
| 408 | ADCm.9.lbp.4.5. |
| 409 | ADCm.9.lbp.4.6. |
| 410 | ADCm.9.lbp.4.7. |
| 411 | ADCm.9.lbp.4.8. |
| 412 | ADCm.9.lbp.4.9. |
| 413 | ADCm.11.lbp.5.0. |
| 414 | ADCm.11.lbp.5.1. |
| 415 | ADCm.11.lbp.5.2. |
| 416 | ADCm.11.lbp.5.3. |
| 417 | ADCm.11.lbp.5.4. |
| 418 | ADCm.11.lbp.5.5. |
| 419 | ADCm.11.lbp.5.6. |
| 420 | ADCm.11.lbp.5.7. |
| 421 | ADCm.11.lbp.5.8. |
| 422 | ADCm.11.lbp.5.9. |
| 423 | ADCm.13.lbp.6.0. |
| 424 | ADCm.13.lbp.6.1. |
| 425 | ADCm.13.lbp.6.2. |
| 426 | ADCm.13.lbp.6.3. |
| 427 | ADCm.13.lbp.6.4. |
| 428 | ADCm.13.lbp.6.5. |
| 429 | ADCm.13.lbp.6.6. |
| 430 | ADCm.13.lbp.6.7. |
| 431 | ADCm.13.lbp.6.8. |
| 432 | ADCm.13.lbp.6.9. |
| 433 | ADCm.15.lbp.7.0. |
| 434 | ADCm.15.lbp.7.1. |
| 435 | ADCm.15.lbp.7.2. |
| 436 | ADCm.15.lbp.7.3. |
| 437 | ADCm.15.lbp.7.4. |
| 438 | ADCm.15.lbp.7.5. |
| 439 | ADCm.15.lbp.7.6. |
| 440 | ADCm.15.lbp.7.7. |
| 441 | ADCm.15.lbp.7.8. |
| 442 | ADCm.15.lbp.7.9. |
| 443 | ADCm.3.hog |
| 444 | ADCm.5.hog |
| 445 | ADCm.7.hog |
| 446 | ADCm.9.hog |
| 447 | ADCm.11.hog |
| 448 | ADCm.13.hog |
| 449 | ADCm.15.hog |
| 450 | ADCm.3.gabor.1.0.1.mean. |
| 451 | ADCm.3.gabor.1.0.1.var. |
| 452 | ADCm.3.gabor.1.0.1.absmean. |
| 453 | ADCm.3.gabor.1.0.1.mag. |
| 454 | ADCm.3.gabor.1.0.2.mean. |
| 455 | ADCm.3.gabor.1.0.2.var. |
| 456 | ADCm.3.gabor.1.0.2.absmean. |
| 457 | ADCm.3.gabor.1.0.2.mag. |
| 458 | ADCm.3.gabor.1.0.3.mean. |
| 459 | ADCm.3.gabor.1.0.3.var. |
| 460 | ADCm.3.gabor.1.0.3.absmean. |
| 461 | ADCm.3.gabor.1.0.3.mag. |
| 462 | ADCm.3.gabor.1.0.4.mean. |
| 463 | ADCm.3.gabor.1.0.4.var. |
| 464 | ADCm.3.gabor.1.0.4.absmean. |
| 465 | ADCm.3.gabor.1.0.4.mag. |
| 466 | ADCm.3.gabor.1.0.5.mean. |
| 467 | ADCm.3.gabor.1.0.5.var. |
| 468 | ADCm.3.gabor.1.0.5.absmean. |
| 469 | ADCm.3.gabor.1.0.5.mag. |
| 470 | ADCm.3.gabor.2.0.1.mean. |
| 471 | ADCm.3.gabor.2.0.1.var. |
| 472 | ADCm.3.gabor.2.0.1.absmean. |
| 473 | ADCm.3.gabor.2.0.1.mag. |
| 474 | ADCm.3.gabor.2.0.2.mean. |
| 475 | ADCm.3.gabor.2.0.2.var. |
| 476 | ADCm.3.gabor.2.0.2.absmean. |
| 477 | ADCm.3.gabor.2.0.2.mag. |
| 478 | ADCm.3.gabor.2.0.3.mean. |
| 479 | ADCm.3.gabor.2.0.3.var. |
| 480 | ADCm.3.gabor.2.0.3.absmean. |
| 481 | ADCm.3.gabor.2.0.3.mag. |
| 482 | ADCm.3.gabor.2.0.4.mean. |
| 483 | ADCm.3.gabor.2.0.4.var. |
| 484 | ADCm.3.gabor.2.0.4.absmean. |
| 485 | ADCm.3.gabor.2.0.4.mag. |
| 486 | ADCm.3.gabor.2.0.5.mean. |
| 487 | ADCm.3.gabor.2.0.5.var. |
| 488 | ADCm.3.gabor.2.0.5.absmean. |
| 489 | ADCm.3.gabor.2.0.5.mag. |
| 490 | ADCm.3.gabor.3.0.1.mean. |
| 491 | ADCm.3.gabor.3.0.1.var. |
| 492 | ADCm.3.gabor.3.0.1.absmean. |
| 493 | ADCm.3.gabor.3.0.1.mag. |
| 494 | ADCm.3.gabor.3.0.2.mean. |
| 495 | ADCm.3.gabor.3.0.2.var. |
| 496 | ADCm.3.gabor.3.0.2.absmean. |
| 497 | ADCm.3.gabor.3.0.2.mag. |
| 498 | ADCm.3.gabor.3.0.3.mean. |
| 499 | ADCm.3.gabor.3.0.3.var. |
| 500 | ADCm.3.gabor.3.0.3.absmean. |
| 501 | ADCm.3.gabor.3.0.3.mag. |
| 502 | ADCm.3.gabor.3.0.4.mean. |
| 503 | ADCm.3.gabor.3.0.4.var. |
| 504 | ADCm.3.gabor.3.0.4.absmean. |
| 505 | ADCm.3.gabor.3.0.4.mag. |
| 506 | ADCm.3.gabor.3.0.5.mean. |
| 507 | ADCm.3.gabor.3.0.5.var. |
| 508 | ADCm.3.gabor.3.0.5.absmean. |
| 509 | ADCm.3.gabor.3.0.5.mag. |
| 510 | ADCm.5.gabor.1.0.1.mean. |
| 511 | ADCm.5.gabor.1.0.1.var. |
| 512 | ADCm.5.gabor.1.0.1.absmean. |
| 513 | ADCm.5.gabor.1.0.1.mag. |
| 514 | ADCm.5.gabor.1.0.2.mean. |
| 515 | ADCm.5.gabor.1.0.2.var. |
| 516 | ADCm.5.gabor.1.0.2.absmean. |
| 517 | ADCm.5.gabor.1.0.2.mag. |
| 518 | ADCm.5.gabor.1.0.3.mean. |
| 519 | ADCm.5.gabor.1.0.3.var. |
| 520 | ADCm.5.gabor.1.0.3.absmean. |
| 521 | ADCm.5.gabor.1.0.3.mag. |
| 522 | ADCm.5.gabor.1.0.4.mean. |
| 523 | ADCm.5.gabor.1.0.4.var. |
| 524 | ADCm.5.gabor.1.0.4.absmean. |
| 525 | ADCm.5.gabor.1.0.4.mag. |
| 526 | ADCm.5.gabor.1.0.5.mean. |
| 527 | ADCm.5.gabor.1.0.5.var. |
| 528 | ADCm.5.gabor.1.0.5.absmean. |
| 529 | ADCm.5.gabor.1.0.5.mag. |
| 530 | ADCm.5.gabor.2.0.1.mean. |
| 531 | ADCm.5.gabor.2.0.1.var. |
| 532 | ADCm.5.gabor.2.0.1.absmean. |
| 533 | ADCm.5.gabor.2.0.1.mag. |
| 534 | ADCm.5.gabor.2.0.2.mean. |
| 535 | ADCm.5.gabor.2.0.2.var. |
| 536 | ADCm.5.gabor.2.0.2.absmean. |
| 537 | ADCm.5.gabor.2.0.2.mag. |
| 538 | ADCm.5.gabor.2.0.3.mean. |
| 539 | ADCm.5.gabor.2.0.3.var. |
| 540 | ADCm.5.gabor.2.0.3.absmean. |
| 541 | ADCm.5.gabor.2.0.3.mag. |
| 542 | ADCm.5.gabor.2.0.4.mean. |
| 543 | ADCm.5.gabor.2.0.4.var. |
| 544 | ADCm.5.gabor.2.0.4.absmean. |
| 545 | ADCm.5.gabor.2.0.4.mag. |
| 546 | ADCm.5.gabor.2.0.5.mean. |
| 547 | ADCm.5.gabor.2.0.5.var. |
| 548 | ADCm.5.gabor.2.0.5.absmean. |
| 549 | ADCm.5.gabor.2.0.5.mag. |
| 550 | ADCm.5.gabor.3.0.1.mean. |
| 551 | ADCm.5.gabor.3.0.1.var. |
| 552 | ADCm.5.gabor.3.0.1.absmean. |
| 553 | ADCm.5.gabor.3.0.1.mag. |
| 554 | ADCm.5.gabor.3.0.2.mean. |
| 555 | ADCm.5.gabor.3.0.2.var. |
| 556 | ADCm.5.gabor.3.0.2.absmean. |
| 557 | ADCm.5.gabor.3.0.2.mag. |
| 558 | ADCm.5.gabor.3.0.3.mean. |
| 559 | ADCm.5.gabor.3.0.3.var. |
| 560 | ADCm.5.gabor.3.0.3.absmean. |
| 561 | ADCm.5.gabor.3.0.3.mag. |
| 562 | ADCm.5.gabor.3.0.4.mean. |
| 563 | ADCm.5.gabor.3.0.4.var. |
| 564 | ADCm.5.gabor.3.0.4.absmean. |
| 565 | ADCm.5.gabor.3.0.4.mag. |
| 566 | ADCm.5.gabor.3.0.5.mean. |
| 567 | ADCm.5.gabor.3.0.5.var. |
| 568 | ADCm.5.gabor.3.0.5.absmean. |
| 569 | ADCm.5.gabor.3.0.5.mag. |
| 570 | ADCm.7.gabor.1.0.1.mean. |
| 571 | ADCm.7.gabor.1.0.1.var. |
| 572 | ADCm.7.gabor.1.0.1.absmean. |
| 573 | ADCm.7.gabor.1.0.1.mag. |
| 574 | ADCm.7.gabor.1.0.2.mean. |
| 575 | ADCm.7.gabor.1.0.2.var. |
| 576 | ADCm.7.gabor.1.0.2.absmean. |
| 577 | ADCm.7.gabor.1.0.2.mag. |
| 578 | ADCm.7.gabor.1.0.3.mean. |
| 579 | ADCm.7.gabor.1.0.3.var. |
| 580 | ADCm.7.gabor.1.0.3.absmean. |
| 581 | ADCm.7.gabor.1.0.3.mag. |
| 582 | ADCm.7.gabor.1.0.4.mean. |
| 583 | ADCm.7.gabor.1.0.4.var. |
| 584 | ADCm.7.gabor.1.0.4.absmean. |
| 585 | ADCm.7.gabor.1.0.4.mag. |
| 586 | ADCm.7.gabor.1.0.5.mean. |
| 587 | ADCm.7.gabor.1.0.5.var. |
| 588 | ADCm.7.gabor.1.0.5.absmean. |
| 589 | ADCm.7.gabor.1.0.5.mag. |
| 590 | ADCm.7.gabor.2.0.1.mean. |
| 591 | ADCm.7.gabor.2.0.1.var. |
| 592 | ADCm.7.gabor.2.0.1.absmean. |
| 593 | ADCm.7.gabor.2.0.1.mag. |
| 594 | ADCm.7.gabor.2.0.2.mean. |
| 595 | ADCm.7.gabor.2.0.2.var. |
| 596 | ADCm.7.gabor.2.0.2.absmean. |
| 597 | ADCm.7.gabor.2.0.2.mag. |
| 598 | ADCm.7.gabor.2.0.3.mean. |
| 599 | ADCm.7.gabor.2.0.3.var. |
| 600 | ADCm.7.gabor.2.0.3.absmean. |
| 601 | ADCm.7.gabor.2.0.3.mag. |
| 602 | ADCm.7.gabor.2.0.4.mean. |
| 603 | ADCm.7.gabor.2.0.4.var. |
| 604 | ADCm.7.gabor.2.0.4.absmean. |
| 605 | ADCm.7.gabor.2.0.4.mag. |
| 606 | ADCm.7.gabor.2.0.5.mean. |
| 607 | ADCm.7.gabor.2.0.5.var. |
| 608 | ADCm.7.gabor.2.0.5.absmean. |
| 609 | ADCm.7.gabor.2.0.5.mag. |
| 610 | ADCm.7.gabor.3.0.1.mean. |
| 611 | ADCm.7.gabor.3.0.1.var. |
| 612 | ADCm.7.gabor.3.0.1.absmean. |
| 613 | ADCm.7.gabor.3.0.1.mag. |
| 614 | ADCm.7.gabor.3.0.2.mean. |
| 615 | ADCm.7.gabor.3.0.2.var. |
| 616 | ADCm.7.gabor.3.0.2.absmean. |
| 617 | ADCm.7.gabor.3.0.2.mag. |
| 618 | ADCm.7.gabor.3.0.3.mean. |
| 619 | ADCm.7.gabor.3.0.3.var. |
| 620 | ADCm.7.gabor.3.0.3.absmean. |
| 621 | ADCm.7.gabor.3.0.3.mag. |
| 622 | ADCm.7.gabor.3.0.4.mean. |
| 623 | ADCm.7.gabor.3.0.4.var. |
| 624 | ADCm.7.gabor.3.0.4.absmean. |
| 625 | ADCm.7.gabor.3.0.4.mag. |
| 626 | ADCm.7.gabor.3.0.5.mean. |
| 627 | ADCm.7.gabor.3.0.5.var. |
| 628 | ADCm.7.gabor.3.0.5.absmean. |
| 629 | ADCm.7.gabor.3.0.5.mag. |
| 630 | ADCm.9.gabor.1.0.1.mean. |
| 631 | ADCm.9.gabor.1.0.1.var. |
| 632 | ADCm.9.gabor.1.0.1.absmean. |
| 633 | ADCm.9.gabor.1.0.1.mag. |
| 634 | ADCm.9.gabor.1.0.2.mean. |
| 635 | ADCm.9.gabor.1.0.2.var. |
| 636 | ADCm.9.gabor.1.0.2.absmean. |
| 637 | ADCm.9.gabor.1.0.2.mag. |
| 638 | ADCm.9.gabor.1.0.3.mean. |
| 639 | ADCm.9.gabor.1.0.3.var. |
| 640 | ADCm.9.gabor.1.0.3.absmean. |
| 641 | ADCm.9.gabor.1.0.3.mag. |
| 642 | ADCm.9.gabor.1.0.4.mean. |
| 643 | ADCm.9.gabor.1.0.4.var. |
| 644 | ADCm.9.gabor.1.0.4.absmean. |
| 645 | ADCm.9.gabor.1.0.4.mag. |
| 646 | ADCm.9.gabor.1.0.5.mean. |
| 647 | ADCm.9.gabor.1.0.5.var. |
| 648 | ADCm.9.gabor.1.0.5.absmean. |
| 649 | ADCm.9.gabor.1.0.5.mag. |
| 650 | ADCm.9.gabor.2.0.1.mean. |
| 651 | ADCm.9.gabor.2.0.1.var. |
| 652 | ADCm.9.gabor.2.0.1.absmean. |
| 653 | ADCm.9.gabor.2.0.1.mag. |
| 654 | ADCm.9.gabor.2.0.2.mean. |
| 655 | ADCm.9.gabor.2.0.2.var. |
| 656 | ADCm.9.gabor.2.0.2.absmean. |
| 657 | ADCm.9.gabor.2.0.2.mag. |
| 658 | ADCm.9.gabor.2.0.3.mean. |
| 659 | ADCm.9.gabor.2.0.3.var. |
| 660 | ADCm.9.gabor.2.0.3.absmean. |
| 661 | ADCm.9.gabor.2.0.3.mag. |
| 662 | ADCm.9.gabor.2.0.4.mean. |
| 663 | ADCm.9.gabor.2.0.4.var. |
| 664 | ADCm.9.gabor.2.0.4.absmean. |
| 665 | ADCm.9.gabor.2.0.4.mag. |
| 666 | ADCm.9.gabor.2.0.5.mean. |
| 667 | ADCm.9.gabor.2.0.5.var. |
| 668 | ADCm.9.gabor.2.0.5.absmean. |
| 669 | ADCm.9.gabor.2.0.5.mag. |
| 670 | ADCm.9.gabor.3.0.1.mean. |
| 671 | ADCm.9.gabor.3.0.1.var. |
| 672 | ADCm.9.gabor.3.0.1.absmean. |
| 673 | ADCm.9.gabor.3.0.1.mag. |
| 674 | ADCm.9.gabor.3.0.2.mean. |
| 675 | ADCm.9.gabor.3.0.2.var. |
| 676 | ADCm.9.gabor.3.0.2.absmean. |
| 677 | ADCm.9.gabor.3.0.2.mag. |
| 678 | ADCm.9.gabor.3.0.3.mean. |
| 679 | ADCm.9.gabor.3.0.3.var. |
| 680 | ADCm.9.gabor.3.0.3.absmean. |
| 681 | ADCm.9.gabor.3.0.3.mag. |
| 682 | ADCm.9.gabor.3.0.4.mean. |
| 683 | ADCm.9.gabor.3.0.4.var. |
| 684 | ADCm.9.gabor.3.0.4.absmean. |
| 685 | ADCm.9.gabor.3.0.4.mag. |
| 686 | ADCm.9.gabor.3.0.5.mean. |
| 687 | ADCm.9.gabor.3.0.5.var. |
| 688 | ADCm.9.gabor.3.0.5.absmean. |
| 689 | ADCm.9.gabor.3.0.5.mag. |
| 690 | ADCm.11.gabor.1.0.1.mean. |
| 691 | ADCm.11.gabor.1.0.1.var. |
| 692 | ADCm.11.gabor.1.0.1.absmean. |
| 693 | ADCm.11.gabor.1.0.1.mag. |
| 694 | ADCm.11.gabor.1.0.2.mean. |
| 695 | ADCm.11.gabor.1.0.2.var. |
| 696 | ADCm.11.gabor.1.0.2.absmean. |
| 697 | ADCm.11.gabor.1.0.2.mag. |
| 698 | ADCm.11.gabor.1.0.3.mean. |
| 699 | ADCm.11.gabor.1.0.3.var. |
| 700 | ADCm.11.gabor.1.0.3.absmean. |
| 701 | ADCm.11.gabor.1.0.3.mag. |
| 702 | ADCm.11.gabor.1.0.4.mean. |
| 703 | ADCm.11.gabor.1.0.4.var. |
| 704 | ADCm.11.gabor.1.0.4.absmean. |
| 705 | ADCm.11.gabor.1.0.4.mag. |
| 706 | ADCm.11.gabor.1.0.5.mean. |
| 707 | ADCm.11.gabor.1.0.5.var. |
| 708 | ADCm.11.gabor.1.0.5.absmean. |
| 709 | ADCm.11.gabor.1.0.5.mag. |
| 710 | ADCm.11.gabor.2.0.1.mean. |
| 711 | ADCm.11.gabor.2.0.1.var. |
| 712 | ADCm.11.gabor.2.0.1.absmean. |
| 713 | ADCm.11.gabor.2.0.1.mag. |
| 714 | ADCm.11.gabor.2.0.2.mean. |
| 715 | ADCm.11.gabor.2.0.2.var. |
| 716 | ADCm.11.gabor.2.0.2.absmean. |
| 717 | ADCm.11.gabor.2.0.2.mag. |
| 718 | ADCm.11.gabor.2.0.3.mean. |
| 719 | ADCm.11.gabor.2.0.3.var. |
| 720 | ADCm.11.gabor.2.0.3.absmean. |
| 721 | ADCm.11.gabor.2.0.3.mag. |
| 722 | ADCm.11.gabor.2.0.4.mean. |
| 723 | ADCm.11.gabor.2.0.4.var. |
| 724 | ADCm.11.gabor.2.0.4.absmean. |
| 725 | ADCm.11.gabor.2.0.4.mag. |
| 726 | ADCm.11.gabor.2.0.5.mean. |
| 727 | ADCm.11.gabor.2.0.5.var. |
| 728 | ADCm.11.gabor.2.0.5.absmean. |
| 729 | ADCm.11.gabor.2.0.5.mag. |
| 730 | ADCm.11.gabor.3.0.1.mean. |
| 731 | ADCm.11.gabor.3.0.1.var. |
| 732 | ADCm.11.gabor.3.0.1.absmean. |
| 733 | ADCm.11.gabor.3.0.1.mag. |
| 734 | ADCm.11.gabor.3.0.2.mean. |
| 735 | ADCm.11.gabor.3.0.2.var. |
| 736 | ADCm.11.gabor.3.0.2.absmean. |
| 737 | ADCm.11.gabor.3.0.2.mag. |
| 738 | ADCm.11.gabor.3.0.3.mean. |
| 739 | ADCm.11.gabor.3.0.3.var. |
| 740 | ADCm.11.gabor.3.0.3.absmean. |
| 741 | ADCm.11.gabor.3.0.3.mag. |
| 742 | ADCm.11.gabor.3.0.4.mean. |
| 743 | ADCm.11.gabor.3.0.4.var. |
| 744 | ADCm.11.gabor.3.0.4.absmean. |
| 745 | ADCm.11.gabor.3.0.4.mag. |
| 746 | ADCm.11.gabor.3.0.5.mean. |
| 747 | ADCm.11.gabor.3.0.5.var. |
| 748 | ADCm.11.gabor.3.0.5.absmean. |
| 749 | ADCm.11.gabor.3.0.5.mag. |
| 750 | ADCm.13.gabor.1.0.1.mean. |
| 751 | ADCm.13.gabor.1.0.1.var. |
| 752 | ADCm.13.gabor.1.0.1.absmean. |
| 753 | ADCm.13.gabor.1.0.1.mag. |
| 754 | ADCm.13.gabor.1.0.2.mean. |
| 755 | ADCm.13.gabor.1.0.2.var. |
| 756 | ADCm.13.gabor.1.0.2.absmean. |
| 757 | ADCm.13.gabor.1.0.2.mag. |
| 758 | ADCm.13.gabor.1.0.3.mean. |
| 759 | ADCm.13.gabor.1.0.3.var. |
| 760 | ADCm.13.gabor.1.0.3.absmean. |
| 761 | ADCm.13.gabor.1.0.3.mag. |
| 762 | ADCm.13.gabor.1.0.4.mean. |
| 763 | ADCm.13.gabor.1.0.4.var. |
| 764 | ADCm.13.gabor.1.0.4.absmean. |
| 765 | ADCm.13.gabor.1.0.4.mag. |
| 766 | ADCm.13.gabor.1.0.5.mean. |
| 767 | ADCm.13.gabor.1.0.5.var. |
| 768 | ADCm.13.gabor.1.0.5.absmean. |
| 769 | ADCm.13.gabor.1.0.5.mag. |
| 770 | ADCm.13.gabor.2.0.1.mean. |
| 771 | ADCm.13.gabor.2.0.1.var. |
| 772 | ADCm.13.gabor.2.0.1.absmean. |
| 773 | ADCm.13.gabor.2.0.1.mag. |
| 774 | ADCm.13.gabor.2.0.2.mean. |
| 775 | ADCm.13.gabor.2.0.2.var. |
| 776 | ADCm.13.gabor.2.0.2.absmean. |
| 777 | ADCm.13.gabor.2.0.2.mag. |
| 778 | ADCm.13.gabor.2.0.3.mean. |
| 779 | ADCm.13.gabor.2.0.3.var. |
| 780 | ADCm.13.gabor.2.0.3.absmean. |
| 781 | ADCm.13.gabor.2.0.3.mag. |
| 782 | ADCm.13.gabor.2.0.4.mean. |
| 783 | ADCm.13.gabor.2.0.4.var. |
| 784 | ADCm.13.gabor.2.0.4.absmean. |
| 785 | ADCm.13.gabor.2.0.4.mag. |
| 786 | ADCm.13.gabor.2.0.5.mean. |
| 787 | ADCm.13.gabor.2.0.5.var. |
| 788 | ADCm.13.gabor.2.0.5.absmean. |
| 789 | ADCm.13.gabor.2.0.5.mag. |
| 790 | ADCm.13.gabor.3.0.1.mean. |
| 791 | ADCm.13.gabor.3.0.1.var. |
| 792 | ADCm.13.gabor.3.0.1.absmean. |
| 793 | ADCm.13.gabor.3.0.1.mag. |
| 794 | ADCm.13.gabor.3.0.2.mean. |
| 795 | ADCm.13.gabor.3.0.2.var. |
| 796 | ADCm.13.gabor.3.0.2.absmean. |
| 797 | ADCm.13.gabor.3.0.2.mag. |
| 798 | ADCm.13.gabor.3.0.3.mean. |
| 799 | ADCm.13.gabor.3.0.3.var. |
| 800 | ADCm.13.gabor.3.0.3.absmean. |
| 801 | ADCm.13.gabor.3.0.3.mag. |
| 802 | ADCm.13.gabor.3.0.4.mean. |
| 803 | ADCm.13.gabor.3.0.4.var. |
| 804 | ADCm.13.gabor.3.0.4.absmean. |
| 805 | ADCm.13.gabor.3.0.4.mag. |
| 806 | ADCm.13.gabor.3.0.5.mean. |
| 807 | ADCm.13.gabor.3.0.5.var. |
| 808 | ADCm.13.gabor.3.0.5.absmean. |
| 809 | ADCm.13.gabor.3.0.5.mag. |
| 810 | ADCm.15.gabor.1.0.1.mean. |
| 811 | ADCm.15.gabor.1.0.1.var. |
| 812 | ADCm.15.gabor.1.0.1.absmean. |
| 813 | ADCm.15.gabor.1.0.1.mag. |
| 814 | ADCm.15.gabor.1.0.2.mean. |
| 815 | ADCm.15.gabor.1.0.2.var. |
| 816 | ADCm.15.gabor.1.0.2.absmean. |
| 817 | ADCm.15.gabor.1.0.2.mag. |
| 818 | ADCm.15.gabor.1.0.3.mean. |
| 819 | ADCm.15.gabor.1.0.3.var. |
| 820 | ADCm.15.gabor.1.0.3.absmean. |
| 821 | ADCm.15.gabor.1.0.3.mag. |
| 822 | ADCm.15.gabor.1.0.4.mean. |
| 823 | ADCm.15.gabor.1.0.4.var. |
| 824 | ADCm.15.gabor.1.0.4.absmean. |
| 825 | ADCm.15.gabor.1.0.4.mag. |
| 826 | ADCm.15.gabor.1.0.5.mean. |
| 827 | ADCm.15.gabor.1.0.5.var. |
| 828 | ADCm.15.gabor.1.0.5.absmean. |
| 829 | ADCm.15.gabor.1.0.5.mag. |
| 830 | ADCm.15.gabor.2.0.1.mean. |
| 831 | ADCm.15.gabor.2.0.1.var. |
| 832 | ADCm.15.gabor.2.0.1.absmean. |
| 833 | ADCm.15.gabor.2.0.1.mag. |
| 834 | ADCm.15.gabor.2.0.2.mean. |
| 835 | ADCm.15.gabor.2.0.2.var. |
| 836 | ADCm.15.gabor.2.0.2.absmean. |
| 837 | ADCm.15.gabor.2.0.2.mag. |
| 838 | ADCm.15.gabor.2.0.3.mean. |
| 839 | ADCm.15.gabor.2.0.3.var. |
| 840 | ADCm.15.gabor.2.0.3.absmean. |
| 841 | ADCm.15.gabor.2.0.3.mag. |
| 842 | ADCm.15.gabor.2.0.4.mean. |
| 843 | ADCm.15.gabor.2.0.4.var. |
| 844 | ADCm.15.gabor.2.0.4.absmean. |
| 845 | ADCm.15.gabor.2.0.4.mag. |
| 846 | ADCm.15.gabor.2.0.5.mean. |
| 847 | ADCm.15.gabor.2.0.5.var. |
| 848 | ADCm.15.gabor.2.0.5.absmean. |
| 849 | ADCm.15.gabor.2.0.5.mag. |
| 850 | ADCm.15.gabor.3.0.1.mean. |
| 851 | ADCm.15.gabor.3.0.1.var. |
| 852 | ADCm.15.gabor.3.0.1.absmean. |
| 853 | ADCm.15.gabor.3.0.1.mag. |
| 854 | ADCm.15.gabor.3.0.2.mean. |
| 855 | ADCm.15.gabor.3.0.2.var. |
| 856 | ADCm.15.gabor.3.0.2.absmean. |
| 857 | ADCm.15.gabor.3.0.2.mag. |
| 858 | ADCm.15.gabor.3.0.3.mean. |
| 859 | ADCm.15.gabor.3.0.3.var. |
| 860 | ADCm.15.gabor.3.0.3.absmean. |
| 861 | ADCm.15.gabor.3.0.3.mag. |
| 862 | ADCm.15.gabor.3.0.4.mean. |
| 863 | ADCm.15.gabor.3.0.4.var. |
| 864 | ADCm.15.gabor.3.0.4.absmean. |
| 865 | ADCm.15.gabor.3.0.4.mag. |
| 866 | ADCm.15.gabor.3.0.5.mean. |
| 867 | ADCm.15.gabor.3.0.5.var. |
| 868 | ADCm.15.gabor.3.0.5.absmean. |
| 869 | ADCm.15.gabor.3.0.5.mag. |
| 870 | ADCm.3.haar.1.1.aav. |
| 871 | ADCm.3.haar.1.1.std. |
| 872 | ADCm.3.haar.1.2.aav. |
| 873 | ADCm.3.haar.1.2.std. |
| 874 | ADCm.3.haar.1.3.aav. |
| 875 | ADCm.3.haar.1.3.std. |
| 876 | ADCm.3.haar.2.1.aav. |
| 877 | ADCm.3.haar.2.1.std. |
| 878 | ADCm.3.haar.2.2.aav. |
| 879 | ADCm.3.haar.2.2.std. |
| 880 | ADCm.3.haar.2.3.aav. |
| 881 | ADCm.3.haar.2.3.std. |
| 882 | ADCm.3.haar.3.1.aav. |
| 883 | ADCm.3.haar.3.1.std. |
| 884 | ADCm.3.haar.3.2.aav. |
| 885 | ADCm.3.haar.3.2.std. |
| 886 | ADCm.3.haar.3.3.aav. |
| 887 | ADCm.3.haar.3.3.std. |
| 888 | ADCm.3.haar.4.1.aav. |
| 889 | ADCm.3.haar.4.1.std. |
| 890 | ADCm.3.haar.4.2.aav. |
| 891 | ADCm.3.haar.4.2.std. |
| 892 | ADCm.3.haar.4.3.aav. |
| 893 | ADCm.3.haar.4.3.std. |
| 894 | ADCm.5.haar.1.1.aav. |
| 895 | ADCm.5.haar.1.1.std. |
| 896 | ADCm.5.haar.1.2.aav. |
| 897 | ADCm.5.haar.1.2.std. |
| 898 | ADCm.5.haar.1.3.aav. |
| 899 | ADCm.5.haar.1.3.std. |
| 900 | ADCm.5.haar.2.1.aav. |
| 901 | ADCm.5.haar.2.1.std. |
| 902 | ADCm.5.haar.2.2.aav. |
| 903 | ADCm.5.haar.2.2.std. |
| 904 | ADCm.5.haar.2.3.aav. |
| 905 | ADCm.5.haar.2.3.std. |
| 906 | ADCm.5.haar.3.1.aav. |
| 907 | ADCm.5.haar.3.1.std. |
| 908 | ADCm.5.haar.3.2.aav. |
| 909 | ADCm.5.haar.3.2.std. |
| 910 | ADCm.5.haar.3.3.aav. |
| 911 | ADCm.5.haar.3.3.std. |
| 912 | ADCm.5.haar.4.1.aav. |
| 913 | ADCm.5.haar.4.1.std. |
| 914 | ADCm.5.haar.4.2.aav. |
| 915 | ADCm.5.haar.4.2.std. |
| 916 | ADCm.5.haar.4.3.aav. |
| 917 | ADCm.5.haar.4.3.std. |
| 918 | ADCm.7.haar.1.1.aav. |
| 919 | ADCm.7.haar.1.1.std. |
| 920 | ADCm.7.haar.1.2.aav. |
| 921 | ADCm.7.haar.1.2.std. |
| 922 | ADCm.7.haar.1.3.aav. |
| 923 | ADCm.7.haar.1.3.std. |
| 924 | ADCm.7.haar.2.1.aav. |
| 925 | ADCm.7.haar.2.1.std. |
| 926 | ADCm.7.haar.2.2.aav. |
| 927 | ADCm.7.haar.2.2.std. |
| 928 | ADCm.7.haar.2.3.aav. |
| 929 | ADCm.7.haar.2.3.std. |
| 930 | ADCm.7.haar.3.1.aav. |
| 931 | ADCm.7.haar.3.1.std. |
| 932 | ADCm.7.haar.3.2.aav. |
| 933 | ADCm.7.haar.3.2.std. |
| 934 | ADCm.7.haar.3.3.aav. |
| 935 | ADCm.7.haar.3.3.std. |
| 936 | ADCm.7.haar.4.1.aav. |
| 937 | ADCm.7.haar.4.1.std. |
| 938 | ADCm.7.haar.4.2.aav. |
| 939 | ADCm.7.haar.4.2.std. |
| 940 | ADCm.7.haar.4.3.aav. |
| 941 | ADCm.7.haar.4.3.std. |
| 942 | ADCm.9.haar.1.1.aav. |
| 943 | ADCm.9.haar.1.1.std. |
| 944 | ADCm.9.haar.1.2.aav. |
| 945 | ADCm.9.haar.1.2.std. |
| 946 | ADCm.9.haar.1.3.aav. |
| 947 | ADCm.9.haar.1.3.std. |
| 948 | ADCm.9.haar.2.1.aav. |
| 949 | ADCm.9.haar.2.1.std. |
| 950 | ADCm.9.haar.2.2.aav. |
| 951 | ADCm.9.haar.2.2.std. |
| 952 | ADCm.9.haar.2.3.aav. |
| 953 | ADCm.9.haar.2.3.std. |
| 954 | ADCm.9.haar.3.1.aav. |
| 955 | ADCm.9.haar.3.1.std. |
| 956 | ADCm.9.haar.3.2.aav. |
| 957 | ADCm.9.haar.3.2.std. |
| 958 | ADCm.9.haar.3.3.aav. |
| 959 | ADCm.9.haar.3.3.std. |
| 960 | ADCm.9.haar.4.1.aav. |
| 961 | ADCm.9.haar.4.1.std. |
| 962 | ADCm.9.haar.4.2.aav. |
| 963 | ADCm.9.haar.4.2.std. |
| 964 | ADCm.9.haar.4.3.aav. |
| 965 | ADCm.9.haar.4.3.std. |
| 966 | ADCm.11.haar.1.1.aav. |
| 967 | ADCm.11.haar.1.1.std. |
| 968 | ADCm.11.haar.1.2.aav. |
| 969 | ADCm.11.haar.1.2.std. |
| 970 | ADCm.11.haar.1.3.aav. |
| 971 | ADCm.11.haar.1.3.std. |
| 972 | ADCm.11.haar.2.1.aav. |
| 973 | ADCm.11.haar.2.1.std. |
| 974 | ADCm.11.haar.2.2.aav. |
| 975 | ADCm.11.haar.2.2.std. |
| 976 | ADCm.11.haar.2.3.aav. |
| 977 | ADCm.11.haar.2.3.std. |
| 978 | ADCm.11.haar.3.1.aav. |
| 979 | ADCm.11.haar.3.1.std. |
| 980 | ADCm.11.haar.3.2.aav. |
| 981 | ADCm.11.haar.3.2.std. |
| 982 | ADCm.11.haar.3.3.aav. |
| 983 | ADCm.11.haar.3.3.std. |
| 984 | ADCm.11.haar.4.1.aav. |
| 985 | ADCm.11.haar.4.1.std. |
| 986 | ADCm.11.haar.4.2.aav. |
| 987 | ADCm.11.haar.4.2.std. |
| 988 | ADCm.11.haar.4.3.aav. |
| 989 | ADCm.11.haar.4.3.std. |
| 990 | ADCm.13.haar.1.1.aav. |
| 991 | ADCm.13.haar.1.1.std. |
| 992 | ADCm.13.haar.1.2.aav. |
| 993 | ADCm.13.haar.1.2.std. |
| 994 | ADCm.13.haar.1.3.aav. |
| 995 | ADCm.13.haar.1.3.std. |
| 996 | ADCm.13.haar.2.1.aav. |
| 997 | ADCm.13.haar.2.1.std. |
| 998 | ADCm.13.haar.2.2.aav. |
| 999 | ADCm.13.haar.2.2.std. |
| 1000 | ADCm.13.haar.2.3.aav. |
| 1001 | ADCm.13.haar.2.3.std. |
| 1002 | ADCm.13.haar.3.1.aav. |
| 1003 | ADCm.13.haar.3.1.std. |
| 1004 | ADCm.13.haar.3.2.aav. |
| 1005 | ADCm.13.haar.3.2.std. |
| 1006 | ADCm.13.haar.3.3.aav. |
| 1007 | ADCm.13.haar.3.3.std. |
| 1008 | ADCm.13.haar.4.1.aav. |
| 1009 | ADCm.13.haar.4.1.std. |
| 1010 | ADCm.13.haar.4.2.aav. |
| 1011 | ADCm.13.haar.4.2.std. |
| 1012 | ADCm.13.haar.4.3.aav. |
| 1013 | ADCm.13.haar.4.3.std. |
| 1014 | ADCm.15.haar.1.1.aav. |
| 1015 | ADCm.15.haar.1.1.std. |
| 1016 | ADCm.15.haar.1.2.aav. |
| 1017 | ADCm.15.haar.1.2.std. |
| 1018 | ADCm.15.haar.1.3.aav. |
| 1019 | ADCm.15.haar.1.3.std. |
| 1020 | ADCm.15.haar.2.1.aav. |
| 1021 | ADCm.15.haar.2.1.std. |
| 1022 | ADCm.15.haar.2.2.aav. |
| 1023 | ADCm.15.haar.2.2.std. |
| 1024 | ADCm.15.haar.2.3.aav. |
| 1025 | ADCm.15.haar.2.3.std. |
| 1026 | ADCm.15.haar.3.1.aav. |
| 1027 | ADCm.15.haar.3.1.std. |
| 1028 | ADCm.15.haar.3.2.aav. |
| 1029 | ADCm.15.haar.3.2.std. |
| 1030 | ADCm.15.haar.3.3.aav. |
| 1031 | ADCm.15.haar.3.3.std. |
| 1032 | ADCm.15.haar.4.1.aav. |
| 1033 | ADCm.15.haar.4.1.std. |
| 1034 | ADCm.15.haar.4.2.aav. |
| 1035 | ADCm.15.haar.4.2.std. |
| 1036 | ADCm.15.haar.4.3.aav. |
| 1037 | ADCm.15.haar.4.3.std. |
| 1038 | ADCm.3.hu.0. |
| 1039 | ADCm.3.hu.1. |
| 1040 | ADCm.3.hu.2. |
| 1041 | ADCm.3.hu.3. |
| 1042 | ADCm.3.hu.4. |
| 1043 | ADCm.3.hu.5. |
| 1044 | ADCm.3.hu.6. |
| 1045 | ADCm.5.hu.0. |
| 1046 | ADCm.5.hu.1. |
| 1047 | ADCm.5.hu.2. |
| 1048 | ADCm.5.hu.3. |
| 1049 | ADCm.5.hu.4. |
| 1050 | ADCm.5.hu.5. |
| 1051 | ADCm.5.hu.6. |
| 1052 | ADCm.7.hu.0. |
| 1053 | ADCm.7.hu.1. |
| 1054 | ADCm.7.hu.2. |
| 1055 | ADCm.7.hu.3. |
| 1056 | ADCm.7.hu.4. |
| 1057 | ADCm.7.hu.5. |
| 1058 | ADCm.7.hu.6. |
| 1059 | ADCm.9.hu.0. |
| 1060 | ADCm.9.hu.1. |
| 1061 | ADCm.9.hu.2. |
| 1062 | ADCm.9.hu.3. |
| 1063 | ADCm.9.hu.4. |
| 1064 | ADCm.9.hu.5. |
| 1065 | ADCm.9.hu.6. |
| 1066 | ADCm.11.hu.0. |
| 1067 | ADCm.11.hu.1. |
| 1068 | ADCm.11.hu.2. |
| 1069 | ADCm.11.hu.3. |
| 1070 | ADCm.11.hu.4. |
| 1071 | ADCm.11.hu.5. |
| 1072 | ADCm.11.hu.6. |
| 1073 | ADCm.13.hu.0. |
| 1074 | ADCm.13.hu.1. |
| 1075 | ADCm.13.hu.2. |
| 1076 | ADCm.13.hu.3. |
| 1077 | ADCm.13.hu.4. |
| 1078 | ADCm.13.hu.5. |
| 1079 | ADCm.13.hu.6. |
| 1080 | ADCm.15.hu.0. |
| 1081 | ADCm.15.hu.1. |
| 1082 | ADCm.15.hu.2. |
| 1083 | ADCm.15.hu.3. |
| 1084 | ADCm.15.hu.4. |
| 1085 | ADCm.15.hu.5. |
| 1086 | ADCm.15.hu.6. |
| 1087 | ADCm.3.zernike.0. |
| 1088 | ADCm.3.zernike.1. |
| 1089 | ADCm.3.zernike.2. |
| 1090 | ADCm.3.zernike.3. |
| 1091 | ADCm.3.zernike.4. |
| 1092 | ADCm.3.zernike.5. |
| 1093 | ADCm.3.zernike.6. |
| 1094 | ADCm.3.zernike.7. |
| 1095 | ADCm.3.zernike.8. |
| 1096 | ADCm.3.zernike.9. |
| 1097 | ADCm.3.zernike.10. |
| 1098 | ADCm.3.zernike.11. |
| 1099 | ADCm.3.zernike.12. |
| 1100 | ADCm.3.zernike.13. |
| 1101 | ADCm.3.zernike.14. |
| 1102 | ADCm.3.zernike.15. |
| 1103 | ADCm.3.zernike.16. |
| 1104 | ADCm.3.zernike.17. |
| 1105 | ADCm.3.zernike.18. |
| 1106 | ADCm.3.zernike.19. |
| 1107 | ADCm.3.zernike.20. |
| 1108 | ADCm.3.zernike.21. |
| 1109 | ADCm.3.zernike.22. |
| 1110 | ADCm.3.zernike.23. |
| 1111 | ADCm.3.zernike.24. |
| 1112 | ADCm.5.zernike.0. |
| 1113 | ADCm.5.zernike.1. |
| 1114 | ADCm.5.zernike.2. |
| 1115 | ADCm.5.zernike.3. |
| 1116 | ADCm.5.zernike.4. |
| 1117 | ADCm.5.zernike.5. |
| 1118 | ADCm.5.zernike.6. |
| 1119 | ADCm.5.zernike.7. |
| 1120 | ADCm.5.zernike.8. |
| 1121 | ADCm.5.zernike.9. |
| 1122 | ADCm.5.zernike.10. |
| 1123 | ADCm.5.zernike.11. |
| 1124 | ADCm.5.zernike.12. |
| 1125 | ADCm.5.zernike.13. |
| 1126 | ADCm.5.zernike.14. |
| 1127 | ADCm.5.zernike.15. |
| 1128 | ADCm.5.zernike.16. |
| 1129 | ADCm.5.zernike.17. |
| 1130 | ADCm.5.zernike.18. |
| 1131 | ADCm.5.zernike.19. |
| 1132 | ADCm.5.zernike.20. |
| 1133 | ADCm.5.zernike.21. |
| 1134 | ADCm.5.zernike.22. |
| 1135 | ADCm.5.zernike.23. |
| 1136 | ADCm.5.zernike.24. |
| 1137 | ADCm.7.zernike.0. |
| 1138 | ADCm.7.zernike.1. |
| 1139 | ADCm.7.zernike.2. |
| 1140 | ADCm.7.zernike.3. |
| 1141 | ADCm.7.zernike.4. |
| 1142 | ADCm.7.zernike.5. |
| 1143 | ADCm.7.zernike.6. |
| 1144 | ADCm.7.zernike.7. |
| 1145 | ADCm.7.zernike.8. |
| 1146 | ADCm.7.zernike.9. |
| 1147 | ADCm.7.zernike.10. |
| 1148 | ADCm.7.zernike.11. |
| 1149 | ADCm.7.zernike.12. |
| 1150 | ADCm.7.zernike.13. |
| 1151 | ADCm.7.zernike.14. |
| 1152 | ADCm.7.zernike.15. |
| 1153 | ADCm.7.zernike.16. |
| 1154 | ADCm.7.zernike.17. |
| 1155 | ADCm.7.zernike.18. |
| 1156 | ADCm.7.zernike.19. |
| 1157 | ADCm.7.zernike.20. |
| 1158 | ADCm.7.zernike.21. |
| 1159 | ADCm.7.zernike.22. |
| 1160 | ADCm.7.zernike.23. |
| 1161 | ADCm.7.zernike.24. |
| 1162 | ADCm.9.zernike.0. |
| 1163 | ADCm.9.zernike.1. |
| 1164 | ADCm.9.zernike.2. |
| 1165 | ADCm.9.zernike.3. |
| 1166 | ADCm.9.zernike.4. |
| 1167 | ADCm.9.zernike.5. |
| 1168 | ADCm.9.zernike.6. |
| 1169 | ADCm.9.zernike.7. |
| 1170 | ADCm.9.zernike.8. |
| 1171 | ADCm.9.zernike.9. |
| 1172 | ADCm.9.zernike.10. |
| 1173 | ADCm.9.zernike.11. |
| 1174 | ADCm.9.zernike.12. |
| 1175 | ADCm.9.zernike.13. |
| 1176 | ADCm.9.zernike.14. |
| 1177 | ADCm.9.zernike.15. |
| 1178 | ADCm.9.zernike.16. |
| 1179 | ADCm.9.zernike.17. |
| 1180 | ADCm.9.zernike.18. |
| 1181 | ADCm.9.zernike.19. |
| 1182 | ADCm.9.zernike.20. |
| 1183 | ADCm.9.zernike.21. |
| 1184 | ADCm.9.zernike.22. |
| 1185 | ADCm.9.zernike.23. |
| 1186 | ADCm.9.zernike.24. |
| 1187 | ADCm.11.zernike.0. |
| 1188 | ADCm.11.zernike.1. |
| 1189 | ADCm.11.zernike.2. |
| 1190 | ADCm.11.zernike.3. |
| 1191 | ADCm.11.zernike.4. |
| 1192 | ADCm.11.zernike.5. |
| 1193 | ADCm.11.zernike.6. |
| 1194 | ADCm.11.zernike.7. |
| 1195 | ADCm.11.zernike.8. |
| 1196 | ADCm.11.zernike.9. |
| 1197 | ADCm.11.zernike.10. |
| 1198 | ADCm.11.zernike.11. |
| 1199 | ADCm.11.zernike.12. |
| 1200 | ADCm.11.zernike.13. |
| 1201 | ADCm.11.zernike.14. |
| 1202 | ADCm.11.zernike.15. |
| 1203 | ADCm.11.zernike.16. |
| 1204 | ADCm.11.zernike.17. |
| 1205 | ADCm.11.zernike.18. |
| 1206 | ADCm.11.zernike.19. |
| 1207 | ADCm.11.zernike.20. |
| 1208 | ADCm.11.zernike.21. |
| 1209 | ADCm.11.zernike.22. |
| 1210 | ADCm.11.zernike.23. |
| 1211 | ADCm.11.zernike.24. |
| 1212 | ADCm.13.zernike.0. |
| 1213 | ADCm.13.zernike.1. |
| 1214 | ADCm.13.zernike.2. |
| 1215 | ADCm.13.zernike.3. |
| 1216 | ADCm.13.zernike.4. |
| 1217 | ADCm.13.zernike.5. |
| 1218 | ADCm.13.zernike.6. |
| 1219 | ADCm.13.zernike.7. |
| 1220 | ADCm.13.zernike.8. |
| 1221 | ADCm.13.zernike.9. |
| 1222 | ADCm.13.zernike.10. |
| 1223 | ADCm.13.zernike.11. |
| 1224 | ADCm.13.zernike.12. |
| 1225 | ADCm.13.zernike.13. |
| 1226 | ADCm.13.zernike.14. |
| 1227 | ADCm.13.zernike.15. |
| 1228 | ADCm.13.zernike.16. |
| 1229 | ADCm.13.zernike.17. |
| 1230 | ADCm.13.zernike.18. |
| 1231 | ADCm.13.zernike.19. |
| 1232 | ADCm.13.zernike.20. |
| 1233 | ADCm.13.zernike.21. |
| 1234 | ADCm.13.zernike.22. |
| 1235 | ADCm.13.zernike.23. |
| 1236 | ADCm.13.zernike.24. |
| 1237 | ADCm.15.zernike.0. |
| 1238 | ADCm.15.zernike.1. |
| 1239 | ADCm.15.zernike.2. |
| 1240 | ADCm.15.zernike.3. |
| 1241 | ADCm.15.zernike.4. |
| 1242 | ADCm.15.zernike.5. |
| 1243 | ADCm.15.zernike.6. |
| 1244 | ADCm.15.zernike.7. |
| 1245 | ADCm.15.zernike.8. |
| 1246 | ADCm.15.zernike.9. |
| 1247 | ADCm.15.zernike.10. |
| 1248 | ADCm.15.zernike.11. |
| 1249 | ADCm.15.zernike.12. |
| 1250 | ADCm.15.zernike.13. |
| 1251 | ADCm.15.zernike.14. |
| 1252 | ADCm.15.zernike.15. |
| 1253 | ADCm.15.zernike.16. |
| 1254 | ADCm.15.zernike.17. |
| 1255 | ADCm.15.zernike.18. |
| 1256 | ADCm.15.zernike.19. |
| 1257 | ADCm.15.zernike.20. |
| 1258 | ADCm.15.zernike.21. |
| 1259 | ADCm.15.zernike.22. |
| 1260 | ADCm.15.zernike.23. |
| 1261 | ADCm.15.zernike.24. |
| 1262 | ADCm.3.sobel |
| 1263 | ADCm.3.sobel_mask |
| 1264 | ADCm.all.stats.p000. |
| 1265 | ADCm.all.stats.p010. |
| 1266 | ADCm.all.stats.p020. |
| 1267 | ADCm.all.stats.p025. |
| 1268 | ADCm.all.stats.p030. |
| 1269 | ADCm.all.stats.p040. |
| 1270 | ADCm.all.stats.p050. |
| 1271 | ADCm.all.stats.p060. |
| 1272 | ADCm.all.stats.p070. |
| 1273 | ADCm.all.stats.p075. |
| 1274 | ADCm.all.stats.p080. |
| 1275 | ADCm.all.stats.p090. |
| 1276 | ADCm.all.stats.p100. |
| 1277 | ADCm.all.stats.range. |
| 1278 | ADCm.all.stats.mean. |
| 1279 | ADCm.all.stats.stddev. |
| 1280 | ADCm.all.stats.kurtosis. |
| 1281 | ADCm.all.stats.skewness. |
| 1282 | ADCk.3.glcm.contrast.1.mean. |
| 1283 | ADCk.3.glcm.contrast.1.range. |
| 1284 | ADCk.3.glcm.contrast.2.mean. |
| 1285 | ADCk.3.glcm.contrast.2.range. |
| 1286 | ADCk.3.glcm.contrast.3.mean. |
| 1287 | ADCk.3.glcm.contrast.3.range. |
| 1288 | ADCk.3.glcm.dissimilarity.1.mean. |
| 1289 | ADCk.3.glcm.dissimilarity.1.range. |
| 1290 | ADCk.3.glcm.dissimilarity.2.mean. |
| 1291 | ADCk.3.glcm.dissimilarity.2.range. |
| 1292 | ADCk.3.glcm.dissimilarity.3.mean. |
| 1293 | ADCk.3.glcm.dissimilarity.3.range. |
| 1294 | ADCk.3.glcm.homogeneity.1.mean. |
| 1295 | ADCk.3.glcm.homogeneity.1.range. |
| 1296 | ADCk.3.glcm.homogeneity.2.mean. |
| 1297 | ADCk.3.glcm.homogeneity.2.range. |
| 1298 | ADCk.3.glcm.homogeneity.3.mean. |
| 1299 | ADCk.3.glcm.homogeneity.3.range. |
| 1300 | ADCk.3.glcm.energy.1.mean. |
| 1301 | ADCk.3.glcm.energy.1.range. |
| 1302 | ADCk.3.glcm.energy.2.mean. |
| 1303 | ADCk.3.glcm.energy.2.range. |
| 1304 | ADCk.3.glcm.energy.3.mean. |
| 1305 | ADCk.3.glcm.energy.3.range. |
| 1306 | ADCk.3.glcm.correlation.1.mean. |
| 1307 | ADCk.3.glcm.correlation.1.range. |
| 1308 | ADCk.3.glcm.correlation.2.mean. |
| 1309 | ADCk.3.glcm.correlation.2.range. |
| 1310 | ADCk.3.glcm.correlation.3.mean. |
| 1311 | ADCk.3.glcm.correlation.3.range. |
| 1312 | ADCk.3.glcm.ASM.1.mean. |
| 1313 | ADCk.3.glcm.ASM.1.range. |
| 1314 | ADCk.3.glcm.ASM.2.mean. |
| 1315 | ADCk.3.glcm.ASM.2.range. |
| 1316 | ADCk.3.glcm.ASM.3.mean. |
| 1317 | ADCk.3.glcm.ASM.3.range. |
| 1318 | ADCk.5.glcm.contrast.1.mean. |
| 1319 | ADCk.5.glcm.contrast.1.range. |
| 1320 | ADCk.5.glcm.contrast.2.mean. |
| 1321 | ADCk.5.glcm.contrast.2.range. |
| 1322 | ADCk.5.glcm.contrast.3.mean. |
| 1323 | ADCk.5.glcm.contrast.3.range. |
| 1324 | ADCk.5.glcm.contrast.4.mean. |
| 1325 | ADCk.5.glcm.contrast.4.range. |
| 1326 | ADCk.5.glcm.dissimilarity.1.mean. |
| 1327 | ADCk.5.glcm.dissimilarity.1.range. |
| 1328 | ADCk.5.glcm.dissimilarity.2.mean. |
| 1329 | ADCk.5.glcm.dissimilarity.2.range. |
| 1330 | ADCk.5.glcm.dissimilarity.3.mean. |
| 1331 | ADCk.5.glcm.dissimilarity.3.range. |
| 1332 | ADCk.5.glcm.dissimilarity.4.mean. |
| 1333 | ADCk.5.glcm.dissimilarity.4.range. |
| 1334 | ADCk.5.glcm.homogeneity.1.mean. |
| 1335 | ADCk.5.glcm.homogeneity.1.range. |
| 1336 | ADCk.5.glcm.homogeneity.2.mean. |
| 1337 | ADCk.5.glcm.homogeneity.2.range. |
| 1338 | ADCk.5.glcm.homogeneity.3.mean. |
| 1339 | ADCk.5.glcm.homogeneity.3.range. |
| 1340 | ADCk.5.glcm.homogeneity.4.mean. |
| 1341 | ADCk.5.glcm.homogeneity.4.range. |
| 1342 | ADCk.5.glcm.energy.1.mean. |
| 1343 | ADCk.5.glcm.energy.1.range. |
| 1344 | ADCk.5.glcm.energy.2.mean. |
| 1345 | ADCk.5.glcm.energy.2.range. |
| 1346 | ADCk.5.glcm.energy.3.mean. |
| 1347 | ADCk.5.glcm.energy.3.range. |
| 1348 | ADCk.5.glcm.energy.4.mean. |
| 1349 | ADCk.5.glcm.energy.4.range. |
| 1350 | ADCk.5.glcm.correlation.1.mean. |
| 1351 | ADCk.5.glcm.correlation.1.range. |
| 1352 | ADCk.5.glcm.correlation.2.mean. |
| 1353 | ADCk.5.glcm.correlation.2.range. |
| 1354 | ADCk.5.glcm.correlation.3.mean. |
| 1355 | ADCk.5.glcm.correlation.3.range. |
| 1356 | ADCk.5.glcm.correlation.4.mean. |
| 1357 | ADCk.5.glcm.correlation.4.range. |
| 1358 | ADCk.5.glcm.ASM.1.mean. |
| 1359 | ADCk.5.glcm.ASM.1.range. |
| 1360 | ADCk.5.glcm.ASM.2.mean. |
| 1361 | ADCk.5.glcm.ASM.2.range. |
| 1362 | ADCk.5.glcm.ASM.3.mean. |
| 1363 | ADCk.5.glcm.ASM.3.range. |
| 1364 | ADCk.5.glcm.ASM.4.mean. |
| 1365 | ADCk.5.glcm.ASM.4.range. |
| 1366 | ADCk.7.glcm.contrast.1.mean. |
| 1367 | ADCk.7.glcm.contrast.1.range. |
| 1368 | ADCk.7.glcm.contrast.2.mean. |
| 1369 | ADCk.7.glcm.contrast.2.range. |
| 1370 | ADCk.7.glcm.contrast.3.mean. |
| 1371 | ADCk.7.glcm.contrast.3.range. |
| 1372 | ADCk.7.glcm.contrast.4.mean. |
| 1373 | ADCk.7.glcm.contrast.4.range. |
| 1374 | ADCk.7.glcm.dissimilarity.1.mean. |
| 1375 | ADCk.7.glcm.dissimilarity.1.range. |
| 1376 | ADCk.7.glcm.dissimilarity.2.mean. |
| 1377 | ADCk.7.glcm.dissimilarity.2.range. |
| 1378 | ADCk.7.glcm.dissimilarity.3.mean. |
| 1379 | ADCk.7.glcm.dissimilarity.3.range. |
| 1380 | ADCk.7.glcm.dissimilarity.4.mean. |
| 1381 | ADCk.7.glcm.dissimilarity.4.range. |
| 1382 | ADCk.7.glcm.homogeneity.1.mean. |
| 1383 | ADCk.7.glcm.homogeneity.1.range. |
| 1384 | ADCk.7.glcm.homogeneity.2.mean. |
| 1385 | ADCk.7.glcm.homogeneity.2.range. |
| 1386 | ADCk.7.glcm.homogeneity.3.mean. |
| 1387 | ADCk.7.glcm.homogeneity.3.range. |
| 1388 | ADCk.7.glcm.homogeneity.4.mean. |
| 1389 | ADCk.7.glcm.homogeneity.4.range. |
| 1390 | ADCk.7.glcm.energy.1.mean. |
| 1391 | ADCk.7.glcm.energy.1.range. |
| 1392 | ADCk.7.glcm.energy.2.mean. |
| 1393 | ADCk.7.glcm.energy.2.range. |
| 1394 | ADCk.7.glcm.energy.3.mean. |
| 1395 | ADCk.7.glcm.energy.3.range. |
| 1396 | ADCk.7.glcm.energy.4.mean. |
| 1397 | ADCk.7.glcm.energy.4.range. |
| 1398 | ADCk.7.glcm.correlation.1.mean. |
| 1399 | ADCk.7.glcm.correlation.1.range. |
| 1400 | ADCk.7.glcm.correlation.2.mean. |
| 1401 | ADCk.7.glcm.correlation.2.range. |
| 1402 | ADCk.7.glcm.correlation.3.mean. |
| 1403 | ADCk.7.glcm.correlation.3.range. |
| 1404 | ADCk.7.glcm.correlation.4.mean. |
| 1405 | ADCk.7.glcm.correlation.4.range. |
| 1406 | ADCk.7.glcm.ASM.1.mean. |
| 1407 | ADCk.7.glcm.ASM.1.range. |
| 1408 | ADCk.7.glcm.ASM.2.mean. |
| 1409 | ADCk.7.glcm.ASM.2.range. |
| 1410 | ADCk.7.glcm.ASM.3.mean. |
| 1411 | ADCk.7.glcm.ASM.3.range. |
| 1412 | ADCk.7.glcm.ASM.4.mean. |
| 1413 | ADCk.7.glcm.ASM.4.range. |
| 1414 | ADCk.9.glcm.contrast.1.mean. |
| 1415 | ADCk.9.glcm.contrast.1.range. |
| 1416 | ADCk.9.glcm.contrast.2.mean. |
| 1417 | ADCk.9.glcm.contrast.2.range. |
| 1418 | ADCk.9.glcm.contrast.3.mean. |
| 1419 | ADCk.9.glcm.contrast.3.range. |
| 1420 | ADCk.9.glcm.contrast.4.mean. |
| 1421 | ADCk.9.glcm.contrast.4.range. |
| 1422 | ADCk.9.glcm.dissimilarity.1.mean. |
| 1423 | ADCk.9.glcm.dissimilarity.1.range. |
| 1424 | ADCk.9.glcm.dissimilarity.2.mean. |
| 1425 | ADCk.9.glcm.dissimilarity.2.range. |
| 1426 | ADCk.9.glcm.dissimilarity.3.mean. |
| 1427 | ADCk.9.glcm.dissimilarity.3.range. |
| 1428 | ADCk.9.glcm.dissimilarity.4.mean. |
| 1429 | ADCk.9.glcm.dissimilarity.4.range. |
| 1430 | ADCk.9.glcm.homogeneity.1.mean. |
| 1431 | ADCk.9.glcm.homogeneity.1.range. |
| 1432 | ADCk.9.glcm.homogeneity.2.mean. |
| 1433 | ADCk.9.glcm.homogeneity.2.range. |
| 1434 | ADCk.9.glcm.homogeneity.3.mean. |
| 1435 | ADCk.9.glcm.homogeneity.3.range. |
| 1436 | ADCk.9.glcm.homogeneity.4.mean. |
| 1437 | ADCk.9.glcm.homogeneity.4.range. |
| 1438 | ADCk.9.glcm.energy.1.mean. |
| 1439 | ADCk.9.glcm.energy.1.range. |
| 1440 | ADCk.9.glcm.energy.2.mean. |
| 1441 | ADCk.9.glcm.energy.2.range. |
| 1442 | ADCk.9.glcm.energy.3.mean. |
| 1443 | ADCk.9.glcm.energy.3.range. |
| 1444 | ADCk.9.glcm.energy.4.mean. |
| 1445 | ADCk.9.glcm.energy.4.range. |
| 1446 | ADCk.9.glcm.correlation.1.mean. |
| 1447 | ADCk.9.glcm.correlation.1.range. |
| 1448 | ADCk.9.glcm.correlation.2.mean. |
| 1449 | ADCk.9.glcm.correlation.2.range. |
| 1450 | ADCk.9.glcm.correlation.3.mean. |
| 1451 | ADCk.9.glcm.correlation.3.range. |
| 1452 | ADCk.9.glcm.correlation.4.mean. |
| 1453 | ADCk.9.glcm.correlation.4.range. |
| 1454 | ADCk.9.glcm.ASM.1.mean. |
| 1455 | ADCk.9.glcm.ASM.1.range. |
| 1456 | ADCk.9.glcm.ASM.2.mean. |
| 1457 | ADCk.9.glcm.ASM.2.range. |
| 1458 | ADCk.9.glcm.ASM.3.mean. |
| 1459 | ADCk.9.glcm.ASM.3.range. |
| 1460 | ADCk.9.glcm.ASM.4.mean. |
| 1461 | ADCk.9.glcm.ASM.4.range. |
| 1462 | ADCk.11.glcm.contrast.1.mean. |
| 1463 | ADCk.11.glcm.contrast.1.range. |
| 1464 | ADCk.11.glcm.contrast.2.mean. |
| 1465 | ADCk.11.glcm.contrast.2.range. |
| 1466 | ADCk.11.glcm.contrast.3.mean. |
| 1467 | ADCk.11.glcm.contrast.3.range. |
| 1468 | ADCk.11.glcm.contrast.4.mean. |
| 1469 | ADCk.11.glcm.contrast.4.range. |
| 1470 | ADCk.11.glcm.dissimilarity.1.mean. |
| 1471 | ADCk.11.glcm.dissimilarity.1.range. |
| 1472 | ADCk.11.glcm.dissimilarity.2.mean. |
| 1473 | ADCk.11.glcm.dissimilarity.2.range. |
| 1474 | ADCk.11.glcm.dissimilarity.3.mean. |
| 1475 | ADCk.11.glcm.dissimilarity.3.range. |
| 1476 | ADCk.11.glcm.dissimilarity.4.mean. |
| 1477 | ADCk.11.glcm.dissimilarity.4.range. |
| 1478 | ADCk.11.glcm.homogeneity.1.mean. |
| 1479 | ADCk.11.glcm.homogeneity.1.range. |
| 1480 | ADCk.11.glcm.homogeneity.2.mean. |
| 1481 | ADCk.11.glcm.homogeneity.2.range. |
| 1482 | ADCk.11.glcm.homogeneity.3.mean. |
| 1483 | ADCk.11.glcm.homogeneity.3.range. |
| 1484 | ADCk.11.glcm.homogeneity.4.mean. |
| 1485 | ADCk.11.glcm.homogeneity.4.range. |
| 1486 | ADCk.11.glcm.energy.1.mean. |
| 1487 | ADCk.11.glcm.energy.1.range. |
| 1488 | ADCk.11.glcm.energy.2.mean. |
| 1489 | ADCk.11.glcm.energy.2.range. |
| 1490 | ADCk.11.glcm.energy.3.mean. |
| 1491 | ADCk.11.glcm.energy.3.range. |
| 1492 | ADCk.11.glcm.energy.4.mean. |
| 1493 | ADCk.11.glcm.energy.4.range. |
| 1494 | ADCk.11.glcm.correlation.1.mean. |
| 1495 | ADCk.11.glcm.correlation.1.range. |
| 1496 | ADCk.11.glcm.correlation.2.mean. |
| 1497 | ADCk.11.glcm.correlation.2.range. |
| 1498 | ADCk.11.glcm.correlation.3.mean. |
| 1499 | ADCk.11.glcm.correlation.3.range. |
| 1500 | ADCk.11.glcm.correlation.4.mean. |
| 1501 | ADCk.11.glcm.correlation.4.range. |
| 1502 | ADCk.11.glcm.ASM.1.mean. |
| 1503 | ADCk.11.glcm.ASM.1.range. |
| 1504 | ADCk.11.glcm.ASM.2.mean. |
| 1505 | ADCk.11.glcm.ASM.2.range. |
| 1506 | ADCk.11.glcm.ASM.3.mean. |
| 1507 | ADCk.11.glcm.ASM.3.range. |
| 1508 | ADCk.11.glcm.ASM.4.mean. |
| 1509 | ADCk.11.glcm.ASM.4.range. |
| 1510 | ADCk.13.glcm.contrast.1.mean. |
| 1511 | ADCk.13.glcm.contrast.1.range. |
| 1512 | ADCk.13.glcm.contrast.2.mean. |
| 1513 | ADCk.13.glcm.contrast.2.range. |
| 1514 | ADCk.13.glcm.contrast.3.mean. |
| 1515 | ADCk.13.glcm.contrast.3.range. |
| 1516 | ADCk.13.glcm.contrast.4.mean. |
| 1517 | ADCk.13.glcm.contrast.4.range. |
| 1518 | ADCk.13.glcm.dissimilarity.1.mean. |
| 1519 | ADCk.13.glcm.dissimilarity.1.range. |
| 1520 | ADCk.13.glcm.dissimilarity.2.mean. |
| 1521 | ADCk.13.glcm.dissimilarity.2.range. |
| 1522 | ADCk.13.glcm.dissimilarity.3.mean. |
| 1523 | ADCk.13.glcm.dissimilarity.3.range. |
| 1524 | ADCk.13.glcm.dissimilarity.4.mean. |
| 1525 | ADCk.13.glcm.dissimilarity.4.range. |
| 1526 | ADCk.13.glcm.homogeneity.1.mean. |
| 1527 | ADCk.13.glcm.homogeneity.1.range. |
| 1528 | ADCk.13.glcm.homogeneity.2.mean. |
| 1529 | ADCk.13.glcm.homogeneity.2.range. |
| 1530 | ADCk.13.glcm.homogeneity.3.mean. |
| 1531 | ADCk.13.glcm.homogeneity.3.range. |
| 1532 | ADCk.13.glcm.homogeneity.4.mean. |
| 1533 | ADCk.13.glcm.homogeneity.4.range. |
| 1534 | ADCk.13.glcm.energy.1.mean. |
| 1535 | ADCk.13.glcm.energy.1.range. |
| 1536 | ADCk.13.glcm.energy.2.mean. |
| 1537 | ADCk.13.glcm.energy.2.range. |
| 1538 | ADCk.13.glcm.energy.3.mean. |
| 1539 | ADCk.13.glcm.energy.3.range. |
| 1540 | ADCk.13.glcm.energy.4.mean. |
| 1541 | ADCk.13.glcm.energy.4.range. |
| 1542 | ADCk.13.glcm.correlation.1.mean. |
| 1543 | ADCk.13.glcm.correlation.1.range. |
| 1544 | ADCk.13.glcm.correlation.2.mean. |
| 1545 | ADCk.13.glcm.correlation.2.range. |
| 1546 | ADCk.13.glcm.correlation.3.mean. |
| 1547 | ADCk.13.glcm.correlation.3.range. |
| 1548 | ADCk.13.glcm.correlation.4.mean. |
| 1549 | ADCk.13.glcm.correlation.4.range. |
| 1550 | ADCk.13.glcm.ASM.1.mean. |
| 1551 | ADCk.13.glcm.ASM.1.range. |
| 1552 | ADCk.13.glcm.ASM.2.mean. |
| 1553 | ADCk.13.glcm.ASM.2.range. |
| 1554 | ADCk.13.glcm.ASM.3.mean. |
| 1555 | ADCk.13.glcm.ASM.3.range. |
| 1556 | ADCk.13.glcm.ASM.4.mean. |
| 1557 | ADCk.13.glcm.ASM.4.range. |
| 1558 | ADCk.15.glcm.contrast.1.mean. |
| 1559 | ADCk.15.glcm.contrast.1.range. |
| 1560 | ADCk.15.glcm.contrast.2.mean. |
| 1561 | ADCk.15.glcm.contrast.2.range. |
| 1562 | ADCk.15.glcm.contrast.3.mean. |
| 1563 | ADCk.15.glcm.contrast.3.range. |
| 1564 | ADCk.15.glcm.contrast.4.mean. |
| 1565 | ADCk.15.glcm.contrast.4.range. |
| 1566 | ADCk.15.glcm.dissimilarity.1.mean. |
| 1567 | ADCk.15.glcm.dissimilarity.1.range. |
| 1568 | ADCk.15.glcm.dissimilarity.2.mean. |
| 1569 | ADCk.15.glcm.dissimilarity.2.range. |
| 1570 | ADCk.15.glcm.dissimilarity.3.mean. |
| 1571 | ADCk.15.glcm.dissimilarity.3.range. |
| 1572 | ADCk.15.glcm.dissimilarity.4.mean. |
| 1573 | ADCk.15.glcm.dissimilarity.4.range. |
| 1574 | ADCk.15.glcm.homogeneity.1.mean. |
| 1575 | ADCk.15.glcm.homogeneity.1.range. |
| 1576 | ADCk.15.glcm.homogeneity.2.mean. |
| 1577 | ADCk.15.glcm.homogeneity.2.range. |
| 1578 | ADCk.15.glcm.homogeneity.3.mean. |
| 1579 | ADCk.15.glcm.homogeneity.3.range. |
| 1580 | ADCk.15.glcm.homogeneity.4.mean. |
| 1581 | ADCk.15.glcm.homogeneity.4.range. |
| 1582 | ADCk.15.glcm.energy.1.mean. |
| 1583 | ADCk.15.glcm.energy.1.range. |
| 1584 | ADCk.15.glcm.energy.2.mean. |
| 1585 | ADCk.15.glcm.energy.2.range. |
| 1586 | ADCk.15.glcm.energy.3.mean. |
| 1587 | ADCk.15.glcm.energy.3.range. |
| 1588 | ADCk.15.glcm.energy.4.mean. |
| 1589 | ADCk.15.glcm.energy.4.range. |
| 1590 | ADCk.15.glcm.correlation.1.mean. |
| 1591 | ADCk.15.glcm.correlation.1.range. |
| 1592 | ADCk.15.glcm.correlation.2.mean. |
| 1593 | ADCk.15.glcm.correlation.2.range. |
| 1594 | ADCk.15.glcm.correlation.3.mean. |
| 1595 | ADCk.15.glcm.correlation.3.range. |
| 1596 | ADCk.15.glcm.correlation.4.mean. |
| 1597 | ADCk.15.glcm.correlation.4.range. |
| 1598 | ADCk.15.glcm.ASM.1.mean. |
| 1599 | ADCk.15.glcm.ASM.1.range. |
| 1600 | ADCk.15.glcm.ASM.2.mean. |
| 1601 | ADCk.15.glcm.ASM.2.range. |
| 1602 | ADCk.15.glcm.ASM.3.mean. |
| 1603 | ADCk.15.glcm.ASM.3.range. |
| 1604 | ADCk.15.glcm.ASM.4.mean. |
| 1605 | ADCk.15.glcm.ASM.4.range. |
| 1606 | ADCk.mbb.glcm.contrast.1.mean. |
| 1607 | ADCk.mbb.glcm.contrast.1.range. |
| 1608 | ADCk.mbb.glcm.contrast.2.mean. |
| 1609 | ADCk.mbb.glcm.contrast.2.range. |
| 1610 | ADCk.mbb.glcm.contrast.3.mean. |
| 1611 | ADCk.mbb.glcm.contrast.3.range. |
| 1612 | ADCk.mbb.glcm.contrast.4.mean. |
| 1613 | ADCk.mbb.glcm.contrast.4.range. |
| 1614 | ADCk.mbb.glcm.dissimilarity.1.mean. |
| 1615 | ADCk.mbb.glcm.dissimilarity.1.range. |
| 1616 | ADCk.mbb.glcm.dissimilarity.2.mean. |
| 1617 | ADCk.mbb.glcm.dissimilarity.2.range. |
| 1618 | ADCk.mbb.glcm.dissimilarity.3.mean. |
| 1619 | ADCk.mbb.glcm.dissimilarity.3.range. |
| 1620 | ADCk.mbb.glcm.dissimilarity.4.mean. |
| 1621 | ADCk.mbb.glcm.dissimilarity.4.range. |
| 1622 | ADCk.mbb.glcm.homogeneity.1.mean. |
| 1623 | ADCk.mbb.glcm.homogeneity.1.range. |
| 1624 | ADCk.mbb.glcm.homogeneity.2.mean. |
| 1625 | ADCk.mbb.glcm.homogeneity.2.range. |
| 1626 | ADCk.mbb.glcm.homogeneity.3.mean. |
| 1627 | ADCk.mbb.glcm.homogeneity.3.range. |
| 1628 | ADCk.mbb.glcm.homogeneity.4.mean. |
| 1629 | ADCk.mbb.glcm.homogeneity.4.range. |
| 1630 | ADCk.mbb.glcm.energy.1.mean. |
| 1631 | ADCk.mbb.glcm.energy.1.range. |
| 1632 | ADCk.mbb.glcm.energy.2.mean. |
| 1633 | ADCk.mbb.glcm.energy.2.range. |
| 1634 | ADCk.mbb.glcm.energy.3.mean. |
| 1635 | ADCk.mbb.glcm.energy.3.range. |
| 1636 | ADCk.mbb.glcm.energy.4.mean. |
| 1637 | ADCk.mbb.glcm.energy.4.range. |
| 1638 | ADCk.mbb.glcm.correlation.1.mean. |
| 1639 | ADCk.mbb.glcm.correlation.1.range. |
| 1640 | ADCk.mbb.glcm.correlation.2.mean. |
| 1641 | ADCk.mbb.glcm.correlation.2.range. |
| 1642 | ADCk.mbb.glcm.correlation.3.mean. |
| 1643 | ADCk.mbb.glcm.correlation.3.range. |
| 1644 | ADCk.mbb.glcm.correlation.4.mean. |
| 1645 | ADCk.mbb.glcm.correlation.4.range. |
| 1646 | ADCk.mbb.glcm.ASM.1.mean. |
| 1647 | ADCk.mbb.glcm.ASM.1.range. |
| 1648 | ADCk.mbb.glcm.ASM.2.mean. |
| 1649 | ADCk.mbb.glcm.ASM.2.range. |
| 1650 | ADCk.mbb.glcm.ASM.3.mean. |
| 1651 | ADCk.mbb.glcm.ASM.3.range. |
| 1652 | ADCk.mbb.glcm.ASM.4.mean. |
| 1653 | ADCk.mbb.glcm.ASM.4.range. |
| 1654 | ADCk.3.lbp.1.0. |
| 1655 | ADCk.3.lbp.1.1. |
| 1656 | ADCk.3.lbp.1.2. |
| 1657 | ADCk.3.lbp.1.3. |
| 1658 | ADCk.3.lbp.1.4. |
| 1659 | ADCk.3.lbp.1.5. |
| 1660 | ADCk.3.lbp.1.6. |
| 1661 | ADCk.3.lbp.1.7. |
| 1662 | ADCk.3.lbp.1.8. |
| 1663 | ADCk.3.lbp.1.9. |
| 1664 | ADCk.5.lbp.2.0. |
| 1665 | ADCk.5.lbp.2.1. |
| 1666 | ADCk.5.lbp.2.2. |
| 1667 | ADCk.5.lbp.2.3. |
| 1668 | ADCk.5.lbp.2.4. |
| 1669 | ADCk.5.lbp.2.5. |
| 1670 | ADCk.5.lbp.2.6. |
| 1671 | ADCk.5.lbp.2.7. |
| 1672 | ADCk.5.lbp.2.8. |
| 1673 | ADCk.5.lbp.2.9. |
| 1674 | ADCk.7.lbp.3.0. |
| 1675 | ADCk.7.lbp.3.1. |
| 1676 | ADCk.7.lbp.3.2. |
| 1677 | ADCk.7.lbp.3.3. |
| 1678 | ADCk.7.lbp.3.4. |
| 1679 | ADCk.7.lbp.3.5. |
| 1680 | ADCk.7.lbp.3.6. |
| 1681 | ADCk.7.lbp.3.7. |
| 1682 | ADCk.7.lbp.3.8. |
| 1683 | ADCk.7.lbp.3.9. |
| 1684 | ADCk.9.lbp.4.0. |
| 1685 | ADCk.9.lbp.4.1. |
| 1686 | ADCk.9.lbp.4.2. |
| 1687 | ADCk.9.lbp.4.3. |
| 1688 | ADCk.9.lbp.4.4. |
| 1689 | ADCk.9.lbp.4.5. |
| 1690 | ADCk.9.lbp.4.6. |
| 1691 | ADCk.9.lbp.4.7. |
| 1692 | ADCk.9.lbp.4.8. |
| 1693 | ADCk.9.lbp.4.9. |
| 1694 | ADCk.11.lbp.5.0. |
| 1695 | ADCk.11.lbp.5.1. |
| 1696 | ADCk.11.lbp.5.2. |
| 1697 | ADCk.11.lbp.5.3. |
| 1698 | ADCk.11.lbp.5.4. |
| 1699 | ADCk.11.lbp.5.5. |
| 1700 | ADCk.11.lbp.5.6. |
| 1701 | ADCk.11.lbp.5.7. |
| 1702 | ADCk.11.lbp.5.8. |
| 1703 | ADCk.11.lbp.5.9. |
| 1704 | ADCk.13.lbp.6.0. |
| 1705 | ADCk.13.lbp.6.1. |
| 1706 | ADCk.13.lbp.6.2. |
| 1707 | ADCk.13.lbp.6.3. |
| 1708 | ADCk.13.lbp.6.4. |
| 1709 | ADCk.13.lbp.6.5. |
| 1710 | ADCk.13.lbp.6.6. |
| 1711 | ADCk.13.lbp.6.7. |
| 1712 | ADCk.13.lbp.6.8. |
| 1713 | ADCk.13.lbp.6.9. |
| 1714 | ADCk.15.lbp.7.0. |
| 1715 | ADCk.15.lbp.7.1. |
| 1716 | ADCk.15.lbp.7.2. |
| 1717 | ADCk.15.lbp.7.3. |
| 1718 | ADCk.15.lbp.7.4. |
| 1719 | ADCk.15.lbp.7.5. |
| 1720 | ADCk.15.lbp.7.6. |
| 1721 | ADCk.15.lbp.7.7. |
| 1722 | ADCk.15.lbp.7.8. |
| 1723 | ADCk.15.lbp.7.9. |
| 1724 | ADCk.3.hog |
| 1725 | ADCk.5.hog |
| 1726 | ADCk.7.hog |
| 1727 | ADCk.9.hog |
| 1728 | ADCk.11.hog |
| 1729 | ADCk.13.hog |
| 1730 | ADCk.15.hog |
| 1731 | ADCk.3.gabor.1.0.1.mean. |
| 1732 | ADCk.3.gabor.1.0.1.var. |
| 1733 | ADCk.3.gabor.1.0.1.absmean. |
| 1734 | ADCk.3.gabor.1.0.1.mag. |
| 1735 | ADCk.3.gabor.1.0.2.mean. |
| 1736 | ADCk.3.gabor.1.0.2.var. |
| 1737 | ADCk.3.gabor.1.0.2.absmean. |
| 1738 | ADCk.3.gabor.1.0.2.mag. |
| 1739 | ADCk.3.gabor.1.0.3.mean. |
| 1740 | ADCk.3.gabor.1.0.3.var. |
| 1741 | ADCk.3.gabor.1.0.3.absmean. |
| 1742 | ADCk.3.gabor.1.0.3.mag. |
| 1743 | ADCk.3.gabor.1.0.4.mean. |
| 1744 | ADCk.3.gabor.1.0.4.var. |
| 1745 | ADCk.3.gabor.1.0.4.absmean. |
| 1746 | ADCk.3.gabor.1.0.4.mag. |
| 1747 | ADCk.3.gabor.1.0.5.mean. |
| 1748 | ADCk.3.gabor.1.0.5.var. |
| 1749 | ADCk.3.gabor.1.0.5.absmean. |
| 1750 | ADCk.3.gabor.1.0.5.mag. |
| 1751 | ADCk.3.gabor.2.0.1.mean. |
| 1752 | ADCk.3.gabor.2.0.1.var. |
| 1753 | ADCk.3.gabor.2.0.1.absmean. |
| 1754 | ADCk.3.gabor.2.0.1.mag. |
| 1755 | ADCk.3.gabor.2.0.2.mean. |
| 1756 | ADCk.3.gabor.2.0.2.var. |
| 1757 | ADCk.3.gabor.2.0.2.absmean. |
| 1758 | ADCk.3.gabor.2.0.2.mag. |
| 1759 | ADCk.3.gabor.2.0.3.mean. |
| 1760 | ADCk.3.gabor.2.0.3.var. |
| 1761 | ADCk.3.gabor.2.0.3.absmean. |
| 1762 | ADCk.3.gabor.2.0.3.mag. |
| 1763 | ADCk.3.gabor.2.0.4.mean. |
| 1764 | ADCk.3.gabor.2.0.4.var. |
| 1765 | ADCk.3.gabor.2.0.4.absmean. |
| 1766 | ADCk.3.gabor.2.0.4.mag. |
| 1767 | ADCk.3.gabor.2.0.5.mean. |
| 1768 | ADCk.3.gabor.2.0.5.var. |
| 1769 | ADCk.3.gabor.2.0.5.absmean. |
| 1770 | ADCk.3.gabor.2.0.5.mag. |
| 1771 | ADCk.3.gabor.3.0.1.mean. |
| 1772 | ADCk.3.gabor.3.0.1.var. |
| 1773 | ADCk.3.gabor.3.0.1.absmean. |
| 1774 | ADCk.3.gabor.3.0.1.mag. |
| 1775 | ADCk.3.gabor.3.0.2.mean. |
| 1776 | ADCk.3.gabor.3.0.2.var. |
| 1777 | ADCk.3.gabor.3.0.2.absmean. |
| 1778 | ADCk.3.gabor.3.0.2.mag. |
| 1779 | ADCk.3.gabor.3.0.3.mean. |
| 1780 | ADCk.3.gabor.3.0.3.var. |
| 1781 | ADCk.3.gabor.3.0.3.absmean. |
| 1782 | ADCk.3.gabor.3.0.3.mag. |
| 1783 | ADCk.3.gabor.3.0.4.mean. |
| 1784 | ADCk.3.gabor.3.0.4.var. |
| 1785 | ADCk.3.gabor.3.0.4.absmean. |
| 1786 | ADCk.3.gabor.3.0.4.mag. |
| 1787 | ADCk.3.gabor.3.0.5.mean. |
| 1788 | ADCk.3.gabor.3.0.5.var. |
| 1789 | ADCk.3.gabor.3.0.5.absmean. |
| 1790 | ADCk.3.gabor.3.0.5.mag. |
| 1791 | ADCk.5.gabor.1.0.1.mean. |
| 1792 | ADCk.5.gabor.1.0.1.var. |
| 1793 | ADCk.5.gabor.1.0.1.absmean. |
| 1794 | ADCk.5.gabor.1.0.1.mag. |
| 1795 | ADCk.5.gabor.1.0.2.mean. |
| 1796 | ADCk.5.gabor.1.0.2.var. |
| 1797 | ADCk.5.gabor.1.0.2.absmean. |
| 1798 | ADCk.5.gabor.1.0.2.mag. |
| 1799 | ADCk.5.gabor.1.0.3.mean. |
| 1800 | ADCk.5.gabor.1.0.3.var. |
| 1801 | ADCk.5.gabor.1.0.3.absmean. |
| 1802 | ADCk.5.gabor.1.0.3.mag. |
| 1803 | ADCk.5.gabor.1.0.4.mean. |
| 1804 | ADCk.5.gabor.1.0.4.var. |
| 1805 | ADCk.5.gabor.1.0.4.absmean. |
| 1806 | ADCk.5.gabor.1.0.4.mag. |
| 1807 | ADCk.5.gabor.1.0.5.mean. |
| 1808 | ADCk.5.gabor.1.0.5.var. |
| 1809 | ADCk.5.gabor.1.0.5.absmean. |
| 1810 | ADCk.5.gabor.1.0.5.mag. |
| 1811 | ADCk.5.gabor.2.0.1.mean. |
| 1812 | ADCk.5.gabor.2.0.1.var. |
| 1813 | ADCk.5.gabor.2.0.1.absmean. |
| 1814 | ADCk.5.gabor.2.0.1.mag. |
| 1815 | ADCk.5.gabor.2.0.2.mean. |
| 1816 | ADCk.5.gabor.2.0.2.var. |
| 1817 | ADCk.5.gabor.2.0.2.absmean. |
| 1818 | ADCk.5.gabor.2.0.2.mag. |
| 1819 | ADCk.5.gabor.2.0.3.mean. |
| 1820 | ADCk.5.gabor.2.0.3.var. |
| 1821 | ADCk.5.gabor.2.0.3.absmean. |
| 1822 | ADCk.5.gabor.2.0.3.mag. |
| 1823 | ADCk.5.gabor.2.0.4.mean. |
| 1824 | ADCk.5.gabor.2.0.4.var. |
| 1825 | ADCk.5.gabor.2.0.4.absmean. |
| 1826 | ADCk.5.gabor.2.0.4.mag. |
| 1827 | ADCk.5.gabor.2.0.5.mean. |
| 1828 | ADCk.5.gabor.2.0.5.var. |
| 1829 | ADCk.5.gabor.2.0.5.absmean. |
| 1830 | ADCk.5.gabor.2.0.5.mag. |
| 1831 | ADCk.5.gabor.3.0.1.mean. |
| 1832 | ADCk.5.gabor.3.0.1.var. |
| 1833 | ADCk.5.gabor.3.0.1.absmean. |
| 1834 | ADCk.5.gabor.3.0.1.mag. |
| 1835 | ADCk.5.gabor.3.0.2.mean. |
| 1836 | ADCk.5.gabor.3.0.2.var. |
| 1837 | ADCk.5.gabor.3.0.2.absmean. |
| 1838 | ADCk.5.gabor.3.0.2.mag. |
| 1839 | ADCk.5.gabor.3.0.3.mean. |
| 1840 | ADCk.5.gabor.3.0.3.var. |
| 1841 | ADCk.5.gabor.3.0.3.absmean. |
| 1842 | ADCk.5.gabor.3.0.3.mag. |
| 1843 | ADCk.5.gabor.3.0.4.mean. |
| 1844 | ADCk.5.gabor.3.0.4.var. |
| 1845 | ADCk.5.gabor.3.0.4.absmean. |
| 1846 | ADCk.5.gabor.3.0.4.mag. |
| 1847 | ADCk.5.gabor.3.0.5.mean. |
| 1848 | ADCk.5.gabor.3.0.5.var. |
| 1849 | ADCk.5.gabor.3.0.5.absmean. |
| 1850 | ADCk.5.gabor.3.0.5.mag. |
| 1851 | ADCk.7.gabor.1.0.1.mean. |
| 1852 | ADCk.7.gabor.1.0.1.var. |
| 1853 | ADCk.7.gabor.1.0.1.absmean. |
| 1854 | ADCk.7.gabor.1.0.1.mag. |
| 1855 | ADCk.7.gabor.1.0.2.mean. |
| 1856 | ADCk.7.gabor.1.0.2.var. |
| 1857 | ADCk.7.gabor.1.0.2.absmean. |
| 1858 | ADCk.7.gabor.1.0.2.mag. |
| 1859 | ADCk.7.gabor.1.0.3.mean. |
| 1860 | ADCk.7.gabor.1.0.3.var. |
| 1861 | ADCk.7.gabor.1.0.3.absmean. |
| 1862 | ADCk.7.gabor.1.0.3.mag. |
| 1863 | ADCk.7.gabor.1.0.4.mean. |
| 1864 | ADCk.7.gabor.1.0.4.var. |
| 1865 | ADCk.7.gabor.1.0.4.absmean. |
| 1866 | ADCk.7.gabor.1.0.4.mag. |
| 1867 | ADCk.7.gabor.1.0.5.mean. |
| 1868 | ADCk.7.gabor.1.0.5.var. |
| 1869 | ADCk.7.gabor.1.0.5.absmean. |
| 1870 | ADCk.7.gabor.1.0.5.mag. |
| 1871 | ADCk.7.gabor.2.0.1.mean. |
| 1872 | ADCk.7.gabor.2.0.1.var. |
| 1873 | ADCk.7.gabor.2.0.1.absmean. |
| 1874 | ADCk.7.gabor.2.0.1.mag. |
| 1875 | ADCk.7.gabor.2.0.2.mean. |
| 1876 | ADCk.7.gabor.2.0.2.var. |
| 1877 | ADCk.7.gabor.2.0.2.absmean. |
| 1878 | ADCk.7.gabor.2.0.2.mag. |
| 1879 | ADCk.7.gabor.2.0.3.mean. |
| 1880 | ADCk.7.gabor.2.0.3.var. |
| 1881 | ADCk.7.gabor.2.0.3.absmean. |
| 1882 | ADCk.7.gabor.2.0.3.mag. |
| 1883 | ADCk.7.gabor.2.0.4.mean. |
| 1884 | ADCk.7.gabor.2.0.4.var. |
| 1885 | ADCk.7.gabor.2.0.4.absmean. |
| 1886 | ADCk.7.gabor.2.0.4.mag. |
| 1887 | ADCk.7.gabor.2.0.5.mean. |
| 1888 | ADCk.7.gabor.2.0.5.var. |
| 1889 | ADCk.7.gabor.2.0.5.absmean. |
| 1890 | ADCk.7.gabor.2.0.5.mag. |
| 1891 | ADCk.7.gabor.3.0.1.mean. |
| 1892 | ADCk.7.gabor.3.0.1.var. |
| 1893 | ADCk.7.gabor.3.0.1.absmean. |
| 1894 | ADCk.7.gabor.3.0.1.mag. |
| 1895 | ADCk.7.gabor.3.0.2.mean. |
| 1896 | ADCk.7.gabor.3.0.2.var. |
| 1897 | ADCk.7.gabor.3.0.2.absmean. |
| 1898 | ADCk.7.gabor.3.0.2.mag. |
| 1899 | ADCk.7.gabor.3.0.3.mean. |
| 1900 | ADCk.7.gabor.3.0.3.var. |
| 1901 | ADCk.7.gabor.3.0.3.absmean. |
| 1902 | ADCk.7.gabor.3.0.3.mag. |
| 1903 | ADCk.7.gabor.3.0.4.mean. |
| 1904 | ADCk.7.gabor.3.0.4.var. |
| 1905 | ADCk.7.gabor.3.0.4.absmean. |
| 1906 | ADCk.7.gabor.3.0.4.mag. |
| 1907 | ADCk.7.gabor.3.0.5.mean. |
| 1908 | ADCk.7.gabor.3.0.5.var. |
| 1909 | ADCk.7.gabor.3.0.5.absmean. |
| 1910 | ADCk.7.gabor.3.0.5.mag. |
| 1911 | ADCk.9.gabor.1.0.1.mean. |
| 1912 | ADCk.9.gabor.1.0.1.var. |
| 1913 | ADCk.9.gabor.1.0.1.absmean. |
| 1914 | ADCk.9.gabor.1.0.1.mag. |
| 1915 | ADCk.9.gabor.1.0.2.mean. |
| 1916 | ADCk.9.gabor.1.0.2.var. |
| 1917 | ADCk.9.gabor.1.0.2.absmean. |
| 1918 | ADCk.9.gabor.1.0.2.mag. |
| 1919 | ADCk.9.gabor.1.0.3.mean. |
| 1920 | ADCk.9.gabor.1.0.3.var. |
| 1921 | ADCk.9.gabor.1.0.3.absmean. |
| 1922 | ADCk.9.gabor.1.0.3.mag. |
| 1923 | ADCk.9.gabor.1.0.4.mean. |
| 1924 | ADCk.9.gabor.1.0.4.var. |
| 1925 | ADCk.9.gabor.1.0.4.absmean. |
| 1926 | ADCk.9.gabor.1.0.4.mag. |
| 1927 | ADCk.9.gabor.1.0.5.mean. |
| 1928 | ADCk.9.gabor.1.0.5.var. |
| 1929 | ADCk.9.gabor.1.0.5.absmean. |
| 1930 | ADCk.9.gabor.1.0.5.mag. |
| 1931 | ADCk.9.gabor.2.0.1.mean. |
| 1932 | ADCk.9.gabor.2.0.1.var. |
| 1933 | ADCk.9.gabor.2.0.1.absmean. |
| 1934 | ADCk.9.gabor.2.0.1.mag. |
| 1935 | ADCk.9.gabor.2.0.2.mean. |
| 1936 | ADCk.9.gabor.2.0.2.var. |
| 1937 | ADCk.9.gabor.2.0.2.absmean. |
| 1938 | ADCk.9.gabor.2.0.2.mag. |
| 1939 | ADCk.9.gabor.2.0.3.mean. |
| 1940 | ADCk.9.gabor.2.0.3.var. |
| 1941 | ADCk.9.gabor.2.0.3.absmean. |
| 1942 | ADCk.9.gabor.2.0.3.mag. |
| 1943 | ADCk.9.gabor.2.0.4.mean. |
| 1944 | ADCk.9.gabor.2.0.4.var. |
| 1945 | ADCk.9.gabor.2.0.4.absmean. |
| 1946 | ADCk.9.gabor.2.0.4.mag. |
| 1947 | ADCk.9.gabor.2.0.5.mean. |
| 1948 | ADCk.9.gabor.2.0.5.var. |
| 1949 | ADCk.9.gabor.2.0.5.absmean. |
| 1950 | ADCk.9.gabor.2.0.5.mag. |
| 1951 | ADCk.9.gabor.3.0.1.mean. |
| 1952 | ADCk.9.gabor.3.0.1.var. |
| 1953 | ADCk.9.gabor.3.0.1.absmean. |
| 1954 | ADCk.9.gabor.3.0.1.mag. |
| 1955 | ADCk.9.gabor.3.0.2.mean. |
| 1956 | ADCk.9.gabor.3.0.2.var. |
| 1957 | ADCk.9.gabor.3.0.2.absmean. |
| 1958 | ADCk.9.gabor.3.0.2.mag. |
| 1959 | ADCk.9.gabor.3.0.3.mean. |
| 1960 | ADCk.9.gabor.3.0.3.var. |
| 1961 | ADCk.9.gabor.3.0.3.absmean. |
| 1962 | ADCk.9.gabor.3.0.3.mag. |
| 1963 | ADCk.9.gabor.3.0.4.mean. |
| 1964 | ADCk.9.gabor.3.0.4.var. |
| 1965 | ADCk.9.gabor.3.0.4.absmean. |
| 1966 | ADCk.9.gabor.3.0.4.mag. |
| 1967 | ADCk.9.gabor.3.0.5.mean. |
| 1968 | ADCk.9.gabor.3.0.5.var. |
| 1969 | ADCk.9.gabor.3.0.5.absmean. |
| 1970 | ADCk.9.gabor.3.0.5.mag. |
| 1971 | ADCk.11.gabor.1.0.1.mean. |
| 1972 | ADCk.11.gabor.1.0.1.var. |
| 1973 | ADCk.11.gabor.1.0.1.absmean. |
| 1974 | ADCk.11.gabor.1.0.1.mag. |
| 1975 | ADCk.11.gabor.1.0.2.mean. |
| 1976 | ADCk.11.gabor.1.0.2.var. |
| 1977 | ADCk.11.gabor.1.0.2.absmean. |
| 1978 | ADCk.11.gabor.1.0.2.mag. |
| 1979 | ADCk.11.gabor.1.0.3.mean. |
| 1980 | ADCk.11.gabor.1.0.3.var. |
| 1981 | ADCk.11.gabor.1.0.3.absmean. |
| 1982 | ADCk.11.gabor.1.0.3.mag. |
| 1983 | ADCk.11.gabor.1.0.4.mean. |
| 1984 | ADCk.11.gabor.1.0.4.var. |
| 1985 | ADCk.11.gabor.1.0.4.absmean. |
| 1986 | ADCk.11.gabor.1.0.4.mag. |
| 1987 | ADCk.11.gabor.1.0.5.mean. |
| 1988 | ADCk.11.gabor.1.0.5.var. |
| 1989 | ADCk.11.gabor.1.0.5.absmean. |
| 1990 | ADCk.11.gabor.1.0.5.mag. |
| 1991 | ADCk.11.gabor.2.0.1.mean. |
| 1992 | ADCk.11.gabor.2.0.1.var. |
| 1993 | ADCk.11.gabor.2.0.1.absmean. |
| 1994 | ADCk.11.gabor.2.0.1.mag. |
| 1995 | ADCk.11.gabor.2.0.2.mean. |
| 1996 | ADCk.11.gabor.2.0.2.var. |
| 1997 | ADCk.11.gabor.2.0.2.absmean. |
| 1998 | ADCk.11.gabor.2.0.2.mag. |
| 1999 | ADCk.11.gabor.2.0.3.mean. |
| 2000 | ADCk.11.gabor.2.0.3.var. |
| 2001 | ADCk.11.gabor.2.0.3.absmean. |
| 2002 | ADCk.11.gabor.2.0.3.mag. |
| 2003 | ADCk.11.gabor.2.0.4.mean. |
| 2004 | ADCk.11.gabor.2.0.4.var. |
| 2005 | ADCk.11.gabor.2.0.4.absmean. |
| 2006 | ADCk.11.gabor.2.0.4.mag. |
| 2007 | ADCk.11.gabor.2.0.5.mean. |
| 2008 | ADCk.11.gabor.2.0.5.var. |
| 2009 | ADCk.11.gabor.2.0.5.absmean. |
| 2010 | ADCk.11.gabor.2.0.5.mag. |
| 2011 | ADCk.11.gabor.3.0.1.mean. |
| 2012 | ADCk.11.gabor.3.0.1.var. |
| 2013 | ADCk.11.gabor.3.0.1.absmean. |
| 2014 | ADCk.11.gabor.3.0.1.mag. |
| 2015 | ADCk.11.gabor.3.0.2.mean. |
| 2016 | ADCk.11.gabor.3.0.2.var. |
| 2017 | ADCk.11.gabor.3.0.2.absmean. |
| 2018 | ADCk.11.gabor.3.0.2.mag. |
| 2019 | ADCk.11.gabor.3.0.3.mean. |
| 2020 | ADCk.11.gabor.3.0.3.var. |
| 2021 | ADCk.11.gabor.3.0.3.absmean. |
| 2022 | ADCk.11.gabor.3.0.3.mag. |
| 2023 | ADCk.11.gabor.3.0.4.mean. |
| 2024 | ADCk.11.gabor.3.0.4.var. |
| 2025 | ADCk.11.gabor.3.0.4.absmean. |
| 2026 | ADCk.11.gabor.3.0.4.mag. |
| 2027 | ADCk.11.gabor.3.0.5.mean. |
| 2028 | ADCk.11.gabor.3.0.5.var. |
| 2029 | ADCk.11.gabor.3.0.5.absmean. |
| 2030 | ADCk.11.gabor.3.0.5.mag. |
| 2031 | ADCk.13.gabor.1.0.1.mean. |
| 2032 | ADCk.13.gabor.1.0.1.var. |
| 2033 | ADCk.13.gabor.1.0.1.absmean. |
| 2034 | ADCk.13.gabor.1.0.1.mag. |
| 2035 | ADCk.13.gabor.1.0.2.mean. |
| 2036 | ADCk.13.gabor.1.0.2.var. |
| 2037 | ADCk.13.gabor.1.0.2.absmean. |
| 2038 | ADCk.13.gabor.1.0.2.mag. |
| 2039 | ADCk.13.gabor.1.0.3.mean. |
| 2040 | ADCk.13.gabor.1.0.3.var. |
| 2041 | ADCk.13.gabor.1.0.3.absmean. |
| 2042 | ADCk.13.gabor.1.0.3.mag. |
| 2043 | ADCk.13.gabor.1.0.4.mean. |
| 2044 | ADCk.13.gabor.1.0.4.var. |
| 2045 | ADCk.13.gabor.1.0.4.absmean. |
| 2046 | ADCk.13.gabor.1.0.4.mag. |
| 2047 | ADCk.13.gabor.1.0.5.mean. |
| 2048 | ADCk.13.gabor.1.0.5.var. |
| 2049 | ADCk.13.gabor.1.0.5.absmean. |
| 2050 | ADCk.13.gabor.1.0.5.mag. |
| 2051 | ADCk.13.gabor.2.0.1.mean. |
| 2052 | ADCk.13.gabor.2.0.1.var. |
| 2053 | ADCk.13.gabor.2.0.1.absmean. |
| 2054 | ADCk.13.gabor.2.0.1.mag. |
| 2055 | ADCk.13.gabor.2.0.2.mean. |
| 2056 | ADCk.13.gabor.2.0.2.var. |
| 2057 | ADCk.13.gabor.2.0.2.absmean. |
| 2058 | ADCk.13.gabor.2.0.2.mag. |
| 2059 | ADCk.13.gabor.2.0.3.mean. |
| 2060 | ADCk.13.gabor.2.0.3.var. |
| 2061 | ADCk.13.gabor.2.0.3.absmean. |
| 2062 | ADCk.13.gabor.2.0.3.mag. |
| 2063 | ADCk.13.gabor.2.0.4.mean. |
| 2064 | ADCk.13.gabor.2.0.4.var. |
| 2065 | ADCk.13.gabor.2.0.4.absmean. |
| 2066 | ADCk.13.gabor.2.0.4.mag. |
| 2067 | ADCk.13.gabor.2.0.5.mean. |
| 2068 | ADCk.13.gabor.2.0.5.var. |
| 2069 | ADCk.13.gabor.2.0.5.absmean. |
| 2070 | ADCk.13.gabor.2.0.5.mag. |
| 2071 | ADCk.13.gabor.3.0.1.mean. |
| 2072 | ADCk.13.gabor.3.0.1.var. |
| 2073 | ADCk.13.gabor.3.0.1.absmean. |
| 2074 | ADCk.13.gabor.3.0.1.mag. |
| 2075 | ADCk.13.gabor.3.0.2.mean. |
| 2076 | ADCk.13.gabor.3.0.2.var. |
| 2077 | ADCk.13.gabor.3.0.2.absmean. |
| 2078 | ADCk.13.gabor.3.0.2.mag. |
| 2079 | ADCk.13.gabor.3.0.3.mean. |
| 2080 | ADCk.13.gabor.3.0.3.var. |
| 2081 | ADCk.13.gabor.3.0.3.absmean. |
| 2082 | ADCk.13.gabor.3.0.3.mag. |
| 2083 | ADCk.13.gabor.3.0.4.mean. |
| 2084 | ADCk.13.gabor.3.0.4.var. |
| 2085 | ADCk.13.gabor.3.0.4.absmean. |
| 2086 | ADCk.13.gabor.3.0.4.mag. |
| 2087 | ADCk.13.gabor.3.0.5.mean. |
| 2088 | ADCk.13.gabor.3.0.5.var. |
| 2089 | ADCk.13.gabor.3.0.5.absmean. |
| 2090 | ADCk.13.gabor.3.0.5.mag. |
| 2091 | ADCk.15.gabor.1.0.1.mean. |
| 2092 | ADCk.15.gabor.1.0.1.var. |
| 2093 | ADCk.15.gabor.1.0.1.absmean. |
| 2094 | ADCk.15.gabor.1.0.1.mag. |
| 2095 | ADCk.15.gabor.1.0.2.mean. |
| 2096 | ADCk.15.gabor.1.0.2.var. |
| 2097 | ADCk.15.gabor.1.0.2.absmean. |
| 2098 | ADCk.15.gabor.1.0.2.mag. |
| 2099 | ADCk.15.gabor.1.0.3.mean. |
| 2100 | ADCk.15.gabor.1.0.3.var. |
| 2101 | ADCk.15.gabor.1.0.3.absmean. |
| 2102 | ADCk.15.gabor.1.0.3.mag. |
| 2103 | ADCk.15.gabor.1.0.4.mean. |
| 2104 | ADCk.15.gabor.1.0.4.var. |
| 2105 | ADCk.15.gabor.1.0.4.absmean. |
| 2106 | ADCk.15.gabor.1.0.4.mag. |
| 2107 | ADCk.15.gabor.1.0.5.mean. |
| 2108 | ADCk.15.gabor.1.0.5.var. |
| 2109 | ADCk.15.gabor.1.0.5.absmean. |
| 2110 | ADCk.15.gabor.1.0.5.mag. |
| 2111 | ADCk.15.gabor.2.0.1.mean. |
| 2112 | ADCk.15.gabor.2.0.1.var. |
| 2113 | ADCk.15.gabor.2.0.1.absmean. |
| 2114 | ADCk.15.gabor.2.0.1.mag. |
| 2115 | ADCk.15.gabor.2.0.2.mean. |
| 2116 | ADCk.15.gabor.2.0.2.var. |
| 2117 | ADCk.15.gabor.2.0.2.absmean. |
| 2118 | ADCk.15.gabor.2.0.2.mag. |
| 2119 | ADCk.15.gabor.2.0.3.mean. |
| 2120 | ADCk.15.gabor.2.0.3.var. |
| 2121 | ADCk.15.gabor.2.0.3.absmean. |
| 2122 | ADCk.15.gabor.2.0.3.mag. |
| 2123 | ADCk.15.gabor.2.0.4.mean. |
| 2124 | ADCk.15.gabor.2.0.4.var. |
| 2125 | ADCk.15.gabor.2.0.4.absmean. |
| 2126 | ADCk.15.gabor.2.0.4.mag. |
| 2127 | ADCk.15.gabor.2.0.5.mean. |
| 2128 | ADCk.15.gabor.2.0.5.var. |
| 2129 | ADCk.15.gabor.2.0.5.absmean. |
| 2130 | ADCk.15.gabor.2.0.5.mag. |
| 2131 | ADCk.15.gabor.3.0.1.mean. |
| 2132 | ADCk.15.gabor.3.0.1.var. |
| 2133 | ADCk.15.gabor.3.0.1.absmean. |
| 2134 | ADCk.15.gabor.3.0.1.mag. |
| 2135 | ADCk.15.gabor.3.0.2.mean. |
| 2136 | ADCk.15.gabor.3.0.2.var. |
| 2137 | ADCk.15.gabor.3.0.2.absmean. |
| 2138 | ADCk.15.gabor.3.0.2.mag. |
| 2139 | ADCk.15.gabor.3.0.3.mean. |
| 2140 | ADCk.15.gabor.3.0.3.var. |
| 2141 | ADCk.15.gabor.3.0.3.absmean. |
| 2142 | ADCk.15.gabor.3.0.3.mag. |
| 2143 | ADCk.15.gabor.3.0.4.mean. |
| 2144 | ADCk.15.gabor.3.0.4.var. |
| 2145 | ADCk.15.gabor.3.0.4.absmean. |
| 2146 | ADCk.15.gabor.3.0.4.mag. |
| 2147 | ADCk.15.gabor.3.0.5.mean. |
| 2148 | ADCk.15.gabor.3.0.5.var. |
| 2149 | ADCk.15.gabor.3.0.5.absmean. |
| 2150 | ADCk.15.gabor.3.0.5.mag. |
| 2151 | ADCk.3.haar.1.1.aav. |
| 2152 | ADCk.3.haar.1.1.std. |
| 2153 | ADCk.3.haar.1.2.aav. |
| 2154 | ADCk.3.haar.1.2.std. |
| 2155 | ADCk.3.haar.1.3.aav. |
| 2156 | ADCk.3.haar.1.3.std. |
| 2157 | ADCk.3.haar.2.1.aav. |
| 2158 | ADCk.3.haar.2.1.std. |
| 2159 | ADCk.3.haar.2.2.aav. |
| 2160 | ADCk.3.haar.2.2.std. |
| 2161 | ADCk.3.haar.2.3.aav. |
| 2162 | ADCk.3.haar.2.3.std. |
| 2163 | ADCk.3.haar.3.1.aav. |
| 2164 | ADCk.3.haar.3.1.std. |
| 2165 | ADCk.3.haar.3.2.aav. |
| 2166 | ADCk.3.haar.3.2.std. |
| 2167 | ADCk.3.haar.3.3.aav. |
| 2168 | ADCk.3.haar.3.3.std. |
| 2169 | ADCk.3.haar.4.1.aav. |
| 2170 | ADCk.3.haar.4.1.std. |
| 2171 | ADCk.3.haar.4.2.aav. |
| 2172 | ADCk.3.haar.4.2.std. |
| 2173 | ADCk.3.haar.4.3.aav. |
| 2174 | ADCk.3.haar.4.3.std. |
| 2175 | ADCk.5.haar.1.1.aav. |
| 2176 | ADCk.5.haar.1.1.std. |
| 2177 | ADCk.5.haar.1.2.aav. |
| 2178 | ADCk.5.haar.1.2.std. |
| 2179 | ADCk.5.haar.1.3.aav. |
| 2180 | ADCk.5.haar.1.3.std. |
| 2181 | ADCk.5.haar.2.1.aav. |
| 2182 | ADCk.5.haar.2.1.std. |
| 2183 | ADCk.5.haar.2.2.aav. |
| 2184 | ADCk.5.haar.2.2.std. |
| 2185 | ADCk.5.haar.2.3.aav. |
| 2186 | ADCk.5.haar.2.3.std. |
| 2187 | ADCk.5.haar.3.1.aav. |
| 2188 | ADCk.5.haar.3.1.std. |
| 2189 | ADCk.5.haar.3.2.aav. |
| 2190 | ADCk.5.haar.3.2.std. |
| 2191 | ADCk.5.haar.3.3.aav. |
| 2192 | ADCk.5.haar.3.3.std. |
| 2193 | ADCk.5.haar.4.1.aav. |
| 2194 | ADCk.5.haar.4.1.std. |
| 2195 | ADCk.5.haar.4.2.aav. |
| 2196 | ADCk.5.haar.4.2.std. |
| 2197 | ADCk.5.haar.4.3.aav. |
| 2198 | ADCk.5.haar.4.3.std. |
| 2199 | ADCk.7.haar.1.1.aav. |
| 2200 | ADCk.7.haar.1.1.std. |
| 2201 | ADCk.7.haar.1.2.aav. |
| 2202 | ADCk.7.haar.1.2.std. |
| 2203 | ADCk.7.haar.1.3.aav. |
| 2204 | ADCk.7.haar.1.3.std. |
| 2205 | ADCk.7.haar.2.1.aav. |
| 2206 | ADCk.7.haar.2.1.std. |
| 2207 | ADCk.7.haar.2.2.aav. |
| 2208 | ADCk.7.haar.2.2.std. |
| 2209 | ADCk.7.haar.2.3.aav. |
| 2210 | ADCk.7.haar.2.3.std. |
| 2211 | ADCk.7.haar.3.1.aav. |
| 2212 | ADCk.7.haar.3.1.std. |
| 2213 | ADCk.7.haar.3.2.aav. |
| 2214 | ADCk.7.haar.3.2.std. |
| 2215 | ADCk.7.haar.3.3.aav. |
| 2216 | ADCk.7.haar.3.3.std. |
| 2217 | ADCk.7.haar.4.1.aav. |
| 2218 | ADCk.7.haar.4.1.std. |
| 2219 | ADCk.7.haar.4.2.aav. |
| 2220 | ADCk.7.haar.4.2.std. |
| 2221 | ADCk.7.haar.4.3.aav. |
| 2222 | ADCk.7.haar.4.3.std. |
| 2223 | ADCk.9.haar.1.1.aav. |
| 2224 | ADCk.9.haar.1.1.std. |
| 2225 | ADCk.9.haar.1.2.aav. |
| 2226 | ADCk.9.haar.1.2.std. |
| 2227 | ADCk.9.haar.1.3.aav. |
| 2228 | ADCk.9.haar.1.3.std. |
| 2229 | ADCk.9.haar.2.1.aav. |
| 2230 | ADCk.9.haar.2.1.std. |
| 2231 | ADCk.9.haar.2.2.aav. |
| 2232 | ADCk.9.haar.2.2.std. |
| 2233 | ADCk.9.haar.2.3.aav. |
| 2234 | ADCk.9.haar.2.3.std. |
| 2235 | ADCk.9.haar.3.1.aav. |
| 2236 | ADCk.9.haar.3.1.std. |
| 2237 | ADCk.9.haar.3.2.aav. |
| 2238 | ADCk.9.haar.3.2.std. |
| 2239 | ADCk.9.haar.3.3.aav. |
| 2240 | ADCk.9.haar.3.3.std. |
| 2241 | ADCk.9.haar.4.1.aav. |
| 2242 | ADCk.9.haar.4.1.std. |
| 2243 | ADCk.9.haar.4.2.aav. |
| 2244 | ADCk.9.haar.4.2.std. |
| 2245 | ADCk.9.haar.4.3.aav. |
| 2246 | ADCk.9.haar.4.3.std. |
| 2247 | ADCk.11.haar.1.1.aav. |
| 2248 | ADCk.11.haar.1.1.std. |
| 2249 | ADCk.11.haar.1.2.aav. |
| 2250 | ADCk.11.haar.1.2.std. |
| 2251 | ADCk.11.haar.1.3.aav. |
| 2252 | ADCk.11.haar.1.3.std. |
| 2253 | ADCk.11.haar.2.1.aav. |
| 2254 | ADCk.11.haar.2.1.std. |
| 2255 | ADCk.11.haar.2.2.aav. |
| 2256 | ADCk.11.haar.2.2.std. |
| 2257 | ADCk.11.haar.2.3.aav. |
| 2258 | ADCk.11.haar.2.3.std. |
| 2259 | ADCk.11.haar.3.1.aav. |
| 2260 | ADCk.11.haar.3.1.std. |
| 2261 | ADCk.11.haar.3.2.aav. |
| 2262 | ADCk.11.haar.3.2.std. |
| 2263 | ADCk.11.haar.3.3.aav. |
| 2264 | ADCk.11.haar.3.3.std. |
| 2265 | ADCk.11.haar.4.1.aav. |
| 2266 | ADCk.11.haar.4.1.std. |
| 2267 | ADCk.11.haar.4.2.aav. |
| 2268 | ADCk.11.haar.4.2.std. |
| 2269 | ADCk.11.haar.4.3.aav. |
| 2270 | ADCk.11.haar.4.3.std. |
| 2271 | ADCk.13.haar.1.1.aav. |
| 2272 | ADCk.13.haar.1.1.std. |
| 2273 | ADCk.13.haar.1.2.aav. |
| 2274 | ADCk.13.haar.1.2.std. |
| 2275 | ADCk.13.haar.1.3.aav. |
| 2276 | ADCk.13.haar.1.3.std. |
| 2277 | ADCk.13.haar.2.1.aav. |
| 2278 | ADCk.13.haar.2.1.std. |
| 2279 | ADCk.13.haar.2.2.aav. |
| 2280 | ADCk.13.haar.2.2.std. |
| 2281 | ADCk.13.haar.2.3.aav. |
| 2282 | ADCk.13.haar.2.3.std. |
| 2283 | ADCk.13.haar.3.1.aav. |
| 2284 | ADCk.13.haar.3.1.std. |
| 2285 | ADCk.13.haar.3.2.aav. |
| 2286 | ADCk.13.haar.3.2.std. |
| 2287 | ADCk.13.haar.3.3.aav. |
| 2288 | ADCk.13.haar.3.3.std. |
| 2289 | ADCk.13.haar.4.1.aav. |
| 2290 | ADCk.13.haar.4.1.std. |
| 2291 | ADCk.13.haar.4.2.aav. |
| 2292 | ADCk.13.haar.4.2.std. |
| 2293 | ADCk.13.haar.4.3.aav. |
| 2294 | ADCk.13.haar.4.3.std. |
| 2295 | ADCk.15.haar.1.1.aav. |
| 2296 | ADCk.15.haar.1.1.std. |
| 2297 | ADCk.15.haar.1.2.aav. |
| 2298 | ADCk.15.haar.1.2.std. |
| 2299 | ADCk.15.haar.1.3.aav. |
| 2300 | ADCk.15.haar.1.3.std. |
| 2301 | ADCk.15.haar.2.1.aav. |
| 2302 | ADCk.15.haar.2.1.std. |
| 2303 | ADCk.15.haar.2.2.aav. |
| 2304 | ADCk.15.haar.2.2.std. |
| 2305 | ADCk.15.haar.2.3.aav. |
| 2306 | ADCk.15.haar.2.3.std. |
| 2307 | ADCk.15.haar.3.1.aav. |
| 2308 | ADCk.15.haar.3.1.std. |
| 2309 | ADCk.15.haar.3.2.aav. |
| 2310 | ADCk.15.haar.3.2.std. |
| 2311 | ADCk.15.haar.3.3.aav. |
| 2312 | ADCk.15.haar.3.3.std. |
| 2313 | ADCk.15.haar.4.1.aav. |
| 2314 | ADCk.15.haar.4.1.std. |
| 2315 | ADCk.15.haar.4.2.aav. |
| 2316 | ADCk.15.haar.4.2.std. |
| 2317 | ADCk.15.haar.4.3.aav. |
| 2318 | ADCk.15.haar.4.3.std. |
| 2319 | ADCk.3.hu.0. |
| 2320 | ADCk.3.hu.1. |
| 2321 | ADCk.3.hu.2. |
| 2322 | ADCk.3.hu.3. |
| 2323 | ADCk.3.hu.4. |
| 2324 | ADCk.3.hu.5. |
| 2325 | ADCk.3.hu.6. |
| 2326 | ADCk.5.hu.0. |
| 2327 | ADCk.5.hu.1. |
| 2328 | ADCk.5.hu.2. |
| 2329 | ADCk.5.hu.3. |
| 2330 | ADCk.5.hu.4. |
| 2331 | ADCk.5.hu.5. |
| 2332 | ADCk.5.hu.6. |
| 2333 | ADCk.7.hu.0. |
| 2334 | ADCk.7.hu.1. |
| 2335 | ADCk.7.hu.2. |
| 2336 | ADCk.7.hu.3. |
| 2337 | ADCk.7.hu.4. |
| 2338 | ADCk.7.hu.5. |
| 2339 | ADCk.7.hu.6. |
| 2340 | ADCk.9.hu.0. |
| 2341 | ADCk.9.hu.1. |
| 2342 | ADCk.9.hu.2. |
| 2343 | ADCk.9.hu.3. |
| 2344 | ADCk.9.hu.4. |
| 2345 | ADCk.9.hu.5. |
| 2346 | ADCk.9.hu.6. |
| 2347 | ADCk.11.hu.0. |
| 2348 | ADCk.11.hu.1. |
| 2349 | ADCk.11.hu.2. |
| 2350 | ADCk.11.hu.3. |
| 2351 | ADCk.11.hu.4. |
| 2352 | ADCk.11.hu.5. |
| 2353 | ADCk.11.hu.6. |
| 2354 | ADCk.13.hu.0. |
| 2355 | ADCk.13.hu.1. |
| 2356 | ADCk.13.hu.2. |
| 2357 | ADCk.13.hu.3. |
| 2358 | ADCk.13.hu.4. |
| 2359 | ADCk.13.hu.5. |
| 2360 | ADCk.13.hu.6. |
| 2361 | ADCk.15.hu.0. |
| 2362 | ADCk.15.hu.1. |
| 2363 | ADCk.15.hu.2. |
| 2364 | ADCk.15.hu.3. |
| 2365 | ADCk.15.hu.4. |
| 2366 | ADCk.15.hu.5. |
| 2367 | ADCk.15.hu.6. |
| 2368 | ADCk.3.zernike.0. |
| 2369 | ADCk.3.zernike.1. |
| 2370 | ADCk.3.zernike.2. |
| 2371 | ADCk.3.zernike.3. |
| 2372 | ADCk.3.zernike.4. |
| 2373 | ADCk.3.zernike.5. |
| 2374 | ADCk.3.zernike.6. |
| 2375 | ADCk.3.zernike.7. |
| 2376 | ADCk.3.zernike.8. |
| 2377 | ADCk.3.zernike.9. |
| 2378 | ADCk.3.zernike.10. |
| 2379 | ADCk.3.zernike.11. |
| 2380 | ADCk.3.zernike.12. |
| 2381 | ADCk.3.zernike.13. |
| 2382 | ADCk.3.zernike.14. |
| 2383 | ADCk.3.zernike.15. |
| 2384 | ADCk.3.zernike.16. |
| 2385 | ADCk.3.zernike.17. |
| 2386 | ADCk.3.zernike.18. |
| 2387 | ADCk.3.zernike.19. |
| 2388 | ADCk.3.zernike.20. |
| 2389 | ADCk.3.zernike.21. |
| 2390 | ADCk.3.zernike.22. |
| 2391 | ADCk.3.zernike.23. |
| 2392 | ADCk.3.zernike.24. |
| 2393 | ADCk.5.zernike.0. |
| 2394 | ADCk.5.zernike.1. |
| 2395 | ADCk.5.zernike.2. |
| 2396 | ADCk.5.zernike.3. |
| 2397 | ADCk.5.zernike.4. |
| 2398 | ADCk.5.zernike.5. |
| 2399 | ADCk.5.zernike.6. |
| 2400 | ADCk.5.zernike.7. |
| 2401 | ADCk.5.zernike.8. |
| 2402 | ADCk.5.zernike.9. |
| 2403 | ADCk.5.zernike.10. |
| 2404 | ADCk.5.zernike.11. |
| 2405 | ADCk.5.zernike.12. |
| 2406 | ADCk.5.zernike.13. |
| 2407 | ADCk.5.zernike.14. |
| 2408 | ADCk.5.zernike.15. |
| 2409 | ADCk.5.zernike.16. |
| 2410 | ADCk.5.zernike.17. |
| 2411 | ADCk.5.zernike.18. |
| 2412 | ADCk.5.zernike.19. |
| 2413 | ADCk.5.zernike.20. |
| 2414 | ADCk.5.zernike.21. |
| 2415 | ADCk.5.zernike.22. |
| 2416 | ADCk.5.zernike.23. |
| 2417 | ADCk.5.zernike.24. |
| 2418 | ADCk.7.zernike.0. |
| 2419 | ADCk.7.zernike.1. |
| 2420 | ADCk.7.zernike.2. |
| 2421 | ADCk.7.zernike.3. |
| 2422 | ADCk.7.zernike.4. |
| 2423 | ADCk.7.zernike.5. |
| 2424 | ADCk.7.zernike.6. |
| 2425 | ADCk.7.zernike.7. |
| 2426 | ADCk.7.zernike.8. |
| 2427 | ADCk.7.zernike.9. |
| 2428 | ADCk.7.zernike.10. |
| 2429 | ADCk.7.zernike.11. |
| 2430 | ADCk.7.zernike.12. |
| 2431 | ADCk.7.zernike.13. |
| 2432 | ADCk.7.zernike.14. |
| 2433 | ADCk.7.zernike.15. |
| 2434 | ADCk.7.zernike.16. |
| 2435 | ADCk.7.zernike.17. |
| 2436 | ADCk.7.zernike.18. |
| 2437 | ADCk.7.zernike.19. |
| 2438 | ADCk.7.zernike.20. |
| 2439 | ADCk.7.zernike.21. |
| 2440 | ADCk.7.zernike.22. |
| 2441 | ADCk.7.zernike.23. |
| 2442 | ADCk.7.zernike.24. |
| 2443 | ADCk.9.zernike.0. |
| 2444 | ADCk.9.zernike.1. |
| 2445 | ADCk.9.zernike.2. |
| 2446 | ADCk.9.zernike.3. |
| 2447 | ADCk.9.zernike.4. |
| 2448 | ADCk.9.zernike.5. |
| 2449 | ADCk.9.zernike.6. |
| 2450 | ADCk.9.zernike.7. |
| 2451 | ADCk.9.zernike.8. |
| 2452 | ADCk.9.zernike.9. |
| 2453 | ADCk.9.zernike.10. |
| 2454 | ADCk.9.zernike.11. |
| 2455 | ADCk.9.zernike.12. |
| 2456 | ADCk.9.zernike.13. |
| 2457 | ADCk.9.zernike.14. |
| 2458 | ADCk.9.zernike.15. |
| 2459 | ADCk.9.zernike.16. |
| 2460 | ADCk.9.zernike.17. |
| 2461 | ADCk.9.zernike.18. |
| 2462 | ADCk.9.zernike.19. |
| 2463 | ADCk.9.zernike.20. |
| 2464 | ADCk.9.zernike.21. |
| 2465 | ADCk.9.zernike.22. |
| 2466 | ADCk.9.zernike.23. |
| 2467 | ADCk.9.zernike.24. |
| 2468 | ADCk.11.zernike.0. |
| 2469 | ADCk.11.zernike.1. |
| 2470 | ADCk.11.zernike.2. |
| 2471 | ADCk.11.zernike.3. |
| 2472 | ADCk.11.zernike.4. |
| 2473 | ADCk.11.zernike.5. |
| 2474 | ADCk.11.zernike.6. |
| 2475 | ADCk.11.zernike.7. |
| 2476 | ADCk.11.zernike.8. |
| 2477 | ADCk.11.zernike.9. |
| 2478 | ADCk.11.zernike.10. |
| 2479 | ADCk.11.zernike.11. |
| 2480 | ADCk.11.zernike.12. |
| 2481 | ADCk.11.zernike.13. |
| 2482 | ADCk.11.zernike.14. |
| 2483 | ADCk.11.zernike.15. |
| 2484 | ADCk.11.zernike.16. |
| 2485 | ADCk.11.zernike.17. |
| 2486 | ADCk.11.zernike.18. |
| 2487 | ADCk.11.zernike.19. |
| 2488 | ADCk.11.zernike.20. |
| 2489 | ADCk.11.zernike.21. |
| 2490 | ADCk.11.zernike.22. |
| 2491 | ADCk.11.zernike.23. |
| 2492 | ADCk.11.zernike.24. |
| 2493 | ADCk.13.zernike.0. |
| 2494 | ADCk.13.zernike.1. |
| 2495 | ADCk.13.zernike.2. |
| 2496 | ADCk.13.zernike.3. |
| 2497 | ADCk.13.zernike.4. |
| 2498 | ADCk.13.zernike.5. |
| 2499 | ADCk.13.zernike.6. |
| 2500 | ADCk.13.zernike.7. |
| 2501 | ADCk.13.zernike.8. |
| 2502 | ADCk.13.zernike.9. |
| 2503 | ADCk.13.zernike.10. |
| 2504 | ADCk.13.zernike.11. |
| 2505 | ADCk.13.zernike.12. |
| 2506 | ADCk.13.zernike.13. |
| 2507 | ADCk.13.zernike.14. |
| 2508 | ADCk.13.zernike.15. |
| 2509 | ADCk.13.zernike.16. |
| 2510 | ADCk.13.zernike.17. |
| 2511 | ADCk.13.zernike.18. |
| 2512 | ADCk.13.zernike.19. |
| 2513 | ADCk.13.zernike.20. |
| 2514 | ADCk.13.zernike.21. |
| 2515 | ADCk.13.zernike.22. |
| 2516 | ADCk.13.zernike.23. |
| 2517 | ADCk.13.zernike.24. |
| 2518 | ADCk.15.zernike.0. |
| 2519 | ADCk.15.zernike.1. |
| 2520 | ADCk.15.zernike.2. |
| 2521 | ADCk.15.zernike.3. |
| 2522 | ADCk.15.zernike.4. |
| 2523 | ADCk.15.zernike.5. |
| 2524 | ADCk.15.zernike.6. |
| 2525 | ADCk.15.zernike.7. |
| 2526 | ADCk.15.zernike.8. |
| 2527 | ADCk.15.zernike.9. |
| 2528 | ADCk.15.zernike.10. |
| 2529 | ADCk.15.zernike.11. |
| 2530 | ADCk.15.zernike.12. |
| 2531 | ADCk.15.zernike.13. |
| 2532 | ADCk.15.zernike.14. |
| 2533 | ADCk.15.zernike.15. |
| 2534 | ADCk.15.zernike.16. |
| 2535 | ADCk.15.zernike.17. |
| 2536 | ADCk.15.zernike.18. |
| 2537 | ADCk.15.zernike.19. |
| 2538 | ADCk.15.zernike.20. |
| 2539 | ADCk.15.zernike.21. |
| 2540 | ADCk.15.zernike.22. |
| 2541 | ADCk.15.zernike.23. |
| 2542 | ADCk.15.zernike.24. |
| 2543 | ADCk.3.sobel |
| 2544 | ADCk.3.sobel_mask |
| 2545 | ADCk.all.stats.p000. |
| 2546 | ADCk.all.stats.p010. |
| 2547 | ADCk.all.stats.p020. |
| 2548 | ADCk.all.stats.p025. |
| 2549 | ADCk.all.stats.p030. |
| 2550 | ADCk.all.stats.p040. |
| 2551 | ADCk.all.stats.p050. |
| 2552 | ADCk.all.stats.p060. |
| 2553 | ADCk.all.stats.p070. |
| 2554 | ADCk.all.stats.p075. |
| 2555 | ADCk.all.stats.p080. |
| 2556 | ADCk.all.stats.p090. |
| 2557 | ADCk.all.stats.p100. |
| 2558 | ADCk.all.stats.range. |
| 2559 | ADCk.all.stats.mean. |
| 2560 | ADCk.all.stats.stddev. |
| 2561 | ADCk.all.stats.kurtosis. |
| 2562 | ADCk.all.stats.skewness. |
| 2563 | K.3.glcm.contrast.1.mean. |
| 2564 | K.3.glcm.contrast.1.range. |
| 2565 | K.3.glcm.contrast.2.mean. |
| 2566 | K.3.glcm.contrast.2.range. |
| 2567 | K.3.glcm.contrast.3.mean. |
| 2568 | K.3.glcm.contrast.3.range. |
| 2569 | K.3.glcm.dissimilarity.1.mean. |
| 2570 | K.3.glcm.dissimilarity.1.range. |
| 2571 | K.3.glcm.dissimilarity.2.mean. |
| 2572 | K.3.glcm.dissimilarity.2.range. |
| 2573 | K.3.glcm.dissimilarity.3.mean. |
| 2574 | K.3.glcm.dissimilarity.3.range. |
| 2575 | K.3.glcm.homogeneity.1.mean. |
| 2576 | K.3.glcm.homogeneity.1.range. |
| 2577 | K.3.glcm.homogeneity.2.mean. |
| 2578 | K.3.glcm.homogeneity.2.range. |
| 2579 | K.3.glcm.homogeneity.3.mean. |
| 2580 | K.3.glcm.homogeneity.3.range. |
| 2581 | K.3.glcm.energy.1.mean. |
| 2582 | K.3.glcm.energy.1.range. |
| 2583 | K.3.glcm.energy.2.mean. |
| 2584 | K.3.glcm.energy.2.range. |
| 2585 | K.3.glcm.energy.3.mean. |
| 2586 | K.3.glcm.energy.3.range. |
| 2587 | K.3.glcm.correlation.1.mean. |
| 2588 | K.3.glcm.correlation.1.range. |
| 2589 | K.3.glcm.correlation.2.mean. |
| 2590 | K.3.glcm.correlation.2.range. |
| 2591 | K.3.glcm.correlation.3.mean. |
| 2592 | K.3.glcm.correlation.3.range. |
| 2593 | K.3.glcm.ASM.1.mean. |
| 2594 | K.3.glcm.ASM.1.range. |
| 2595 | K.3.glcm.ASM.2.mean. |
| 2596 | K.3.glcm.ASM.2.range. |
| 2597 | K.3.glcm.ASM.3.mean. |
| 2598 | K.3.glcm.ASM.3.range. |
| 2599 | K.5.glcm.contrast.1.mean. |
| 2600 | K.5.glcm.contrast.1.range. |
| 2601 | K.5.glcm.contrast.2.mean. |
| 2602 | K.5.glcm.contrast.2.range. |
| 2603 | K.5.glcm.contrast.3.mean. |
| 2604 | K.5.glcm.contrast.3.range. |
| 2605 | K.5.glcm.contrast.4.mean. |
| 2606 | K.5.glcm.contrast.4.range. |
| 2607 | K.5.glcm.dissimilarity.1.mean. |
| 2608 | K.5.glcm.dissimilarity.1.range. |
| 2609 | K.5.glcm.dissimilarity.2.mean. |
| 2610 | K.5.glcm.dissimilarity.2.range. |
| 2611 | K.5.glcm.dissimilarity.3.mean. |
| 2612 | K.5.glcm.dissimilarity.3.range. |
| 2613 | K.5.glcm.dissimilarity.4.mean. |
| 2614 | K.5.glcm.dissimilarity.4.range. |
| 2615 | K.5.glcm.homogeneity.1.mean. |
| 2616 | K.5.glcm.homogeneity.1.range. |
| 2617 | K.5.glcm.homogeneity.2.mean. |
| 2618 | K.5.glcm.homogeneity.2.range. |
| 2619 | K.5.glcm.homogeneity.3.mean. |
| 2620 | K.5.glcm.homogeneity.3.range. |
| 2621 | K.5.glcm.homogeneity.4.mean. |
| 2622 | K.5.glcm.homogeneity.4.range. |
| 2623 | K.5.glcm.energy.1.mean. |
| 2624 | K.5.glcm.energy.1.range. |
| 2625 | K.5.glcm.energy.2.mean. |
| 2626 | K.5.glcm.energy.2.range. |
| 2627 | K.5.glcm.energy.3.mean. |
| 2628 | K.5.glcm.energy.3.range. |
| 2629 | K.5.glcm.energy.4.mean. |
| 2630 | K.5.glcm.energy.4.range. |
| 2631 | K.5.glcm.correlation.1.mean. |
| 2632 | K.5.glcm.correlation.1.range. |
| 2633 | K.5.glcm.correlation.2.mean. |
| 2634 | K.5.glcm.correlation.2.range. |
| 2635 | K.5.glcm.correlation.3.mean. |
| 2636 | K.5.glcm.correlation.3.range. |
| 2637 | K.5.glcm.correlation.4.mean. |
| 2638 | K.5.glcm.correlation.4.range. |
| 2639 | K.5.glcm.ASM.1.mean. |
| 2640 | K.5.glcm.ASM.1.range. |
| 2641 | K.5.glcm.ASM.2.mean. |
| 2642 | K.5.glcm.ASM.2.range. |
| 2643 | K.5.glcm.ASM.3.mean. |
| 2644 | K.5.glcm.ASM.3.range. |
| 2645 | K.5.glcm.ASM.4.mean. |
| 2646 | K.5.glcm.ASM.4.range. |
| 2647 | K.7.glcm.contrast.1.mean. |
| 2648 | K.7.glcm.contrast.1.range. |
| 2649 | K.7.glcm.contrast.2.mean. |
| 2650 | K.7.glcm.contrast.2.range. |
| 2651 | K.7.glcm.contrast.3.mean. |
| 2652 | K.7.glcm.contrast.3.range. |
| 2653 | K.7.glcm.contrast.4.mean. |
| 2654 | K.7.glcm.contrast.4.range. |
| 2655 | K.7.glcm.dissimilarity.1.mean. |
| 2656 | K.7.glcm.dissimilarity.1.range. |
| 2657 | K.7.glcm.dissimilarity.2.mean. |
| 2658 | K.7.glcm.dissimilarity.2.range. |
| 2659 | K.7.glcm.dissimilarity.3.mean. |
| 2660 | K.7.glcm.dissimilarity.3.range. |
| 2661 | K.7.glcm.dissimilarity.4.mean. |
| 2662 | K.7.glcm.dissimilarity.4.range. |
| 2663 | K.7.glcm.homogeneity.1.mean. |
| 2664 | K.7.glcm.homogeneity.1.range. |
| 2665 | K.7.glcm.homogeneity.2.mean. |
| 2666 | K.7.glcm.homogeneity.2.range. |
| 2667 | K.7.glcm.homogeneity.3.mean. |
| 2668 | K.7.glcm.homogeneity.3.range. |
| 2669 | K.7.glcm.homogeneity.4.mean. |
| 2670 | K.7.glcm.homogeneity.4.range. |
| 2671 | K.7.glcm.energy.1.mean. |
| 2672 | K.7.glcm.energy.1.range. |
| 2673 | K.7.glcm.energy.2.mean. |
| 2674 | K.7.glcm.energy.2.range. |
| 2675 | K.7.glcm.energy.3.mean. |
| 2676 | K.7.glcm.energy.3.range. |
| 2677 | K.7.glcm.energy.4.mean. |
| 2678 | K.7.glcm.energy.4.range. |
| 2679 | K.7.glcm.correlation.1.mean. |
| 2680 | K.7.glcm.correlation.1.range. |
| 2681 | K.7.glcm.correlation.2.mean. |
| 2682 | K.7.glcm.correlation.2.range. |
| 2683 | K.7.glcm.correlation.3.mean. |
| 2684 | K.7.glcm.correlation.3.range. |
| 2685 | K.7.glcm.correlation.4.mean. |
| 2686 | K.7.glcm.correlation.4.range. |
| 2687 | K.7.glcm.ASM.1.mean. |
| 2688 | K.7.glcm.ASM.1.range. |
| 2689 | K.7.glcm.ASM.2.mean. |
| 2690 | K.7.glcm.ASM.2.range. |
| 2691 | K.7.glcm.ASM.3.mean. |
| 2692 | K.7.glcm.ASM.3.range. |
| 2693 | K.7.glcm.ASM.4.mean. |
| 2694 | K.7.glcm.ASM.4.range. |
| 2695 | K.9.glcm.contrast.1.mean. |
| 2696 | K.9.glcm.contrast.1.range. |
| 2697 | K.9.glcm.contrast.2.mean. |
| 2698 | K.9.glcm.contrast.2.range. |
| 2699 | K.9.glcm.contrast.3.mean. |
| 2700 | K.9.glcm.contrast.3.range. |
| 2701 | K.9.glcm.contrast.4.mean. |
| 2702 | K.9.glcm.contrast.4.range. |
| 2703 | K.9.glcm.dissimilarity.1.mean. |
| 2704 | K.9.glcm.dissimilarity.1.range. |
| 2705 | K.9.glcm.dissimilarity.2.mean. |
| 2706 | K.9.glcm.dissimilarity.2.range. |
| 2707 | K.9.glcm.dissimilarity.3.mean. |
| 2708 | K.9.glcm.dissimilarity.3.range. |
| 2709 | K.9.glcm.dissimilarity.4.mean. |
| 2710 | K.9.glcm.dissimilarity.4.range. |
| 2711 | K.9.glcm.homogeneity.1.mean. |
| 2712 | K.9.glcm.homogeneity.1.range. |
| 2713 | K.9.glcm.homogeneity.2.mean. |
| 2714 | K.9.glcm.homogeneity.2.range. |
| 2715 | K.9.glcm.homogeneity.3.mean. |
| 2716 | K.9.glcm.homogeneity.3.range. |
| 2717 | K.9.glcm.homogeneity.4.mean. |
| 2718 | K.9.glcm.homogeneity.4.range. |
| 2719 | K.9.glcm.energy.1.mean. |
| 2720 | K.9.glcm.energy.1.range. |
| 2721 | K.9.glcm.energy.2.mean. |
| 2722 | K.9.glcm.energy.2.range. |
| 2723 | K.9.glcm.energy.3.mean. |
| 2724 | K.9.glcm.energy.3.range. |
| 2725 | K.9.glcm.energy.4.mean. |
| 2726 | K.9.glcm.energy.4.range. |
| 2727 | K.9.glcm.correlation.1.mean. |
| 2728 | K.9.glcm.correlation.1.range. |
| 2729 | K.9.glcm.correlation.2.mean. |
| 2730 | K.9.glcm.correlation.2.range. |
| 2731 | K.9.glcm.correlation.3.mean. |
| 2732 | K.9.glcm.correlation.3.range. |
| 2733 | K.9.glcm.correlation.4.mean. |
| 2734 | K.9.glcm.correlation.4.range. |
| 2735 | K.9.glcm.ASM.1.mean. |
| 2736 | K.9.glcm.ASM.1.range. |
| 2737 | K.9.glcm.ASM.2.mean. |
| 2738 | K.9.glcm.ASM.2.range. |
| 2739 | K.9.glcm.ASM.3.mean. |
| 2740 | K.9.glcm.ASM.3.range. |
| 2741 | K.9.glcm.ASM.4.mean. |
| 2742 | K.9.glcm.ASM.4.range. |
| 2743 | K.11.glcm.contrast.1.mean. |
| 2744 | K.11.glcm.contrast.1.range. |
| 2745 | K.11.glcm.contrast.2.mean. |
| 2746 | K.11.glcm.contrast.2.range. |
| 2747 | K.11.glcm.contrast.3.mean. |
| 2748 | K.11.glcm.contrast.3.range. |
| 2749 | K.11.glcm.contrast.4.mean. |
| 2750 | K.11.glcm.contrast.4.range. |
| 2751 | K.11.glcm.dissimilarity.1.mean. |
| 2752 | K.11.glcm.dissimilarity.1.range. |
| 2753 | K.11.glcm.dissimilarity.2.mean. |
| 2754 | K.11.glcm.dissimilarity.2.range. |
| 2755 | K.11.glcm.dissimilarity.3.mean. |
| 2756 | K.11.glcm.dissimilarity.3.range. |
| 2757 | K.11.glcm.dissimilarity.4.mean. |
| 2758 | K.11.glcm.dissimilarity.4.range. |
| 2759 | K.11.glcm.homogeneity.1.mean. |
| 2760 | K.11.glcm.homogeneity.1.range. |
| 2761 | K.11.glcm.homogeneity.2.mean. |
| 2762 | K.11.glcm.homogeneity.2.range. |
| 2763 | K.11.glcm.homogeneity.3.mean. |
| 2764 | K.11.glcm.homogeneity.3.range. |
| 2765 | K.11.glcm.homogeneity.4.mean. |
| 2766 | K.11.glcm.homogeneity.4.range. |
| 2767 | K.11.glcm.energy.1.mean. |
| 2768 | K.11.glcm.energy.1.range. |
| 2769 | K.11.glcm.energy.2.mean. |
| 2770 | K.11.glcm.energy.2.range. |
| 2771 | K.11.glcm.energy.3.mean. |
| 2772 | K.11.glcm.energy.3.range. |
| 2773 | K.11.glcm.energy.4.mean. |
| 2774 | K.11.glcm.energy.4.range. |
| 2775 | K.11.glcm.correlation.1.mean. |
| 2776 | K.11.glcm.correlation.1.range. |
| 2777 | K.11.glcm.correlation.2.mean. |
| 2778 | K.11.glcm.correlation.2.range. |
| 2779 | K.11.glcm.correlation.3.mean. |
| 2780 | K.11.glcm.correlation.3.range. |
| 2781 | K.11.glcm.correlation.4.mean. |
| 2782 | K.11.glcm.correlation.4.range. |
| 2783 | K.11.glcm.ASM.1.mean. |
| 2784 | K.11.glcm.ASM.1.range. |
| 2785 | K.11.glcm.ASM.2.mean. |
| 2786 | K.11.glcm.ASM.2.range. |
| 2787 | K.11.glcm.ASM.3.mean. |
| 2788 | K.11.glcm.ASM.3.range. |
| 2789 | K.11.glcm.ASM.4.mean. |
| 2790 | K.11.glcm.ASM.4.range. |
| 2791 | K.13.glcm.contrast.1.mean. |
| 2792 | K.13.glcm.contrast.1.range. |
| 2793 | K.13.glcm.contrast.2.mean. |
| 2794 | K.13.glcm.contrast.2.range. |
| 2795 | K.13.glcm.contrast.3.mean. |
| 2796 | K.13.glcm.contrast.3.range. |
| 2797 | K.13.glcm.contrast.4.mean. |
| 2798 | K.13.glcm.contrast.4.range. |
| 2799 | K.13.glcm.dissimilarity.1.mean. |
| 2800 | K.13.glcm.dissimilarity.1.range. |
| 2801 | K.13.glcm.dissimilarity.2.mean. |
| 2802 | K.13.glcm.dissimilarity.2.range. |
| 2803 | K.13.glcm.dissimilarity.3.mean. |
| 2804 | K.13.glcm.dissimilarity.3.range. |
| 2805 | K.13.glcm.dissimilarity.4.mean. |
| 2806 | K.13.glcm.dissimilarity.4.range. |
| 2807 | K.13.glcm.homogeneity.1.mean. |
| 2808 | K.13.glcm.homogeneity.1.range. |
| 2809 | K.13.glcm.homogeneity.2.mean. |
| 2810 | K.13.glcm.homogeneity.2.range. |
| 2811 | K.13.glcm.homogeneity.3.mean. |
| 2812 | K.13.glcm.homogeneity.3.range. |
| 2813 | K.13.glcm.homogeneity.4.mean. |
| 2814 | K.13.glcm.homogeneity.4.range. |
| 2815 | K.13.glcm.energy.1.mean. |
| 2816 | K.13.glcm.energy.1.range. |
| 2817 | K.13.glcm.energy.2.mean. |
| 2818 | K.13.glcm.energy.2.range. |
| 2819 | K.13.glcm.energy.3.mean. |
| 2820 | K.13.glcm.energy.3.range. |
| 2821 | K.13.glcm.energy.4.mean. |
| 2822 | K.13.glcm.energy.4.range. |
| 2823 | K.13.glcm.correlation.1.mean. |
| 2824 | K.13.glcm.correlation.1.range. |
| 2825 | K.13.glcm.correlation.2.mean. |
| 2826 | K.13.glcm.correlation.2.range. |
| 2827 | K.13.glcm.correlation.3.mean. |
| 2828 | K.13.glcm.correlation.3.range. |
| 2829 | K.13.glcm.correlation.4.mean. |
| 2830 | K.13.glcm.correlation.4.range. |
| 2831 | K.13.glcm.ASM.1.mean. |
| 2832 | K.13.glcm.ASM.1.range. |
| 2833 | K.13.glcm.ASM.2.mean. |
| 2834 | K.13.glcm.ASM.2.range. |
| 2835 | K.13.glcm.ASM.3.mean. |
| 2836 | K.13.glcm.ASM.3.range. |
| 2837 | K.13.glcm.ASM.4.mean. |
| 2838 | K.13.glcm.ASM.4.range. |
| 2839 | K.15.glcm.contrast.1.mean. |
| 2840 | K.15.glcm.contrast.1.range. |
| 2841 | K.15.glcm.contrast.2.mean. |
| 2842 | K.15.glcm.contrast.2.range. |
| 2843 | K.15.glcm.contrast.3.mean. |
| 2844 | K.15.glcm.contrast.3.range. |
| 2845 | K.15.glcm.contrast.4.mean. |
| 2846 | K.15.glcm.contrast.4.range. |
| 2847 | K.15.glcm.dissimilarity.1.mean. |
| 2848 | K.15.glcm.dissimilarity.1.range. |
| 2849 | K.15.glcm.dissimilarity.2.mean. |
| 2850 | K.15.glcm.dissimilarity.2.range. |
| 2851 | K.15.glcm.dissimilarity.3.mean. |
| 2852 | K.15.glcm.dissimilarity.3.range. |
| 2853 | K.15.glcm.dissimilarity.4.mean. |
| 2854 | K.15.glcm.dissimilarity.4.range. |
| 2855 | K.15.glcm.homogeneity.1.mean. |
| 2856 | K.15.glcm.homogeneity.1.range. |
| 2857 | K.15.glcm.homogeneity.2.mean. |
| 2858 | K.15.glcm.homogeneity.2.range. |
| 2859 | K.15.glcm.homogeneity.3.mean. |
| 2860 | K.15.glcm.homogeneity.3.range. |
| 2861 | K.15.glcm.homogeneity.4.mean. |
| 2862 | K.15.glcm.homogeneity.4.range. |
| 2863 | K.15.glcm.energy.1.mean. |
| 2864 | K.15.glcm.energy.1.range. |
| 2865 | K.15.glcm.energy.2.mean. |
| 2866 | K.15.glcm.energy.2.range. |
| 2867 | K.15.glcm.energy.3.mean. |
| 2868 | K.15.glcm.energy.3.range. |
| 2869 | K.15.glcm.energy.4.mean. |
| 2870 | K.15.glcm.energy.4.range. |
| 2871 | K.15.glcm.correlation.1.mean. |
| 2872 | K.15.glcm.correlation.1.range. |
| 2873 | K.15.glcm.correlation.2.mean. |
| 2874 | K.15.glcm.correlation.2.range. |
| 2875 | K.15.glcm.correlation.3.mean. |
| 2876 | K.15.glcm.correlation.3.range. |
| 2877 | K.15.glcm.correlation.4.mean. |
| 2878 | K.15.glcm.correlation.4.range. |
| 2879 | K.15.glcm.ASM.1.mean. |
| 2880 | K.15.glcm.ASM.1.range. |
| 2881 | K.15.glcm.ASM.2.mean. |
| 2882 | K.15.glcm.ASM.2.range. |
| 2883 | K.15.glcm.ASM.3.mean. |
| 2884 | K.15.glcm.ASM.3.range. |
| 2885 | K.15.glcm.ASM.4.mean. |
| 2886 | K.15.glcm.ASM.4.range. |
| 2887 | K.mbb.glcm.contrast.1.mean. |
| 2888 | K.mbb.glcm.contrast.1.range. |
| 2889 | K.mbb.glcm.contrast.2.mean. |
| 2890 | K.mbb.glcm.contrast.2.range. |
| 2891 | K.mbb.glcm.contrast.3.mean. |
| 2892 | K.mbb.glcm.contrast.3.range. |
| 2893 | K.mbb.glcm.contrast.4.mean. |
| 2894 | K.mbb.glcm.contrast.4.range. |
| 2895 | K.mbb.glcm.dissimilarity.1.mean. |
| 2896 | K.mbb.glcm.dissimilarity.1.range. |
| 2897 | K.mbb.glcm.dissimilarity.2.mean. |
| 2898 | K.mbb.glcm.dissimilarity.2.range. |
| 2899 | K.mbb.glcm.dissimilarity.3.mean. |
| 2900 | K.mbb.glcm.dissimilarity.3.range. |
| 2901 | K.mbb.glcm.dissimilarity.4.mean. |
| 2902 | K.mbb.glcm.dissimilarity.4.range. |
| 2903 | K.mbb.glcm.homogeneity.1.mean. |
| 2904 | K.mbb.glcm.homogeneity.1.range. |
| 2905 | K.mbb.glcm.homogeneity.2.mean. |
| 2906 | K.mbb.glcm.homogeneity.2.range. |
| 2907 | K.mbb.glcm.homogeneity.3.mean. |
| 2908 | K.mbb.glcm.homogeneity.3.range. |
| 2909 | K.mbb.glcm.homogeneity.4.mean. |
| 2910 | K.mbb.glcm.homogeneity.4.range. |
| 2911 | K.mbb.glcm.energy.1.mean. |
| 2912 | K.mbb.glcm.energy.1.range. |
| 2913 | K.mbb.glcm.energy.2.mean. |
| 2914 | K.mbb.glcm.energy.2.range. |
| 2915 | K.mbb.glcm.energy.3.mean. |
| 2916 | K.mbb.glcm.energy.3.range. |
| 2917 | K.mbb.glcm.energy.4.mean. |
| 2918 | K.mbb.glcm.energy.4.range. |
| 2919 | K.mbb.glcm.correlation.1.mean. |
| 2920 | K.mbb.glcm.correlation.1.range. |
| 2921 | K.mbb.glcm.correlation.2.mean. |
| 2922 | K.mbb.glcm.correlation.2.range. |
| 2923 | K.mbb.glcm.correlation.3.mean. |
| 2924 | K.mbb.glcm.correlation.3.range. |
| 2925 | K.mbb.glcm.correlation.4.mean. |
| 2926 | K.mbb.glcm.correlation.4.range. |
| 2927 | K.mbb.glcm.ASM.1.mean. |
| 2928 | K.mbb.glcm.ASM.1.range. |
| 2929 | K.mbb.glcm.ASM.2.mean. |
| 2930 | K.mbb.glcm.ASM.2.range. |
| 2931 | K.mbb.glcm.ASM.3.mean. |
| 2932 | K.mbb.glcm.ASM.3.range. |
| 2933 | K.mbb.glcm.ASM.4.mean. |
| 2934 | K.mbb.glcm.ASM.4.range. |
| 2935 | K.3.lbp.1.0. |
| 2936 | K.3.lbp.1.1. |
| 2937 | K.3.lbp.1.2. |
| 2938 | K.3.lbp.1.3. |
| 2939 | K.3.lbp.1.4. |
| 2940 | K.3.lbp.1.5. |
| 2941 | K.3.lbp.1.6. |
| 2942 | K.3.lbp.1.7. |
| 2943 | K.3.lbp.1.8. |
| 2944 | K.3.lbp.1.9. |
| 2945 | K.5.lbp.2.0. |
| 2946 | K.5.lbp.2.1. |
| 2947 | K.5.lbp.2.2. |
| 2948 | K.5.lbp.2.3. |
| 2949 | K.5.lbp.2.4. |
| 2950 | K.5.lbp.2.5. |
| 2951 | K.5.lbp.2.6. |
| 2952 | K.5.lbp.2.7. |
| 2953 | K.5.lbp.2.8. |
| 2954 | K.5.lbp.2.9. |
| 2955 | K.7.lbp.3.0. |
| 2956 | K.7.lbp.3.1. |
| 2957 | K.7.lbp.3.2. |
| 2958 | K.7.lbp.3.3. |
| 2959 | K.7.lbp.3.4. |
| 2960 | K.7.lbp.3.5. |
| 2961 | K.7.lbp.3.6. |
| 2962 | K.7.lbp.3.7. |
| 2963 | K.7.lbp.3.8. |
| 2964 | K.7.lbp.3.9. |
| 2965 | K.9.lbp.4.0. |
| 2966 | K.9.lbp.4.1. |
| 2967 | K.9.lbp.4.2. |
| 2968 | K.9.lbp.4.3. |
| 2969 | K.9.lbp.4.4. |
| 2970 | K.9.lbp.4.5. |
| 2971 | K.9.lbp.4.6. |
| 2972 | K.9.lbp.4.7. |
| 2973 | K.9.lbp.4.8. |
| 2974 | K.9.lbp.4.9. |
| 2975 | K.11.lbp.5.0. |
| 2976 | K.11.lbp.5.1. |
| 2977 | K.11.lbp.5.2. |
| 2978 | K.11.lbp.5.3. |
| 2979 | K.11.lbp.5.4. |
| 2980 | K.11.lbp.5.5. |
| 2981 | K.11.lbp.5.6. |
| 2982 | K.11.lbp.5.7. |
| 2983 | K.11.lbp.5.8. |
| 2984 | K.11.lbp.5.9. |
| 2985 | K.13.lbp.6.0. |
| 2986 | K.13.lbp.6.1. |
| 2987 | K.13.lbp.6.2. |
| 2988 | K.13.lbp.6.3. |
| 2989 | K.13.lbp.6.4. |
| 2990 | K.13.lbp.6.5. |
| 2991 | K.13.lbp.6.6. |
| 2992 | K.13.lbp.6.7. |
| 2993 | K.13.lbp.6.8. |
| 2994 | K.13.lbp.6.9. |
| 2995 | K.15.lbp.7.0. |
| 2996 | K.15.lbp.7.1. |
| 2997 | K.15.lbp.7.2. |
| 2998 | K.15.lbp.7.3. |
| 2999 | K.15.lbp.7.4. |
| 3000 | K.15.lbp.7.5. |
| 3001 | K.15.lbp.7.6. |
| 3002 | K.15.lbp.7.7. |
| 3003 | K.15.lbp.7.8. |
| 3004 | K.15.lbp.7.9. |
| 3005 | K.3.hog |
| 3006 | K.5.hog |
| 3007 | K.7.hog |
| 3008 | K.9.hog |
| 3009 | K.11.hog |
| 3010 | K.13.hog |
| 3011 | K.15.hog |
| 3012 | K.3.gabor.1.0.1.mean. |
| 3013 | K.3.gabor.1.0.1.var. |
| 3014 | K.3.gabor.1.0.1.absmean. |
| 3015 | K.3.gabor.1.0.1.mag. |
| 3016 | K.3.gabor.1.0.2.mean. |
| 3017 | K.3.gabor.1.0.2.var. |
| 3018 | K.3.gabor.1.0.2.absmean. |
| 3019 | K.3.gabor.1.0.2.mag. |
| 3020 | K.3.gabor.1.0.3.mean. |
| 3021 | K.3.gabor.1.0.3.var. |
| 3022 | K.3.gabor.1.0.3.absmean. |
| 3023 | K.3.gabor.1.0.3.mag. |
| 3024 | K.3.gabor.1.0.4.mean. |
| 3025 | K.3.gabor.1.0.4.var. |
| 3026 | K.3.gabor.1.0.4.absmean. |
| 3027 | K.3.gabor.1.0.4.mag. |
| 3028 | K.3.gabor.1.0.5.mean. |
| 3029 | K.3.gabor.1.0.5.var. |
| 3030 | K.3.gabor.1.0.5.absmean. |
| 3031 | K.3.gabor.1.0.5.mag. |
| 3032 | K.3.gabor.2.0.1.mean. |
| 3033 | K.3.gabor.2.0.1.var. |
| 3034 | K.3.gabor.2.0.1.absmean. |
| 3035 | K.3.gabor.2.0.1.mag. |
| 3036 | K.3.gabor.2.0.2.mean. |
| 3037 | K.3.gabor.2.0.2.var. |
| 3038 | K.3.gabor.2.0.2.absmean. |
| 3039 | K.3.gabor.2.0.2.mag. |
| 3040 | K.3.gabor.2.0.3.mean. |
| 3041 | K.3.gabor.2.0.3.var. |
| 3042 | K.3.gabor.2.0.3.absmean. |
| 3043 | K.3.gabor.2.0.3.mag. |
| 3044 | K.3.gabor.2.0.4.mean. |
| 3045 | K.3.gabor.2.0.4.var. |
| 3046 | K.3.gabor.2.0.4.absmean. |
| 3047 | K.3.gabor.2.0.4.mag. |
| 3048 | K.3.gabor.2.0.5.mean. |
| 3049 | K.3.gabor.2.0.5.var. |
| 3050 | K.3.gabor.2.0.5.absmean. |
| 3051 | K.3.gabor.2.0.5.mag. |
| 3052 | K.3.gabor.3.0.1.mean. |
| 3053 | K.3.gabor.3.0.1.var. |
| 3054 | K.3.gabor.3.0.1.absmean. |
| 3055 | K.3.gabor.3.0.1.mag. |
| 3056 | K.3.gabor.3.0.2.mean. |
| 3057 | K.3.gabor.3.0.2.var. |
| 3058 | K.3.gabor.3.0.2.absmean. |
| 3059 | K.3.gabor.3.0.2.mag. |
| 3060 | K.3.gabor.3.0.3.mean. |
| 3061 | K.3.gabor.3.0.3.var. |
| 3062 | K.3.gabor.3.0.3.absmean. |
| 3063 | K.3.gabor.3.0.3.mag. |
| 3064 | K.3.gabor.3.0.4.mean. |
| 3065 | K.3.gabor.3.0.4.var. |
| 3066 | K.3.gabor.3.0.4.absmean. |
| 3067 | K.3.gabor.3.0.4.mag. |
| 3068 | K.3.gabor.3.0.5.mean. |
| 3069 | K.3.gabor.3.0.5.var. |
| 3070 | K.3.gabor.3.0.5.absmean. |
| 3071 | K.3.gabor.3.0.5.mag. |
| 3072 | K.5.gabor.1.0.1.mean. |
| 3073 | K.5.gabor.1.0.1.var. |
| 3074 | K.5.gabor.1.0.1.absmean. |
| 3075 | K.5.gabor.1.0.1.mag. |
| 3076 | K.5.gabor.1.0.2.mean. |
| 3077 | K.5.gabor.1.0.2.var. |
| 3078 | K.5.gabor.1.0.2.absmean. |
| 3079 | K.5.gabor.1.0.2.mag. |
| 3080 | K.5.gabor.1.0.3.mean. |
| 3081 | K.5.gabor.1.0.3.var. |
| 3082 | K.5.gabor.1.0.3.absmean. |
| 3083 | K.5.gabor.1.0.3.mag. |
| 3084 | K.5.gabor.1.0.4.mean. |
| 3085 | K.5.gabor.1.0.4.var. |
| 3086 | K.5.gabor.1.0.4.absmean. |
| 3087 | K.5.gabor.1.0.4.mag. |
| 3088 | K.5.gabor.1.0.5.mean. |
| 3089 | K.5.gabor.1.0.5.var. |
| 3090 | K.5.gabor.1.0.5.absmean. |
| 3091 | K.5.gabor.1.0.5.mag. |
| 3092 | K.5.gabor.2.0.1.mean. |
| 3093 | K.5.gabor.2.0.1.var. |
| 3094 | K.5.gabor.2.0.1.absmean. |
| 3095 | K.5.gabor.2.0.1.mag. |
| 3096 | K.5.gabor.2.0.2.mean. |
| 3097 | K.5.gabor.2.0.2.var. |
| 3098 | K.5.gabor.2.0.2.absmean. |
| 3099 | K.5.gabor.2.0.2.mag. |
| 3100 | K.5.gabor.2.0.3.mean. |
| 3101 | K.5.gabor.2.0.3.var. |
| 3102 | K.5.gabor.2.0.3.absmean. |
| 3103 | K.5.gabor.2.0.3.mag. |
| 3104 | K.5.gabor.2.0.4.mean. |
| 3105 | K.5.gabor.2.0.4.var. |
| 3106 | K.5.gabor.2.0.4.absmean. |
| 3107 | K.5.gabor.2.0.4.mag. |
| 3108 | K.5.gabor.2.0.5.mean. |
| 3109 | K.5.gabor.2.0.5.var. |
| 3110 | K.5.gabor.2.0.5.absmean. |
| 3111 | K.5.gabor.2.0.5.mag. |
| 3112 | K.5.gabor.3.0.1.mean. |
| 3113 | K.5.gabor.3.0.1.var. |
| 3114 | K.5.gabor.3.0.1.absmean. |
| 3115 | K.5.gabor.3.0.1.mag. |
| 3116 | K.5.gabor.3.0.2.mean. |
| 3117 | K.5.gabor.3.0.2.var. |
| 3118 | K.5.gabor.3.0.2.absmean. |
| 3119 | K.5.gabor.3.0.2.mag. |
| 3120 | K.5.gabor.3.0.3.mean. |
| 3121 | K.5.gabor.3.0.3.var. |
| 3122 | K.5.gabor.3.0.3.absmean. |
| 3123 | K.5.gabor.3.0.3.mag. |
| 3124 | K.5.gabor.3.0.4.mean. |
| 3125 | K.5.gabor.3.0.4.var. |
| 3126 | K.5.gabor.3.0.4.absmean. |
| 3127 | K.5.gabor.3.0.4.mag. |
| 3128 | K.5.gabor.3.0.5.mean. |
| 3129 | K.5.gabor.3.0.5.var. |
| 3130 | K.5.gabor.3.0.5.absmean. |
| 3131 | K.5.gabor.3.0.5.mag. |
| 3132 | K.7.gabor.1.0.1.mean. |
| 3133 | K.7.gabor.1.0.1.var. |
| 3134 | K.7.gabor.1.0.1.absmean. |
| 3135 | K.7.gabor.1.0.1.mag. |
| 3136 | K.7.gabor.1.0.2.mean. |
| 3137 | K.7.gabor.1.0.2.var. |
| 3138 | K.7.gabor.1.0.2.absmean. |
| 3139 | K.7.gabor.1.0.2.mag. |
| 3140 | K.7.gabor.1.0.3.mean. |
| 3141 | K.7.gabor.1.0.3.var. |
| 3142 | K.7.gabor.1.0.3.absmean. |
| 3143 | K.7.gabor.1.0.3.mag. |
| 3144 | K.7.gabor.1.0.4.mean. |
| 3145 | K.7.gabor.1.0.4.var. |
| 3146 | K.7.gabor.1.0.4.absmean. |
| 3147 | K.7.gabor.1.0.4.mag. |
| 3148 | K.7.gabor.1.0.5.mean. |
| 3149 | K.7.gabor.1.0.5.var. |
| 3150 | K.7.gabor.1.0.5.absmean. |
| 3151 | K.7.gabor.1.0.5.mag. |
| 3152 | K.7.gabor.2.0.1.mean. |
| 3153 | K.7.gabor.2.0.1.var. |
| 3154 | K.7.gabor.2.0.1.absmean. |
| 3155 | K.7.gabor.2.0.1.mag. |
| 3156 | K.7.gabor.2.0.2.mean. |
| 3157 | K.7.gabor.2.0.2.var. |
| 3158 | K.7.gabor.2.0.2.absmean. |
| 3159 | K.7.gabor.2.0.2.mag. |
| 3160 | K.7.gabor.2.0.3.mean. |
| 3161 | K.7.gabor.2.0.3.var. |
| 3162 | K.7.gabor.2.0.3.absmean. |
| 3163 | K.7.gabor.2.0.3.mag. |
| 3164 | K.7.gabor.2.0.4.mean. |
| 3165 | K.7.gabor.2.0.4.var. |
| 3166 | K.7.gabor.2.0.4.absmean. |
| 3167 | K.7.gabor.2.0.4.mag. |
| 3168 | K.7.gabor.2.0.5.mean. |
| 3169 | K.7.gabor.2.0.5.var. |
| 3170 | K.7.gabor.2.0.5.absmean. |
| 3171 | K.7.gabor.2.0.5.mag. |
| 3172 | K.7.gabor.3.0.1.mean. |
| 3173 | K.7.gabor.3.0.1.var. |
| 3174 | K.7.gabor.3.0.1.absmean. |
| 3175 | K.7.gabor.3.0.1.mag. |
| 3176 | K.7.gabor.3.0.2.mean. |
| 3177 | K.7.gabor.3.0.2.var. |
| 3178 | K.7.gabor.3.0.2.absmean. |
| 3179 | K.7.gabor.3.0.2.mag. |
| 3180 | K.7.gabor.3.0.3.mean. |
| 3181 | K.7.gabor.3.0.3.var. |
| 3182 | K.7.gabor.3.0.3.absmean. |
| 3183 | K.7.gabor.3.0.3.mag. |
| 3184 | K.7.gabor.3.0.4.mean. |
| 3185 | K.7.gabor.3.0.4.var. |
| 3186 | K.7.gabor.3.0.4.absmean. |
| 3187 | K.7.gabor.3.0.4.mag. |
| 3188 | K.7.gabor.3.0.5.mean. |
| 3189 | K.7.gabor.3.0.5.var. |
| 3190 | K.7.gabor.3.0.5.absmean. |
| 3191 | K.7.gabor.3.0.5.mag. |
| 3192 | K.9.gabor.1.0.1.mean. |
| 3193 | K.9.gabor.1.0.1.var. |
| 3194 | K.9.gabor.1.0.1.absmean. |
| 3195 | K.9.gabor.1.0.1.mag. |
| 3196 | K.9.gabor.1.0.2.mean. |
| 3197 | K.9.gabor.1.0.2.var. |
| 3198 | K.9.gabor.1.0.2.absmean. |
| 3199 | K.9.gabor.1.0.2.mag. |
| 3200 | K.9.gabor.1.0.3.mean. |
| 3201 | K.9.gabor.1.0.3.var. |
| 3202 | K.9.gabor.1.0.3.absmean. |
| 3203 | K.9.gabor.1.0.3.mag. |
| 3204 | K.9.gabor.1.0.4.mean. |
| 3205 | K.9.gabor.1.0.4.var. |
| 3206 | K.9.gabor.1.0.4.absmean. |
| 3207 | K.9.gabor.1.0.4.mag. |
| 3208 | K.9.gabor.1.0.5.mean. |
| 3209 | K.9.gabor.1.0.5.var. |
| 3210 | K.9.gabor.1.0.5.absmean. |
| 3211 | K.9.gabor.1.0.5.mag. |
| 3212 | K.9.gabor.2.0.1.mean. |
| 3213 | K.9.gabor.2.0.1.var. |
| 3214 | K.9.gabor.2.0.1.absmean. |
| 3215 | K.9.gabor.2.0.1.mag. |
| 3216 | K.9.gabor.2.0.2.mean. |
| 3217 | K.9.gabor.2.0.2.var. |
| 3218 | K.9.gabor.2.0.2.absmean. |
| 3219 | K.9.gabor.2.0.2.mag. |
| 3220 | K.9.gabor.2.0.3.mean. |
| 3221 | K.9.gabor.2.0.3.var. |
| 3222 | K.9.gabor.2.0.3.absmean. |
| 3223 | K.9.gabor.2.0.3.mag. |
| 3224 | K.9.gabor.2.0.4.mean. |
| 3225 | K.9.gabor.2.0.4.var. |
| 3226 | K.9.gabor.2.0.4.absmean. |
| 3227 | K.9.gabor.2.0.4.mag. |
| 3228 | K.9.gabor.2.0.5.mean. |
| 3229 | K.9.gabor.2.0.5.var. |
| 3230 | K.9.gabor.2.0.5.absmean. |
| 3231 | K.9.gabor.2.0.5.mag. |
| 3232 | K.9.gabor.3.0.1.mean. |
| 3233 | K.9.gabor.3.0.1.var. |
| 3234 | K.9.gabor.3.0.1.absmean. |
| 3235 | K.9.gabor.3.0.1.mag. |
| 3236 | K.9.gabor.3.0.2.mean. |
| 3237 | K.9.gabor.3.0.2.var. |
| 3238 | K.9.gabor.3.0.2.absmean. |
| 3239 | K.9.gabor.3.0.2.mag. |
| 3240 | K.9.gabor.3.0.3.mean. |
| 3241 | K.9.gabor.3.0.3.var. |
| 3242 | K.9.gabor.3.0.3.absmean. |
| 3243 | K.9.gabor.3.0.3.mag. |
| 3244 | K.9.gabor.3.0.4.mean. |
| 3245 | K.9.gabor.3.0.4.var. |
| 3246 | K.9.gabor.3.0.4.absmean. |
| 3247 | K.9.gabor.3.0.4.mag. |
| 3248 | K.9.gabor.3.0.5.mean. |
| 3249 | K.9.gabor.3.0.5.var. |
| 3250 | K.9.gabor.3.0.5.absmean. |
| 3251 | K.9.gabor.3.0.5.mag. |
| 3252 | K.11.gabor.1.0.1.mean. |
| 3253 | K.11.gabor.1.0.1.var. |
| 3254 | K.11.gabor.1.0.1.absmean. |
| 3255 | K.11.gabor.1.0.1.mag. |
| 3256 | K.11.gabor.1.0.2.mean. |
| 3257 | K.11.gabor.1.0.2.var. |
| 3258 | K.11.gabor.1.0.2.absmean. |
| 3259 | K.11.gabor.1.0.2.mag. |
| 3260 | K.11.gabor.1.0.3.mean. |
| 3261 | K.11.gabor.1.0.3.var. |
| 3262 | K.11.gabor.1.0.3.absmean. |
| 3263 | K.11.gabor.1.0.3.mag. |
| 3264 | K.11.gabor.1.0.4.mean. |
| 3265 | K.11.gabor.1.0.4.var. |
| 3266 | K.11.gabor.1.0.4.absmean. |
| 3267 | K.11.gabor.1.0.4.mag. |
| 3268 | K.11.gabor.1.0.5.mean. |
| 3269 | K.11.gabor.1.0.5.var. |
| 3270 | K.11.gabor.1.0.5.absmean. |
| 3271 | K.11.gabor.1.0.5.mag. |
| 3272 | K.11.gabor.2.0.1.mean. |
| 3273 | K.11.gabor.2.0.1.var. |
| 3274 | K.11.gabor.2.0.1.absmean. |
| 3275 | K.11.gabor.2.0.1.mag. |
| 3276 | K.11.gabor.2.0.2.mean. |
| 3277 | K.11.gabor.2.0.2.var. |
| 3278 | K.11.gabor.2.0.2.absmean. |
| 3279 | K.11.gabor.2.0.2.mag. |
| 3280 | K.11.gabor.2.0.3.mean. |
| 3281 | K.11.gabor.2.0.3.var. |
| 3282 | K.11.gabor.2.0.3.absmean. |
| 3283 | K.11.gabor.2.0.3.mag. |
| 3284 | K.11.gabor.2.0.4.mean. |
| 3285 | K.11.gabor.2.0.4.var. |
| 3286 | K.11.gabor.2.0.4.absmean. |
| 3287 | K.11.gabor.2.0.4.mag. |
| 3288 | K.11.gabor.2.0.5.mean. |
| 3289 | K.11.gabor.2.0.5.var. |
| 3290 | K.11.gabor.2.0.5.absmean. |
| 3291 | K.11.gabor.2.0.5.mag. |
| 3292 | K.11.gabor.3.0.1.mean. |
| 3293 | K.11.gabor.3.0.1.var. |
| 3294 | K.11.gabor.3.0.1.absmean. |
| 3295 | K.11.gabor.3.0.1.mag. |
| 3296 | K.11.gabor.3.0.2.mean. |
| 3297 | K.11.gabor.3.0.2.var. |
| 3298 | K.11.gabor.3.0.2.absmean. |
| 3299 | K.11.gabor.3.0.2.mag. |
| 3300 | K.11.gabor.3.0.3.mean. |
| 3301 | K.11.gabor.3.0.3.var. |
| 3302 | K.11.gabor.3.0.3.absmean. |
| 3303 | K.11.gabor.3.0.3.mag. |
| 3304 | K.11.gabor.3.0.4.mean. |
| 3305 | K.11.gabor.3.0.4.var. |
| 3306 | K.11.gabor.3.0.4.absmean. |
| 3307 | K.11.gabor.3.0.4.mag. |
| 3308 | K.11.gabor.3.0.5.mean. |
| 3309 | K.11.gabor.3.0.5.var. |
| 3310 | K.11.gabor.3.0.5.absmean. |
| 3311 | K.11.gabor.3.0.5.mag. |
| 3312 | K.13.gabor.1.0.1.mean. |
| 3313 | K.13.gabor.1.0.1.var. |
| 3314 | K.13.gabor.1.0.1.absmean. |
| 3315 | K.13.gabor.1.0.1.mag. |
| 3316 | K.13.gabor.1.0.2.mean. |
| 3317 | K.13.gabor.1.0.2.var. |
| 3318 | K.13.gabor.1.0.2.absmean. |
| 3319 | K.13.gabor.1.0.2.mag. |
| 3320 | K.13.gabor.1.0.3.mean. |
| 3321 | K.13.gabor.1.0.3.var. |
| 3322 | K.13.gabor.1.0.3.absmean. |
| 3323 | K.13.gabor.1.0.3.mag. |
| 3324 | K.13.gabor.1.0.4.mean. |
| 3325 | K.13.gabor.1.0.4.var. |
| 3326 | K.13.gabor.1.0.4.absmean. |
| 3327 | K.13.gabor.1.0.4.mag. |
| 3328 | K.13.gabor.1.0.5.mean. |
| 3329 | K.13.gabor.1.0.5.var. |
| 3330 | K.13.gabor.1.0.5.absmean. |
| 3331 | K.13.gabor.1.0.5.mag. |
| 3332 | K.13.gabor.2.0.1.mean. |
| 3333 | K.13.gabor.2.0.1.var. |
| 3334 | K.13.gabor.2.0.1.absmean. |
| 3335 | K.13.gabor.2.0.1.mag. |
| 3336 | K.13.gabor.2.0.2.mean. |
| 3337 | K.13.gabor.2.0.2.var. |
| 3338 | K.13.gabor.2.0.2.absmean. |
| 3339 | K.13.gabor.2.0.2.mag. |
| 3340 | K.13.gabor.2.0.3.mean. |
| 3341 | K.13.gabor.2.0.3.var. |
| 3342 | K.13.gabor.2.0.3.absmean. |
| 3343 | K.13.gabor.2.0.3.mag. |
| 3344 | K.13.gabor.2.0.4.mean. |
| 3345 | K.13.gabor.2.0.4.var. |
| 3346 | K.13.gabor.2.0.4.absmean. |
| 3347 | K.13.gabor.2.0.4.mag. |
| 3348 | K.13.gabor.2.0.5.mean. |
| 3349 | K.13.gabor.2.0.5.var. |
| 3350 | K.13.gabor.2.0.5.absmean. |
| 3351 | K.13.gabor.2.0.5.mag. |
| 3352 | K.13.gabor.3.0.1.mean. |
| 3353 | K.13.gabor.3.0.1.var. |
| 3354 | K.13.gabor.3.0.1.absmean. |
| 3355 | K.13.gabor.3.0.1.mag. |
| 3356 | K.13.gabor.3.0.2.mean. |
| 3357 | K.13.gabor.3.0.2.var. |
| 3358 | K.13.gabor.3.0.2.absmean. |
| 3359 | K.13.gabor.3.0.2.mag. |
| 3360 | K.13.gabor.3.0.3.mean. |
| 3361 | K.13.gabor.3.0.3.var. |
| 3362 | K.13.gabor.3.0.3.absmean. |
| 3363 | K.13.gabor.3.0.3.mag. |
| 3364 | K.13.gabor.3.0.4.mean. |
| 3365 | K.13.gabor.3.0.4.var. |
| 3366 | K.13.gabor.3.0.4.absmean. |
| 3367 | K.13.gabor.3.0.4.mag. |
| 3368 | K.13.gabor.3.0.5.mean. |
| 3369 | K.13.gabor.3.0.5.var. |
| 3370 | K.13.gabor.3.0.5.absmean. |
| 3371 | K.13.gabor.3.0.5.mag. |
| 3372 | K.15.gabor.1.0.1.mean. |
| 3373 | K.15.gabor.1.0.1.var. |
| 3374 | K.15.gabor.1.0.1.absmean. |
| 3375 | K.15.gabor.1.0.1.mag. |
| 3376 | K.15.gabor.1.0.2.mean. |
| 3377 | K.15.gabor.1.0.2.var. |
| 3378 | K.15.gabor.1.0.2.absmean. |
| 3379 | K.15.gabor.1.0.2.mag. |
| 3380 | K.15.gabor.1.0.3.mean. |
| 3381 | K.15.gabor.1.0.3.var. |
| 3382 | K.15.gabor.1.0.3.absmean. |
| 3383 | K.15.gabor.1.0.3.mag. |
| 3384 | K.15.gabor.1.0.4.mean. |
| 3385 | K.15.gabor.1.0.4.var. |
| 3386 | K.15.gabor.1.0.4.absmean. |
| 3387 | K.15.gabor.1.0.4.mag. |
| 3388 | K.15.gabor.1.0.5.mean. |
| 3389 | K.15.gabor.1.0.5.var. |
| 3390 | K.15.gabor.1.0.5.absmean. |
| 3391 | K.15.gabor.1.0.5.mag. |
| 3392 | K.15.gabor.2.0.1.mean. |
| 3393 | K.15.gabor.2.0.1.var. |
| 3394 | K.15.gabor.2.0.1.absmean. |
| 3395 | K.15.gabor.2.0.1.mag. |
| 3396 | K.15.gabor.2.0.2.mean. |
| 3397 | K.15.gabor.2.0.2.var. |
| 3398 | K.15.gabor.2.0.2.absmean. |
| 3399 | K.15.gabor.2.0.2.mag. |
| 3400 | K.15.gabor.2.0.3.mean. |
| 3401 | K.15.gabor.2.0.3.var. |
| 3402 | K.15.gabor.2.0.3.absmean. |
| 3403 | K.15.gabor.2.0.3.mag. |
| 3404 | K.15.gabor.2.0.4.mean. |
| 3405 | K.15.gabor.2.0.4.var. |
| 3406 | K.15.gabor.2.0.4.absmean. |
| 3407 | K.15.gabor.2.0.4.mag. |
| 3408 | K.15.gabor.2.0.5.mean. |
| 3409 | K.15.gabor.2.0.5.var. |
| 3410 | K.15.gabor.2.0.5.absmean. |
| 3411 | K.15.gabor.2.0.5.mag. |
| 3412 | K.15.gabor.3.0.1.mean. |
| 3413 | K.15.gabor.3.0.1.var. |
| 3414 | K.15.gabor.3.0.1.absmean. |
| 3415 | K.15.gabor.3.0.1.mag. |
| 3416 | K.15.gabor.3.0.2.mean. |
| 3417 | K.15.gabor.3.0.2.var. |
| 3418 | K.15.gabor.3.0.2.absmean. |
| 3419 | K.15.gabor.3.0.2.mag. |
| 3420 | K.15.gabor.3.0.3.mean. |
| 3421 | K.15.gabor.3.0.3.var. |
| 3422 | K.15.gabor.3.0.3.absmean. |
| 3423 | K.15.gabor.3.0.3.mag. |
| 3424 | K.15.gabor.3.0.4.mean. |
| 3425 | K.15.gabor.3.0.4.var. |
| 3426 | K.15.gabor.3.0.4.absmean. |
| 3427 | K.15.gabor.3.0.4.mag. |
| 3428 | K.15.gabor.3.0.5.mean. |
| 3429 | K.15.gabor.3.0.5.var. |
| 3430 | K.15.gabor.3.0.5.absmean. |
| 3431 | K.15.gabor.3.0.5.mag. |
| 3432 | K.3.haar.1.1.aav. |
| 3433 | K.3.haar.1.1.std. |
| 3434 | K.3.haar.1.2.aav. |
| 3435 | K.3.haar.1.2.std. |
| 3436 | K.3.haar.1.3.aav. |
| 3437 | K.3.haar.1.3.std. |
| 3438 | K.3.haar.2.1.aav. |
| 3439 | K.3.haar.2.1.std. |
| 3440 | K.3.haar.2.2.aav. |
| 3441 | K.3.haar.2.2.std. |
| 3442 | K.3.haar.2.3.aav. |
| 3443 | K.3.haar.2.3.std. |
| 3444 | K.3.haar.3.1.aav. |
| 3445 | K.3.haar.3.1.std. |
| 3446 | K.3.haar.3.2.aav. |
| 3447 | K.3.haar.3.2.std. |
| 3448 | K.3.haar.3.3.aav. |
| 3449 | K.3.haar.3.3.std. |
| 3450 | K.3.haar.4.1.aav. |
| 3451 | K.3.haar.4.1.std. |
| 3452 | K.3.haar.4.2.aav. |
| 3453 | K.3.haar.4.2.std. |
| 3454 | K.3.haar.4.3.aav. |
| 3455 | K.3.haar.4.3.std. |
| 3456 | K.5.haar.1.1.aav. |
| 3457 | K.5.haar.1.1.std. |
| 3458 | K.5.haar.1.2.aav. |
| 3459 | K.5.haar.1.2.std. |
| 3460 | K.5.haar.1.3.aav. |
| 3461 | K.5.haar.1.3.std. |
| 3462 | K.5.haar.2.1.aav. |
| 3463 | K.5.haar.2.1.std. |
| 3464 | K.5.haar.2.2.aav. |
| 3465 | K.5.haar.2.2.std. |
| 3466 | K.5.haar.2.3.aav. |
| 3467 | K.5.haar.2.3.std. |
| 3468 | K.5.haar.3.1.aav. |
| 3469 | K.5.haar.3.1.std. |
| 3470 | K.5.haar.3.2.aav. |
| 3471 | K.5.haar.3.2.std. |
| 3472 | K.5.haar.3.3.aav. |
| 3473 | K.5.haar.3.3.std. |
| 3474 | K.5.haar.4.1.aav. |
| 3475 | K.5.haar.4.1.std. |
| 3476 | K.5.haar.4.2.aav. |
| 3477 | K.5.haar.4.2.std. |
| 3478 | K.5.haar.4.3.aav. |
| 3479 | K.5.haar.4.3.std. |
| 3480 | K.7.haar.1.1.aav. |
| 3481 | K.7.haar.1.1.std. |
| 3482 | K.7.haar.1.2.aav. |
| 3483 | K.7.haar.1.2.std. |
| 3484 | K.7.haar.1.3.aav. |
| 3485 | K.7.haar.1.3.std. |
| 3486 | K.7.haar.2.1.aav. |
| 3487 | K.7.haar.2.1.std. |
| 3488 | K.7.haar.2.2.aav. |
| 3489 | K.7.haar.2.2.std. |
| 3490 | K.7.haar.2.3.aav. |
| 3491 | K.7.haar.2.3.std. |
| 3492 | K.7.haar.3.1.aav. |
| 3493 | K.7.haar.3.1.std. |
| 3494 | K.7.haar.3.2.aav. |
| 3495 | K.7.haar.3.2.std. |
| 3496 | K.7.haar.3.3.aav. |
| 3497 | K.7.haar.3.3.std. |
| 3498 | K.7.haar.4.1.aav. |
| 3499 | K.7.haar.4.1.std. |
| 3500 | K.7.haar.4.2.aav. |
| 3501 | K.7.haar.4.2.std. |
| 3502 | K.7.haar.4.3.aav. |
| 3503 | K.7.haar.4.3.std. |
| 3504 | K.9.haar.1.1.aav. |
| 3505 | K.9.haar.1.1.std. |
| 3506 | K.9.haar.1.2.aav. |
| 3507 | K.9.haar.1.2.std. |
| 3508 | K.9.haar.1.3.aav. |
| 3509 | K.9.haar.1.3.std. |
| 3510 | K.9.haar.2.1.aav. |
| 3511 | K.9.haar.2.1.std. |
| 3512 | K.9.haar.2.2.aav. |
| 3513 | K.9.haar.2.2.std. |
| 3514 | K.9.haar.2.3.aav. |
| 3515 | K.9.haar.2.3.std. |
| 3516 | K.9.haar.3.1.aav. |
| 3517 | K.9.haar.3.1.std. |
| 3518 | K.9.haar.3.2.aav. |
| 3519 | K.9.haar.3.2.std. |
| 3520 | K.9.haar.3.3.aav. |
| 3521 | K.9.haar.3.3.std. |
| 3522 | K.9.haar.4.1.aav. |
| 3523 | K.9.haar.4.1.std. |
| 3524 | K.9.haar.4.2.aav. |
| 3525 | K.9.haar.4.2.std. |
| 3526 | K.9.haar.4.3.aav. |
| 3527 | K.9.haar.4.3.std. |
| 3528 | K.11.haar.1.1.aav. |
| 3529 | K.11.haar.1.1.std. |
| 3530 | K.11.haar.1.2.aav. |
| 3531 | K.11.haar.1.2.std. |
| 3532 | K.11.haar.1.3.aav. |
| 3533 | K.11.haar.1.3.std. |
| 3534 | K.11.haar.2.1.aav. |
| 3535 | K.11.haar.2.1.std. |
| 3536 | K.11.haar.2.2.aav. |
| 3537 | K.11.haar.2.2.std. |
| 3538 | K.11.haar.2.3.aav. |
| 3539 | K.11.haar.2.3.std. |
| 3540 | K.11.haar.3.1.aav. |
| 3541 | K.11.haar.3.1.std. |
| 3542 | K.11.haar.3.2.aav. |
| 3543 | K.11.haar.3.2.std. |
| 3544 | K.11.haar.3.3.aav. |
| 3545 | K.11.haar.3.3.std. |
| 3546 | K.11.haar.4.1.aav. |
| 3547 | K.11.haar.4.1.std. |
| 3548 | K.11.haar.4.2.aav. |
| 3549 | K.11.haar.4.2.std. |
| 3550 | K.11.haar.4.3.aav. |
| 3551 | K.11.haar.4.3.std. |
| 3552 | K.13.haar.1.1.aav. |
| 3553 | K.13.haar.1.1.std. |
| 3554 | K.13.haar.1.2.aav. |
| 3555 | K.13.haar.1.2.std. |
| 3556 | K.13.haar.1.3.aav. |
| 3557 | K.13.haar.1.3.std. |
| 3558 | K.13.haar.2.1.aav. |
| 3559 | K.13.haar.2.1.std. |
| 3560 | K.13.haar.2.2.aav. |
| 3561 | K.13.haar.2.2.std. |
| 3562 | K.13.haar.2.3.aav. |
| 3563 | K.13.haar.2.3.std. |
| 3564 | K.13.haar.3.1.aav. |
| 3565 | K.13.haar.3.1.std. |
| 3566 | K.13.haar.3.2.aav. |
| 3567 | K.13.haar.3.2.std. |
| 3568 | K.13.haar.3.3.aav. |
| 3569 | K.13.haar.3.3.std. |
| 3570 | K.13.haar.4.1.aav. |
| 3571 | K.13.haar.4.1.std. |
| 3572 | K.13.haar.4.2.aav. |
| 3573 | K.13.haar.4.2.std. |
| 3574 | K.13.haar.4.3.aav. |
| 3575 | K.13.haar.4.3.std. |
| 3576 | K.15.haar.1.1.aav. |
| 3577 | K.15.haar.1.1.std. |
| 3578 | K.15.haar.1.2.aav. |
| 3579 | K.15.haar.1.2.std. |
| 3580 | K.15.haar.1.3.aav. |
| 3581 | K.15.haar.1.3.std. |
| 3582 | K.15.haar.2.1.aav. |
| 3583 | K.15.haar.2.1.std. |
| 3584 | K.15.haar.2.2.aav. |
| 3585 | K.15.haar.2.2.std. |
| 3586 | K.15.haar.2.3.aav. |
| 3587 | K.15.haar.2.3.std. |
| 3588 | K.15.haar.3.1.aav. |
| 3589 | K.15.haar.3.1.std. |
| 3590 | K.15.haar.3.2.aav. |
| 3591 | K.15.haar.3.2.std. |
| 3592 | K.15.haar.3.3.aav. |
| 3593 | K.15.haar.3.3.std. |
| 3594 | K.15.haar.4.1.aav. |
| 3595 | K.15.haar.4.1.std. |
| 3596 | K.15.haar.4.2.aav. |
| 3597 | K.15.haar.4.2.std. |
| 3598 | K.15.haar.4.3.aav. |
| 3599 | K.15.haar.4.3.std. |
| 3600 | K.3.hu.0. |
| 3601 | K.3.hu.1. |
| 3602 | K.3.hu.2. |
| 3603 | K.3.hu.3. |
| 3604 | K.3.hu.4. |
| 3605 | K.3.hu.5. |
| 3606 | K.3.hu.6. |
| 3607 | K.5.hu.0. |
| 3608 | K.5.hu.1. |
| 3609 | K.5.hu.2. |
| 3610 | K.5.hu.3. |
| 3611 | K.5.hu.4. |
| 3612 | K.5.hu.5. |
| 3613 | K.5.hu.6. |
| 3614 | K.7.hu.0. |
| 3615 | K.7.hu.1. |
| 3616 | K.7.hu.2. |
| 3617 | K.7.hu.3. |
| 3618 | K.7.hu.4. |
| 3619 | K.7.hu.5. |
| 3620 | K.7.hu.6. |
| 3621 | K.9.hu.0. |
| 3622 | K.9.hu.1. |
| 3623 | K.9.hu.2. |
| 3624 | K.9.hu.3. |
| 3625 | K.9.hu.4. |
| 3626 | K.9.hu.5. |
| 3627 | K.9.hu.6. |
| 3628 | K.11.hu.0. |
| 3629 | K.11.hu.1. |
| 3630 | K.11.hu.2. |
| 3631 | K.11.hu.3. |
| 3632 | K.11.hu.4. |
| 3633 | K.11.hu.5. |
| 3634 | K.11.hu.6. |
| 3635 | K.13.hu.0. |
| 3636 | K.13.hu.1. |
| 3637 | K.13.hu.2. |
| 3638 | K.13.hu.3. |
| 3639 | K.13.hu.4. |
| 3640 | K.13.hu.5. |
| 3641 | K.13.hu.6. |
| 3642 | K.15.hu.0. |
| 3643 | K.15.hu.1. |
| 3644 | K.15.hu.2. |
| 3645 | K.15.hu.3. |
| 3646 | K.15.hu.4. |
| 3647 | K.15.hu.5. |
| 3648 | K.15.hu.6. |
| 3649 | K.3.zernike.0. |
| 3650 | K.3.zernike.1. |
| 3651 | K.3.zernike.2. |
| 3652 | K.3.zernike.3. |
| 3653 | K.3.zernike.4. |
| 3654 | K.3.zernike.5. |
| 3655 | K.3.zernike.6. |
| 3656 | K.3.zernike.7. |
| 3657 | K.3.zernike.8. |
| 3658 | K.3.zernike.9. |
| 3659 | K.3.zernike.10. |
| 3660 | K.3.zernike.11. |
| 3661 | K.3.zernike.12. |
| 3662 | K.3.zernike.13. |
| 3663 | K.3.zernike.14. |
| 3664 | K.3.zernike.15. |
| 3665 | K.3.zernike.16. |
| 3666 | K.3.zernike.17. |
| 3667 | K.3.zernike.18. |
| 3668 | K.3.zernike.19. |
| 3669 | K.3.zernike.20. |
| 3670 | K.3.zernike.21. |
| 3671 | K.3.zernike.22. |
| 3672 | K.3.zernike.23. |
| 3673 | K.3.zernike.24. |
| 3674 | K.5.zernike.0. |
| 3675 | K.5.zernike.1. |
| 3676 | K.5.zernike.2. |
| 3677 | K.5.zernike.3. |
| 3678 | K.5.zernike.4. |
| 3679 | K.5.zernike.5. |
| 3680 | K.5.zernike.6. |
| 3681 | K.5.zernike.7. |
| 3682 | K.5.zernike.8. |
| 3683 | K.5.zernike.9. |
| 3684 | K.5.zernike.10. |
| 3685 | K.5.zernike.11. |
| 3686 | K.5.zernike.12. |
| 3687 | K.5.zernike.13. |
| 3688 | K.5.zernike.14. |
| 3689 | K.5.zernike.15. |
| 3690 | K.5.zernike.16. |
| 3691 | K.5.zernike.17. |
| 3692 | K.5.zernike.18. |
| 3693 | K.5.zernike.19. |
| 3694 | K.5.zernike.20. |
| 3695 | K.5.zernike.21. |
| 3696 | K.5.zernike.22. |
| 3697 | K.5.zernike.23. |
| 3698 | K.5.zernike.24. |
| 3699 | K.7.zernike.0. |
| 3700 | K.7.zernike.1. |
| 3701 | K.7.zernike.2. |
| 3702 | K.7.zernike.3. |
| 3703 | K.7.zernike.4. |
| 3704 | K.7.zernike.5. |
| 3705 | K.7.zernike.6. |
| 3706 | K.7.zernike.7. |
| 3707 | K.7.zernike.8. |
| 3708 | K.7.zernike.9. |
| 3709 | K.7.zernike.10. |
| 3710 | K.7.zernike.11. |
| 3711 | K.7.zernike.12. |
| 3712 | K.7.zernike.13. |
| 3713 | K.7.zernike.14. |
| 3714 | K.7.zernike.15. |
| 3715 | K.7.zernike.16. |
| 3716 | K.7.zernike.17. |
| 3717 | K.7.zernike.18. |
| 3718 | K.7.zernike.19. |
| 3719 | K.7.zernike.20. |
| 3720 | K.7.zernike.21. |
| 3721 | K.7.zernike.22. |
| 3722 | K.7.zernike.23. |
| 3723 | K.7.zernike.24. |
| 3724 | K.9.zernike.0. |
| 3725 | K.9.zernike.1. |
| 3726 | K.9.zernike.2. |
| 3727 | K.9.zernike.3. |
| 3728 | K.9.zernike.4. |
| 3729 | K.9.zernike.5. |
| 3730 | K.9.zernike.6. |
| 3731 | K.9.zernike.7. |
| 3732 | K.9.zernike.8. |
| 3733 | K.9.zernike.9. |
| 3734 | K.9.zernike.10. |
| 3735 | K.9.zernike.11. |
| 3736 | K.9.zernike.12. |
| 3737 | K.9.zernike.13. |
| 3738 | K.9.zernike.14. |
| 3739 | K.9.zernike.15. |
| 3740 | K.9.zernike.16. |
| 3741 | K.9.zernike.17. |
| 3742 | K.9.zernike.18. |
| 3743 | K.9.zernike.19. |
| 3744 | K.9.zernike.20. |
| 3745 | K.9.zernike.21. |
| 3746 | K.9.zernike.22. |
| 3747 | K.9.zernike.23. |
| 3748 | K.9.zernike.24. |
| 3749 | K.11.zernike.0. |
| 3750 | K.11.zernike.1. |
| 3751 | K.11.zernike.2. |
| 3752 | K.11.zernike.3. |
| 3753 | K.11.zernike.4. |
| 3754 | K.11.zernike.5. |
| 3755 | K.11.zernike.6. |
| 3756 | K.11.zernike.7. |
| 3757 | K.11.zernike.8. |
| 3758 | K.11.zernike.9. |
| 3759 | K.11.zernike.10. |
| 3760 | K.11.zernike.11. |
| 3761 | K.11.zernike.12. |
| 3762 | K.11.zernike.13. |
| 3763 | K.11.zernike.14. |
| 3764 | K.11.zernike.15. |
| 3765 | K.11.zernike.16. |
| 3766 | K.11.zernike.17. |
| 3767 | K.11.zernike.18. |
| 3768 | K.11.zernike.19. |
| 3769 | K.11.zernike.20. |
| 3770 | K.11.zernike.21. |
| 3771 | K.11.zernike.22. |
| 3772 | K.11.zernike.23. |
| 3773 | K.11.zernike.24. |
| 3774 | K.13.zernike.0. |
| 3775 | K.13.zernike.1. |
| 3776 | K.13.zernike.2. |
| 3777 | K.13.zernike.3. |
| 3778 | K.13.zernike.4. |
| 3779 | K.13.zernike.5. |
| 3780 | K.13.zernike.6. |
| 3781 | K.13.zernike.7. |
| 3782 | K.13.zernike.8. |
| 3783 | K.13.zernike.9. |
| 3784 | K.13.zernike.10. |
| 3785 | K.13.zernike.11. |
| 3786 | K.13.zernike.12. |
| 3787 | K.13.zernike.13. |
| 3788 | K.13.zernike.14. |
| 3789 | K.13.zernike.15. |
| 3790 | K.13.zernike.16. |
| 3791 | K.13.zernike.17. |
| 3792 | K.13.zernike.18. |
| 3793 | K.13.zernike.19. |
| 3794 | K.13.zernike.20. |
| 3795 | K.13.zernike.21. |
| 3796 | K.13.zernike.22. |
| 3797 | K.13.zernike.23. |
| 3798 | K.13.zernike.24. |
| 3799 | K.15.zernike.0. |
| 3800 | K.15.zernike.1. |
| 3801 | K.15.zernike.2. |
| 3802 | K.15.zernike.3. |
| 3803 | K.15.zernike.4. |
| 3804 | K.15.zernike.5. |
| 3805 | K.15.zernike.6. |
| 3806 | K.15.zernike.7. |
| 3807 | K.15.zernike.8. |
| 3808 | K.15.zernike.9. |
| 3809 | K.15.zernike.10. |
| 3810 | K.15.zernike.11. |
| 3811 | K.15.zernike.12. |
| 3812 | K.15.zernike.13. |
| 3813 | K.15.zernike.14. |
| 3814 | K.15.zernike.15. |
| 3815 | K.15.zernike.16. |
| 3816 | K.15.zernike.17. |
| 3817 | K.15.zernike.18. |
| 3818 | K.15.zernike.19. |
| 3819 | K.15.zernike.20. |
| 3820 | K.15.zernike.21. |
| 3821 | K.15.zernike.22. |
| 3822 | K.15.zernike.23. |
| 3823 | K.15.zernike.24. |
| 3824 | K.3.sobel |
| 3825 | K.3.sobel_mask |
| 3826 | K.all.stats.p000. |
| 3827 | K.all.stats.p010. |
| 3828 | K.all.stats.p020. |
| 3829 | K.all.stats.p025. |
| 3830 | K.all.stats.p030. |
| 3831 | K.all.stats.p040. |
| 3832 | K.all.stats.p050. |
| 3833 | K.all.stats.p060. |
| 3834 | K.all.stats.p070. |
| 3835 | K.all.stats.p075. |
| 3836 | K.all.stats.p080. |
| 3837 | K.all.stats.p090. |
| 3838 | K.all.stats.p100. |
| 3839 | K.all.stats.range. |
| 3840 | K.all.stats.mean. |
| 3841 | K.all.stats.stddev. |
| 3842 | K.all.stats.kurtosis. |
| 3843 | K.all.stats.skewness. |
| 3844 | T2.3.glcm.contrast.1.mean. |
| 3845 | T2.3.glcm.contrast.1.range. |
| 3846 | T2.3.glcm.contrast.2.mean. |
| 3847 | T2.3.glcm.contrast.2.range. |
| 3848 | T2.3.glcm.contrast.3.mean. |
| 3849 | T2.3.glcm.contrast.3.range. |
| 3850 | T2.3.glcm.dissimilarity.1.mean. |
| 3851 | T2.3.glcm.dissimilarity.1.range. |
| 3852 | T2.3.glcm.dissimilarity.2.mean. |
| 3853 | T2.3.glcm.dissimilarity.2.range. |
| 3854 | T2.3.glcm.dissimilarity.3.mean. |
| 3855 | T2.3.glcm.dissimilarity.3.range. |
| 3856 | T2.3.glcm.homogeneity.1.mean. |
| 3857 | T2.3.glcm.homogeneity.1.range. |
| 3858 | T2.3.glcm.homogeneity.2.mean. |
| 3859 | T2.3.glcm.homogeneity.2.range. |
| 3860 | T2.3.glcm.homogeneity.3.mean. |
| 3861 | T2.3.glcm.homogeneity.3.range. |
| 3862 | T2.3.glcm.energy.1.mean. |
| 3863 | T2.3.glcm.energy.1.range. |
| 3864 | T2.3.glcm.energy.2.mean. |
| 3865 | T2.3.glcm.energy.2.range. |
| 3866 | T2.3.glcm.energy.3.mean. |
| 3867 | T2.3.glcm.energy.3.range. |
| 3868 | T2.3.glcm.correlation.1.mean. |
| 3869 | T2.3.glcm.correlation.1.range. |
| 3870 | T2.3.glcm.correlation.2.mean. |
| 3871 | T2.3.glcm.correlation.2.range. |
| 3872 | T2.3.glcm.correlation.3.mean. |
| 3873 | T2.3.glcm.correlation.3.range. |
| 3874 | T2.3.glcm.ASM.1.mean. |
| 3875 | T2.3.glcm.ASM.1.range. |
| 3876 | T2.3.glcm.ASM.2.mean. |
| 3877 | T2.3.glcm.ASM.2.range. |
| 3878 | T2.3.glcm.ASM.3.mean. |
| 3879 | T2.3.glcm.ASM.3.range. |
| 3880 | T2.7.glcm.contrast.1.mean. |
| 3881 | T2.7.glcm.contrast.1.range. |
| 3882 | T2.7.glcm.contrast.2.mean. |
| 3883 | T2.7.glcm.contrast.2.range. |
| 3884 | T2.7.glcm.contrast.3.mean. |
| 3885 | T2.7.glcm.contrast.3.range. |
| 3886 | T2.7.glcm.contrast.4.mean. |
| 3887 | T2.7.glcm.contrast.4.range. |
| 3888 | T2.7.glcm.dissimilarity.1.mean. |
| 3889 | T2.7.glcm.dissimilarity.1.range. |
| 3890 | T2.7.glcm.dissimilarity.2.mean. |
| 3891 | T2.7.glcm.dissimilarity.2.range. |
| 3892 | T2.7.glcm.dissimilarity.3.mean. |
| 3893 | T2.7.glcm.dissimilarity.3.range. |
| 3894 | T2.7.glcm.dissimilarity.4.mean. |
| 3895 | T2.7.glcm.dissimilarity.4.range. |
| 3896 | T2.7.glcm.homogeneity.1.mean. |
| 3897 | T2.7.glcm.homogeneity.1.range. |
| 3898 | T2.7.glcm.homogeneity.2.mean. |
| 3899 | T2.7.glcm.homogeneity.2.range. |
| 3900 | T2.7.glcm.homogeneity.3.mean. |
| 3901 | T2.7.glcm.homogeneity.3.range. |
| 3902 | T2.7.glcm.homogeneity.4.mean. |
| 3903 | T2.7.glcm.homogeneity.4.range. |
| 3904 | T2.7.glcm.energy.1.mean. |
| 3905 | T2.7.glcm.energy.1.range. |
| 3906 | T2.7.glcm.energy.2.mean. |
| 3907 | T2.7.glcm.energy.2.range. |
| 3908 | T2.7.glcm.energy.3.mean. |
| 3909 | T2.7.glcm.energy.3.range. |
| 3910 | T2.7.glcm.energy.4.mean. |
| 3911 | T2.7.glcm.energy.4.range. |
| 3912 | T2.7.glcm.correlation.1.mean. |
| 3913 | T2.7.glcm.correlation.1.range. |
| 3914 | T2.7.glcm.correlation.2.mean. |
| 3915 | T2.7.glcm.correlation.2.range. |
| 3916 | T2.7.glcm.correlation.3.mean. |
| 3917 | T2.7.glcm.correlation.3.range. |
| 3918 | T2.7.glcm.correlation.4.mean. |
| 3919 | T2.7.glcm.correlation.4.range. |
| 3920 | T2.7.glcm.ASM.1.mean. |
| 3921 | T2.7.glcm.ASM.1.range. |
| 3922 | T2.7.glcm.ASM.2.mean. |
| 3923 | T2.7.glcm.ASM.2.range. |
| 3924 | T2.7.glcm.ASM.3.mean. |
| 3925 | T2.7.glcm.ASM.3.range. |
| 3926 | T2.7.glcm.ASM.4.mean. |
| 3927 | T2.7.glcm.ASM.4.range. |
| 3928 | T2.11.glcm.contrast.1.mean. |
| 3929 | T2.11.glcm.contrast.1.range. |
| 3930 | T2.11.glcm.contrast.2.mean. |
| 3931 | T2.11.glcm.contrast.2.range. |
| 3932 | T2.11.glcm.contrast.3.mean. |
| 3933 | T2.11.glcm.contrast.3.range. |
| 3934 | T2.11.glcm.contrast.4.mean. |
| 3935 | T2.11.glcm.contrast.4.range. |
| 3936 | T2.11.glcm.dissimilarity.1.mean. |
| 3937 | T2.11.glcm.dissimilarity.1.range. |
| 3938 | T2.11.glcm.dissimilarity.2.mean. |
| 3939 | T2.11.glcm.dissimilarity.2.range. |
| 3940 | T2.11.glcm.dissimilarity.3.mean. |
| 3941 | T2.11.glcm.dissimilarity.3.range. |
| 3942 | T2.11.glcm.dissimilarity.4.mean. |
| 3943 | T2.11.glcm.dissimilarity.4.range. |
| 3944 | T2.11.glcm.homogeneity.1.mean. |
| 3945 | T2.11.glcm.homogeneity.1.range. |
| 3946 | T2.11.glcm.homogeneity.2.mean. |
| 3947 | T2.11.glcm.homogeneity.2.range. |
| 3948 | T2.11.glcm.homogeneity.3.mean. |
| 3949 | T2.11.glcm.homogeneity.3.range. |
| 3950 | T2.11.glcm.homogeneity.4.mean. |
| 3951 | T2.11.glcm.homogeneity.4.range. |
| 3952 | T2.11.glcm.energy.1.mean. |
| 3953 | T2.11.glcm.energy.1.range. |
| 3954 | T2.11.glcm.energy.2.mean. |
| 3955 | T2.11.glcm.energy.2.range. |
| 3956 | T2.11.glcm.energy.3.mean. |
| 3957 | T2.11.glcm.energy.3.range. |
| 3958 | T2.11.glcm.energy.4.mean. |
| 3959 | T2.11.glcm.energy.4.range. |
| 3960 | T2.11.glcm.correlation.1.mean. |
| 3961 | T2.11.glcm.correlation.1.range. |
| 3962 | T2.11.glcm.correlation.2.mean. |
| 3963 | T2.11.glcm.correlation.2.range. |
| 3964 | T2.11.glcm.correlation.3.mean. |
| 3965 | T2.11.glcm.correlation.3.range. |
| 3966 | T2.11.glcm.correlation.4.mean. |
| 3967 | T2.11.glcm.correlation.4.range. |
| 3968 | T2.11.glcm.ASM.1.mean. |
| 3969 | T2.11.glcm.ASM.1.range. |
| 3970 | T2.11.glcm.ASM.2.mean. |
| 3971 | T2.11.glcm.ASM.2.range. |
| 3972 | T2.11.glcm.ASM.3.mean. |
| 3973 | T2.11.glcm.ASM.3.range. |
| 3974 | T2.11.glcm.ASM.4.mean. |
| 3975 | T2.11.glcm.ASM.4.range. |
| 3976 | T2.15.glcm.contrast.1.mean. |
| 3977 | T2.15.glcm.contrast.1.range. |
| 3978 | T2.15.glcm.contrast.2.mean. |
| 3979 | T2.15.glcm.contrast.2.range. |
| 3980 | T2.15.glcm.contrast.3.mean. |
| 3981 | T2.15.glcm.contrast.3.range. |
| 3982 | T2.15.glcm.contrast.4.mean. |
| 3983 | T2.15.glcm.contrast.4.range. |
| 3984 | T2.15.glcm.dissimilarity.1.mean. |
| 3985 | T2.15.glcm.dissimilarity.1.range. |
| 3986 | T2.15.glcm.dissimilarity.2.mean. |
| 3987 | T2.15.glcm.dissimilarity.2.range. |
| 3988 | T2.15.glcm.dissimilarity.3.mean. |
| 3989 | T2.15.glcm.dissimilarity.3.range. |
| 3990 | T2.15.glcm.dissimilarity.4.mean. |
| 3991 | T2.15.glcm.dissimilarity.4.range. |
| 3992 | T2.15.glcm.homogeneity.1.mean. |
| 3993 | T2.15.glcm.homogeneity.1.range. |
| 3994 | T2.15.glcm.homogeneity.2.mean. |
| 3995 | T2.15.glcm.homogeneity.2.range. |
| 3996 | T2.15.glcm.homogeneity.3.mean. |
| 3997 | T2.15.glcm.homogeneity.3.range. |
| 3998 | T2.15.glcm.homogeneity.4.mean. |
| 3999 | T2.15.glcm.homogeneity.4.range. |
| 4000 | T2.15.glcm.energy.1.mean. |
| 4001 | T2.15.glcm.energy.1.range. |
| 4002 | T2.15.glcm.energy.2.mean. |
| 4003 | T2.15.glcm.energy.2.range. |
| 4004 | T2.15.glcm.energy.3.mean. |
| 4005 | T2.15.glcm.energy.3.range. |
| 4006 | T2.15.glcm.energy.4.mean. |
| 4007 | T2.15.glcm.energy.4.range. |
| 4008 | T2.15.glcm.correlation.1.mean. |
| 4009 | T2.15.glcm.correlation.1.range. |
| 4010 | T2.15.glcm.correlation.2.mean. |
| 4011 | T2.15.glcm.correlation.2.range. |
| 4012 | T2.15.glcm.correlation.3.mean. |
| 4013 | T2.15.glcm.correlation.3.range. |
| 4014 | T2.15.glcm.correlation.4.mean. |
| 4015 | T2.15.glcm.correlation.4.range. |
| 4016 | T2.15.glcm.ASM.1.mean. |
| 4017 | T2.15.glcm.ASM.1.range. |
| 4018 | T2.15.glcm.ASM.2.mean. |
| 4019 | T2.15.glcm.ASM.2.range. |
| 4020 | T2.15.glcm.ASM.3.mean. |
| 4021 | T2.15.glcm.ASM.3.range. |
| 4022 | T2.15.glcm.ASM.4.mean. |
| 4023 | T2.15.glcm.ASM.4.range. |
| 4024 | T2.19.glcm.contrast.1.mean. |
| 4025 | T2.19.glcm.contrast.1.range. |
| 4026 | T2.19.glcm.contrast.2.mean. |
| 4027 | T2.19.glcm.contrast.2.range. |
| 4028 | T2.19.glcm.contrast.3.mean. |
| 4029 | T2.19.glcm.contrast.3.range. |
| 4030 | T2.19.glcm.contrast.4.mean. |
| 4031 | T2.19.glcm.contrast.4.range. |
| 4032 | T2.19.glcm.dissimilarity.1.mean. |
| 4033 | T2.19.glcm.dissimilarity.1.range. |
| 4034 | T2.19.glcm.dissimilarity.2.mean. |
| 4035 | T2.19.glcm.dissimilarity.2.range. |
| 4036 | T2.19.glcm.dissimilarity.3.mean. |
| 4037 | T2.19.glcm.dissimilarity.3.range. |
| 4038 | T2.19.glcm.dissimilarity.4.mean. |
| 4039 | T2.19.glcm.dissimilarity.4.range. |
| 4040 | T2.19.glcm.homogeneity.1.mean. |
| 4041 | T2.19.glcm.homogeneity.1.range. |
| 4042 | T2.19.glcm.homogeneity.2.mean. |
| 4043 | T2.19.glcm.homogeneity.2.range. |
| 4044 | T2.19.glcm.homogeneity.3.mean. |
| 4045 | T2.19.glcm.homogeneity.3.range. |
| 4046 | T2.19.glcm.homogeneity.4.mean. |
| 4047 | T2.19.glcm.homogeneity.4.range. |
| 4048 | T2.19.glcm.energy.1.mean. |
| 4049 | T2.19.glcm.energy.1.range. |
| 4050 | T2.19.glcm.energy.2.mean. |
| 4051 | T2.19.glcm.energy.2.range. |
| 4052 | T2.19.glcm.energy.3.mean. |
| 4053 | T2.19.glcm.energy.3.range. |
| 4054 | T2.19.glcm.energy.4.mean. |
| 4055 | T2.19.glcm.energy.4.range. |
| 4056 | T2.19.glcm.correlation.1.mean. |
| 4057 | T2.19.glcm.correlation.1.range. |
| 4058 | T2.19.glcm.correlation.2.mean. |
| 4059 | T2.19.glcm.correlation.2.range. |
| 4060 | T2.19.glcm.correlation.3.mean. |
| 4061 | T2.19.glcm.correlation.3.range. |
| 4062 | T2.19.glcm.correlation.4.mean. |
| 4063 | T2.19.glcm.correlation.4.range. |
| 4064 | T2.19.glcm.ASM.1.mean. |
| 4065 | T2.19.glcm.ASM.1.range. |
| 4066 | T2.19.glcm.ASM.2.mean. |
| 4067 | T2.19.glcm.ASM.2.range. |
| 4068 | T2.19.glcm.ASM.3.mean. |
| 4069 | T2.19.glcm.ASM.3.range. |
| 4070 | T2.19.glcm.ASM.4.mean. |
| 4071 | T2.19.glcm.ASM.4.range. |
| 4072 | T2.23.glcm.contrast.1.mean. |
| 4073 | T2.23.glcm.contrast.1.range. |
| 4074 | T2.23.glcm.contrast.2.mean. |
| 4075 | T2.23.glcm.contrast.2.range. |
| 4076 | T2.23.glcm.contrast.3.mean. |
| 4077 | T2.23.glcm.contrast.3.range. |
| 4078 | T2.23.glcm.contrast.4.mean. |
| 4079 | T2.23.glcm.contrast.4.range. |
| 4080 | T2.23.glcm.dissimilarity.1.mean. |
| 4081 | T2.23.glcm.dissimilarity.1.range. |
| 4082 | T2.23.glcm.dissimilarity.2.mean. |
| 4083 | T2.23.glcm.dissimilarity.2.range. |
| 4084 | T2.23.glcm.dissimilarity.3.mean. |
| 4085 | T2.23.glcm.dissimilarity.3.range. |
| 4086 | T2.23.glcm.dissimilarity.4.mean. |
| 4087 | T2.23.glcm.dissimilarity.4.range. |
| 4088 | T2.23.glcm.homogeneity.1.mean. |
| 4089 | T2.23.glcm.homogeneity.1.range. |
| 4090 | T2.23.glcm.homogeneity.2.mean. |
| 4091 | T2.23.glcm.homogeneity.2.range. |
| 4092 | T2.23.glcm.homogeneity.3.mean. |
| 4093 | T2.23.glcm.homogeneity.3.range. |
| 4094 | T2.23.glcm.homogeneity.4.mean. |
| 4095 | T2.23.glcm.homogeneity.4.range. |
| 4096 | T2.23.glcm.energy.1.mean. |
| 4097 | T2.23.glcm.energy.1.range. |
| 4098 | T2.23.glcm.energy.2.mean. |
| 4099 | T2.23.glcm.energy.2.range. |
| 4100 | T2.23.glcm.energy.3.mean. |
| 4101 | T2.23.glcm.energy.3.range. |
| 4102 | T2.23.glcm.energy.4.mean. |
| 4103 | T2.23.glcm.energy.4.range. |
| 4104 | T2.23.glcm.correlation.1.mean. |
| 4105 | T2.23.glcm.correlation.1.range. |
| 4106 | T2.23.glcm.correlation.2.mean. |
| 4107 | T2.23.glcm.correlation.2.range. |
| 4108 | T2.23.glcm.correlation.3.mean. |
| 4109 | T2.23.glcm.correlation.3.range. |
| 4110 | T2.23.glcm.correlation.4.mean. |
| 4111 | T2.23.glcm.correlation.4.range. |
| 4112 | T2.23.glcm.ASM.1.mean. |
| 4113 | T2.23.glcm.ASM.1.range. |
| 4114 | T2.23.glcm.ASM.2.mean. |
| 4115 | T2.23.glcm.ASM.2.range. |
| 4116 | T2.23.glcm.ASM.3.mean. |
| 4117 | T2.23.glcm.ASM.3.range. |
| 4118 | T2.23.glcm.ASM.4.mean. |
| 4119 | T2.23.glcm.ASM.4.range. |
| 4120 | T2.27.glcm.contrast.1.mean. |
| 4121 | T2.27.glcm.contrast.1.range. |
| 4122 | T2.27.glcm.contrast.2.mean. |
| 4123 | T2.27.glcm.contrast.2.range. |
| 4124 | T2.27.glcm.contrast.3.mean. |
| 4125 | T2.27.glcm.contrast.3.range. |
| 4126 | T2.27.glcm.contrast.4.mean. |
| 4127 | T2.27.glcm.contrast.4.range. |
| 4128 | T2.27.glcm.dissimilarity.1.mean. |
| 4129 | T2.27.glcm.dissimilarity.1.range. |
| 4130 | T2.27.glcm.dissimilarity.2.mean. |
| 4131 | T2.27.glcm.dissimilarity.2.range. |
| 4132 | T2.27.glcm.dissimilarity.3.mean. |
| 4133 | T2.27.glcm.dissimilarity.3.range. |
| 4134 | T2.27.glcm.dissimilarity.4.mean. |
| 4135 | T2.27.glcm.dissimilarity.4.range. |
| 4136 | T2.27.glcm.homogeneity.1.mean. |
| 4137 | T2.27.glcm.homogeneity.1.range. |
| 4138 | T2.27.glcm.homogeneity.2.mean. |
| 4139 | T2.27.glcm.homogeneity.2.range. |
| 4140 | T2.27.glcm.homogeneity.3.mean. |
| 4141 | T2.27.glcm.homogeneity.3.range. |
| 4142 | T2.27.glcm.homogeneity.4.mean. |
| 4143 | T2.27.glcm.homogeneity.4.range. |
| 4144 | T2.27.glcm.energy.1.mean. |
| 4145 | T2.27.glcm.energy.1.range. |
| 4146 | T2.27.glcm.energy.2.mean. |
| 4147 | T2.27.glcm.energy.2.range. |
| 4148 | T2.27.glcm.energy.3.mean. |
| 4149 | T2.27.glcm.energy.3.range. |
| 4150 | T2.27.glcm.energy.4.mean. |
| 4151 | T2.27.glcm.energy.4.range. |
| 4152 | T2.27.glcm.correlation.1.mean. |
| 4153 | T2.27.glcm.correlation.1.range. |
| 4154 | T2.27.glcm.correlation.2.mean. |
| 4155 | T2.27.glcm.correlation.2.range. |
| 4156 | T2.27.glcm.correlation.3.mean. |
| 4157 | T2.27.glcm.correlation.3.range. |
| 4158 | T2.27.glcm.correlation.4.mean. |
| 4159 | T2.27.glcm.correlation.4.range. |
| 4160 | T2.27.glcm.ASM.1.mean. |
| 4161 | T2.27.glcm.ASM.1.range. |
| 4162 | T2.27.glcm.ASM.2.mean. |
| 4163 | T2.27.glcm.ASM.2.range. |
| 4164 | T2.27.glcm.ASM.3.mean. |
| 4165 | T2.27.glcm.ASM.3.range. |
| 4166 | T2.27.glcm.ASM.4.mean. |
| 4167 | T2.27.glcm.ASM.4.range. |
| 4168 | T2.31.glcm.contrast.1.mean. |
| 4169 | T2.31.glcm.contrast.1.range. |
| 4170 | T2.31.glcm.contrast.2.mean. |
| 4171 | T2.31.glcm.contrast.2.range. |
| 4172 | T2.31.glcm.contrast.3.mean. |
| 4173 | T2.31.glcm.contrast.3.range. |
| 4174 | T2.31.glcm.contrast.4.mean. |
| 4175 | T2.31.glcm.contrast.4.range. |
| 4176 | T2.31.glcm.dissimilarity.1.mean. |
| 4177 | T2.31.glcm.dissimilarity.1.range. |
| 4178 | T2.31.glcm.dissimilarity.2.mean. |
| 4179 | T2.31.glcm.dissimilarity.2.range. |
| 4180 | T2.31.glcm.dissimilarity.3.mean. |
| 4181 | T2.31.glcm.dissimilarity.3.range. |
| 4182 | T2.31.glcm.dissimilarity.4.mean. |
| 4183 | T2.31.glcm.dissimilarity.4.range. |
| 4184 | T2.31.glcm.homogeneity.1.mean. |
| 4185 | T2.31.glcm.homogeneity.1.range. |
| 4186 | T2.31.glcm.homogeneity.2.mean. |
| 4187 | T2.31.glcm.homogeneity.2.range. |
| 4188 | T2.31.glcm.homogeneity.3.mean. |
| 4189 | T2.31.glcm.homogeneity.3.range. |
| 4190 | T2.31.glcm.homogeneity.4.mean. |
| 4191 | T2.31.glcm.homogeneity.4.range. |
| 4192 | T2.31.glcm.energy.1.mean. |
| 4193 | T2.31.glcm.energy.1.range. |
| 4194 | T2.31.glcm.energy.2.mean. |
| 4195 | T2.31.glcm.energy.2.range. |
| 4196 | T2.31.glcm.energy.3.mean. |
| 4197 | T2.31.glcm.energy.3.range. |
| 4198 | T2.31.glcm.energy.4.mean. |
| 4199 | T2.31.glcm.energy.4.range. |
| 4200 | T2.31.glcm.correlation.1.mean. |
| 4201 | T2.31.glcm.correlation.1.range. |
| 4202 | T2.31.glcm.correlation.2.mean. |
| 4203 | T2.31.glcm.correlation.2.range. |
| 4204 | T2.31.glcm.correlation.3.mean. |
| 4205 | T2.31.glcm.correlation.3.range. |
| 4206 | T2.31.glcm.correlation.4.mean. |
| 4207 | T2.31.glcm.correlation.4.range. |
| 4208 | T2.31.glcm.ASM.1.mean. |
| 4209 | T2.31.glcm.ASM.1.range. |
| 4210 | T2.31.glcm.ASM.2.mean. |
| 4211 | T2.31.glcm.ASM.2.range. |
| 4212 | T2.31.glcm.ASM.3.mean. |
| 4213 | T2.31.glcm.ASM.3.range. |
| 4214 | T2.31.glcm.ASM.4.mean. |
| 4215 | T2.31.glcm.ASM.4.range. |
| 4216 | T2.35.glcm.contrast.1.mean. |
| 4217 | T2.35.glcm.contrast.1.range. |
| 4218 | T2.35.glcm.contrast.2.mean. |
| 4219 | T2.35.glcm.contrast.2.range. |
| 4220 | T2.35.glcm.contrast.3.mean. |
| 4221 | T2.35.glcm.contrast.3.range. |
| 4222 | T2.35.glcm.contrast.4.mean. |
| 4223 | T2.35.glcm.contrast.4.range. |
| 4224 | T2.35.glcm.dissimilarity.1.mean. |
| 4225 | T2.35.glcm.dissimilarity.1.range. |
| 4226 | T2.35.glcm.dissimilarity.2.mean. |
| 4227 | T2.35.glcm.dissimilarity.2.range. |
| 4228 | T2.35.glcm.dissimilarity.3.mean. |
| 4229 | T2.35.glcm.dissimilarity.3.range. |
| 4230 | T2.35.glcm.dissimilarity.4.mean. |
| 4231 | T2.35.glcm.dissimilarity.4.range. |
| 4232 | T2.35.glcm.homogeneity.1.mean. |
| 4233 | T2.35.glcm.homogeneity.1.range. |
| 4234 | T2.35.glcm.homogeneity.2.mean. |
| 4235 | T2.35.glcm.homogeneity.2.range. |
| 4236 | T2.35.glcm.homogeneity.3.mean. |
| 4237 | T2.35.glcm.homogeneity.3.range. |
| 4238 | T2.35.glcm.homogeneity.4.mean. |
| 4239 | T2.35.glcm.homogeneity.4.range. |
| 4240 | T2.35.glcm.energy.1.mean. |
| 4241 | T2.35.glcm.energy.1.range. |
| 4242 | T2.35.glcm.energy.2.mean. |
| 4243 | T2.35.glcm.energy.2.range. |
| 4244 | T2.35.glcm.energy.3.mean. |
| 4245 | T2.35.glcm.energy.3.range. |
| 4246 | T2.35.glcm.energy.4.mean. |
| 4247 | T2.35.glcm.energy.4.range. |
| 4248 | T2.35.glcm.correlation.1.mean. |
| 4249 | T2.35.glcm.correlation.1.range. |
| 4250 | T2.35.glcm.correlation.2.mean. |
| 4251 | T2.35.glcm.correlation.2.range. |
| 4252 | T2.35.glcm.correlation.3.mean. |
| 4253 | T2.35.glcm.correlation.3.range. |
| 4254 | T2.35.glcm.correlation.4.mean. |
| 4255 | T2.35.glcm.correlation.4.range. |
| 4256 | T2.35.glcm.ASM.1.mean. |
| 4257 | T2.35.glcm.ASM.1.range. |
| 4258 | T2.35.glcm.ASM.2.mean. |
| 4259 | T2.35.glcm.ASM.2.range. |
| 4260 | T2.35.glcm.ASM.3.mean. |
| 4261 | T2.35.glcm.ASM.3.range. |
| 4262 | T2.35.glcm.ASM.4.mean. |
| 4263 | T2.35.glcm.ASM.4.range. |
| 4264 | T2.mbb.glcm.contrast.1.mean. |
| 4265 | T2.mbb.glcm.contrast.1.range. |
| 4266 | T2.mbb.glcm.contrast.2.mean. |
| 4267 | T2.mbb.glcm.contrast.2.range. |
| 4268 | T2.mbb.glcm.contrast.3.mean. |
| 4269 | T2.mbb.glcm.contrast.3.range. |
| 4270 | T2.mbb.glcm.contrast.4.mean. |
| 4271 | T2.mbb.glcm.contrast.4.range. |
| 4272 | T2.mbb.glcm.dissimilarity.1.mean. |
| 4273 | T2.mbb.glcm.dissimilarity.1.range. |
| 4274 | T2.mbb.glcm.dissimilarity.2.mean. |
| 4275 | T2.mbb.glcm.dissimilarity.2.range. |
| 4276 | T2.mbb.glcm.dissimilarity.3.mean. |
| 4277 | T2.mbb.glcm.dissimilarity.3.range. |
| 4278 | T2.mbb.glcm.dissimilarity.4.mean. |
| 4279 | T2.mbb.glcm.dissimilarity.4.range. |
| 4280 | T2.mbb.glcm.homogeneity.1.mean. |
| 4281 | T2.mbb.glcm.homogeneity.1.range. |
| 4282 | T2.mbb.glcm.homogeneity.2.mean. |
| 4283 | T2.mbb.glcm.homogeneity.2.range. |
| 4284 | T2.mbb.glcm.homogeneity.3.mean. |
| 4285 | T2.mbb.glcm.homogeneity.3.range. |
| 4286 | T2.mbb.glcm.homogeneity.4.mean. |
| 4287 | T2.mbb.glcm.homogeneity.4.range. |
| 4288 | T2.mbb.glcm.energy.1.mean. |
| 4289 | T2.mbb.glcm.energy.1.range. |
| 4290 | T2.mbb.glcm.energy.2.mean. |
| 4291 | T2.mbb.glcm.energy.2.range. |
| 4292 | T2.mbb.glcm.energy.3.mean. |
| 4293 | T2.mbb.glcm.energy.3.range. |
| 4294 | T2.mbb.glcm.energy.4.mean. |
| 4295 | T2.mbb.glcm.energy.4.range. |
| 4296 | T2.mbb.glcm.correlation.1.mean. |
| 4297 | T2.mbb.glcm.correlation.1.range. |
| 4298 | T2.mbb.glcm.correlation.2.mean. |
| 4299 | T2.mbb.glcm.correlation.2.range. |
| 4300 | T2.mbb.glcm.correlation.3.mean. |
| 4301 | T2.mbb.glcm.correlation.3.range. |
| 4302 | T2.mbb.glcm.correlation.4.mean. |
| 4303 | T2.mbb.glcm.correlation.4.range. |
| 4304 | T2.mbb.glcm.ASM.1.mean. |
| 4305 | T2.mbb.glcm.ASM.1.range. |
| 4306 | T2.mbb.glcm.ASM.2.mean. |
| 4307 | T2.mbb.glcm.ASM.2.range. |
| 4308 | T2.mbb.glcm.ASM.3.mean. |
| 4309 | T2.mbb.glcm.ASM.3.range. |
| 4310 | T2.mbb.glcm.ASM.4.mean. |
| 4311 | T2.mbb.glcm.ASM.4.range. |
| 4312 | T2.3.lbp.1.0. |
| 4313 | T2.3.lbp.1.1. |
| 4314 | T2.3.lbp.1.2. |
| 4315 | T2.3.lbp.1.3. |
| 4316 | T2.3.lbp.1.4. |
| 4317 | T2.3.lbp.1.5. |
| 4318 | T2.3.lbp.1.6. |
| 4319 | T2.3.lbp.1.7. |
| 4320 | T2.3.lbp.1.8. |
| 4321 | T2.3.lbp.1.9. |
| 4322 | T2.7.lbp.3.0. |
| 4323 | T2.7.lbp.3.1. |
| 4324 | T2.7.lbp.3.2. |
| 4325 | T2.7.lbp.3.3. |
| 4326 | T2.7.lbp.3.4. |
| 4327 | T2.7.lbp.3.5. |
| 4328 | T2.7.lbp.3.6. |
| 4329 | T2.7.lbp.3.7. |
| 4330 | T2.7.lbp.3.8. |
| 4331 | T2.7.lbp.3.9. |
| 4332 | T2.11.lbp.5.0. |
| 4333 | T2.11.lbp.5.1. |
| 4334 | T2.11.lbp.5.2. |
| 4335 | T2.11.lbp.5.3. |
| 4336 | T2.11.lbp.5.4. |
| 4337 | T2.11.lbp.5.5. |
| 4338 | T2.11.lbp.5.6. |
| 4339 | T2.11.lbp.5.7. |
| 4340 | T2.11.lbp.5.8. |
| 4341 | T2.11.lbp.5.9. |
| 4342 | T2.15.lbp.7.0. |
| 4343 | T2.15.lbp.7.1. |
| 4344 | T2.15.lbp.7.2. |
| 4345 | T2.15.lbp.7.3. |
| 4346 | T2.15.lbp.7.4. |
| 4347 | T2.15.lbp.7.5. |
| 4348 | T2.15.lbp.7.6. |
| 4349 | T2.15.lbp.7.7. |
| 4350 | T2.15.lbp.7.8. |
| 4351 | T2.15.lbp.7.9. |
| 4352 | T2.19.lbp.9.0. |
| 4353 | T2.19.lbp.9.1. |
| 4354 | T2.19.lbp.9.2. |
| 4355 | T2.19.lbp.9.3. |
| 4356 | T2.19.lbp.9.4. |
| 4357 | T2.19.lbp.9.5. |
| 4358 | T2.19.lbp.9.6. |
| 4359 | T2.19.lbp.9.7. |
| 4360 | T2.19.lbp.9.8. |
| 4361 | T2.19.lbp.9.9. |
| 4362 | T2.23.lbp.11.0. |
| 4363 | T2.23.lbp.11.1. |
| 4364 | T2.23.lbp.11.2. |
| 4365 | T2.23.lbp.11.3. |
| 4366 | T2.23.lbp.11.4. |
| 4367 | T2.23.lbp.11.5. |
| 4368 | T2.23.lbp.11.6. |
| 4369 | T2.23.lbp.11.7. |
| 4370 | T2.23.lbp.11.8. |
| 4371 | T2.23.lbp.11.9. |
| 4372 | T2.27.lbp.13.0. |
| 4373 | T2.27.lbp.13.1. |
| 4374 | T2.27.lbp.13.2. |
| 4375 | T2.27.lbp.13.3. |
| 4376 | T2.27.lbp.13.4. |
| 4377 | T2.27.lbp.13.5. |
| 4378 | T2.27.lbp.13.6. |
| 4379 | T2.27.lbp.13.7. |
| 4380 | T2.27.lbp.13.8. |
| 4381 | T2.27.lbp.13.9. |
| 4382 | T2.31.lbp.15.0. |
| 4383 | T2.31.lbp.15.1. |
| 4384 | T2.31.lbp.15.2. |
| 4385 | T2.31.lbp.15.3. |
| 4386 | T2.31.lbp.15.4. |
| 4387 | T2.31.lbp.15.5. |
| 4388 | T2.31.lbp.15.6. |
| 4389 | T2.31.lbp.15.7. |
| 4390 | T2.31.lbp.15.8. |
| 4391 | T2.31.lbp.15.9. |
| 4392 | T2.35.lbp.17.0. |
| 4393 | T2.35.lbp.17.1. |
| 4394 | T2.35.lbp.17.2. |
| 4395 | T2.35.lbp.17.3. |
| 4396 | T2.35.lbp.17.4. |
| 4397 | T2.35.lbp.17.5. |
| 4398 | T2.35.lbp.17.6. |
| 4399 | T2.35.lbp.17.7. |
| 4400 | T2.35.lbp.17.8. |
| 4401 | T2.35.lbp.17.9. |
| 4402 | T2.3.hog |
| 4403 | T2.7.hog |
| 4404 | T2.11.hog |
| 4405 | T2.15.hog |
| 4406 | T2.19.hog |
| 4407 | T2.23.hog |
| 4408 | T2.27.hog |
| 4409 | T2.31.hog |
| 4410 | T2.35.hog |
| 4411 | T2.3.gabor.1.0.1.mean. |
| 4412 | T2.3.gabor.1.0.1.var. |
| 4413 | T2.3.gabor.1.0.1.absmean. |
| 4414 | T2.3.gabor.1.0.1.mag. |
| 4415 | T2.3.gabor.1.0.2.mean. |
| 4416 | T2.3.gabor.1.0.2.var. |
| 4417 | T2.3.gabor.1.0.2.absmean. |
| 4418 | T2.3.gabor.1.0.2.mag. |
| 4419 | T2.3.gabor.1.0.3.mean. |
| 4420 | T2.3.gabor.1.0.3.var. |
| 4421 | T2.3.gabor.1.0.3.absmean. |
| 4422 | T2.3.gabor.1.0.3.mag. |
| 4423 | T2.3.gabor.1.0.4.mean. |
| 4424 | T2.3.gabor.1.0.4.var. |
| 4425 | T2.3.gabor.1.0.4.absmean. |
| 4426 | T2.3.gabor.1.0.4.mag. |
| 4427 | T2.3.gabor.1.0.5.mean. |
| 4428 | T2.3.gabor.1.0.5.var. |
| 4429 | T2.3.gabor.1.0.5.absmean. |
| 4430 | T2.3.gabor.1.0.5.mag. |
| 4431 | T2.3.gabor.2.0.1.mean. |
| 4432 | T2.3.gabor.2.0.1.var. |
| 4433 | T2.3.gabor.2.0.1.absmean. |
| 4434 | T2.3.gabor.2.0.1.mag. |
| 4435 | T2.3.gabor.2.0.2.mean. |
| 4436 | T2.3.gabor.2.0.2.var. |
| 4437 | T2.3.gabor.2.0.2.absmean. |
| 4438 | T2.3.gabor.2.0.2.mag. |
| 4439 | T2.3.gabor.2.0.3.mean. |
| 4440 | T2.3.gabor.2.0.3.var. |
| 4441 | T2.3.gabor.2.0.3.absmean. |
| 4442 | T2.3.gabor.2.0.3.mag. |
| 4443 | T2.3.gabor.2.0.4.mean. |
| 4444 | T2.3.gabor.2.0.4.var. |
| 4445 | T2.3.gabor.2.0.4.absmean. |
| 4446 | T2.3.gabor.2.0.4.mag. |
| 4447 | T2.3.gabor.2.0.5.mean. |
| 4448 | T2.3.gabor.2.0.5.var. |
| 4449 | T2.3.gabor.2.0.5.absmean. |
| 4450 | T2.3.gabor.2.0.5.mag. |
| 4451 | T2.3.gabor.3.0.1.mean. |
| 4452 | T2.3.gabor.3.0.1.var. |
| 4453 | T2.3.gabor.3.0.1.absmean. |
| 4454 | T2.3.gabor.3.0.1.mag. |
| 4455 | T2.3.gabor.3.0.2.mean. |
| 4456 | T2.3.gabor.3.0.2.var. |
| 4457 | T2.3.gabor.3.0.2.absmean. |
| 4458 | T2.3.gabor.3.0.2.mag. |
| 4459 | T2.3.gabor.3.0.3.mean. |
| 4460 | T2.3.gabor.3.0.3.var. |
| 4461 | T2.3.gabor.3.0.3.absmean. |
| 4462 | T2.3.gabor.3.0.3.mag. |
| 4463 | T2.3.gabor.3.0.4.mean. |
| 4464 | T2.3.gabor.3.0.4.var. |
| 4465 | T2.3.gabor.3.0.4.absmean. |
| 4466 | T2.3.gabor.3.0.4.mag. |
| 4467 | T2.3.gabor.3.0.5.mean. |
| 4468 | T2.3.gabor.3.0.5.var. |
| 4469 | T2.3.gabor.3.0.5.absmean. |
| 4470 | T2.3.gabor.3.0.5.mag. |
| 4471 | T2.7.gabor.1.0.1.mean. |
| 4472 | T2.7.gabor.1.0.1.var. |
| 4473 | T2.7.gabor.1.0.1.absmean. |
| 4474 | T2.7.gabor.1.0.1.mag. |
| 4475 | T2.7.gabor.1.0.2.mean. |
| 4476 | T2.7.gabor.1.0.2.var. |
| 4477 | T2.7.gabor.1.0.2.absmean. |
| 4478 | T2.7.gabor.1.0.2.mag. |
| 4479 | T2.7.gabor.1.0.3.mean. |
| 4480 | T2.7.gabor.1.0.3.var. |
| 4481 | T2.7.gabor.1.0.3.absmean. |
| 4482 | T2.7.gabor.1.0.3.mag. |
| 4483 | T2.7.gabor.1.0.4.mean. |
| 4484 | T2.7.gabor.1.0.4.var. |
| 4485 | T2.7.gabor.1.0.4.absmean. |
| 4486 | T2.7.gabor.1.0.4.mag. |
| 4487 | T2.7.gabor.1.0.5.mean. |
| 4488 | T2.7.gabor.1.0.5.var. |
| 4489 | T2.7.gabor.1.0.5.absmean. |
| 4490 | T2.7.gabor.1.0.5.mag. |
| 4491 | T2.7.gabor.2.0.1.mean. |
| 4492 | T2.7.gabor.2.0.1.var. |
| 4493 | T2.7.gabor.2.0.1.absmean. |
| 4494 | T2.7.gabor.2.0.1.mag. |
| 4495 | T2.7.gabor.2.0.2.mean. |
| 4496 | T2.7.gabor.2.0.2.var. |
| 4497 | T2.7.gabor.2.0.2.absmean. |
| 4498 | T2.7.gabor.2.0.2.mag. |
| 4499 | T2.7.gabor.2.0.3.mean. |
| 4500 | T2.7.gabor.2.0.3.var. |
| 4501 | T2.7.gabor.2.0.3.absmean. |
| 4502 | T2.7.gabor.2.0.3.mag. |
| 4503 | T2.7.gabor.2.0.4.mean. |
| 4504 | T2.7.gabor.2.0.4.var. |
| 4505 | T2.7.gabor.2.0.4.absmean. |
| 4506 | T2.7.gabor.2.0.4.mag. |
| 4507 | T2.7.gabor.2.0.5.mean. |
| 4508 | T2.7.gabor.2.0.5.var. |
| 4509 | T2.7.gabor.2.0.5.absmean. |
| 4510 | T2.7.gabor.2.0.5.mag. |
| 4511 | T2.7.gabor.3.0.1.mean. |
| 4512 | T2.7.gabor.3.0.1.var. |
| 4513 | T2.7.gabor.3.0.1.absmean. |
| 4514 | T2.7.gabor.3.0.1.mag. |
| 4515 | T2.7.gabor.3.0.2.mean. |
| 4516 | T2.7.gabor.3.0.2.var. |
| 4517 | T2.7.gabor.3.0.2.absmean. |
| 4518 | T2.7.gabor.3.0.2.mag. |
| 4519 | T2.7.gabor.3.0.3.mean. |
| 4520 | T2.7.gabor.3.0.3.var. |
| 4521 | T2.7.gabor.3.0.3.absmean. |
| 4522 | T2.7.gabor.3.0.3.mag. |
| 4523 | T2.7.gabor.3.0.4.mean. |
| 4524 | T2.7.gabor.3.0.4.var. |
| 4525 | T2.7.gabor.3.0.4.absmean. |
| 4526 | T2.7.gabor.3.0.4.mag. |
| 4527 | T2.7.gabor.3.0.5.mean. |
| 4528 | T2.7.gabor.3.0.5.var. |
| 4529 | T2.7.gabor.3.0.5.absmean. |
| 4530 | T2.7.gabor.3.0.5.mag. |
| 4531 | T2.11.gabor.1.0.1.mean. |
| 4532 | T2.11.gabor.1.0.1.var. |
| 4533 | T2.11.gabor.1.0.1.absmean. |
| 4534 | T2.11.gabor.1.0.1.mag. |
| 4535 | T2.11.gabor.1.0.2.mean. |
| 4536 | T2.11.gabor.1.0.2.var. |
| 4537 | T2.11.gabor.1.0.2.absmean. |
| 4538 | T2.11.gabor.1.0.2.mag. |
| 4539 | T2.11.gabor.1.0.3.mean. |
| 4540 | T2.11.gabor.1.0.3.var. |
| 4541 | T2.11.gabor.1.0.3.absmean. |
| 4542 | T2.11.gabor.1.0.3.mag. |
| 4543 | T2.11.gabor.1.0.4.mean. |
| 4544 | T2.11.gabor.1.0.4.var. |
| 4545 | T2.11.gabor.1.0.4.absmean. |
| 4546 | T2.11.gabor.1.0.4.mag. |
| 4547 | T2.11.gabor.1.0.5.mean. |
| 4548 | T2.11.gabor.1.0.5.var. |
| 4549 | T2.11.gabor.1.0.5.absmean. |
| 4550 | T2.11.gabor.1.0.5.mag. |
| 4551 | T2.11.gabor.2.0.1.mean. |
| 4552 | T2.11.gabor.2.0.1.var. |
| 4553 | T2.11.gabor.2.0.1.absmean. |
| 4554 | T2.11.gabor.2.0.1.mag. |
| 4555 | T2.11.gabor.2.0.2.mean. |
| 4556 | T2.11.gabor.2.0.2.var. |
| 4557 | T2.11.gabor.2.0.2.absmean. |
| 4558 | T2.11.gabor.2.0.2.mag. |
| 4559 | T2.11.gabor.2.0.3.mean. |
| 4560 | T2.11.gabor.2.0.3.var. |
| 4561 | T2.11.gabor.2.0.3.absmean. |
| 4562 | T2.11.gabor.2.0.3.mag. |
| 4563 | T2.11.gabor.2.0.4.mean. |
| 4564 | T2.11.gabor.2.0.4.var. |
| 4565 | T2.11.gabor.2.0.4.absmean. |
| 4566 | T2.11.gabor.2.0.4.mag. |
| 4567 | T2.11.gabor.2.0.5.mean. |
| 4568 | T2.11.gabor.2.0.5.var. |
| 4569 | T2.11.gabor.2.0.5.absmean. |
| 4570 | T2.11.gabor.2.0.5.mag. |
| 4571 | T2.11.gabor.3.0.1.mean. |
| 4572 | T2.11.gabor.3.0.1.var. |
| 4573 | T2.11.gabor.3.0.1.absmean. |
| 4574 | T2.11.gabor.3.0.1.mag. |
| 4575 | T2.11.gabor.3.0.2.mean. |
| 4576 | T2.11.gabor.3.0.2.var. |
| 4577 | T2.11.gabor.3.0.2.absmean. |
| 4578 | T2.11.gabor.3.0.2.mag. |
| 4579 | T2.11.gabor.3.0.3.mean. |
| 4580 | T2.11.gabor.3.0.3.var. |
| 4581 | T2.11.gabor.3.0.3.absmean. |
| 4582 | T2.11.gabor.3.0.3.mag. |
| 4583 | T2.11.gabor.3.0.4.mean. |
| 4584 | T2.11.gabor.3.0.4.var. |
| 4585 | T2.11.gabor.3.0.4.absmean. |
| 4586 | T2.11.gabor.3.0.4.mag. |
| 4587 | T2.11.gabor.3.0.5.mean. |
| 4588 | T2.11.gabor.3.0.5.var. |
| 4589 | T2.11.gabor.3.0.5.absmean. |
| 4590 | T2.11.gabor.3.0.5.mag. |
| 4591 | T2.15.gabor.1.0.1.mean. |
| 4592 | T2.15.gabor.1.0.1.var. |
| 4593 | T2.15.gabor.1.0.1.absmean. |
| 4594 | T2.15.gabor.1.0.1.mag. |
| 4595 | T2.15.gabor.1.0.2.mean. |
| 4596 | T2.15.gabor.1.0.2.var. |
| 4597 | T2.15.gabor.1.0.2.absmean. |
| 4598 | T2.15.gabor.1.0.2.mag. |
| 4599 | T2.15.gabor.1.0.3.mean. |
| 4600 | T2.15.gabor.1.0.3.var. |
| 4601 | T2.15.gabor.1.0.3.absmean. |
| 4602 | T2.15.gabor.1.0.3.mag. |
| 4603 | T2.15.gabor.1.0.4.mean. |
| 4604 | T2.15.gabor.1.0.4.var. |
| 4605 | T2.15.gabor.1.0.4.absmean. |
| 4606 | T2.15.gabor.1.0.4.mag. |
| 4607 | T2.15.gabor.1.0.5.mean. |
| 4608 | T2.15.gabor.1.0.5.var. |
| 4609 | T2.15.gabor.1.0.5.absmean. |
| 4610 | T2.15.gabor.1.0.5.mag. |
| 4611 | T2.15.gabor.2.0.1.mean. |
| 4612 | T2.15.gabor.2.0.1.var. |
| 4613 | T2.15.gabor.2.0.1.absmean. |
| 4614 | T2.15.gabor.2.0.1.mag. |
| 4615 | T2.15.gabor.2.0.2.mean. |
| 4616 | T2.15.gabor.2.0.2.var. |
| 4617 | T2.15.gabor.2.0.2.absmean. |
| 4618 | T2.15.gabor.2.0.2.mag. |
| 4619 | T2.15.gabor.2.0.3.mean. |
| 4620 | T2.15.gabor.2.0.3.var. |
| 4621 | T2.15.gabor.2.0.3.absmean. |
| 4622 | T2.15.gabor.2.0.3.mag. |
| 4623 | T2.15.gabor.2.0.4.mean. |
| 4624 | T2.15.gabor.2.0.4.var. |
| 4625 | T2.15.gabor.2.0.4.absmean. |
| 4626 | T2.15.gabor.2.0.4.mag. |
| 4627 | T2.15.gabor.2.0.5.mean. |
| 4628 | T2.15.gabor.2.0.5.var. |
| 4629 | T2.15.gabor.2.0.5.absmean. |
| 4630 | T2.15.gabor.2.0.5.mag. |
| 4631 | T2.15.gabor.3.0.1.mean. |
| 4632 | T2.15.gabor.3.0.1.var. |
| 4633 | T2.15.gabor.3.0.1.absmean. |
| 4634 | T2.15.gabor.3.0.1.mag. |
| 4635 | T2.15.gabor.3.0.2.mean. |
| 4636 | T2.15.gabor.3.0.2.var. |
| 4637 | T2.15.gabor.3.0.2.absmean. |
| 4638 | T2.15.gabor.3.0.2.mag. |
| 4639 | T2.15.gabor.3.0.3.mean. |
| 4640 | T2.15.gabor.3.0.3.var. |
| 4641 | T2.15.gabor.3.0.3.absmean. |
| 4642 | T2.15.gabor.3.0.3.mag. |
| 4643 | T2.15.gabor.3.0.4.mean. |
| 4644 | T2.15.gabor.3.0.4.var. |
| 4645 | T2.15.gabor.3.0.4.absmean. |
| 4646 | T2.15.gabor.3.0.4.mag. |
| 4647 | T2.15.gabor.3.0.5.mean. |
| 4648 | T2.15.gabor.3.0.5.var. |
| 4649 | T2.15.gabor.3.0.5.absmean. |
| 4650 | T2.15.gabor.3.0.5.mag. |
| 4651 | T2.19.gabor.1.0.1.mean. |
| 4652 | T2.19.gabor.1.0.1.var. |
| 4653 | T2.19.gabor.1.0.1.absmean. |
| 4654 | T2.19.gabor.1.0.1.mag. |
| 4655 | T2.19.gabor.1.0.2.mean. |
| 4656 | T2.19.gabor.1.0.2.var. |
| 4657 | T2.19.gabor.1.0.2.absmean. |
| 4658 | T2.19.gabor.1.0.2.mag. |
| 4659 | T2.19.gabor.1.0.3.mean. |
| 4660 | T2.19.gabor.1.0.3.var. |
| 4661 | T2.19.gabor.1.0.3.absmean. |
| 4662 | T2.19.gabor.1.0.3.mag. |
| 4663 | T2.19.gabor.1.0.4.mean. |
| 4664 | T2.19.gabor.1.0.4.var. |
| 4665 | T2.19.gabor.1.0.4.absmean. |
| 4666 | T2.19.gabor.1.0.4.mag. |
| 4667 | T2.19.gabor.1.0.5.mean. |
| 4668 | T2.19.gabor.1.0.5.var. |
| 4669 | T2.19.gabor.1.0.5.absmean. |
| 4670 | T2.19.gabor.1.0.5.mag. |
| 4671 | T2.19.gabor.2.0.1.mean. |
| 4672 | T2.19.gabor.2.0.1.var. |
| 4673 | T2.19.gabor.2.0.1.absmean. |
| 4674 | T2.19.gabor.2.0.1.mag. |
| 4675 | T2.19.gabor.2.0.2.mean. |
| 4676 | T2.19.gabor.2.0.2.var. |
| 4677 | T2.19.gabor.2.0.2.absmean. |
| 4678 | T2.19.gabor.2.0.2.mag. |
| 4679 | T2.19.gabor.2.0.3.mean. |
| 4680 | T2.19.gabor.2.0.3.var. |
| 4681 | T2.19.gabor.2.0.3.absmean. |
| 4682 | T2.19.gabor.2.0.3.mag. |
| 4683 | T2.19.gabor.2.0.4.mean. |
| 4684 | T2.19.gabor.2.0.4.var. |
| 4685 | T2.19.gabor.2.0.4.absmean. |
| 4686 | T2.19.gabor.2.0.4.mag. |
| 4687 | T2.19.gabor.2.0.5.mean. |
| 4688 | T2.19.gabor.2.0.5.var. |
| 4689 | T2.19.gabor.2.0.5.absmean. |
| 4690 | T2.19.gabor.2.0.5.mag. |
| 4691 | T2.19.gabor.3.0.1.mean. |
| 4692 | T2.19.gabor.3.0.1.var. |
| 4693 | T2.19.gabor.3.0.1.absmean. |
| 4694 | T2.19.gabor.3.0.1.mag. |
| 4695 | T2.19.gabor.3.0.2.mean. |
| 4696 | T2.19.gabor.3.0.2.var. |
| 4697 | T2.19.gabor.3.0.2.absmean. |
| 4698 | T2.19.gabor.3.0.2.mag. |
| 4699 | T2.19.gabor.3.0.3.mean. |
| 4700 | T2.19.gabor.3.0.3.var. |
| 4701 | T2.19.gabor.3.0.3.absmean. |
| 4702 | T2.19.gabor.3.0.3.mag. |
| 4703 | T2.19.gabor.3.0.4.mean. |
| 4704 | T2.19.gabor.3.0.4.var. |
| 4705 | T2.19.gabor.3.0.4.absmean. |
| 4706 | T2.19.gabor.3.0.4.mag. |
| 4707 | T2.19.gabor.3.0.5.mean. |
| 4708 | T2.19.gabor.3.0.5.var. |
| 4709 | T2.19.gabor.3.0.5.absmean. |
| 4710 | T2.19.gabor.3.0.5.mag. |
| 4711 | T2.23.gabor.1.0.1.mean. |
| 4712 | T2.23.gabor.1.0.1.var. |
| 4713 | T2.23.gabor.1.0.1.absmean. |
| 4714 | T2.23.gabor.1.0.1.mag. |
| 4715 | T2.23.gabor.1.0.2.mean. |
| 4716 | T2.23.gabor.1.0.2.var. |
| 4717 | T2.23.gabor.1.0.2.absmean. |
| 4718 | T2.23.gabor.1.0.2.mag. |
| 4719 | T2.23.gabor.1.0.3.mean. |
| 4720 | T2.23.gabor.1.0.3.var. |
| 4721 | T2.23.gabor.1.0.3.absmean. |
| 4722 | T2.23.gabor.1.0.3.mag. |
| 4723 | T2.23.gabor.1.0.4.mean. |
| 4724 | T2.23.gabor.1.0.4.var. |
| 4725 | T2.23.gabor.1.0.4.absmean. |
| 4726 | T2.23.gabor.1.0.4.mag. |
| 4727 | T2.23.gabor.1.0.5.mean. |
| 4728 | T2.23.gabor.1.0.5.var. |
| 4729 | T2.23.gabor.1.0.5.absmean. |
| 4730 | T2.23.gabor.1.0.5.mag. |
| 4731 | T2.23.gabor.2.0.1.mean. |
| 4732 | T2.23.gabor.2.0.1.var. |
| 4733 | T2.23.gabor.2.0.1.absmean. |
| 4734 | T2.23.gabor.2.0.1.mag. |
| 4735 | T2.23.gabor.2.0.2.mean. |
| 4736 | T2.23.gabor.2.0.2.var. |
| 4737 | T2.23.gabor.2.0.2.absmean. |
| 4738 | T2.23.gabor.2.0.2.mag. |
| 4739 | T2.23.gabor.2.0.3.mean. |
| 4740 | T2.23.gabor.2.0.3.var. |
| 4741 | T2.23.gabor.2.0.3.absmean. |
| 4742 | T2.23.gabor.2.0.3.mag. |
| 4743 | T2.23.gabor.2.0.4.mean. |
| 4744 | T2.23.gabor.2.0.4.var. |
| 4745 | T2.23.gabor.2.0.4.absmean. |
| 4746 | T2.23.gabor.2.0.4.mag. |
| 4747 | T2.23.gabor.2.0.5.mean. |
| 4748 | T2.23.gabor.2.0.5.var. |
| 4749 | T2.23.gabor.2.0.5.absmean. |
| 4750 | T2.23.gabor.2.0.5.mag. |
| 4751 | T2.23.gabor.3.0.1.mean. |
| 4752 | T2.23.gabor.3.0.1.var. |
| 4753 | T2.23.gabor.3.0.1.absmean. |
| 4754 | T2.23.gabor.3.0.1.mag. |
| 4755 | T2.23.gabor.3.0.2.mean. |
| 4756 | T2.23.gabor.3.0.2.var. |
| 4757 | T2.23.gabor.3.0.2.absmean. |
| 4758 | T2.23.gabor.3.0.2.mag. |
| 4759 | T2.23.gabor.3.0.3.mean. |
| 4760 | T2.23.gabor.3.0.3.var. |
| 4761 | T2.23.gabor.3.0.3.absmean. |
| 4762 | T2.23.gabor.3.0.3.mag. |
| 4763 | T2.23.gabor.3.0.4.mean. |
| 4764 | T2.23.gabor.3.0.4.var. |
| 4765 | T2.23.gabor.3.0.4.absmean. |
| 4766 | T2.23.gabor.3.0.4.mag. |
| 4767 | T2.23.gabor.3.0.5.mean. |
| 4768 | T2.23.gabor.3.0.5.var. |
| 4769 | T2.23.gabor.3.0.5.absmean. |
| 4770 | T2.23.gabor.3.0.5.mag. |
| 4771 | T2.27.gabor.1.0.1.mean. |
| 4772 | T2.27.gabor.1.0.1.var. |
| 4773 | T2.27.gabor.1.0.1.absmean. |
| 4774 | T2.27.gabor.1.0.1.mag. |
| 4775 | T2.27.gabor.1.0.2.mean. |
| 4776 | T2.27.gabor.1.0.2.var. |
| 4777 | T2.27.gabor.1.0.2.absmean. |
| 4778 | T2.27.gabor.1.0.2.mag. |
| 4779 | T2.27.gabor.1.0.3.mean. |
| 4780 | T2.27.gabor.1.0.3.var. |
| 4781 | T2.27.gabor.1.0.3.absmean. |
| 4782 | T2.27.gabor.1.0.3.mag. |
| 4783 | T2.27.gabor.1.0.4.mean. |
| 4784 | T2.27.gabor.1.0.4.var. |
| 4785 | T2.27.gabor.1.0.4.absmean. |
| 4786 | T2.27.gabor.1.0.4.mag. |
| 4787 | T2.27.gabor.1.0.5.mean. |
| 4788 | T2.27.gabor.1.0.5.var. |
| 4789 | T2.27.gabor.1.0.5.absmean. |
| 4790 | T2.27.gabor.1.0.5.mag. |
| 4791 | T2.27.gabor.2.0.1.mean. |
| 4792 | T2.27.gabor.2.0.1.var. |
| 4793 | T2.27.gabor.2.0.1.absmean. |
| 4794 | T2.27.gabor.2.0.1.mag. |
| 4795 | T2.27.gabor.2.0.2.mean. |
| 4796 | T2.27.gabor.2.0.2.var. |
| 4797 | T2.27.gabor.2.0.2.absmean. |
| 4798 | T2.27.gabor.2.0.2.mag. |
| 4799 | T2.27.gabor.2.0.3.mean. |
| 4800 | T2.27.gabor.2.0.3.var. |
| 4801 | T2.27.gabor.2.0.3.absmean. |
| 4802 | T2.27.gabor.2.0.3.mag. |
| 4803 | T2.27.gabor.2.0.4.mean. |
| 4804 | T2.27.gabor.2.0.4.var. |
| 4805 | T2.27.gabor.2.0.4.absmean. |
| 4806 | T2.27.gabor.2.0.4.mag. |
| 4807 | T2.27.gabor.2.0.5.mean. |
| 4808 | T2.27.gabor.2.0.5.var. |
| 4809 | T2.27.gabor.2.0.5.absmean. |
| 4810 | T2.27.gabor.2.0.5.mag. |
| 4811 | T2.27.gabor.3.0.1.mean. |
| 4812 | T2.27.gabor.3.0.1.var. |
| 4813 | T2.27.gabor.3.0.1.absmean. |
| 4814 | T2.27.gabor.3.0.1.mag. |
| 4815 | T2.27.gabor.3.0.2.mean. |
| 4816 | T2.27.gabor.3.0.2.var. |
| 4817 | T2.27.gabor.3.0.2.absmean. |
| 4818 | T2.27.gabor.3.0.2.mag. |
| 4819 | T2.27.gabor.3.0.3.mean. |
| 4820 | T2.27.gabor.3.0.3.var. |
| 4821 | T2.27.gabor.3.0.3.absmean. |
| 4822 | T2.27.gabor.3.0.3.mag. |
| 4823 | T2.27.gabor.3.0.4.mean. |
| 4824 | T2.27.gabor.3.0.4.var. |
| 4825 | T2.27.gabor.3.0.4.absmean. |
| 4826 | T2.27.gabor.3.0.4.mag. |
| 4827 | T2.27.gabor.3.0.5.mean. |
| 4828 | T2.27.gabor.3.0.5.var. |
| 4829 | T2.27.gabor.3.0.5.absmean. |
| 4830 | T2.27.gabor.3.0.5.mag. |
| 4831 | T2.31.gabor.1.0.1.mean. |
| 4832 | T2.31.gabor.1.0.1.var. |
| 4833 | T2.31.gabor.1.0.1.absmean. |
| 4834 | T2.31.gabor.1.0.1.mag. |
| 4835 | T2.31.gabor.1.0.2.mean. |
| 4836 | T2.31.gabor.1.0.2.var. |
| 4837 | T2.31.gabor.1.0.2.absmean. |
| 4838 | T2.31.gabor.1.0.2.mag. |
| 4839 | T2.31.gabor.1.0.3.mean. |
| 4840 | T2.31.gabor.1.0.3.var. |
| 4841 | T2.31.gabor.1.0.3.absmean. |
| 4842 | T2.31.gabor.1.0.3.mag. |
| 4843 | T2.31.gabor.1.0.4.mean. |
| 4844 | T2.31.gabor.1.0.4.var. |
| 4845 | T2.31.gabor.1.0.4.absmean. |
| 4846 | T2.31.gabor.1.0.4.mag. |
| 4847 | T2.31.gabor.1.0.5.mean. |
| 4848 | T2.31.gabor.1.0.5.var. |
| 4849 | T2.31.gabor.1.0.5.absmean. |
| 4850 | T2.31.gabor.1.0.5.mag. |
| 4851 | T2.31.gabor.2.0.1.mean. |
| 4852 | T2.31.gabor.2.0.1.var. |
| 4853 | T2.31.gabor.2.0.1.absmean. |
| 4854 | T2.31.gabor.2.0.1.mag. |
| 4855 | T2.31.gabor.2.0.2.mean. |
| 4856 | T2.31.gabor.2.0.2.var. |
| 4857 | T2.31.gabor.2.0.2.absmean. |
| 4858 | T2.31.gabor.2.0.2.mag. |
| 4859 | T2.31.gabor.2.0.3.mean. |
| 4860 | T2.31.gabor.2.0.3.var. |
| 4861 | T2.31.gabor.2.0.3.absmean. |
| 4862 | T2.31.gabor.2.0.3.mag. |
| 4863 | T2.31.gabor.2.0.4.mean. |
| 4864 | T2.31.gabor.2.0.4.var. |
| 4865 | T2.31.gabor.2.0.4.absmean. |
| 4866 | T2.31.gabor.2.0.4.mag. |
| 4867 | T2.31.gabor.2.0.5.mean. |
| 4868 | T2.31.gabor.2.0.5.var. |
| 4869 | T2.31.gabor.2.0.5.absmean. |
| 4870 | T2.31.gabor.2.0.5.mag. |
| 4871 | T2.31.gabor.3.0.1.mean. |
| 4872 | T2.31.gabor.3.0.1.var. |
| 4873 | T2.31.gabor.3.0.1.absmean. |
| 4874 | T2.31.gabor.3.0.1.mag. |
| 4875 | T2.31.gabor.3.0.2.mean. |
| 4876 | T2.31.gabor.3.0.2.var. |
| 4877 | T2.31.gabor.3.0.2.absmean. |
| 4878 | T2.31.gabor.3.0.2.mag. |
| 4879 | T2.31.gabor.3.0.3.mean. |
| 4880 | T2.31.gabor.3.0.3.var. |
| 4881 | T2.31.gabor.3.0.3.absmean. |
| 4882 | T2.31.gabor.3.0.3.mag. |
| 4883 | T2.31.gabor.3.0.4.mean. |
| 4884 | T2.31.gabor.3.0.4.var. |
| 4885 | T2.31.gabor.3.0.4.absmean. |
| 4886 | T2.31.gabor.3.0.4.mag. |
| 4887 | T2.31.gabor.3.0.5.mean. |
| 4888 | T2.31.gabor.3.0.5.var. |
| 4889 | T2.31.gabor.3.0.5.absmean. |
| 4890 | T2.31.gabor.3.0.5.mag. |
| 4891 | T2.35.gabor.1.0.1.mean. |
| 4892 | T2.35.gabor.1.0.1.var. |
| 4893 | T2.35.gabor.1.0.1.absmean. |
| 4894 | T2.35.gabor.1.0.1.mag. |
| 4895 | T2.35.gabor.1.0.2.mean. |
| 4896 | T2.35.gabor.1.0.2.var. |
| 4897 | T2.35.gabor.1.0.2.absmean. |
| 4898 | T2.35.gabor.1.0.2.mag. |
| 4899 | T2.35.gabor.1.0.3.mean. |
| 4900 | T2.35.gabor.1.0.3.var. |
| 4901 | T2.35.gabor.1.0.3.absmean. |
| 4902 | T2.35.gabor.1.0.3.mag. |
| 4903 | T2.35.gabor.1.0.4.mean. |
| 4904 | T2.35.gabor.1.0.4.var. |
| 4905 | T2.35.gabor.1.0.4.absmean. |
| 4906 | T2.35.gabor.1.0.4.mag. |
| 4907 | T2.35.gabor.1.0.5.mean. |
| 4908 | T2.35.gabor.1.0.5.var. |
| 4909 | T2.35.gabor.1.0.5.absmean. |
| 4910 | T2.35.gabor.1.0.5.mag. |
| 4911 | T2.35.gabor.2.0.1.mean. |
| 4912 | T2.35.gabor.2.0.1.var. |
| 4913 | T2.35.gabor.2.0.1.absmean. |
| 4914 | T2.35.gabor.2.0.1.mag. |
| 4915 | T2.35.gabor.2.0.2.mean. |
| 4916 | T2.35.gabor.2.0.2.var. |
| 4917 | T2.35.gabor.2.0.2.absmean. |
| 4918 | T2.35.gabor.2.0.2.mag. |
| 4919 | T2.35.gabor.2.0.3.mean. |
| 4920 | T2.35.gabor.2.0.3.var. |
| 4921 | T2.35.gabor.2.0.3.absmean. |
| 4922 | T2.35.gabor.2.0.3.mag. |
| 4923 | T2.35.gabor.2.0.4.mean. |
| 4924 | T2.35.gabor.2.0.4.var. |
| 4925 | T2.35.gabor.2.0.4.absmean. |
| 4926 | T2.35.gabor.2.0.4.mag. |
| 4927 | T2.35.gabor.2.0.5.mean. |
| 4928 | T2.35.gabor.2.0.5.var. |
| 4929 | T2.35.gabor.2.0.5.absmean. |
| 4930 | T2.35.gabor.2.0.5.mag. |
| 4931 | T2.35.gabor.3.0.1.mean. |
| 4932 | T2.35.gabor.3.0.1.var. |
| 4933 | T2.35.gabor.3.0.1.absmean. |
| 4934 | T2.35.gabor.3.0.1.mag. |
| 4935 | T2.35.gabor.3.0.2.mean. |
| 4936 | T2.35.gabor.3.0.2.var. |
| 4937 | T2.35.gabor.3.0.2.absmean. |
| 4938 | T2.35.gabor.3.0.2.mag. |
| 4939 | T2.35.gabor.3.0.3.mean. |
| 4940 | T2.35.gabor.3.0.3.var. |
| 4941 | T2.35.gabor.3.0.3.absmean. |
| 4942 | T2.35.gabor.3.0.3.mag. |
| 4943 | T2.35.gabor.3.0.4.mean. |
| 4944 | T2.35.gabor.3.0.4.var. |
| 4945 | T2.35.gabor.3.0.4.absmean. |
| 4946 | T2.35.gabor.3.0.4.mag. |
| 4947 | T2.35.gabor.3.0.5.mean. |
| 4948 | T2.35.gabor.3.0.5.var. |
| 4949 | T2.35.gabor.3.0.5.absmean. |
| 4950 | T2.35.gabor.3.0.5.mag. |
| 4951 | T2.3.haar.1.1.aav. |
| 4952 | T2.3.haar.1.1.std. |
| 4953 | T2.3.haar.1.2.aav. |
| 4954 | T2.3.haar.1.2.std. |
| 4955 | T2.3.haar.1.3.aav. |
| 4956 | T2.3.haar.1.3.std. |
| 4957 | T2.3.haar.2.1.aav. |
| 4958 | T2.3.haar.2.1.std. |
| 4959 | T2.3.haar.2.2.aav. |
| 4960 | T2.3.haar.2.2.std. |
| 4961 | T2.3.haar.2.3.aav. |
| 4962 | T2.3.haar.2.3.std. |
| 4963 | T2.3.haar.3.1.aav. |
| 4964 | T2.3.haar.3.1.std. |
| 4965 | T2.3.haar.3.2.aav. |
| 4966 | T2.3.haar.3.2.std. |
| 4967 | T2.3.haar.3.3.aav. |
| 4968 | T2.3.haar.3.3.std. |
| 4969 | T2.3.haar.4.1.aav. |
| 4970 | T2.3.haar.4.1.std. |
| 4971 | T2.3.haar.4.2.aav. |
| 4972 | T2.3.haar.4.2.std. |
| 4973 | T2.3.haar.4.3.aav. |
| 4974 | T2.3.haar.4.3.std. |
| 4975 | T2.7.haar.1.1.aav. |
| 4976 | T2.7.haar.1.1.std. |
| 4977 | T2.7.haar.1.2.aav. |
| 4978 | T2.7.haar.1.2.std. |
| 4979 | T2.7.haar.1.3.aav. |
| 4980 | T2.7.haar.1.3.std. |
| 4981 | T2.7.haar.2.1.aav. |
| 4982 | T2.7.haar.2.1.std. |
| 4983 | T2.7.haar.2.2.aav. |
| 4984 | T2.7.haar.2.2.std. |
| 4985 | T2.7.haar.2.3.aav. |
| 4986 | T2.7.haar.2.3.std. |
| 4987 | T2.7.haar.3.1.aav. |
| 4988 | T2.7.haar.3.1.std. |
| 4989 | T2.7.haar.3.2.aav. |
| 4990 | T2.7.haar.3.2.std. |
| 4991 | T2.7.haar.3.3.aav. |
| 4992 | T2.7.haar.3.3.std. |
| 4993 | T2.7.haar.4.1.aav. |
| 4994 | T2.7.haar.4.1.std. |
| 4995 | T2.7.haar.4.2.aav. |
| 4996 | T2.7.haar.4.2.std. |
| 4997 | T2.7.haar.4.3.aav. |
| 4998 | T2.7.haar.4.3.std. |
| 4999 | T2.11.haar.1.1.aav. |
| 5000 | T2.11.haar.1.1.std. |
| 5001 | T2.11.haar.1.2.aav. |
| 5002 | T2.11.haar.1.2.std. |
| 5003 | T2.11.haar.1.3.aav. |
| 5004 | T2.11.haar.1.3.std. |
| 5005 | T2.11.haar.2.1.aav. |
| 5006 | T2.11.haar.2.1.std. |
| 5007 | T2.11.haar.2.2.aav. |
| 5008 | T2.11.haar.2.2.std. |
| 5009 | T2.11.haar.2.3.aav. |
| 5010 | T2.11.haar.2.3.std. |
| 5011 | T2.11.haar.3.1.aav. |
| 5012 | T2.11.haar.3.1.std. |
| 5013 | T2.11.haar.3.2.aav. |
| 5014 | T2.11.haar.3.2.std. |
| 5015 | T2.11.haar.3.3.aav. |
| 5016 | T2.11.haar.3.3.std. |
| 5017 | T2.11.haar.4.1.aav. |
| 5018 | T2.11.haar.4.1.std. |
| 5019 | T2.11.haar.4.2.aav. |
| 5020 | T2.11.haar.4.2.std. |
| 5021 | T2.11.haar.4.3.aav. |
| 5022 | T2.11.haar.4.3.std. |
| 5023 | T2.15.haar.1.1.aav. |
| 5024 | T2.15.haar.1.1.std. |
| 5025 | T2.15.haar.1.2.aav. |
| 5026 | T2.15.haar.1.2.std. |
| 5027 | T2.15.haar.1.3.aav. |
| 5028 | T2.15.haar.1.3.std. |
| 5029 | T2.15.haar.2.1.aav. |
| 5030 | T2.15.haar.2.1.std. |
| 5031 | T2.15.haar.2.2.aav. |
| 5032 | T2.15.haar.2.2.std. |
| 5033 | T2.15.haar.2.3.aav. |
| 5034 | T2.15.haar.2.3.std. |
| 5035 | T2.15.haar.3.1.aav. |
| 5036 | T2.15.haar.3.1.std. |
| 5037 | T2.15.haar.3.2.aav. |
| 5038 | T2.15.haar.3.2.std. |
| 5039 | T2.15.haar.3.3.aav. |
| 5040 | T2.15.haar.3.3.std. |
| 5041 | T2.15.haar.4.1.aav. |
| 5042 | T2.15.haar.4.1.std. |
| 5043 | T2.15.haar.4.2.aav. |
| 5044 | T2.15.haar.4.2.std. |
| 5045 | T2.15.haar.4.3.aav. |
| 5046 | T2.15.haar.4.3.std. |
| 5047 | T2.19.haar.1.1.aav. |
| 5048 | T2.19.haar.1.1.std. |
| 5049 | T2.19.haar.1.2.aav. |
| 5050 | T2.19.haar.1.2.std. |
| 5051 | T2.19.haar.1.3.aav. |
| 5052 | T2.19.haar.1.3.std. |
| 5053 | T2.19.haar.2.1.aav. |
| 5054 | T2.19.haar.2.1.std. |
| 5055 | T2.19.haar.2.2.aav. |
| 5056 | T2.19.haar.2.2.std. |
| 5057 | T2.19.haar.2.3.aav. |
| 5058 | T2.19.haar.2.3.std. |
| 5059 | T2.19.haar.3.1.aav. |
| 5060 | T2.19.haar.3.1.std. |
| 5061 | T2.19.haar.3.2.aav. |
| 5062 | T2.19.haar.3.2.std. |
| 5063 | T2.19.haar.3.3.aav. |
| 5064 | T2.19.haar.3.3.std. |
| 5065 | T2.19.haar.4.1.aav. |
| 5066 | T2.19.haar.4.1.std. |
| 5067 | T2.19.haar.4.2.aav. |
| 5068 | T2.19.haar.4.2.std. |
| 5069 | T2.19.haar.4.3.aav. |
| 5070 | T2.19.haar.4.3.std. |
| 5071 | T2.23.haar.1.1.aav. |
| 5072 | T2.23.haar.1.1.std. |
| 5073 | T2.23.haar.1.2.aav. |
| 5074 | T2.23.haar.1.2.std. |
| 5075 | T2.23.haar.1.3.aav. |
| 5076 | T2.23.haar.1.3.std. |
| 5077 | T2.23.haar.2.1.aav. |
| 5078 | T2.23.haar.2.1.std. |
| 5079 | T2.23.haar.2.2.aav. |
| 5080 | T2.23.haar.2.2.std. |
| 5081 | T2.23.haar.2.3.aav. |
| 5082 | T2.23.haar.2.3.std. |
| 5083 | T2.23.haar.3.1.aav. |
| 5084 | T2.23.haar.3.1.std. |
| 5085 | T2.23.haar.3.2.aav. |
| 5086 | T2.23.haar.3.2.std. |
| 5087 | T2.23.haar.3.3.aav. |
| 5088 | T2.23.haar.3.3.std. |
| 5089 | T2.23.haar.4.1.aav. |
| 5090 | T2.23.haar.4.1.std. |
| 5091 | T2.23.haar.4.2.aav. |
| 5092 | T2.23.haar.4.2.std. |
| 5093 | T2.23.haar.4.3.aav. |
| 5094 | T2.23.haar.4.3.std. |
| 5095 | T2.27.haar.1.1.aav. |
| 5096 | T2.27.haar.1.1.std. |
| 5097 | T2.27.haar.1.2.aav. |
| 5098 | T2.27.haar.1.2.std. |
| 5099 | T2.27.haar.1.3.aav. |
| 5100 | T2.27.haar.1.3.std. |
| 5101 | T2.27.haar.2.1.aav. |
| 5102 | T2.27.haar.2.1.std. |
| 5103 | T2.27.haar.2.2.aav. |
| 5104 | T2.27.haar.2.2.std. |
| 5105 | T2.27.haar.2.3.aav. |
| 5106 | T2.27.haar.2.3.std. |
| 5107 | T2.27.haar.3.1.aav. |
| 5108 | T2.27.haar.3.1.std. |
| 5109 | T2.27.haar.3.2.aav. |
| 5110 | T2.27.haar.3.2.std. |
| 5111 | T2.27.haar.3.3.aav. |
| 5112 | T2.27.haar.3.3.std. |
| 5113 | T2.27.haar.4.1.aav. |
| 5114 | T2.27.haar.4.1.std. |
| 5115 | T2.27.haar.4.2.aav. |
| 5116 | T2.27.haar.4.2.std. |
| 5117 | T2.27.haar.4.3.aav. |
| 5118 | T2.27.haar.4.3.std. |
| 5119 | T2.31.haar.1.1.aav. |
| 5120 | T2.31.haar.1.1.std. |
| 5121 | T2.31.haar.1.2.aav. |
| 5122 | T2.31.haar.1.2.std. |
| 5123 | T2.31.haar.1.3.aav. |
| 5124 | T2.31.haar.1.3.std. |
| 5125 | T2.31.haar.2.1.aav. |
| 5126 | T2.31.haar.2.1.std. |
| 5127 | T2.31.haar.2.2.aav. |
| 5128 | T2.31.haar.2.2.std. |
| 5129 | T2.31.haar.2.3.aav. |
| 5130 | T2.31.haar.2.3.std. |
| 5131 | T2.31.haar.3.1.aav. |
| 5132 | T2.31.haar.3.1.std. |
| 5133 | T2.31.haar.3.2.aav. |
| 5134 | T2.31.haar.3.2.std. |
| 5135 | T2.31.haar.3.3.aav. |
| 5136 | T2.31.haar.3.3.std. |
| 5137 | T2.31.haar.4.1.aav. |
| 5138 | T2.31.haar.4.1.std. |
| 5139 | T2.31.haar.4.2.aav. |
| 5140 | T2.31.haar.4.2.std. |
| 5141 | T2.31.haar.4.3.aav. |
| 5142 | T2.31.haar.4.3.std. |
| 5143 | T2.35.haar.1.1.aav. |
| 5144 | T2.35.haar.1.1.std. |
| 5145 | T2.35.haar.1.2.aav. |
| 5146 | T2.35.haar.1.2.std. |
| 5147 | T2.35.haar.1.3.aav. |
| 5148 | T2.35.haar.1.3.std. |
| 5149 | T2.35.haar.2.1.aav. |
| 5150 | T2.35.haar.2.1.std. |
| 5151 | T2.35.haar.2.2.aav. |
| 5152 | T2.35.haar.2.2.std. |
| 5153 | T2.35.haar.2.3.aav. |
| 5154 | T2.35.haar.2.3.std. |
| 5155 | T2.35.haar.3.1.aav. |
| 5156 | T2.35.haar.3.1.std. |
| 5157 | T2.35.haar.3.2.aav. |
| 5158 | T2.35.haar.3.2.std. |
| 5159 | T2.35.haar.3.3.aav. |
| 5160 | T2.35.haar.3.3.std. |
| 5161 | T2.35.haar.4.1.aav. |
| 5162 | T2.35.haar.4.1.std. |
| 5163 | T2.35.haar.4.2.aav. |
| 5164 | T2.35.haar.4.2.std. |
| 5165 | T2.35.haar.4.3.aav. |
| 5166 | T2.35.haar.4.3.std. |
| 5167 | T2.3.hu.0. |
| 5168 | T2.3.hu.1. |
| 5169 | T2.3.hu.2. |
| 5170 | T2.3.hu.3. |
| 5171 | T2.3.hu.4. |
| 5172 | T2.3.hu.5. |
| 5173 | T2.3.hu.6. |
| 5174 | T2.7.hu.0. |
| 5175 | T2.7.hu.1. |
| 5176 | T2.7.hu.2. |
| 5177 | T2.7.hu.3. |
| 5178 | T2.7.hu.4. |
| 5179 | T2.7.hu.5. |
| 5180 | T2.7.hu.6. |
| 5181 | T2.11.hu.0. |
| 5182 | T2.11.hu.1. |
| 5183 | T2.11.hu.2. |
| 5184 | T2.11.hu.3. |
| 5185 | T2.11.hu.4. |
| 5186 | T2.11.hu.5. |
| 5187 | T2.11.hu.6. |
| 5188 | T2.15.hu.0. |
| 5189 | T2.15.hu.1. |
| 5190 | T2.15.hu.2. |
| 5191 | T2.15.hu.3. |
| 5192 | T2.15.hu.4. |
| 5193 | T2.15.hu.5. |
| 5194 | T2.15.hu.6. |
| 5195 | T2.19.hu.0. |
| 5196 | T2.19.hu.1. |
| 5197 | T2.19.hu.2. |
| 5198 | T2.19.hu.3. |
| 5199 | T2.19.hu.4. |
| 5200 | T2.19.hu.5. |
| 5201 | T2.19.hu.6. |
| 5202 | T2.23.hu.0. |
| 5203 | T2.23.hu.1. |
| 5204 | T2.23.hu.2. |
| 5205 | T2.23.hu.3. |
| 5206 | T2.23.hu.4. |
| 5207 | T2.23.hu.5. |
| 5208 | T2.23.hu.6. |
| 5209 | T2.27.hu.0. |
| 5210 | T2.27.hu.1. |
| 5211 | T2.27.hu.2. |
| 5212 | T2.27.hu.3. |
| 5213 | T2.27.hu.4. |
| 5214 | T2.27.hu.5. |
| 5215 | T2.27.hu.6. |
| 5216 | T2.31.hu.0. |
| 5217 | T2.31.hu.1. |
| 5218 | T2.31.hu.2. |
| 5219 | T2.31.hu.3. |
| 5220 | T2.31.hu.4. |
| 5221 | T2.31.hu.5. |
| 5222 | T2.31.hu.6. |
| 5223 | T2.35.hu.0. |
| 5224 | T2.35.hu.1. |
| 5225 | T2.35.hu.2. |
| 5226 | T2.35.hu.3. |
| 5227 | T2.35.hu.4. |
| 5228 | T2.35.hu.5. |
| 5229 | T2.35.hu.6. |
| 5230 | T2.3.zernike.0. |
| 5231 | T2.3.zernike.1. |
| 5232 | T2.3.zernike.2. |
| 5233 | T2.3.zernike.3. |
| 5234 | T2.3.zernike.4. |
| 5235 | T2.3.zernike.5. |
| 5236 | T2.3.zernike.6. |
| 5237 | T2.3.zernike.7. |
| 5238 | T2.3.zernike.8. |
| 5239 | T2.3.zernike.9. |
| 5240 | T2.3.zernike.10. |
| 5241 | T2.3.zernike.11. |
| 5242 | T2.3.zernike.12. |
| 5243 | T2.3.zernike.13. |
| 5244 | T2.3.zernike.14. |
| 5245 | T2.3.zernike.15. |
| 5246 | T2.3.zernike.16. |
| 5247 | T2.3.zernike.17. |
| 5248 | T2.3.zernike.18. |
| 5249 | T2.3.zernike.19. |
| 5250 | T2.3.zernike.20. |
| 5251 | T2.3.zernike.21. |
| 5252 | T2.3.zernike.22. |
| 5253 | T2.3.zernike.23. |
| 5254 | T2.3.zernike.24. |
| 5255 | T2.7.zernike.0. |
| 5256 | T2.7.zernike.1. |
| 5257 | T2.7.zernike.2. |
| 5258 | T2.7.zernike.3. |
| 5259 | T2.7.zernike.4. |
| 5260 | T2.7.zernike.5. |
| 5261 | T2.7.zernike.6. |
| 5262 | T2.7.zernike.7. |
| 5263 | T2.7.zernike.8. |
| 5264 | T2.7.zernike.9. |
| 5265 | T2.7.zernike.10. |
| 5266 | T2.7.zernike.11. |
| 5267 | T2.7.zernike.12. |
| 5268 | T2.7.zernike.13. |
| 5269 | T2.7.zernike.14. |
| 5270 | T2.7.zernike.15. |
| 5271 | T2.7.zernike.16. |
| 5272 | T2.7.zernike.17. |
| 5273 | T2.7.zernike.18. |
| 5274 | T2.7.zernike.19. |
| 5275 | T2.7.zernike.20. |
| 5276 | T2.7.zernike.21. |
| 5277 | T2.7.zernike.22. |
| 5278 | T2.7.zernike.23. |
| 5279 | T2.7.zernike.24. |
| 5280 | T2.11.zernike.0. |
| 5281 | T2.11.zernike.1. |
| 5282 | T2.11.zernike.2. |
| 5283 | T2.11.zernike.3. |
| 5284 | T2.11.zernike.4. |
| 5285 | T2.11.zernike.5. |
| 5286 | T2.11.zernike.6. |
| 5287 | T2.11.zernike.7. |
| 5288 | T2.11.zernike.8. |
| 5289 | T2.11.zernike.9. |
| 5290 | T2.11.zernike.10. |
| 5291 | T2.11.zernike.11. |
| 5292 | T2.11.zernike.12. |
| 5293 | T2.11.zernike.13. |
| 5294 | T2.11.zernike.14. |
| 5295 | T2.11.zernike.15. |
| 5296 | T2.11.zernike.16. |
| 5297 | T2.11.zernike.17. |
| 5298 | T2.11.zernike.18. |
| 5299 | T2.11.zernike.19. |
| 5300 | T2.11.zernike.20. |
| 5301 | T2.11.zernike.21. |
| 5302 | T2.11.zernike.22. |
| 5303 | T2.11.zernike.23. |
| 5304 | T2.11.zernike.24. |
| 5305 | T2.15.zernike.0. |
| 5306 | T2.15.zernike.1. |
| 5307 | T2.15.zernike.2. |
| 5308 | T2.15.zernike.3. |
| 5309 | T2.15.zernike.4. |
| 5310 | T2.15.zernike.5. |
| 5311 | T2.15.zernike.6. |
| 5312 | T2.15.zernike.7. |
| 5313 | T2.15.zernike.8. |
| 5314 | T2.15.zernike.9. |
| 5315 | T2.15.zernike.10. |
| 5316 | T2.15.zernike.11. |
| 5317 | T2.15.zernike.12. |
| 5318 | T2.15.zernike.13. |
| 5319 | T2.15.zernike.14. |
| 5320 | T2.15.zernike.15. |
| 5321 | T2.15.zernike.16. |
| 5322 | T2.15.zernike.17. |
| 5323 | T2.15.zernike.18. |
| 5324 | T2.15.zernike.19. |
| 5325 | T2.15.zernike.20. |
| 5326 | T2.15.zernike.21. |
| 5327 | T2.15.zernike.22. |
| 5328 | T2.15.zernike.23. |
| 5329 | T2.15.zernike.24. |
| 5330 | T2.19.zernike.0. |
| 5331 | T2.19.zernike.1. |
| 5332 | T2.19.zernike.2. |
| 5333 | T2.19.zernike.3. |
| 5334 | T2.19.zernike.4. |
| 5335 | T2.19.zernike.5. |
| 5336 | T2.19.zernike.6. |
| 5337 | T2.19.zernike.7. |
| 5338 | T2.19.zernike.8. |
| 5339 | T2.19.zernike.9. |
| 5340 | T2.19.zernike.10. |
| 5341 | T2.19.zernike.11. |
| 5342 | T2.19.zernike.12. |
| 5343 | T2.19.zernike.13. |
| 5344 | T2.19.zernike.14. |
| 5345 | T2.19.zernike.15. |
| 5346 | T2.19.zernike.16. |
| 5347 | T2.19.zernike.17. |
| 5348 | T2.19.zernike.18. |
| 5349 | T2.19.zernike.19. |
| 5350 | T2.19.zernike.20. |
| 5351 | T2.19.zernike.21. |
| 5352 | T2.19.zernike.22. |
| 5353 | T2.19.zernike.23. |
| 5354 | T2.19.zernike.24. |
| 5355 | T2.23.zernike.0. |
| 5356 | T2.23.zernike.1. |
| 5357 | T2.23.zernike.2. |
| 5358 | T2.23.zernike.3. |
| 5359 | T2.23.zernike.4. |
| 5360 | T2.23.zernike.5. |
| 5361 | T2.23.zernike.6. |
| 5362 | T2.23.zernike.7. |
| 5363 | T2.23.zernike.8. |
| 5364 | T2.23.zernike.9. |
| 5365 | T2.23.zernike.10. |
| 5366 | T2.23.zernike.11. |
| 5367 | T2.23.zernike.12. |
| 5368 | T2.23.zernike.13. |
| 5369 | T2.23.zernike.14. |
| 5370 | T2.23.zernike.15. |
| 5371 | T2.23.zernike.16. |
| 5372 | T2.23.zernike.17. |
| 5373 | T2.23.zernike.18. |
| 5374 | T2.23.zernike.19. |
| 5375 | T2.23.zernike.20. |
| 5376 | T2.23.zernike.21. |
| 5377 | T2.23.zernike.22. |
| 5378 | T2.23.zernike.23. |
| 5379 | T2.23.zernike.24. |
| 5380 | T2.27.zernike.0. |
| 5381 | T2.27.zernike.1. |
| 5382 | T2.27.zernike.2. |
| 5383 | T2.27.zernike.3. |
| 5384 | T2.27.zernike.4. |
| 5385 | T2.27.zernike.5. |
| 5386 | T2.27.zernike.6. |
| 5387 | T2.27.zernike.7. |
| 5388 | T2.27.zernike.8. |
| 5389 | T2.27.zernike.9. |
| 5390 | T2.27.zernike.10. |
| 5391 | T2.27.zernike.11. |
| 5392 | T2.27.zernike.12. |
| 5393 | T2.27.zernike.13. |
| 5394 | T2.27.zernike.14. |
| 5395 | T2.27.zernike.15. |
| 5396 | T2.27.zernike.16. |
| 5397 | T2.27.zernike.17. |
| 5398 | T2.27.zernike.18. |
| 5399 | T2.27.zernike.19. |
| 5400 | T2.27.zernike.20. |
| 5401 | T2.27.zernike.21. |
| 5402 | T2.27.zernike.22. |
| 5403 | T2.27.zernike.23. |
| 5404 | T2.27.zernike.24. |
| 5405 | T2.31.zernike.0. |
| 5406 | T2.31.zernike.1. |
| 5407 | T2.31.zernike.2. |
| 5408 | T2.31.zernike.3. |
| 5409 | T2.31.zernike.4. |
| 5410 | T2.31.zernike.5. |
| 5411 | T2.31.zernike.6. |
| 5412 | T2.31.zernike.7. |
| 5413 | T2.31.zernike.8. |
| 5414 | T2.31.zernike.9. |
| 5415 | T2.31.zernike.10. |
| 5416 | T2.31.zernike.11. |
| 5417 | T2.31.zernike.12. |
| 5418 | T2.31.zernike.13. |
| 5419 | T2.31.zernike.14. |
| 5420 | T2.31.zernike.15. |
| 5421 | T2.31.zernike.16. |
| 5422 | T2.31.zernike.17. |
| 5423 | T2.31.zernike.18. |
| 5424 | T2.31.zernike.19. |
| 5425 | T2.31.zernike.20. |
| 5426 | T2.31.zernike.21. |
| 5427 | T2.31.zernike.22. |
| 5428 | T2.31.zernike.23. |
| 5429 | T2.31.zernike.24. |
| 5430 | T2.35.zernike.0. |
| 5431 | T2.35.zernike.1. |
| 5432 | T2.35.zernike.2. |
| 5433 | T2.35.zernike.3. |
| 5434 | T2.35.zernike.4. |
| 5435 | T2.35.zernike.5. |
| 5436 | T2.35.zernike.6. |
| 5437 | T2.35.zernike.7. |
| 5438 | T2.35.zernike.8. |
| 5439 | T2.35.zernike.9. |
| 5440 | T2.35.zernike.10. |
| 5441 | T2.35.zernike.11. |
| 5442 | T2.35.zernike.12. |
| 5443 | T2.35.zernike.13. |
| 5444 | T2.35.zernike.14. |
| 5445 | T2.35.zernike.15. |
| 5446 | T2.35.zernike.16. |
| 5447 | T2.35.zernike.17. |
| 5448 | T2.35.zernike.18. |
| 5449 | T2.35.zernike.19. |
| 5450 | T2.35.zernike.20. |
| 5451 | T2.35.zernike.21. |
| 5452 | T2.35.zernike.22. |
| 5453 | T2.35.zernike.23. |
| 5454 | T2.35.zernike.24. |
| 5455 | T2.3.sobel |
| 5456 | T2.3.sobel_mask |
| 5457 | T2.all.stats.p000. |
| 5458 | T2.all.stats.p010. |
| 5459 | T2.all.stats.p020. |
| 5460 | T2.all.stats.p025. |
| 5461 | T2.all.stats.p030. |
| 5462 | T2.all.stats.p040. |
| 5463 | T2.all.stats.p050. |
| 5464 | T2.all.stats.p060. |
| 5465 | T2.all.stats.p070. |
| 5466 | T2.all.stats.p075. |
| 5467 | T2.all.stats.p080. |
| 5468 | T2.all.stats.p090. |
| 5469 | T2.all.stats.p100. |
| 5470 | T2.all.stats.range. |
| 5471 | T2.all.stats.mean. |
| 5472 | T2.all.stats.stddev. |
| 5473 | T2.all.stats.kurtosis. |
| 5474 | T2.all.stats.skewness. |
| 5475 | T2W.3.glcm.contrast.1.mean. |
| 5476 | T2W.3.glcm.contrast.1.range. |
| 5477 | T2W.3.glcm.contrast.2.mean. |
| 5478 | T2W.3.glcm.contrast.2.range. |
| 5479 | T2W.3.glcm.contrast.3.mean. |
| 5480 | T2W.3.glcm.contrast.3.range. |
| 5481 | T2W.3.glcm.dissimilarity.1.mean. |
| 5482 | T2W.3.glcm.dissimilarity.1.range. |
| 5483 | T2W.3.glcm.dissimilarity.2.mean. |
| 5484 | T2W.3.glcm.dissimilarity.2.range. |
| 5485 | T2W.3.glcm.dissimilarity.3.mean. |
| 5486 | T2W.3.glcm.dissimilarity.3.range. |
| 5487 | T2W.3.glcm.homogeneity.1.mean. |
| 5488 | T2W.3.glcm.homogeneity.1.range. |
| 5489 | T2W.3.glcm.homogeneity.2.mean. |
| 5490 | T2W.3.glcm.homogeneity.2.range. |
| 5491 | T2W.3.glcm.homogeneity.3.mean. |
| 5492 | T2W.3.glcm.homogeneity.3.range. |
| 5493 | T2W.3.glcm.energy.1.mean. |
| 5494 | T2W.3.glcm.energy.1.range. |
| 5495 | T2W.3.glcm.energy.2.mean. |
| 5496 | T2W.3.glcm.energy.2.range. |
| 5497 | T2W.3.glcm.energy.3.mean. |
| 5498 | T2W.3.glcm.energy.3.range. |
| 5499 | T2W.3.glcm.correlation.1.mean. |
| 5500 | T2W.3.glcm.correlation.1.range. |
| 5501 | T2W.3.glcm.correlation.2.mean. |
| 5502 | T2W.3.glcm.correlation.2.range. |
| 5503 | T2W.3.glcm.correlation.3.mean. |
| 5504 | T2W.3.glcm.correlation.3.range. |
| 5505 | T2W.3.glcm.ASM.1.mean. |
| 5506 | T2W.3.glcm.ASM.1.range. |
| 5507 | T2W.3.glcm.ASM.2.mean. |
| 5508 | T2W.3.glcm.ASM.2.range. |
| 5509 | T2W.3.glcm.ASM.3.mean. |
| 5510 | T2W.3.glcm.ASM.3.range. |
| 5511 | T2W.7.glcm.contrast.1.mean. |
| 5512 | T2W.7.glcm.contrast.1.range. |
| 5513 | T2W.7.glcm.contrast.2.mean. |
| 5514 | T2W.7.glcm.contrast.2.range. |
| 5515 | T2W.7.glcm.contrast.3.mean. |
| 5516 | T2W.7.glcm.contrast.3.range. |
| 5517 | T2W.7.glcm.contrast.4.mean. |
| 5518 | T2W.7.glcm.contrast.4.range. |
| 5519 | T2W.7.glcm.dissimilarity.1.mean. |
| 5520 | T2W.7.glcm.dissimilarity.1.range. |
| 5521 | T2W.7.glcm.dissimilarity.2.mean. |
| 5522 | T2W.7.glcm.dissimilarity.2.range. |
| 5523 | T2W.7.glcm.dissimilarity.3.mean. |
| 5524 | T2W.7.glcm.dissimilarity.3.range. |
| 5525 | T2W.7.glcm.dissimilarity.4.mean. |
| 5526 | T2W.7.glcm.dissimilarity.4.range. |
| 5527 | T2W.7.glcm.homogeneity.1.mean. |
| 5528 | T2W.7.glcm.homogeneity.1.range. |
| 5529 | T2W.7.glcm.homogeneity.2.mean. |
| 5530 | T2W.7.glcm.homogeneity.2.range. |
| 5531 | T2W.7.glcm.homogeneity.3.mean. |
| 5532 | T2W.7.glcm.homogeneity.3.range. |
| 5533 | T2W.7.glcm.homogeneity.4.mean. |
| 5534 | T2W.7.glcm.homogeneity.4.range. |
| 5535 | T2W.7.glcm.energy.1.mean. |
| 5536 | T2W.7.glcm.energy.1.range. |
| 5537 | T2W.7.glcm.energy.2.mean. |
| 5538 | T2W.7.glcm.energy.2.range. |
| 5539 | T2W.7.glcm.energy.3.mean. |
| 5540 | T2W.7.glcm.energy.3.range. |
| 5541 | T2W.7.glcm.energy.4.mean. |
| 5542 | T2W.7.glcm.energy.4.range. |
| 5543 | T2W.7.glcm.correlation.1.mean. |
| 5544 | T2W.7.glcm.correlation.1.range. |
| 5545 | T2W.7.glcm.correlation.2.mean. |
| 5546 | T2W.7.glcm.correlation.2.range. |
| 5547 | T2W.7.glcm.correlation.3.mean. |
| 5548 | T2W.7.glcm.correlation.3.range. |
| 5549 | T2W.7.glcm.correlation.4.mean. |
| 5550 | T2W.7.glcm.correlation.4.range. |
| 5551 | T2W.7.glcm.ASM.1.mean. |
| 5552 | T2W.7.glcm.ASM.1.range. |
| 5553 | T2W.7.glcm.ASM.2.mean. |
| 5554 | T2W.7.glcm.ASM.2.range. |
| 5555 | T2W.7.glcm.ASM.3.mean. |
| 5556 | T2W.7.glcm.ASM.3.range. |
| 5557 | T2W.7.glcm.ASM.4.mean. |
| 5558 | T2W.7.glcm.ASM.4.range. |
| 5559 | T2W.11.glcm.contrast.1.mean. |
| 5560 | T2W.11.glcm.contrast.1.range. |
| 5561 | T2W.11.glcm.contrast.2.mean. |
| 5562 | T2W.11.glcm.contrast.2.range. |
| 5563 | T2W.11.glcm.contrast.3.mean. |
| 5564 | T2W.11.glcm.contrast.3.range. |
| 5565 | T2W.11.glcm.contrast.4.mean. |
| 5566 | T2W.11.glcm.contrast.4.range. |
| 5567 | T2W.11.glcm.dissimilarity.1.mean. |
| 5568 | T2W.11.glcm.dissimilarity.1.range. |
| 5569 | T2W.11.glcm.dissimilarity.2.mean. |
| 5570 | T2W.11.glcm.dissimilarity.2.range. |
| 5571 | T2W.11.glcm.dissimilarity.3.mean. |
| 5572 | T2W.11.glcm.dissimilarity.3.range. |
| 5573 | T2W.11.glcm.dissimilarity.4.mean. |
| 5574 | T2W.11.glcm.dissimilarity.4.range. |
| 5575 | T2W.11.glcm.homogeneity.1.mean. |
| 5576 | T2W.11.glcm.homogeneity.1.range. |
| 5577 | T2W.11.glcm.homogeneity.2.mean. |
| 5578 | T2W.11.glcm.homogeneity.2.range. |
| 5579 | T2W.11.glcm.homogeneity.3.mean. |
| 5580 | T2W.11.glcm.homogeneity.3.range. |
| 5581 | T2W.11.glcm.homogeneity.4.mean. |
| 5582 | T2W.11.glcm.homogeneity.4.range. |
| 5583 | T2W.11.glcm.energy.1.mean. |
| 5584 | T2W.11.glcm.energy.1.range. |
| 5585 | T2W.11.glcm.energy.2.mean. |
| 5586 | T2W.11.glcm.energy.2.range. |
| 5587 | T2W.11.glcm.energy.3.mean. |
| 5588 | T2W.11.glcm.energy.3.range. |
| 5589 | T2W.11.glcm.energy.4.mean. |
| 5590 | T2W.11.glcm.energy.4.range. |
| 5591 | T2W.11.glcm.correlation.1.mean. |
| 5592 | T2W.11.glcm.correlation.1.range. |
| 5593 | T2W.11.glcm.correlation.2.mean. |
| 5594 | T2W.11.glcm.correlation.2.range. |
| 5595 | T2W.11.glcm.correlation.3.mean. |
| 5596 | T2W.11.glcm.correlation.3.range. |
| 5597 | T2W.11.glcm.correlation.4.mean. |
| 5598 | T2W.11.glcm.correlation.4.range. |
| 5599 | T2W.11.glcm.ASM.1.mean. |
| 5600 | T2W.11.glcm.ASM.1.range. |
| 5601 | T2W.11.glcm.ASM.2.mean. |
| 5602 | T2W.11.glcm.ASM.2.range. |
| 5603 | T2W.11.glcm.ASM.3.mean. |
| 5604 | T2W.11.glcm.ASM.3.range. |
| 5605 | T2W.11.glcm.ASM.4.mean. |
| 5606 | T2W.11.glcm.ASM.4.range. |
| 5607 | T2W.15.glcm.contrast.1.mean. |
| 5608 | T2W.15.glcm.contrast.1.range. |
| 5609 | T2W.15.glcm.contrast.2.mean. |
| 5610 | T2W.15.glcm.contrast.2.range. |
| 5611 | T2W.15.glcm.contrast.3.mean. |
| 5612 | T2W.15.glcm.contrast.3.range. |
| 5613 | T2W.15.glcm.contrast.4.mean. |
| 5614 | T2W.15.glcm.contrast.4.range. |
| 5615 | T2W.15.glcm.dissimilarity.1.mean. |
| 5616 | T2W.15.glcm.dissimilarity.1.range. |
| 5617 | T2W.15.glcm.dissimilarity.2.mean. |
| 5618 | T2W.15.glcm.dissimilarity.2.range. |
| 5619 | T2W.15.glcm.dissimilarity.3.mean. |
| 5620 | T2W.15.glcm.dissimilarity.3.range. |
| 5621 | T2W.15.glcm.dissimilarity.4.mean. |
| 5622 | T2W.15.glcm.dissimilarity.4.range. |
| 5623 | T2W.15.glcm.homogeneity.1.mean. |
| 5624 | T2W.15.glcm.homogeneity.1.range. |
| 5625 | T2W.15.glcm.homogeneity.2.mean. |
| 5626 | T2W.15.glcm.homogeneity.2.range. |
| 5627 | T2W.15.glcm.homogeneity.3.mean. |
| 5628 | T2W.15.glcm.homogeneity.3.range. |
| 5629 | T2W.15.glcm.homogeneity.4.mean. |
| 5630 | T2W.15.glcm.homogeneity.4.range. |
| 5631 | T2W.15.glcm.energy.1.mean. |
| 5632 | T2W.15.glcm.energy.1.range. |
| 5633 | T2W.15.glcm.energy.2.mean. |
| 5634 | T2W.15.glcm.energy.2.range. |
| 5635 | T2W.15.glcm.energy.3.mean. |
| 5636 | T2W.15.glcm.energy.3.range. |
| 5637 | T2W.15.glcm.energy.4.mean. |
| 5638 | T2W.15.glcm.energy.4.range. |
| 5639 | T2W.15.glcm.correlation.1.mean. |
| 5640 | T2W.15.glcm.correlation.1.range. |
| 5641 | T2W.15.glcm.correlation.2.mean. |
| 5642 | T2W.15.glcm.correlation.2.range. |
| 5643 | T2W.15.glcm.correlation.3.mean. |
| 5644 | T2W.15.glcm.correlation.3.range. |
| 5645 | T2W.15.glcm.correlation.4.mean. |
| 5646 | T2W.15.glcm.correlation.4.range. |
| 5647 | T2W.15.glcm.ASM.1.mean. |
| 5648 | T2W.15.glcm.ASM.1.range. |
| 5649 | T2W.15.glcm.ASM.2.mean. |
| 5650 | T2W.15.glcm.ASM.2.range. |
| 5651 | T2W.15.glcm.ASM.3.mean. |
| 5652 | T2W.15.glcm.ASM.3.range. |
| 5653 | T2W.15.glcm.ASM.4.mean. |
| 5654 | T2W.15.glcm.ASM.4.range. |
| 5655 | T2W.19.glcm.contrast.1.mean. |
| 5656 | T2W.19.glcm.contrast.1.range. |
| 5657 | T2W.19.glcm.contrast.2.mean. |
| 5658 | T2W.19.glcm.contrast.2.range. |
| 5659 | T2W.19.glcm.contrast.3.mean. |
| 5660 | T2W.19.glcm.contrast.3.range. |
| 5661 | T2W.19.glcm.contrast.4.mean. |
| 5662 | T2W.19.glcm.contrast.4.range. |
| 5663 | T2W.19.glcm.dissimilarity.1.mean. |
| 5664 | T2W.19.glcm.dissimilarity.1.range. |
| 5665 | T2W.19.glcm.dissimilarity.2.mean. |
| 5666 | T2W.19.glcm.dissimilarity.2.range. |
| 5667 | T2W.19.glcm.dissimilarity.3.mean. |
| 5668 | T2W.19.glcm.dissimilarity.3.range. |
| 5669 | T2W.19.glcm.dissimilarity.4.mean. |
| 5670 | T2W.19.glcm.dissimilarity.4.range. |
| 5671 | T2W.19.glcm.homogeneity.1.mean. |
| 5672 | T2W.19.glcm.homogeneity.1.range. |
| 5673 | T2W.19.glcm.homogeneity.2.mean. |
| 5674 | T2W.19.glcm.homogeneity.2.range. |
| 5675 | T2W.19.glcm.homogeneity.3.mean. |
| 5676 | T2W.19.glcm.homogeneity.3.range. |
| 5677 | T2W.19.glcm.homogeneity.4.mean. |
| 5678 | T2W.19.glcm.homogeneity.4.range. |
| 5679 | T2W.19.glcm.energy.1.mean. |
| 5680 | T2W.19.glcm.energy.1.range. |
| 5681 | T2W.19.glcm.energy.2.mean. |
| 5682 | T2W.19.glcm.energy.2.range. |
| 5683 | T2W.19.glcm.energy.3.mean. |
| 5684 | T2W.19.glcm.energy.3.range. |
| 5685 | T2W.19.glcm.energy.4.mean. |
| 5686 | T2W.19.glcm.energy.4.range. |
| 5687 | T2W.19.glcm.correlation.1.mean. |
| 5688 | T2W.19.glcm.correlation.1.range. |
| 5689 | T2W.19.glcm.correlation.2.mean. |
| 5690 | T2W.19.glcm.correlation.2.range. |
| 5691 | T2W.19.glcm.correlation.3.mean. |
| 5692 | T2W.19.glcm.correlation.3.range. |
| 5693 | T2W.19.glcm.correlation.4.mean. |
| 5694 | T2W.19.glcm.correlation.4.range. |
| 5695 | T2W.19.glcm.ASM.1.mean. |
| 5696 | T2W.19.glcm.ASM.1.range. |
| 5697 | T2W.19.glcm.ASM.2.mean. |
| 5698 | T2W.19.glcm.ASM.2.range. |
| 5699 | T2W.19.glcm.ASM.3.mean. |
| 5700 | T2W.19.glcm.ASM.3.range. |
| 5701 | T2W.19.glcm.ASM.4.mean. |
| 5702 | T2W.19.glcm.ASM.4.range. |
| 5703 | T2W.23.glcm.contrast.1.mean. |
| 5704 | T2W.23.glcm.contrast.1.range. |
| 5705 | T2W.23.glcm.contrast.2.mean. |
| 5706 | T2W.23.glcm.contrast.2.range. |
| 5707 | T2W.23.glcm.contrast.3.mean. |
| 5708 | T2W.23.glcm.contrast.3.range. |
| 5709 | T2W.23.glcm.contrast.4.mean. |
| 5710 | T2W.23.glcm.contrast.4.range. |
| 5711 | T2W.23.glcm.dissimilarity.1.mean. |
| 5712 | T2W.23.glcm.dissimilarity.1.range. |
| 5713 | T2W.23.glcm.dissimilarity.2.mean. |
| 5714 | T2W.23.glcm.dissimilarity.2.range. |
| 5715 | T2W.23.glcm.dissimilarity.3.mean. |
| 5716 | T2W.23.glcm.dissimilarity.3.range. |
| 5717 | T2W.23.glcm.dissimilarity.4.mean. |
| 5718 | T2W.23.glcm.dissimilarity.4.range. |
| 5719 | T2W.23.glcm.homogeneity.1.mean. |
| 5720 | T2W.23.glcm.homogeneity.1.range. |
| 5721 | T2W.23.glcm.homogeneity.2.mean. |
| 5722 | T2W.23.glcm.homogeneity.2.range. |
| 5723 | T2W.23.glcm.homogeneity.3.mean. |
| 5724 | T2W.23.glcm.homogeneity.3.range. |
| 5725 | T2W.23.glcm.homogeneity.4.mean. |
| 5726 | T2W.23.glcm.homogeneity.4.range. |
| 5727 | T2W.23.glcm.energy.1.mean. |
| 5728 | T2W.23.glcm.energy.1.range. |
| 5729 | T2W.23.glcm.energy.2.mean. |
| 5730 | T2W.23.glcm.energy.2.range. |
| 5731 | T2W.23.glcm.energy.3.mean. |
| 5732 | T2W.23.glcm.energy.3.range. |
| 5733 | T2W.23.glcm.energy.4.mean. |
| 5734 | T2W.23.glcm.energy.4.range. |
| 5735 | T2W.23.glcm.correlation.1.mean. |
| 5736 | T2W.23.glcm.correlation.1.range. |
| 5737 | T2W.23.glcm.correlation.2.mean. |
| 5738 | T2W.23.glcm.correlation.2.range. |
| 5739 | T2W.23.glcm.correlation.3.mean. |
| 5740 | T2W.23.glcm.correlation.3.range. |
| 5741 | T2W.23.glcm.correlation.4.mean. |
| 5742 | T2W.23.glcm.correlation.4.range. |
| 5743 | T2W.23.glcm.ASM.1.mean. |
| 5744 | T2W.23.glcm.ASM.1.range. |
| 5745 | T2W.23.glcm.ASM.2.mean. |
| 5746 | T2W.23.glcm.ASM.2.range. |
| 5747 | T2W.23.glcm.ASM.3.mean. |
| 5748 | T2W.23.glcm.ASM.3.range. |
| 5749 | T2W.23.glcm.ASM.4.mean. |
| 5750 | T2W.23.glcm.ASM.4.range. |
| 5751 | T2W.27.glcm.contrast.1.mean. |
| 5752 | T2W.27.glcm.contrast.1.range. |
| 5753 | T2W.27.glcm.contrast.2.mean. |
| 5754 | T2W.27.glcm.contrast.2.range. |
| 5755 | T2W.27.glcm.contrast.3.mean. |
| 5756 | T2W.27.glcm.contrast.3.range. |
| 5757 | T2W.27.glcm.contrast.4.mean. |
| 5758 | T2W.27.glcm.contrast.4.range. |
| 5759 | T2W.27.glcm.dissimilarity.1.mean. |
| 5760 | T2W.27.glcm.dissimilarity.1.range. |
| 5761 | T2W.27.glcm.dissimilarity.2.mean. |
| 5762 | T2W.27.glcm.dissimilarity.2.range. |
| 5763 | T2W.27.glcm.dissimilarity.3.mean. |
| 5764 | T2W.27.glcm.dissimilarity.3.range. |
| 5765 | T2W.27.glcm.dissimilarity.4.mean. |
| 5766 | T2W.27.glcm.dissimilarity.4.range. |
| 5767 | T2W.27.glcm.homogeneity.1.mean. |
| 5768 | T2W.27.glcm.homogeneity.1.range. |
| 5769 | T2W.27.glcm.homogeneity.2.mean. |
| 5770 | T2W.27.glcm.homogeneity.2.range. |
| 5771 | T2W.27.glcm.homogeneity.3.mean. |
| 5772 | T2W.27.glcm.homogeneity.3.range. |
| 5773 | T2W.27.glcm.homogeneity.4.mean. |
| 5774 | T2W.27.glcm.homogeneity.4.range. |
| 5775 | T2W.27.glcm.energy.1.mean. |
| 5776 | T2W.27.glcm.energy.1.range. |
| 5777 | T2W.27.glcm.energy.2.mean. |
| 5778 | T2W.27.glcm.energy.2.range. |
| 5779 | T2W.27.glcm.energy.3.mean. |
| 5780 | T2W.27.glcm.energy.3.range. |
| 5781 | T2W.27.glcm.energy.4.mean. |
| 5782 | T2W.27.glcm.energy.4.range. |
| 5783 | T2W.27.glcm.correlation.1.mean. |
| 5784 | T2W.27.glcm.correlation.1.range. |
| 5785 | T2W.27.glcm.correlation.2.mean. |
| 5786 | T2W.27.glcm.correlation.2.range. |
| 5787 | T2W.27.glcm.correlation.3.mean. |
| 5788 | T2W.27.glcm.correlation.3.range. |
| 5789 | T2W.27.glcm.correlation.4.mean. |
| 5790 | T2W.27.glcm.correlation.4.range. |
| 5791 | T2W.27.glcm.ASM.1.mean. |
| 5792 | T2W.27.glcm.ASM.1.range. |
| 5793 | T2W.27.glcm.ASM.2.mean. |
| 5794 | T2W.27.glcm.ASM.2.range. |
| 5795 | T2W.27.glcm.ASM.3.mean. |
| 5796 | T2W.27.glcm.ASM.3.range. |
| 5797 | T2W.27.glcm.ASM.4.mean. |
| 5798 | T2W.27.glcm.ASM.4.range. |
| 5799 | T2W.31.glcm.contrast.1.mean. |
| 5800 | T2W.31.glcm.contrast.1.range. |
| 5801 | T2W.31.glcm.contrast.2.mean. |
| 5802 | T2W.31.glcm.contrast.2.range. |
| 5803 | T2W.31.glcm.contrast.3.mean. |
| 5804 | T2W.31.glcm.contrast.3.range. |
| 5805 | T2W.31.glcm.contrast.4.mean. |
| 5806 | T2W.31.glcm.contrast.4.range. |
| 5807 | T2W.31.glcm.dissimilarity.1.mean. |
| 5808 | T2W.31.glcm.dissimilarity.1.range. |
| 5809 | T2W.31.glcm.dissimilarity.2.mean. |
| 5810 | T2W.31.glcm.dissimilarity.2.range. |
| 5811 | T2W.31.glcm.dissimilarity.3.mean. |
| 5812 | T2W.31.glcm.dissimilarity.3.range. |
| 5813 | T2W.31.glcm.dissimilarity.4.mean. |
| 5814 | T2W.31.glcm.dissimilarity.4.range. |
| 5815 | T2W.31.glcm.homogeneity.1.mean. |
| 5816 | T2W.31.glcm.homogeneity.1.range. |
| 5817 | T2W.31.glcm.homogeneity.2.mean. |
| 5818 | T2W.31.glcm.homogeneity.2.range. |
| 5819 | T2W.31.glcm.homogeneity.3.mean. |
| 5820 | T2W.31.glcm.homogeneity.3.range. |
| 5821 | T2W.31.glcm.homogeneity.4.mean. |
| 5822 | T2W.31.glcm.homogeneity.4.range. |
| 5823 | T2W.31.glcm.energy.1.mean. |
| 5824 | T2W.31.glcm.energy.1.range. |
| 5825 | T2W.31.glcm.energy.2.mean. |
| 5826 | T2W.31.glcm.energy.2.range. |
| 5827 | T2W.31.glcm.energy.3.mean. |
| 5828 | T2W.31.glcm.energy.3.range. |
| 5829 | T2W.31.glcm.energy.4.mean. |
| 5830 | T2W.31.glcm.energy.4.range. |
| 5831 | T2W.31.glcm.correlation.1.mean. |
| 5832 | T2W.31.glcm.correlation.1.range. |
| 5833 | T2W.31.glcm.correlation.2.mean. |
| 5834 | T2W.31.glcm.correlation.2.range. |
| 5835 | T2W.31.glcm.correlation.3.mean. |
| 5836 | T2W.31.glcm.correlation.3.range. |
| 5837 | T2W.31.glcm.correlation.4.mean. |
| 5838 | T2W.31.glcm.correlation.4.range. |
| 5839 | T2W.31.glcm.ASM.1.mean. |
| 5840 | T2W.31.glcm.ASM.1.range. |
| 5841 | T2W.31.glcm.ASM.2.mean. |
| 5842 | T2W.31.glcm.ASM.2.range. |
| 5843 | T2W.31.glcm.ASM.3.mean. |
| 5844 | T2W.31.glcm.ASM.3.range. |
| 5845 | T2W.31.glcm.ASM.4.mean. |
| 5846 | T2W.31.glcm.ASM.4.range. |
| 5847 | T2W.35.glcm.contrast.1.mean. |
| 5848 | T2W.35.glcm.contrast.1.range. |
| 5849 | T2W.35.glcm.contrast.2.mean. |
| 5850 | T2W.35.glcm.contrast.2.range. |
| 5851 | T2W.35.glcm.contrast.3.mean. |
| 5852 | T2W.35.glcm.contrast.3.range. |
| 5853 | T2W.35.glcm.contrast.4.mean. |
| 5854 | T2W.35.glcm.contrast.4.range. |
| 5855 | T2W.35.glcm.dissimilarity.1.mean. |
| 5856 | T2W.35.glcm.dissimilarity.1.range. |
| 5857 | T2W.35.glcm.dissimilarity.2.mean. |
| 5858 | T2W.35.glcm.dissimilarity.2.range. |
| 5859 | T2W.35.glcm.dissimilarity.3.mean. |
| 5860 | T2W.35.glcm.dissimilarity.3.range. |
| 5861 | T2W.35.glcm.dissimilarity.4.mean. |
| 5862 | T2W.35.glcm.dissimilarity.4.range. |
| 5863 | T2W.35.glcm.homogeneity.1.mean. |
| 5864 | T2W.35.glcm.homogeneity.1.range. |
| 5865 | T2W.35.glcm.homogeneity.2.mean. |
| 5866 | T2W.35.glcm.homogeneity.2.range. |
| 5867 | T2W.35.glcm.homogeneity.3.mean. |
| 5868 | T2W.35.glcm.homogeneity.3.range. |
| 5869 | T2W.35.glcm.homogeneity.4.mean. |
| 5870 | T2W.35.glcm.homogeneity.4.range. |
| 5871 | T2W.35.glcm.energy.1.mean. |
| 5872 | T2W.35.glcm.energy.1.range. |
| 5873 | T2W.35.glcm.energy.2.mean. |
| 5874 | T2W.35.glcm.energy.2.range. |
| 5875 | T2W.35.glcm.energy.3.mean. |
| 5876 | T2W.35.glcm.energy.3.range. |
| 5877 | T2W.35.glcm.energy.4.mean. |
| 5878 | T2W.35.glcm.energy.4.range. |
| 5879 | T2W.35.glcm.correlation.1.mean. |
| 5880 | T2W.35.glcm.correlation.1.range. |
| 5881 | T2W.35.glcm.correlation.2.mean. |
| 5882 | T2W.35.glcm.correlation.2.range. |
| 5883 | T2W.35.glcm.correlation.3.mean. |
| 5884 | T2W.35.glcm.correlation.3.range. |
| 5885 | T2W.35.glcm.correlation.4.mean. |
| 5886 | T2W.35.glcm.correlation.4.range. |
| 5887 | T2W.35.glcm.ASM.1.mean. |
| 5888 | T2W.35.glcm.ASM.1.range. |
| 5889 | T2W.35.glcm.ASM.2.mean. |
| 5890 | T2W.35.glcm.ASM.2.range. |
| 5891 | T2W.35.glcm.ASM.3.mean. |
| 5892 | T2W.35.glcm.ASM.3.range. |
| 5893 | T2W.35.glcm.ASM.4.mean. |
| 5894 | T2W.35.glcm.ASM.4.range. |
| 5895 | T2W.mbb.glcm.contrast.1.mean. |
| 5896 | T2W.mbb.glcm.contrast.1.range. |
| 5897 | T2W.mbb.glcm.contrast.2.mean. |
| 5898 | T2W.mbb.glcm.contrast.2.range. |
| 5899 | T2W.mbb.glcm.contrast.3.mean. |
| 5900 | T2W.mbb.glcm.contrast.3.range. |
| 5901 | T2W.mbb.glcm.contrast.4.mean. |
| 5902 | T2W.mbb.glcm.contrast.4.range. |
| 5903 | T2W.mbb.glcm.dissimilarity.1.mean. |
| 5904 | T2W.mbb.glcm.dissimilarity.1.range. |
| 5905 | T2W.mbb.glcm.dissimilarity.2.mean. |
| 5906 | T2W.mbb.glcm.dissimilarity.2.range. |
| 5907 | T2W.mbb.glcm.dissimilarity.3.mean. |
| 5908 | T2W.mbb.glcm.dissimilarity.3.range. |
| 5909 | T2W.mbb.glcm.dissimilarity.4.mean. |
| 5910 | T2W.mbb.glcm.dissimilarity.4.range. |
| 5911 | T2W.mbb.glcm.homogeneity.1.mean. |
| 5912 | T2W.mbb.glcm.homogeneity.1.range. |
| 5913 | T2W.mbb.glcm.homogeneity.2.mean. |
| 5914 | T2W.mbb.glcm.homogeneity.2.range. |
| 5915 | T2W.mbb.glcm.homogeneity.3.mean. |
| 5916 | T2W.mbb.glcm.homogeneity.3.range. |
| 5917 | T2W.mbb.glcm.homogeneity.4.mean. |
| 5918 | T2W.mbb.glcm.homogeneity.4.range. |
| 5919 | T2W.mbb.glcm.energy.1.mean. |
| 5920 | T2W.mbb.glcm.energy.1.range. |
| 5921 | T2W.mbb.glcm.energy.2.mean. |
| 5922 | T2W.mbb.glcm.energy.2.range. |
| 5923 | T2W.mbb.glcm.energy.3.mean. |
| 5924 | T2W.mbb.glcm.energy.3.range. |
| 5925 | T2W.mbb.glcm.energy.4.mean. |
| 5926 | T2W.mbb.glcm.energy.4.range. |
| 5927 | T2W.mbb.glcm.correlation.1.mean. |
| 5928 | T2W.mbb.glcm.correlation.1.range. |
| 5929 | T2W.mbb.glcm.correlation.2.mean. |
| 5930 | T2W.mbb.glcm.correlation.2.range. |
| 5931 | T2W.mbb.glcm.correlation.3.mean. |
| 5932 | T2W.mbb.glcm.correlation.3.range. |
| 5933 | T2W.mbb.glcm.correlation.4.mean. |
| 5934 | T2W.mbb.glcm.correlation.4.range. |
| 5935 | T2W.mbb.glcm.ASM.1.mean. |
| 5936 | T2W.mbb.glcm.ASM.1.range. |
| 5937 | T2W.mbb.glcm.ASM.2.mean. |
| 5938 | T2W.mbb.glcm.ASM.2.range. |
| 5939 | T2W.mbb.glcm.ASM.3.mean. |
| 5940 | T2W.mbb.glcm.ASM.3.range. |
| 5941 | T2W.mbb.glcm.ASM.4.mean. |
| 5942 | T2W.mbb.glcm.ASM.4.range. |
| 5943 | T2W.3.lbp.1.0. |
| 5944 | T2W.3.lbp.1.1. |
| 5945 | T2W.3.lbp.1.2. |
| 5946 | T2W.3.lbp.1.3. |
| 5947 | T2W.3.lbp.1.4. |
| 5948 | T2W.3.lbp.1.5. |
| 5949 | T2W.3.lbp.1.6. |
| 5950 | T2W.3.lbp.1.7. |
| 5951 | T2W.3.lbp.1.8. |
| 5952 | T2W.3.lbp.1.9. |
| 5953 | T2W.7.lbp.3.0. |
| 5954 | T2W.7.lbp.3.1. |
| 5955 | T2W.7.lbp.3.2. |
| 5956 | T2W.7.lbp.3.3. |
| 5957 | T2W.7.lbp.3.4. |
| 5958 | T2W.7.lbp.3.5. |
| 5959 | T2W.7.lbp.3.6. |
| 5960 | T2W.7.lbp.3.7. |
| 5961 | T2W.7.lbp.3.8. |
| 5962 | T2W.7.lbp.3.9. |
| 5963 | T2W.11.lbp.5.0. |
| 5964 | T2W.11.lbp.5.1. |
| 5965 | T2W.11.lbp.5.2. |
| 5966 | T2W.11.lbp.5.3. |
| 5967 | T2W.11.lbp.5.4. |
| 5968 | T2W.11.lbp.5.5. |
| 5969 | T2W.11.lbp.5.6. |
| 5970 | T2W.11.lbp.5.7. |
| 5971 | T2W.11.lbp.5.8. |
| 5972 | T2W.11.lbp.5.9. |
| 5973 | T2W.15.lbp.7.0. |
| 5974 | T2W.15.lbp.7.1. |
| 5975 | T2W.15.lbp.7.2. |
| 5976 | T2W.15.lbp.7.3. |
| 5977 | T2W.15.lbp.7.4. |
| 5978 | T2W.15.lbp.7.5. |
| 5979 | T2W.15.lbp.7.6. |
| 5980 | T2W.15.lbp.7.7. |
| 5981 | T2W.15.lbp.7.8. |
| 5982 | T2W.15.lbp.7.9. |
| 5983 | T2W.19.lbp.9.0. |
| 5984 | T2W.19.lbp.9.1. |
| 5985 | T2W.19.lbp.9.2. |
| 5986 | T2W.19.lbp.9.3. |
| 5987 | T2W.19.lbp.9.4. |
| 5988 | T2W.19.lbp.9.5. |
| 5989 | T2W.19.lbp.9.6. |
| 5990 | T2W.19.lbp.9.7. |
| 5991 | T2W.19.lbp.9.8. |
| 5992 | T2W.19.lbp.9.9. |
| 5993 | T2W.23.lbp.11.0. |
| 5994 | T2W.23.lbp.11.1. |
| 5995 | T2W.23.lbp.11.2. |
| 5996 | T2W.23.lbp.11.3. |
| 5997 | T2W.23.lbp.11.4. |
| 5998 | T2W.23.lbp.11.5. |
| 5999 | T2W.23.lbp.11.6. |
| 6000 | T2W.23.lbp.11.7. |
| 6001 | T2W.23.lbp.11.8. |
| 6002 | T2W.23.lbp.11.9. |
| 6003 | T2W.27.lbp.13.0. |
| 6004 | T2W.27.lbp.13.1. |
| 6005 | T2W.27.lbp.13.2. |
| 6006 | T2W.27.lbp.13.3. |
| 6007 | T2W.27.lbp.13.4. |
| 6008 | T2W.27.lbp.13.5. |
| 6009 | T2W.27.lbp.13.6. |
| 6010 | T2W.27.lbp.13.7. |
| 6011 | T2W.27.lbp.13.8. |
| 6012 | T2W.27.lbp.13.9. |
| 6013 | T2W.31.lbp.15.0. |
| 6014 | T2W.31.lbp.15.1. |
| 6015 | T2W.31.lbp.15.2. |
| 6016 | T2W.31.lbp.15.3. |
| 6017 | T2W.31.lbp.15.4. |
| 6018 | T2W.31.lbp.15.5. |
| 6019 | T2W.31.lbp.15.6. |
| 6020 | T2W.31.lbp.15.7. |
| 6021 | T2W.31.lbp.15.8. |
| 6022 | T2W.31.lbp.15.9. |
| 6023 | T2W.35.lbp.17.0. |
| 6024 | T2W.35.lbp.17.1. |
| 6025 | T2W.35.lbp.17.2. |
| 6026 | T2W.35.lbp.17.3. |
| 6027 | T2W.35.lbp.17.4. |
| 6028 | T2W.35.lbp.17.5. |
| 6029 | T2W.35.lbp.17.6. |
| 6030 | T2W.35.lbp.17.7. |
| 6031 | T2W.35.lbp.17.8. |
| 6032 | T2W.35.lbp.17.9. |
| 6033 | T2W.3.hog |
| 6034 | T2W.7.hog |
| 6035 | T2W.11.hog |
| 6036 | T2W.15.hog |
| 6037 | T2W.19.hog |
| 6038 | T2W.23.hog |
| 6039 | T2W.27.hog |
| 6040 | T2W.31.hog |
| 6041 | T2W.35.hog |
| 6042 | T2W.3.gabor.1.0.1.mean. |
| 6043 | T2W.3.gabor.1.0.1.var. |
| 6044 | T2W.3.gabor.1.0.1.absmean. |
| 6045 | T2W.3.gabor.1.0.1.mag. |
| 6046 | T2W.3.gabor.1.0.2.mean. |
| 6047 | T2W.3.gabor.1.0.2.var. |
| 6048 | T2W.3.gabor.1.0.2.absmean. |
| 6049 | T2W.3.gabor.1.0.2.mag. |
| 6050 | T2W.3.gabor.1.0.3.mean. |
| 6051 | T2W.3.gabor.1.0.3.var. |
| 6052 | T2W.3.gabor.1.0.3.absmean. |
| 6053 | T2W.3.gabor.1.0.3.mag. |
| 6054 | T2W.3.gabor.1.0.4.mean. |
| 6055 | T2W.3.gabor.1.0.4.var. |
| 6056 | T2W.3.gabor.1.0.4.absmean. |
| 6057 | T2W.3.gabor.1.0.4.mag. |
| 6058 | T2W.3.gabor.1.0.5.mean. |
| 6059 | T2W.3.gabor.1.0.5.var. |
| 6060 | T2W.3.gabor.1.0.5.absmean. |
| 6061 | T2W.3.gabor.1.0.5.mag. |
| 6062 | T2W.3.gabor.2.0.1.mean. |
| 6063 | T2W.3.gabor.2.0.1.var. |
| 6064 | T2W.3.gabor.2.0.1.absmean. |
| 6065 | T2W.3.gabor.2.0.1.mag. |
| 6066 | T2W.3.gabor.2.0.2.mean. |
| 6067 | T2W.3.gabor.2.0.2.var. |
| 6068 | T2W.3.gabor.2.0.2.absmean. |
| 6069 | T2W.3.gabor.2.0.2.mag. |
| 6070 | T2W.3.gabor.2.0.3.mean. |
| 6071 | T2W.3.gabor.2.0.3.var. |
| 6072 | T2W.3.gabor.2.0.3.absmean. |
| 6073 | T2W.3.gabor.2.0.3.mag. |
| 6074 | T2W.3.gabor.2.0.4.mean. |
| 6075 | T2W.3.gabor.2.0.4.var. |
| 6076 | T2W.3.gabor.2.0.4.absmean. |
| 6077 | T2W.3.gabor.2.0.4.mag. |
| 6078 | T2W.3.gabor.2.0.5.mean. |
| 6079 | T2W.3.gabor.2.0.5.var. |
| 6080 | T2W.3.gabor.2.0.5.absmean. |
| 6081 | T2W.3.gabor.2.0.5.mag. |
| 6082 | T2W.3.gabor.3.0.1.mean. |
| 6083 | T2W.3.gabor.3.0.1.var. |
| 6084 | T2W.3.gabor.3.0.1.absmean. |
| 6085 | T2W.3.gabor.3.0.1.mag. |
| 6086 | T2W.3.gabor.3.0.2.mean. |
| 6087 | T2W.3.gabor.3.0.2.var. |
| 6088 | T2W.3.gabor.3.0.2.absmean. |
| 6089 | T2W.3.gabor.3.0.2.mag. |
| 6090 | T2W.3.gabor.3.0.3.mean. |
| 6091 | T2W.3.gabor.3.0.3.var. |
| 6092 | T2W.3.gabor.3.0.3.absmean. |
| 6093 | T2W.3.gabor.3.0.3.mag. |
| 6094 | T2W.3.gabor.3.0.4.mean. |
| 6095 | T2W.3.gabor.3.0.4.var. |
| 6096 | T2W.3.gabor.3.0.4.absmean. |
| 6097 | T2W.3.gabor.3.0.4.mag. |
| 6098 | T2W.3.gabor.3.0.5.mean. |
| 6099 | T2W.3.gabor.3.0.5.var. |
| 6100 | T2W.3.gabor.3.0.5.absmean. |
| 6101 | T2W.3.gabor.3.0.5.mag. |
| 6102 | T2W.7.gabor.1.0.1.mean. |
| 6103 | T2W.7.gabor.1.0.1.var. |
| 6104 | T2W.7.gabor.1.0.1.absmean. |
| 6105 | T2W.7.gabor.1.0.1.mag. |
| 6106 | T2W.7.gabor.1.0.2.mean. |
| 6107 | T2W.7.gabor.1.0.2.var. |
| 6108 | T2W.7.gabor.1.0.2.absmean. |
| 6109 | T2W.7.gabor.1.0.2.mag. |
| 6110 | T2W.7.gabor.1.0.3.mean. |
| 6111 | T2W.7.gabor.1.0.3.var. |
| 6112 | T2W.7.gabor.1.0.3.absmean. |
| 6113 | T2W.7.gabor.1.0.3.mag. |
| 6114 | T2W.7.gabor.1.0.4.mean. |
| 6115 | T2W.7.gabor.1.0.4.var. |
| 6116 | T2W.7.gabor.1.0.4.absmean. |
| 6117 | T2W.7.gabor.1.0.4.mag. |
| 6118 | T2W.7.gabor.1.0.5.mean. |
| 6119 | T2W.7.gabor.1.0.5.var. |
| 6120 | T2W.7.gabor.1.0.5.absmean. |
| 6121 | T2W.7.gabor.1.0.5.mag. |
| 6122 | T2W.7.gabor.2.0.1.mean. |
| 6123 | T2W.7.gabor.2.0.1.var. |
| 6124 | T2W.7.gabor.2.0.1.absmean. |
| 6125 | T2W.7.gabor.2.0.1.mag. |
| 6126 | T2W.7.gabor.2.0.2.mean. |
| 6127 | T2W.7.gabor.2.0.2.var. |
| 6128 | T2W.7.gabor.2.0.2.absmean. |
| 6129 | T2W.7.gabor.2.0.2.mag. |
| 6130 | T2W.7.gabor.2.0.3.mean. |
| 6131 | T2W.7.gabor.2.0.3.var. |
| 6132 | T2W.7.gabor.2.0.3.absmean. |
| 6133 | T2W.7.gabor.2.0.3.mag. |
| 6134 | T2W.7.gabor.2.0.4.mean. |
| 6135 | T2W.7.gabor.2.0.4.var. |
| 6136 | T2W.7.gabor.2.0.4.absmean. |
| 6137 | T2W.7.gabor.2.0.4.mag. |
| 6138 | T2W.7.gabor.2.0.5.mean. |
| 6139 | T2W.7.gabor.2.0.5.var. |
| 6140 | T2W.7.gabor.2.0.5.absmean. |
| 6141 | T2W.7.gabor.2.0.5.mag. |
| 6142 | T2W.7.gabor.3.0.1.mean. |
| 6143 | T2W.7.gabor.3.0.1.var. |
| 6144 | T2W.7.gabor.3.0.1.absmean. |
| 6145 | T2W.7.gabor.3.0.1.mag. |
| 6146 | T2W.7.gabor.3.0.2.mean. |
| 6147 | T2W.7.gabor.3.0.2.var. |
| 6148 | T2W.7.gabor.3.0.2.absmean. |
| 6149 | T2W.7.gabor.3.0.2.mag. |
| 6150 | T2W.7.gabor.3.0.3.mean. |
| 6151 | T2W.7.gabor.3.0.3.var. |
| 6152 | T2W.7.gabor.3.0.3.absmean. |
| 6153 | T2W.7.gabor.3.0.3.mag. |
| 6154 | T2W.7.gabor.3.0.4.mean. |
| 6155 | T2W.7.gabor.3.0.4.var. |
| 6156 | T2W.7.gabor.3.0.4.absmean. |
| 6157 | T2W.7.gabor.3.0.4.mag. |
| 6158 | T2W.7.gabor.3.0.5.mean. |
| 6159 | T2W.7.gabor.3.0.5.var. |
| 6160 | T2W.7.gabor.3.0.5.absmean. |
| 6161 | T2W.7.gabor.3.0.5.mag. |
| 6162 | T2W.11.gabor.1.0.1.mean. |
| 6163 | T2W.11.gabor.1.0.1.var. |
| 6164 | T2W.11.gabor.1.0.1.absmean. |
| 6165 | T2W.11.gabor.1.0.1.mag. |
| 6166 | T2W.11.gabor.1.0.2.mean. |
| 6167 | T2W.11.gabor.1.0.2.var. |
| 6168 | T2W.11.gabor.1.0.2.absmean. |
| 6169 | T2W.11.gabor.1.0.2.mag. |
| 6170 | T2W.11.gabor.1.0.3.mean. |
| 6171 | T2W.11.gabor.1.0.3.var. |
| 6172 | T2W.11.gabor.1.0.3.absmean. |
| 6173 | T2W.11.gabor.1.0.3.mag. |
| 6174 | T2W.11.gabor.1.0.4.mean. |
| 6175 | T2W.11.gabor.1.0.4.var. |
| 6176 | T2W.11.gabor.1.0.4.absmean. |
| 6177 | T2W.11.gabor.1.0.4.mag. |
| 6178 | T2W.11.gabor.1.0.5.mean. |
| 6179 | T2W.11.gabor.1.0.5.var. |
| 6180 | T2W.11.gabor.1.0.5.absmean. |
| 6181 | T2W.11.gabor.1.0.5.mag. |
| 6182 | T2W.11.gabor.2.0.1.mean. |
| 6183 | T2W.11.gabor.2.0.1.var. |
| 6184 | T2W.11.gabor.2.0.1.absmean. |
| 6185 | T2W.11.gabor.2.0.1.mag. |
| 6186 | T2W.11.gabor.2.0.2.mean. |
| 6187 | T2W.11.gabor.2.0.2.var. |
| 6188 | T2W.11.gabor.2.0.2.absmean. |
| 6189 | T2W.11.gabor.2.0.2.mag. |
| 6190 | T2W.11.gabor.2.0.3.mean. |
| 6191 | T2W.11.gabor.2.0.3.var. |
| 6192 | T2W.11.gabor.2.0.3.absmean. |
| 6193 | T2W.11.gabor.2.0.3.mag. |
| 6194 | T2W.11.gabor.2.0.4.mean. |
| 6195 | T2W.11.gabor.2.0.4.var. |
| 6196 | T2W.11.gabor.2.0.4.absmean. |
| 6197 | T2W.11.gabor.2.0.4.mag. |
| 6198 | T2W.11.gabor.2.0.5.mean. |
| 6199 | T2W.11.gabor.2.0.5.var. |
| 6200 | T2W.11.gabor.2.0.5.absmean. |
| 6201 | T2W.11.gabor.2.0.5.mag. |
| 6202 | T2W.11.gabor.3.0.1.mean. |
| 6203 | T2W.11.gabor.3.0.1.var. |
| 6204 | T2W.11.gabor.3.0.1.absmean. |
| 6205 | T2W.11.gabor.3.0.1.mag. |
| 6206 | T2W.11.gabor.3.0.2.mean. |
| 6207 | T2W.11.gabor.3.0.2.var. |
| 6208 | T2W.11.gabor.3.0.2.absmean. |
| 6209 | T2W.11.gabor.3.0.2.mag. |
| 6210 | T2W.11.gabor.3.0.3.mean. |
| 6211 | T2W.11.gabor.3.0.3.var. |
| 6212 | T2W.11.gabor.3.0.3.absmean. |
| 6213 | T2W.11.gabor.3.0.3.mag. |
| 6214 | T2W.11.gabor.3.0.4.mean. |
| 6215 | T2W.11.gabor.3.0.4.var. |
| 6216 | T2W.11.gabor.3.0.4.absmean. |
| 6217 | T2W.11.gabor.3.0.4.mag. |
| 6218 | T2W.11.gabor.3.0.5.mean. |
| 6219 | T2W.11.gabor.3.0.5.var. |
| 6220 | T2W.11.gabor.3.0.5.absmean. |
| 6221 | T2W.11.gabor.3.0.5.mag. |
| 6222 | T2W.15.gabor.1.0.1.mean. |
| 6223 | T2W.15.gabor.1.0.1.var. |
| 6224 | T2W.15.gabor.1.0.1.absmean. |
| 6225 | T2W.15.gabor.1.0.1.mag. |
| 6226 | T2W.15.gabor.1.0.2.mean. |
| 6227 | T2W.15.gabor.1.0.2.var. |
| 6228 | T2W.15.gabor.1.0.2.absmean. |
| 6229 | T2W.15.gabor.1.0.2.mag. |
| 6230 | T2W.15.gabor.1.0.3.mean. |
| 6231 | T2W.15.gabor.1.0.3.var. |
| 6232 | T2W.15.gabor.1.0.3.absmean. |
| 6233 | T2W.15.gabor.1.0.3.mag. |
| 6234 | T2W.15.gabor.1.0.4.mean. |
| 6235 | T2W.15.gabor.1.0.4.var. |
| 6236 | T2W.15.gabor.1.0.4.absmean. |
| 6237 | T2W.15.gabor.1.0.4.mag. |
| 6238 | T2W.15.gabor.1.0.5.mean. |
| 6239 | T2W.15.gabor.1.0.5.var. |
| 6240 | T2W.15.gabor.1.0.5.absmean. |
| 6241 | T2W.15.gabor.1.0.5.mag. |
| 6242 | T2W.15.gabor.2.0.1.mean. |
| 6243 | T2W.15.gabor.2.0.1.var. |
| 6244 | T2W.15.gabor.2.0.1.absmean. |
| 6245 | T2W.15.gabor.2.0.1.mag. |
| 6246 | T2W.15.gabor.2.0.2.mean. |
| 6247 | T2W.15.gabor.2.0.2.var. |
| 6248 | T2W.15.gabor.2.0.2.absmean. |
| 6249 | T2W.15.gabor.2.0.2.mag. |
| 6250 | T2W.15.gabor.2.0.3.mean. |
| 6251 | T2W.15.gabor.2.0.3.var. |
| 6252 | T2W.15.gabor.2.0.3.absmean. |
| 6253 | T2W.15.gabor.2.0.3.mag. |
| 6254 | T2W.15.gabor.2.0.4.mean. |
| 6255 | T2W.15.gabor.2.0.4.var. |
| 6256 | T2W.15.gabor.2.0.4.absmean. |
| 6257 | T2W.15.gabor.2.0.4.mag. |
| 6258 | T2W.15.gabor.2.0.5.mean. |
| 6259 | T2W.15.gabor.2.0.5.var. |
| 6260 | T2W.15.gabor.2.0.5.absmean. |
| 6261 | T2W.15.gabor.2.0.5.mag. |
| 6262 | T2W.15.gabor.3.0.1.mean. |
| 6263 | T2W.15.gabor.3.0.1.var. |
| 6264 | T2W.15.gabor.3.0.1.absmean. |
| 6265 | T2W.15.gabor.3.0.1.mag. |
| 6266 | T2W.15.gabor.3.0.2.mean. |
| 6267 | T2W.15.gabor.3.0.2.var. |
| 6268 | T2W.15.gabor.3.0.2.absmean. |
| 6269 | T2W.15.gabor.3.0.2.mag. |
| 6270 | T2W.15.gabor.3.0.3.mean. |
| 6271 | T2W.15.gabor.3.0.3.var. |
| 6272 | T2W.15.gabor.3.0.3.absmean. |
| 6273 | T2W.15.gabor.3.0.3.mag. |
| 6274 | T2W.15.gabor.3.0.4.mean. |
| 6275 | T2W.15.gabor.3.0.4.var. |
| 6276 | T2W.15.gabor.3.0.4.absmean. |
| 6277 | T2W.15.gabor.3.0.4.mag. |
| 6278 | T2W.15.gabor.3.0.5.mean. |
| 6279 | T2W.15.gabor.3.0.5.var. |
| 6280 | T2W.15.gabor.3.0.5.absmean. |
| 6281 | T2W.15.gabor.3.0.5.mag. |
| 6282 | T2W.19.gabor.1.0.1.mean. |
| 6283 | T2W.19.gabor.1.0.1.var. |
| 6284 | T2W.19.gabor.1.0.1.absmean. |
| 6285 | T2W.19.gabor.1.0.1.mag. |
| 6286 | T2W.19.gabor.1.0.2.mean. |
| 6287 | T2W.19.gabor.1.0.2.var. |
| 6288 | T2W.19.gabor.1.0.2.absmean. |
| 6289 | T2W.19.gabor.1.0.2.mag. |
| 6290 | T2W.19.gabor.1.0.3.mean. |
| 6291 | T2W.19.gabor.1.0.3.var. |
| 6292 | T2W.19.gabor.1.0.3.absmean. |
| 6293 | T2W.19.gabor.1.0.3.mag. |
| 6294 | T2W.19.gabor.1.0.4.mean. |
| 6295 | T2W.19.gabor.1.0.4.var. |
| 6296 | T2W.19.gabor.1.0.4.absmean. |
| 6297 | T2W.19.gabor.1.0.4.mag. |
| 6298 | T2W.19.gabor.1.0.5.mean. |
| 6299 | T2W.19.gabor.1.0.5.var. |
| 6300 | T2W.19.gabor.1.0.5.absmean. |
| 6301 | T2W.19.gabor.1.0.5.mag. |
| 6302 | T2W.19.gabor.2.0.1.mean. |
| 6303 | T2W.19.gabor.2.0.1.var. |
| 6304 | T2W.19.gabor.2.0.1.absmean. |
| 6305 | T2W.19.gabor.2.0.1.mag. |
| 6306 | T2W.19.gabor.2.0.2.mean. |
| 6307 | T2W.19.gabor.2.0.2.var. |
| 6308 | T2W.19.gabor.2.0.2.absmean. |
| 6309 | T2W.19.gabor.2.0.2.mag. |
| 6310 | T2W.19.gabor.2.0.3.mean. |
| 6311 | T2W.19.gabor.2.0.3.var. |
| 6312 | T2W.19.gabor.2.0.3.absmean. |
| 6313 | T2W.19.gabor.2.0.3.mag. |
| 6314 | T2W.19.gabor.2.0.4.mean. |
| 6315 | T2W.19.gabor.2.0.4.var. |
| 6316 | T2W.19.gabor.2.0.4.absmean. |
| 6317 | T2W.19.gabor.2.0.4.mag. |
| 6318 | T2W.19.gabor.2.0.5.mean. |
| 6319 | T2W.19.gabor.2.0.5.var. |
| 6320 | T2W.19.gabor.2.0.5.absmean. |
| 6321 | T2W.19.gabor.2.0.5.mag. |
| 6322 | T2W.19.gabor.3.0.1.mean. |
| 6323 | T2W.19.gabor.3.0.1.var. |
| 6324 | T2W.19.gabor.3.0.1.absmean. |
| 6325 | T2W.19.gabor.3.0.1.mag. |
| 6326 | T2W.19.gabor.3.0.2.mean. |
| 6327 | T2W.19.gabor.3.0.2.var. |
| 6328 | T2W.19.gabor.3.0.2.absmean. |
| 6329 | T2W.19.gabor.3.0.2.mag. |
| 6330 | T2W.19.gabor.3.0.3.mean. |
| 6331 | T2W.19.gabor.3.0.3.var. |
| 6332 | T2W.19.gabor.3.0.3.absmean. |
| 6333 | T2W.19.gabor.3.0.3.mag. |
| 6334 | T2W.19.gabor.3.0.4.mean. |
| 6335 | T2W.19.gabor.3.0.4.var. |
| 6336 | T2W.19.gabor.3.0.4.absmean. |
| 6337 | T2W.19.gabor.3.0.4.mag. |
| 6338 | T2W.19.gabor.3.0.5.mean. |
| 6339 | T2W.19.gabor.3.0.5.var. |
| 6340 | T2W.19.gabor.3.0.5.absmean. |
| 6341 | T2W.19.gabor.3.0.5.mag. |
| 6342 | T2W.23.gabor.1.0.1.mean. |
| 6343 | T2W.23.gabor.1.0.1.var. |
| 6344 | T2W.23.gabor.1.0.1.absmean. |
| 6345 | T2W.23.gabor.1.0.1.mag. |
| 6346 | T2W.23.gabor.1.0.2.mean. |
| 6347 | T2W.23.gabor.1.0.2.var. |
| 6348 | T2W.23.gabor.1.0.2.absmean. |
| 6349 | T2W.23.gabor.1.0.2.mag. |
| 6350 | T2W.23.gabor.1.0.3.mean. |
| 6351 | T2W.23.gabor.1.0.3.var. |
| 6352 | T2W.23.gabor.1.0.3.absmean. |
| 6353 | T2W.23.gabor.1.0.3.mag. |
| 6354 | T2W.23.gabor.1.0.4.mean. |
| 6355 | T2W.23.gabor.1.0.4.var. |
| 6356 | T2W.23.gabor.1.0.4.absmean. |
| 6357 | T2W.23.gabor.1.0.4.mag. |
| 6358 | T2W.23.gabor.1.0.5.mean. |
| 6359 | T2W.23.gabor.1.0.5.var. |
| 6360 | T2W.23.gabor.1.0.5.absmean. |
| 6361 | T2W.23.gabor.1.0.5.mag. |
| 6362 | T2W.23.gabor.2.0.1.mean. |
| 6363 | T2W.23.gabor.2.0.1.var. |
| 6364 | T2W.23.gabor.2.0.1.absmean. |
| 6365 | T2W.23.gabor.2.0.1.mag. |
| 6366 | T2W.23.gabor.2.0.2.mean. |
| 6367 | T2W.23.gabor.2.0.2.var. |
| 6368 | T2W.23.gabor.2.0.2.absmean. |
| 6369 | T2W.23.gabor.2.0.2.mag. |
| 6370 | T2W.23.gabor.2.0.3.mean. |
| 6371 | T2W.23.gabor.2.0.3.var. |
| 6372 | T2W.23.gabor.2.0.3.absmean. |
| 6373 | T2W.23.gabor.2.0.3.mag. |
| 6374 | T2W.23.gabor.2.0.4.mean. |
| 6375 | T2W.23.gabor.2.0.4.var. |
| 6376 | T2W.23.gabor.2.0.4.absmean. |
| 6377 | T2W.23.gabor.2.0.4.mag. |
| 6378 | T2W.23.gabor.2.0.5.mean. |
| 6379 | T2W.23.gabor.2.0.5.var. |
| 6380 | T2W.23.gabor.2.0.5.absmean. |
| 6381 | T2W.23.gabor.2.0.5.mag. |
| 6382 | T2W.23.gabor.3.0.1.mean. |
| 6383 | T2W.23.gabor.3.0.1.var. |
| 6384 | T2W.23.gabor.3.0.1.absmean. |
| 6385 | T2W.23.gabor.3.0.1.mag. |
| 6386 | T2W.23.gabor.3.0.2.mean. |
| 6387 | T2W.23.gabor.3.0.2.var. |
| 6388 | T2W.23.gabor.3.0.2.absmean. |
| 6389 | T2W.23.gabor.3.0.2.mag. |
| 6390 | T2W.23.gabor.3.0.3.mean. |
| 6391 | T2W.23.gabor.3.0.3.var. |
| 6392 | T2W.23.gabor.3.0.3.absmean. |
| 6393 | T2W.23.gabor.3.0.3.mag. |
| 6394 | T2W.23.gabor.3.0.4.mean. |
| 6395 | T2W.23.gabor.3.0.4.var. |
| 6396 | T2W.23.gabor.3.0.4.absmean. |
| 6397 | T2W.23.gabor.3.0.4.mag. |
| 6398 | T2W.23.gabor.3.0.5.mean. |
| 6399 | T2W.23.gabor.3.0.5.var. |
| 6400 | T2W.23.gabor.3.0.5.absmean. |
| 6401 | T2W.23.gabor.3.0.5.mag. |
| 6402 | T2W.27.gabor.1.0.1.mean. |
| 6403 | T2W.27.gabor.1.0.1.var. |
| 6404 | T2W.27.gabor.1.0.1.absmean. |
| 6405 | T2W.27.gabor.1.0.1.mag. |
| 6406 | T2W.27.gabor.1.0.2.mean. |
| 6407 | T2W.27.gabor.1.0.2.var. |
| 6408 | T2W.27.gabor.1.0.2.absmean. |
| 6409 | T2W.27.gabor.1.0.2.mag. |
| 6410 | T2W.27.gabor.1.0.3.mean. |
| 6411 | T2W.27.gabor.1.0.3.var. |
| 6412 | T2W.27.gabor.1.0.3.absmean. |
| 6413 | T2W.27.gabor.1.0.3.mag. |
| 6414 | T2W.27.gabor.1.0.4.mean. |
| 6415 | T2W.27.gabor.1.0.4.var. |
| 6416 | T2W.27.gabor.1.0.4.absmean. |
| 6417 | T2W.27.gabor.1.0.4.mag. |
| 6418 | T2W.27.gabor.1.0.5.mean. |
| 6419 | T2W.27.gabor.1.0.5.var. |
| 6420 | T2W.27.gabor.1.0.5.absmean. |
| 6421 | T2W.27.gabor.1.0.5.mag. |
| 6422 | T2W.27.gabor.2.0.1.mean. |
| 6423 | T2W.27.gabor.2.0.1.var. |
| 6424 | T2W.27.gabor.2.0.1.absmean. |
| 6425 | T2W.27.gabor.2.0.1.mag. |
| 6426 | T2W.27.gabor.2.0.2.mean. |
| 6427 | T2W.27.gabor.2.0.2.var. |
| 6428 | T2W.27.gabor.2.0.2.absmean. |
| 6429 | T2W.27.gabor.2.0.2.mag. |
| 6430 | T2W.27.gabor.2.0.3.mean. |
| 6431 | T2W.27.gabor.2.0.3.var. |
| 6432 | T2W.27.gabor.2.0.3.absmean. |
| 6433 | T2W.27.gabor.2.0.3.mag. |
| 6434 | T2W.27.gabor.2.0.4.mean. |
| 6435 | T2W.27.gabor.2.0.4.var. |
| 6436 | T2W.27.gabor.2.0.4.absmean. |
| 6437 | T2W.27.gabor.2.0.4.mag. |
| 6438 | T2W.27.gabor.2.0.5.mean. |
| 6439 | T2W.27.gabor.2.0.5.var. |
| 6440 | T2W.27.gabor.2.0.5.absmean. |
| 6441 | T2W.27.gabor.2.0.5.mag. |
| 6442 | T2W.27.gabor.3.0.1.mean. |
| 6443 | T2W.27.gabor.3.0.1.var. |
| 6444 | T2W.27.gabor.3.0.1.absmean. |
| 6445 | T2W.27.gabor.3.0.1.mag. |
| 6446 | T2W.27.gabor.3.0.2.mean. |
| 6447 | T2W.27.gabor.3.0.2.var. |
| 6448 | T2W.27.gabor.3.0.2.absmean. |
| 6449 | T2W.27.gabor.3.0.2.mag. |
| 6450 | T2W.27.gabor.3.0.3.mean. |
| 6451 | T2W.27.gabor.3.0.3.var. |
| 6452 | T2W.27.gabor.3.0.3.absmean. |
| 6453 | T2W.27.gabor.3.0.3.mag. |
| 6454 | T2W.27.gabor.3.0.4.mean. |
| 6455 | T2W.27.gabor.3.0.4.var. |
| 6456 | T2W.27.gabor.3.0.4.absmean. |
| 6457 | T2W.27.gabor.3.0.4.mag. |
| 6458 | T2W.27.gabor.3.0.5.mean. |
| 6459 | T2W.27.gabor.3.0.5.var. |
| 6460 | T2W.27.gabor.3.0.5.absmean. |
| 6461 | T2W.27.gabor.3.0.5.mag. |
| 6462 | T2W.31.gabor.1.0.1.mean. |
| 6463 | T2W.31.gabor.1.0.1.var. |
| 6464 | T2W.31.gabor.1.0.1.absmean. |
| 6465 | T2W.31.gabor.1.0.1.mag. |
| 6466 | T2W.31.gabor.1.0.2.mean. |
| 6467 | T2W.31.gabor.1.0.2.var. |
| 6468 | T2W.31.gabor.1.0.2.absmean. |
| 6469 | T2W.31.gabor.1.0.2.mag. |
| 6470 | T2W.31.gabor.1.0.3.mean. |
| 6471 | T2W.31.gabor.1.0.3.var. |
| 6472 | T2W.31.gabor.1.0.3.absmean. |
| 6473 | T2W.31.gabor.1.0.3.mag. |
| 6474 | T2W.31.gabor.1.0.4.mean. |
| 6475 | T2W.31.gabor.1.0.4.var. |
| 6476 | T2W.31.gabor.1.0.4.absmean. |
| 6477 | T2W.31.gabor.1.0.4.mag. |
| 6478 | T2W.31.gabor.1.0.5.mean. |
| 6479 | T2W.31.gabor.1.0.5.var. |
| 6480 | T2W.31.gabor.1.0.5.absmean. |
| 6481 | T2W.31.gabor.1.0.5.mag. |
| 6482 | T2W.31.gabor.2.0.1.mean. |
| 6483 | T2W.31.gabor.2.0.1.var. |
| 6484 | T2W.31.gabor.2.0.1.absmean. |
| 6485 | T2W.31.gabor.2.0.1.mag. |
| 6486 | T2W.31.gabor.2.0.2.mean. |
| 6487 | T2W.31.gabor.2.0.2.var. |
| 6488 | T2W.31.gabor.2.0.2.absmean. |
| 6489 | T2W.31.gabor.2.0.2.mag. |
| 6490 | T2W.31.gabor.2.0.3.mean. |
| 6491 | T2W.31.gabor.2.0.3.var. |
| 6492 | T2W.31.gabor.2.0.3.absmean. |
| 6493 | T2W.31.gabor.2.0.3.mag. |
| 6494 | T2W.31.gabor.2.0.4.mean. |
| 6495 | T2W.31.gabor.2.0.4.var. |
| 6496 | T2W.31.gabor.2.0.4.absmean. |
| 6497 | T2W.31.gabor.2.0.4.mag. |
| 6498 | T2W.31.gabor.2.0.5.mean. |
| 6499 | T2W.31.gabor.2.0.5.var. |
| 6500 | T2W.31.gabor.2.0.5.absmean. |
| 6501 | T2W.31.gabor.2.0.5.mag. |
| 6502 | T2W.31.gabor.3.0.1.mean. |
| 6503 | T2W.31.gabor.3.0.1.var. |
| 6504 | T2W.31.gabor.3.0.1.absmean. |
| 6505 | T2W.31.gabor.3.0.1.mag. |
| 6506 | T2W.31.gabor.3.0.2.mean. |
| 6507 | T2W.31.gabor.3.0.2.var. |
| 6508 | T2W.31.gabor.3.0.2.absmean. |
| 6509 | T2W.31.gabor.3.0.2.mag. |
| 6510 | T2W.31.gabor.3.0.3.mean. |
| 6511 | T2W.31.gabor.3.0.3.var. |
| 6512 | T2W.31.gabor.3.0.3.absmean. |
| 6513 | T2W.31.gabor.3.0.3.mag. |
| 6514 | T2W.31.gabor.3.0.4.mean. |
| 6515 | T2W.31.gabor.3.0.4.var. |
| 6516 | T2W.31.gabor.3.0.4.absmean. |
| 6517 | T2W.31.gabor.3.0.4.mag. |
| 6518 | T2W.31.gabor.3.0.5.mean. |
| 6519 | T2W.31.gabor.3.0.5.var. |
| 6520 | T2W.31.gabor.3.0.5.absmean. |
| 6521 | T2W.31.gabor.3.0.5.mag. |
| 6522 | T2W.35.gabor.1.0.1.mean. |
| 6523 | T2W.35.gabor.1.0.1.var. |
| 6524 | T2W.35.gabor.1.0.1.absmean. |
| 6525 | T2W.35.gabor.1.0.1.mag. |
| 6526 | T2W.35.gabor.1.0.2.mean. |
| 6527 | T2W.35.gabor.1.0.2.var. |
| 6528 | T2W.35.gabor.1.0.2.absmean. |
| 6529 | T2W.35.gabor.1.0.2.mag. |
| 6530 | T2W.35.gabor.1.0.3.mean. |
| 6531 | T2W.35.gabor.1.0.3.var. |
| 6532 | T2W.35.gabor.1.0.3.absmean. |
| 6533 | T2W.35.gabor.1.0.3.mag. |
| 6534 | T2W.35.gabor.1.0.4.mean. |
| 6535 | T2W.35.gabor.1.0.4.var. |
| 6536 | T2W.35.gabor.1.0.4.absmean. |
| 6537 | T2W.35.gabor.1.0.4.mag. |
| 6538 | T2W.35.gabor.1.0.5.mean. |
| 6539 | T2W.35.gabor.1.0.5.var. |
| 6540 | T2W.35.gabor.1.0.5.absmean. |
| 6541 | T2W.35.gabor.1.0.5.mag. |
| 6542 | T2W.35.gabor.2.0.1.mean. |
| 6543 | T2W.35.gabor.2.0.1.var. |
| 6544 | T2W.35.gabor.2.0.1.absmean. |
| 6545 | T2W.35.gabor.2.0.1.mag. |
| 6546 | T2W.35.gabor.2.0.2.mean. |
| 6547 | T2W.35.gabor.2.0.2.var. |
| 6548 | T2W.35.gabor.2.0.2.absmean. |
| 6549 | T2W.35.gabor.2.0.2.mag. |
| 6550 | T2W.35.gabor.2.0.3.mean. |
| 6551 | T2W.35.gabor.2.0.3.var. |
| 6552 | T2W.35.gabor.2.0.3.absmean. |
| 6553 | T2W.35.gabor.2.0.3.mag. |
| 6554 | T2W.35.gabor.2.0.4.mean. |
| 6555 | T2W.35.gabor.2.0.4.var. |
| 6556 | T2W.35.gabor.2.0.4.absmean. |
| 6557 | T2W.35.gabor.2.0.4.mag. |
| 6558 | T2W.35.gabor.2.0.5.mean. |
| 6559 | T2W.35.gabor.2.0.5.var. |
| 6560 | T2W.35.gabor.2.0.5.absmean. |
| 6561 | T2W.35.gabor.2.0.5.mag. |
| 6562 | T2W.35.gabor.3.0.1.mean. |
| 6563 | T2W.35.gabor.3.0.1.var. |
| 6564 | T2W.35.gabor.3.0.1.absmean. |
| 6565 | T2W.35.gabor.3.0.1.mag. |
| 6566 | T2W.35.gabor.3.0.2.mean. |
| 6567 | T2W.35.gabor.3.0.2.var. |
| 6568 | T2W.35.gabor.3.0.2.absmean. |
| 6569 | T2W.35.gabor.3.0.2.mag. |
| 6570 | T2W.35.gabor.3.0.3.mean. |
| 6571 | T2W.35.gabor.3.0.3.var. |
| 6572 | T2W.35.gabor.3.0.3.absmean. |
| 6573 | T2W.35.gabor.3.0.3.mag. |
| 6574 | T2W.35.gabor.3.0.4.mean. |
| 6575 | T2W.35.gabor.3.0.4.var. |
| 6576 | T2W.35.gabor.3.0.4.absmean. |
| 6577 | T2W.35.gabor.3.0.4.mag. |
| 6578 | T2W.35.gabor.3.0.5.mean. |
| 6579 | T2W.35.gabor.3.0.5.var. |
| 6580 | T2W.35.gabor.3.0.5.absmean. |
| 6581 | T2W.35.gabor.3.0.5.mag. |
| 6582 | T2W.3.haar.1.1.aav. |
| 6583 | T2W.3.haar.1.1.std. |
| 6584 | T2W.3.haar.1.2.aav. |
| 6585 | T2W.3.haar.1.2.std. |
| 6586 | T2W.3.haar.1.3.aav. |
| 6587 | T2W.3.haar.1.3.std. |
| 6588 | T2W.3.haar.2.1.aav. |
| 6589 | T2W.3.haar.2.1.std. |
| 6590 | T2W.3.haar.2.2.aav. |
| 6591 | T2W.3.haar.2.2.std. |
| 6592 | T2W.3.haar.2.3.aav. |
| 6593 | T2W.3.haar.2.3.std. |
| 6594 | T2W.3.haar.3.1.aav. |
| 6595 | T2W.3.haar.3.1.std. |
| 6596 | T2W.3.haar.3.2.aav. |
| 6597 | T2W.3.haar.3.2.std. |
| 6598 | T2W.3.haar.3.3.aav. |
| 6599 | T2W.3.haar.3.3.std. |
| 6600 | T2W.3.haar.4.1.aav. |
| 6601 | T2W.3.haar.4.1.std. |
| 6602 | T2W.3.haar.4.2.aav. |
| 6603 | T2W.3.haar.4.2.std. |
| 6604 | T2W.3.haar.4.3.aav. |
| 6605 | T2W.3.haar.4.3.std. |
| 6606 | T2W.7.haar.1.1.aav. |
| 6607 | T2W.7.haar.1.1.std. |
| 6608 | T2W.7.haar.1.2.aav. |
| 6609 | T2W.7.haar.1.2.std. |
| 6610 | T2W.7.haar.1.3.aav. |
| 6611 | T2W.7.haar.1.3.std. |
| 6612 | T2W.7.haar.2.1.aav. |
| 6613 | T2W.7.haar.2.1.std. |
| 6614 | T2W.7.haar.2.2.aav. |
| 6615 | T2W.7.haar.2.2.std. |
| 6616 | T2W.7.haar.2.3.aav. |
| 6617 | T2W.7.haar.2.3.std. |
| 6618 | T2W.7.haar.3.1.aav. |
| 6619 | T2W.7.haar.3.1.std. |
| 6620 | T2W.7.haar.3.2.aav. |
| 6621 | T2W.7.haar.3.2.std. |
| 6622 | T2W.7.haar.3.3.aav. |
| 6623 | T2W.7.haar.3.3.std. |
| 6624 | T2W.7.haar.4.1.aav. |
| 6625 | T2W.7.haar.4.1.std. |
| 6626 | T2W.7.haar.4.2.aav. |
| 6627 | T2W.7.haar.4.2.std. |
| 6628 | T2W.7.haar.4.3.aav. |
| 6629 | T2W.7.haar.4.3.std. |
| 6630 | T2W.11.haar.1.1.aav. |
| 6631 | T2W.11.haar.1.1.std. |
| 6632 | T2W.11.haar.1.2.aav. |
| 6633 | T2W.11.haar.1.2.std. |
| 6634 | T2W.11.haar.1.3.aav. |
| 6635 | T2W.11.haar.1.3.std. |
| 6636 | T2W.11.haar.2.1.aav. |
| 6637 | T2W.11.haar.2.1.std. |
| 6638 | T2W.11.haar.2.2.aav. |
| 6639 | T2W.11.haar.2.2.std. |
| 6640 | T2W.11.haar.2.3.aav. |
| 6641 | T2W.11.haar.2.3.std. |
| 6642 | T2W.11.haar.3.1.aav. |
| 6643 | T2W.11.haar.3.1.std. |
| 6644 | T2W.11.haar.3.2.aav. |
| 6645 | T2W.11.haar.3.2.std. |
| 6646 | T2W.11.haar.3.3.aav. |
| 6647 | T2W.11.haar.3.3.std. |
| 6648 | T2W.11.haar.4.1.aav. |
| 6649 | T2W.11.haar.4.1.std. |
| 6650 | T2W.11.haar.4.2.aav. |
| 6651 | T2W.11.haar.4.2.std. |
| 6652 | T2W.11.haar.4.3.aav. |
| 6653 | T2W.11.haar.4.3.std. |
| 6654 | T2W.15.haar.1.1.aav. |
| 6655 | T2W.15.haar.1.1.std. |
| 6656 | T2W.15.haar.1.2.aav. |
| 6657 | T2W.15.haar.1.2.std. |
| 6658 | T2W.15.haar.1.3.aav. |
| 6659 | T2W.15.haar.1.3.std. |
| 6660 | T2W.15.haar.2.1.aav. |
| 6661 | T2W.15.haar.2.1.std. |
| 6662 | T2W.15.haar.2.2.aav. |
| 6663 | T2W.15.haar.2.2.std. |
| 6664 | T2W.15.haar.2.3.aav. |
| 6665 | T2W.15.haar.2.3.std. |
| 6666 | T2W.15.haar.3.1.aav. |
| 6667 | T2W.15.haar.3.1.std. |
| 6668 | T2W.15.haar.3.2.aav. |
| 6669 | T2W.15.haar.3.2.std. |
| 6670 | T2W.15.haar.3.3.aav. |
| 6671 | T2W.15.haar.3.3.std. |
| 6672 | T2W.15.haar.4.1.aav. |
| 6673 | T2W.15.haar.4.1.std. |
| 6674 | T2W.15.haar.4.2.aav. |
| 6675 | T2W.15.haar.4.2.std. |
| 6676 | T2W.15.haar.4.3.aav. |
| 6677 | T2W.15.haar.4.3.std. |
| 6678 | T2W.19.haar.1.1.aav. |
| 6679 | T2W.19.haar.1.1.std. |
| 6680 | T2W.19.haar.1.2.aav. |
| 6681 | T2W.19.haar.1.2.std. |
| 6682 | T2W.19.haar.1.3.aav. |
| 6683 | T2W.19.haar.1.3.std. |
| 6684 | T2W.19.haar.2.1.aav. |
| 6685 | T2W.19.haar.2.1.std. |
| 6686 | T2W.19.haar.2.2.aav. |
| 6687 | T2W.19.haar.2.2.std. |
| 6688 | T2W.19.haar.2.3.aav. |
| 6689 | T2W.19.haar.2.3.std. |
| 6690 | T2W.19.haar.3.1.aav. |
| 6691 | T2W.19.haar.3.1.std. |
| 6692 | T2W.19.haar.3.2.aav. |
| 6693 | T2W.19.haar.3.2.std. |
| 6694 | T2W.19.haar.3.3.aav. |
| 6695 | T2W.19.haar.3.3.std. |
| 6696 | T2W.19.haar.4.1.aav. |
| 6697 | T2W.19.haar.4.1.std. |
| 6698 | T2W.19.haar.4.2.aav. |
| 6699 | T2W.19.haar.4.2.std. |
| 6700 | T2W.19.haar.4.3.aav. |
| 6701 | T2W.19.haar.4.3.std. |
| 6702 | T2W.23.haar.1.1.aav. |
| 6703 | T2W.23.haar.1.1.std. |
| 6704 | T2W.23.haar.1.2.aav. |
| 6705 | T2W.23.haar.1.2.std. |
| 6706 | T2W.23.haar.1.3.aav. |
| 6707 | T2W.23.haar.1.3.std. |
| 6708 | T2W.23.haar.2.1.aav. |
| 6709 | T2W.23.haar.2.1.std. |
| 6710 | T2W.23.haar.2.2.aav. |
| 6711 | T2W.23.haar.2.2.std. |
| 6712 | T2W.23.haar.2.3.aav. |
| 6713 | T2W.23.haar.2.3.std. |
| 6714 | T2W.23.haar.3.1.aav. |
| 6715 | T2W.23.haar.3.1.std. |
| 6716 | T2W.23.haar.3.2.aav. |
| 6717 | T2W.23.haar.3.2.std. |
| 6718 | T2W.23.haar.3.3.aav. |
| 6719 | T2W.23.haar.3.3.std. |
| 6720 | T2W.23.haar.4.1.aav. |
| 6721 | T2W.23.haar.4.1.std. |
| 6722 | T2W.23.haar.4.2.aav. |
| 6723 | T2W.23.haar.4.2.std. |
| 6724 | T2W.23.haar.4.3.aav. |
| 6725 | T2W.23.haar.4.3.std. |
| 6726 | T2W.27.haar.1.1.aav. |
| 6727 | T2W.27.haar.1.1.std. |
| 6728 | T2W.27.haar.1.2.aav. |
| 6729 | T2W.27.haar.1.2.std. |
| 6730 | T2W.27.haar.1.3.aav. |
| 6731 | T2W.27.haar.1.3.std. |
| 6732 | T2W.27.haar.2.1.aav. |
| 6733 | T2W.27.haar.2.1.std. |
| 6734 | T2W.27.haar.2.2.aav. |
| 6735 | T2W.27.haar.2.2.std. |
| 6736 | T2W.27.haar.2.3.aav. |
| 6737 | T2W.27.haar.2.3.std. |
| 6738 | T2W.27.haar.3.1.aav. |
| 6739 | T2W.27.haar.3.1.std. |
| 6740 | T2W.27.haar.3.2.aav. |
| 6741 | T2W.27.haar.3.2.std. |
| 6742 | T2W.27.haar.3.3.aav. |
| 6743 | T2W.27.haar.3.3.std. |
| 6744 | T2W.27.haar.4.1.aav. |
| 6745 | T2W.27.haar.4.1.std. |
| 6746 | T2W.27.haar.4.2.aav. |
| 6747 | T2W.27.haar.4.2.std. |
| 6748 | T2W.27.haar.4.3.aav. |
| 6749 | T2W.27.haar.4.3.std. |
| 6750 | T2W.31.haar.1.1.aav. |
| 6751 | T2W.31.haar.1.1.std. |
| 6752 | T2W.31.haar.1.2.aav. |
| 6753 | T2W.31.haar.1.2.std. |
| 6754 | T2W.31.haar.1.3.aav. |
| 6755 | T2W.31.haar.1.3.std. |
| 6756 | T2W.31.haar.2.1.aav. |
| 6757 | T2W.31.haar.2.1.std. |
| 6758 | T2W.31.haar.2.2.aav. |
| 6759 | T2W.31.haar.2.2.std. |
| 6760 | T2W.31.haar.2.3.aav. |
| 6761 | T2W.31.haar.2.3.std. |
| 6762 | T2W.31.haar.3.1.aav. |
| 6763 | T2W.31.haar.3.1.std. |
| 6764 | T2W.31.haar.3.2.aav. |
| 6765 | T2W.31.haar.3.2.std. |
| 6766 | T2W.31.haar.3.3.aav. |
| 6767 | T2W.31.haar.3.3.std. |
| 6768 | T2W.31.haar.4.1.aav. |
| 6769 | T2W.31.haar.4.1.std. |
| 6770 | T2W.31.haar.4.2.aav. |
| 6771 | T2W.31.haar.4.2.std. |
| 6772 | T2W.31.haar.4.3.aav. |
| 6773 | T2W.31.haar.4.3.std. |
| 6774 | T2W.35.haar.1.1.aav. |
| 6775 | T2W.35.haar.1.1.std. |
| 6776 | T2W.35.haar.1.2.aav. |
| 6777 | T2W.35.haar.1.2.std. |
| 6778 | T2W.35.haar.1.3.aav. |
| 6779 | T2W.35.haar.1.3.std. |
| 6780 | T2W.35.haar.2.1.aav. |
| 6781 | T2W.35.haar.2.1.std. |
| 6782 | T2W.35.haar.2.2.aav. |
| 6783 | T2W.35.haar.2.2.std. |
| 6784 | T2W.35.haar.2.3.aav. |
| 6785 | T2W.35.haar.2.3.std. |
| 6786 | T2W.35.haar.3.1.aav. |
| 6787 | T2W.35.haar.3.1.std. |
| 6788 | T2W.35.haar.3.2.aav. |
| 6789 | T2W.35.haar.3.2.std. |
| 6790 | T2W.35.haar.3.3.aav. |
| 6791 | T2W.35.haar.3.3.std. |
| 6792 | T2W.35.haar.4.1.aav. |
| 6793 | T2W.35.haar.4.1.std. |
| 6794 | T2W.35.haar.4.2.aav. |
| 6795 | T2W.35.haar.4.2.std. |
| 6796 | T2W.35.haar.4.3.aav. |
| 6797 | T2W.35.haar.4.3.std. |
| 6798 | T2W.3.hu.0. |
| 6799 | T2W.3.hu.1. |
| 6800 | T2W.3.hu.2. |
| 6801 | T2W.3.hu.3. |
| 6802 | T2W.3.hu.4. |
| 6803 | T2W.3.hu.5. |
| 6804 | T2W.3.hu.6. |
| 6805 | T2W.7.hu.0. |
| 6806 | T2W.7.hu.1. |
| 6807 | T2W.7.hu.2. |
| 6808 | T2W.7.hu.3. |
| 6809 | T2W.7.hu.4. |
| 6810 | T2W.7.hu.5. |
| 6811 | T2W.7.hu.6. |
| 6812 | T2W.11.hu.0. |
| 6813 | T2W.11.hu.1. |
| 6814 | T2W.11.hu.2. |
| 6815 | T2W.11.hu.3. |
| 6816 | T2W.11.hu.4. |
| 6817 | T2W.11.hu.5. |
| 6818 | T2W.11.hu.6. |
| 6819 | T2W.15.hu.0. |
| 6820 | T2W.15.hu.1. |
| 6821 | T2W.15.hu.2. |
| 6822 | T2W.15.hu.3. |
| 6823 | T2W.15.hu.4. |
| 6824 | T2W.15.hu.5. |
| 6825 | T2W.15.hu.6. |
| 6826 | T2W.19.hu.0. |
| 6827 | T2W.19.hu.1. |
| 6828 | T2W.19.hu.2. |
| 6829 | T2W.19.hu.3. |
| 6830 | T2W.19.hu.4. |
| 6831 | T2W.19.hu.5. |
| 6832 | T2W.19.hu.6. |
| 6833 | T2W.23.hu.0. |
| 6834 | T2W.23.hu.1. |
| 6835 | T2W.23.hu.2. |
| 6836 | T2W.23.hu.3. |
| 6837 | T2W.23.hu.4. |
| 6838 | T2W.23.hu.5. |
| 6839 | T2W.23.hu.6. |
| 6840 | T2W.27.hu.0. |
| 6841 | T2W.27.hu.1. |
| 6842 | T2W.27.hu.2. |
| 6843 | T2W.27.hu.3. |
| 6844 | T2W.27.hu.4. |
| 6845 | T2W.27.hu.5. |
| 6846 | T2W.27.hu.6. |
| 6847 | T2W.31.hu.0. |
| 6848 | T2W.31.hu.1. |
| 6849 | T2W.31.hu.2. |
| 6850 | T2W.31.hu.3. |
| 6851 | T2W.31.hu.4. |
| 6852 | T2W.31.hu.5. |
| 6853 | T2W.31.hu.6. |
| 6854 | T2W.35.hu.0. |
| 6855 | T2W.35.hu.1. |
| 6856 | T2W.35.hu.2. |
| 6857 | T2W.35.hu.3. |
| 6858 | T2W.35.hu.4. |
| 6859 | T2W.35.hu.5. |
| 6860 | T2W.35.hu.6. |
| 6861 | T2W.3.zernike.0. |
| 6862 | T2W.3.zernike.1. |
| 6863 | T2W.3.zernike.2. |
| 6864 | T2W.3.zernike.3. |
| 6865 | T2W.3.zernike.4. |
| 6866 | T2W.3.zernike.5. |
| 6867 | T2W.3.zernike.6. |
| 6868 | T2W.3.zernike.7. |
| 6869 | T2W.3.zernike.8. |
| 6870 | T2W.3.zernike.9. |
| 6871 | T2W.3.zernike.10. |
| 6872 | T2W.3.zernike.11. |
| 6873 | T2W.3.zernike.12. |
| 6874 | T2W.3.zernike.13. |
| 6875 | T2W.3.zernike.14. |
| 6876 | T2W.3.zernike.15. |
| 6877 | T2W.3.zernike.16. |
| 6878 | T2W.3.zernike.17. |
| 6879 | T2W.3.zernike.18. |
| 6880 | T2W.3.zernike.19. |
| 6881 | T2W.3.zernike.20. |
| 6882 | T2W.3.zernike.21. |
| 6883 | T2W.3.zernike.22. |
| 6884 | T2W.3.zernike.23. |
| 6885 | T2W.3.zernike.24. |
| 6886 | T2W.7.zernike.0. |
| 6887 | T2W.7.zernike.1. |
| 6888 | T2W.7.zernike.2. |
| 6889 | T2W.7.zernike.3. |
| 6890 | T2W.7.zernike.4. |
| 6891 | T2W.7.zernike.5. |
| 6892 | T2W.7.zernike.6. |
| 6893 | T2W.7.zernike.7. |
| 6894 | T2W.7.zernike.8. |
| 6895 | T2W.7.zernike.9. |
| 6896 | T2W.7.zernike.10. |
| 6897 | T2W.7.zernike.11. |
| 6898 | T2W.7.zernike.12. |
| 6899 | T2W.7.zernike.13. |
| 6900 | T2W.7.zernike.14. |
| 6901 | T2W.7.zernike.15. |
| 6902 | T2W.7.zernike.16. |
| 6903 | T2W.7.zernike.17. |
| 6904 | T2W.7.zernike.18. |
| 6905 | T2W.7.zernike.19. |
| 6906 | T2W.7.zernike.20. |
| 6907 | T2W.7.zernike.21. |
| 6908 | T2W.7.zernike.22. |
| 6909 | T2W.7.zernike.23. |
| 6910 | T2W.7.zernike.24. |
| 6911 | T2W.11.zernike.0. |
| 6912 | T2W.11.zernike.1. |
| 6913 | T2W.11.zernike.2. |
| 6914 | T2W.11.zernike.3. |
| 6915 | T2W.11.zernike.4. |
| 6916 | T2W.11.zernike.5. |
| 6917 | T2W.11.zernike.6. |
| 6918 | T2W.11.zernike.7. |
| 6919 | T2W.11.zernike.8. |
| 6920 | T2W.11.zernike.9. |
| 6921 | T2W.11.zernike.10. |
| 6922 | T2W.11.zernike.11. |
| 6923 | T2W.11.zernike.12. |
| 6924 | T2W.11.zernike.13. |
| 6925 | T2W.11.zernike.14. |
| 6926 | T2W.11.zernike.15. |
| 6927 | T2W.11.zernike.16. |
| 6928 | T2W.11.zernike.17. |
| 6929 | T2W.11.zernike.18. |
| 6930 | T2W.11.zernike.19. |
| 6931 | T2W.11.zernike.20. |
| 6932 | T2W.11.zernike.21. |
| 6933 | T2W.11.zernike.22. |
| 6934 | T2W.11.zernike.23. |
| 6935 | T2W.11.zernike.24. |
| 6936 | T2W.15.zernike.0. |
| 6937 | T2W.15.zernike.1. |
| 6938 | T2W.15.zernike.2. |
| 6939 | T2W.15.zernike.3. |
| 6940 | T2W.15.zernike.4. |
| 6941 | T2W.15.zernike.5. |
| 6942 | T2W.15.zernike.6. |
| 6943 | T2W.15.zernike.7. |
| 6944 | T2W.15.zernike.8. |
| 6945 | T2W.15.zernike.9. |
| 6946 | T2W.15.zernike.10. |
| 6947 | T2W.15.zernike.11. |
| 6948 | T2W.15.zernike.12. |
| 6949 | T2W.15.zernike.13. |
| 6950 | T2W.15.zernike.14. |
| 6951 | T2W.15.zernike.15. |
| 6952 | T2W.15.zernike.16. |
| 6953 | T2W.15.zernike.17. |
| 6954 | T2W.15.zernike.18. |
| 6955 | T2W.15.zernike.19. |
| 6956 | T2W.15.zernike.20. |
| 6957 | T2W.15.zernike.21. |
| 6958 | T2W.15.zernike.22. |
| 6959 | T2W.15.zernike.23. |
| 6960 | T2W.15.zernike.24. |
| 6961 | T2W.19.zernike.0. |
| 6962 | T2W.19.zernike.1. |
| 6963 | T2W.19.zernike.2. |
| 6964 | T2W.19.zernike.3. |
| 6965 | T2W.19.zernike.4. |
| 6966 | T2W.19.zernike.5. |
| 6967 | T2W.19.zernike.6. |
| 6968 | T2W.19.zernike.7. |
| 6969 | T2W.19.zernike.8. |
| 6970 | T2W.19.zernike.9. |
| 6971 | T2W.19.zernike.10. |
| 6972 | T2W.19.zernike.11. |
| 6973 | T2W.19.zernike.12. |
| 6974 | T2W.19.zernike.13. |
| 6975 | T2W.19.zernike.14. |
| 6976 | T2W.19.zernike.15. |
| 6977 | T2W.19.zernike.16. |
| 6978 | T2W.19.zernike.17. |
| 6979 | T2W.19.zernike.18. |
| 6980 | T2W.19.zernike.19. |
| 6981 | T2W.19.zernike.20. |
| 6982 | T2W.19.zernike.21. |
| 6983 | T2W.19.zernike.22. |
| 6984 | T2W.19.zernike.23. |
| 6985 | T2W.19.zernike.24. |
| 6986 | T2W.23.zernike.0. |
| 6987 | T2W.23.zernike.1. |
| 6988 | T2W.23.zernike.2. |
| 6989 | T2W.23.zernike.3. |
| 6990 | T2W.23.zernike.4. |
| 6991 | T2W.23.zernike.5. |
| 6992 | T2W.23.zernike.6. |
| 6993 | T2W.23.zernike.7. |
| 6994 | T2W.23.zernike.8. |
| 6995 | T2W.23.zernike.9. |
| 6996 | T2W.23.zernike.10. |
| 6997 | T2W.23.zernike.11. |
| 6998 | T2W.23.zernike.12. |
| 6999 | T2W.23.zernike.13. |
| 7000 | T2W.23.zernike.14. |
| 7001 | T2W.23.zernike.15. |
| 7002 | T2W.23.zernike.16. |
| 7003 | T2W.23.zernike.17. |
| 7004 | T2W.23.zernike.18. |
| 7005 | T2W.23.zernike.19. |
| 7006 | T2W.23.zernike.20. |
| 7007 | T2W.23.zernike.21. |
| 7008 | T2W.23.zernike.22. |
| 7009 | T2W.23.zernike.23. |
| 7010 | T2W.23.zernike.24. |
| 7011 | T2W.27.zernike.0. |
| 7012 | T2W.27.zernike.1. |
| 7013 | T2W.27.zernike.2. |
| 7014 | T2W.27.zernike.3. |
| 7015 | T2W.27.zernike.4. |
| 7016 | T2W.27.zernike.5. |
| 7017 | T2W.27.zernike.6. |
| 7018 | T2W.27.zernike.7. |
| 7019 | T2W.27.zernike.8. |
| 7020 | T2W.27.zernike.9. |
| 7021 | T2W.27.zernike.10. |
| 7022 | T2W.27.zernike.11. |
| 7023 | T2W.27.zernike.12. |
| 7024 | T2W.27.zernike.13. |
| 7025 | T2W.27.zernike.14. |
| 7026 | T2W.27.zernike.15. |
| 7027 | T2W.27.zernike.16. |
| 7028 | T2W.27.zernike.17. |
| 7029 | T2W.27.zernike.18. |
| 7030 | T2W.27.zernike.19. |
| 7031 | T2W.27.zernike.20. |
| 7032 | T2W.27.zernike.21. |
| 7033 | T2W.27.zernike.22. |
| 7034 | T2W.27.zernike.23. |
| 7035 | T2W.27.zernike.24. |
| 7036 | T2W.31.zernike.0. |
| 7037 | T2W.31.zernike.1. |
| 7038 | T2W.31.zernike.2. |
| 7039 | T2W.31.zernike.3. |
| 7040 | T2W.31.zernike.4. |
| 7041 | T2W.31.zernike.5. |
| 7042 | T2W.31.zernike.6. |
| 7043 | T2W.31.zernike.7. |
| 7044 | T2W.31.zernike.8. |
| 7045 | T2W.31.zernike.9. |
| 7046 | T2W.31.zernike.10. |
| 7047 | T2W.31.zernike.11. |
| 7048 | T2W.31.zernike.12. |
| 7049 | T2W.31.zernike.13. |
| 7050 | T2W.31.zernike.14. |
| 7051 | T2W.31.zernike.15. |
| 7052 | T2W.31.zernike.16. |
| 7053 | T2W.31.zernike.17. |
| 7054 | T2W.31.zernike.18. |
| 7055 | T2W.31.zernike.19. |
| 7056 | T2W.31.zernike.20. |
| 7057 | T2W.31.zernike.21. |
| 7058 | T2W.31.zernike.22. |
| 7059 | T2W.31.zernike.23. |
| 7060 | T2W.31.zernike.24. |
| 7061 | T2W.35.zernike.0. |
| 7062 | T2W.35.zernike.1. |
| 7063 | T2W.35.zernike.2. |
| 7064 | T2W.35.zernike.3. |
| 7065 | T2W.35.zernike.4. |
| 7066 | T2W.35.zernike.5. |
| 7067 | T2W.35.zernike.6. |
| 7068 | T2W.35.zernike.7. |
| 7069 | T2W.35.zernike.8. |
| 7070 | T2W.35.zernike.9. |
| 7071 | T2W.35.zernike.10. |
| 7072 | T2W.35.zernike.11. |
| 7073 | T2W.35.zernike.12. |
| 7074 | T2W.35.zernike.13. |
| 7075 | T2W.35.zernike.14. |
| 7076 | T2W.35.zernike.15. |
| 7077 | T2W.35.zernike.16. |
| 7078 | T2W.35.zernike.17. |
| 7079 | T2W.35.zernike.18. |
| 7080 | T2W.35.zernike.19. |
| 7081 | T2W.35.zernike.20. |
| 7082 | T2W.35.zernike.21. |
| 7083 | T2W.35.zernike.22. |
| 7084 | T2W.35.zernike.23. |
| 7085 | T2W.35.zernike.24. |
| 7086 | T2W.3.sobel |
| 7087 | T2W.3.sobel_mask |
| 7088 | T2W.all.stats.p000. |
| 7089 | T2W.all.stats.p010. |
| 7090 | T2W.all.stats.p020. |
| 7091 | T2W.all.stats.p025. |
| 7092 | T2W.all.stats.p030. |
| 7093 | T2W.all.stats.p040. |
| 7094 | T2W.all.stats.p050. |
| 7095 | T2W.all.stats.p060. |
| 7096 | T2W.all.stats.p070. |
| 7097 | T2W.all.stats.p075. |
| 7098 | T2W.all.stats.p080. |
| 7099 | T2W.all.stats.p090. |
| 7100 | T2W.all.stats.p100. |
| 7101 | T2W.all.stats.range. |
| 7102 | T2W.all.stats.mean. |
| 7103 | T2W.all.stats.stddev. |
| 7104 | T2W.all.stats.kurtosis. |
| 7105 | T2W.all.stats.skewness. |

Supplementary Table 3 provides a list of functions along with their corresponding R packages

| No. | Task | Function name in R | Package |
| --- | --- | --- | --- |
| 1 | Dividing the dataset into training set and testing set | createdataPartition | caret |
| 2. | Randomly choose clinical references to be permuted | sample | base |
| 3. | Mann Whitney U test | wilcox.test | stats |
| 4. | Recursive Feature Elimination (RFE) | rfe | caret |
| 5. | Least Absolute Shrinkage and Selection Operator (LASSO) | cv.glmnet | glmnet |
| 6. | Minimum Redundancy Maximum Relevance (MRMR) | mRMR.ensemble | mRMRe |
| 7. | Random Forest (RF) | train  (with method set to “rf”) | caret |

Supplementary Table 4 showing the parameters of the feature selections used in this study

| Feature Selection | Parameters |
| --- | --- |
| Mann Whitney U | P-value < 0.05 |
| Recursive Feature Elimination (RFE) | Linear regression estimator, 10-fold cross-validation, number of features selected depends on the number of features that maximes the performance metric (root mean squared error (RMSE)) based on cross-validation. |
| LASSO | 10 fold cross validation, regularization parameter using minimum cross-validation error criterion |
| MRMR | Mutual information (MI) as relevance measure, conditional mutual information (CMI) as redundancy measure, and a weighted sum method to combine measures |

Supplementary Table 5 showing the parameters of the LASSO and Random Forest models

| Model | Parameters | Evaluation metric |
| --- | --- | --- |
| LASSO | Lambda = lambda min*, 5 and 10-fold CV | AUC |
| Random Forest | 250 trees, max node=5 , mtry=1-50, 5 and 10-fold CV | CFM |

*Lambda min was obtained by using the cv.glmnet() function in R to fit a LASSO regression model with elastic net regularization. 10-fold cross-validation to select the optimal value of the tuning parameter lambda, which controls the strength of the L1 regularization penalty. The lamda.min value which corresponds to the minimum cross-validated error, was then chosen as the optimal value of lambda.

Supplementary Table 6 shows the indices of robust features selected by three different feature selections in each dataset with different levels of permutation of clinical reference.

| Dataset | Ratio of permuted clinical reference | Robust Features selected | | |
| --- | --- | --- | --- | --- |
|  |  | Whitney U Test | RFE | MRMR |
| Dataset1 | 0% | 3, 4, 5, 6, 7, 8, 9, 11, 12, 13, 14, 15, 16, 17, 18, 19, 20, 21, 22, 23, 24, 25, 26, 27, 28, 29, 30, 31, 32, 37, 38, 39, 40, 41, 42, 43, 44, 45, 46, 47, 48, 49, 50, 51, 52, 53, 54, 55, 56, 57, 58, 59, 60, 61, 62, 63, 64, 65, 66, 68, 69, 70, 71, 72, 73, 74, 75, 76, 91, 93, 94, 95, 96, 98, 100, 102, 103, 104, 105, 107, 108, 109, 110, 111, 113, 114, 115, 116, 117, 118, 119, 120, 121, 122, 123, 124, 125, 126, 127, 128, 129, 130, 131, 132, 133, 134, 135, 136, 137, 138, 139, 140, 141, 142, 143, 144, 145, 146, 147, 148, 149, 150, 151, 152, 153, 154, 155, 156, 157, 158, 159, 160, 161, 162, 163, 164, 179, 180, 181, 182, 185, 187, 189, 190, 191, 193, 194, 195, 203, 209, 210, 211, 212, 221, 225, 227, 228, 230, 231, 232, 233, 234, 236, 237, 238, 239, 240, 241, 242, 243, 245, 248, 249, 250, 251, 252, 253, 254, 255, 256, 257, 258, 259, 260, 261, 262, 263, 264, 265 | 254, 78, 166, 109, 142, 100, 79, 167, 104, 255, 55 | 14, 27, 37, 55, 73, 74, 75, 103, 133, 137, 139, 142, 143, 144, 146, 147, 148, 203, 250, 251, 253, 254, 255, 262, 28, 29, 190, 234, 17, 96, 98, 100, 4, 5, 13, 180, 231 |
|  | 5% | 3, 4, 5, 6, 7, 8, 11, 12, 13, 14, 15, 16, 17, 19, 20, 21, 22, 23, 24, 25, 26, 27, 28, 29, 30, 31, 32, 37, 38, 39, 40, 41, 42, 43, 44, 45, 46, 47, 48, 49, 50, 51, 52, 53, 54, 55, 56, 57, 58, 59, 60, 61, 62, 63, 64, 65, 66, 68, 69, 72, 73, 74, 75, 76, 91, 93, 94, 95, 96, 98, 100, 102, 103, 104, 107, 108, 109, 110, 111, 113, 114, 115, 116, 117, 118, 119, 120, 121, 122, 123, 124, 125, 126, 127, 128, 129, 130, 131, 132, 133, 134, 135, 136, 137, 138, 139, 140, 141, 142, 143, 144, 145, 146, 147, 148, 149, 150, 151, 152, 153, 154, 156, 157, 158, 161, 162, 163, 164, 179, 180, 181, 182, 185, 187, 189, 190, 191, 193, 194, 195, 203, 209, 210, 211, 212, 221, 225, 227, 228, 230, 231, 232, 233, 234, 236, 237, 238, 239, 240, 241, 242, 243, 245, 248, 250, 251, 252, 253, 254, 255, 256, 257, 258, 259, 260, 261, 262, 263, 264, 265 | 254 | 27, 55, 74, 133, 144, 146, 147, 148, 203, 251, 254, 255, 190 |
|  | 10% | 3, 4, 5, 6, 7, 8, 11, 12, 13, 14, 15, 16, 17, 19, 20, 21, 22, 23, 25, 26, 27, 28, 29, 30, 31, 32, 37, 38, 39, 40, 41, 42, 43, 44, 45, 46, 47, 48, 49, 50, 51, 52, 53, 54, 55, 56, 57, 58, 59, 60, 61, 62, 63, 64, 65, 66, 68, 69, 72, 73, 74, 75, 76, 91, 93, 94, 95, 96, 98, 100, 102, 103, 104, 107, 108, 109, 110, 111, 113, 114, 115, 116, 117, 118, 119, 120, 121, 122, 124, 125, 126, 127, 128, 129, 130, 131, 132, 133, 135, 136, 137, 139, 140, 142, 143, 144, 145, 146, 147, 148, 149, 150, 151, 153, 154, 157, 158, 162, 163, 164, 179, 180, 181, 185, 187, 189, 190, 191, 193, 203, 209, 210, 211, 221, 225, 227, 228, 230, 231, 232, 233, 234, 236, 237, 239, 241, 242, 243, 245, 250, 251, 252, 253, 254, 255, 256, 257, 258, 259, 260, 261, 262, 263, 264, 265 | 254, 142 | 27, 144, 146, 147, 148, 203, 251, 254, 255, 190 |
|  | 15% | 3, 4, 5, 6, 7, 8, 11, 12, 13, 14, 15, 16, 17, 19, 20, 21, 23, 26, 27, 28, 29, 30, 31, 32, 37, 39, 40, 41, 43, 44, 45, 46, 47, 48, 49, 51, 52, 53, 54, 55, 56, 57, 58, 59, 60, 61, 62, 63, 64, 66, 68, 69, 72, 73, 74, 75, 91, 93, 94, 95, 96, 98, 100, 102, 103, 104, 107, 108, 109, 110, 114, 116, 117, 118, 119, 120, 125, 126, 128, 130, 131, 133, 136, 137, 139, 140, 142, 143, 144, 145, 146, 147, 148, 149, 153, 154, 158, 162, 163, 164, 179, 180, 181, 185, 187, 189, 190, 193, 203, 209, 221, 225, 227, 230, 231, 232, 233, 234, 237, 239, 241, 242, 243, 245, 250, 251, 252, 253, 254, 255, 256, 257, 258, 259, 260, 261, 262, 263, 264, 265 | 254, 142 | 27, 203, 251, 254, 255, 144, 146 |
|  | 20% | 3, 4, 5, 6, 7, 8, 11, 12, 13, 14, 15, 16, 17, 19, 20, 21, 23, 26, 27, 28, 29, 30, 31, 32, 37, 39, 40, 41, 44, 45, 46, 47, 49, 51, 52, 53, 54, 55, 56, 57, 58, 60, 61, 62, 63, 64, 66, 68, 69, 72, 73, 74, 91, 94, 96, 98, 100, 102, 103, 104, 107, 109, 110, 126, 130, 133, 136, 137, 139, 140, 142, 143, 144, 145, 146, 147, 148, 149, 158, 162, 179, 180, 181, 185, 187, 189, 190, 193, 221, 225, 230, 231, 232, 233, 234, 237, 241, 242, 250, 251, 252, 253, 254, 255, 256, 257, 258, 259, 260, 262, 263, 264, 265 |  | 9, 27, 97, 144, 146, 254, 251 |
|  | 25% | 3, 4, 7, 11, 13, 14, 15, 17, 20, 26, 27, 28, 29, 30, 31, 32, 37, 39, 40, 41, 45, 46, 49, 51, 52, 53, 54, 55, 56, 57, 58, 61, 62, 63, 68, 69, 73, 74, 91, 94, 96, 100, 103, 104, 107, 109, 133, 137, 139, 142, 143, 144, 145, 146, 147, 148, 158, 162, 179, 180, 181, 185, 187, 189, 190, 193, 221, 225, 230, 231, 232, 233, 234, 242, 250, 251, 254, 255, 256, 257, 258, 259, 260, 262, 263, 264, 265 | 254 | 9, 97, 254, 2, 27, 251, 255, 24 |
|  | 30% | 4, 7, 11, 13, 14, 15, 17, 20, 26, 27, 28, 29, 30, 31, 32, 37, 41, 46, 49, 53, 54, 55, 56, 57, 58, 62, 68, 69, 73, 74, 96, 100, 103, 104, 109, 133, 137, 139, 142, 143, 144, 146, 147, 148, 190, 225, 230, 231, 232, 234, 250, 251, 254, 255, 256, 257, 258, 260, 262, 263, 264 |  | 9, 97, 106, 112, 200, 253, 254, 2 |
|  | 35% | 4, 7, 14, 20, 26, 27, 28, 29, 30, 31, 32, 37, 46, 49, 55, 56, 57, 58, 62, 74, 96, 100, 103, 104, 133, 137, 139, 142, 143, 144, 146, 147, 148, 251, 254, 255, 257, 260, 262 | 75, 45, 139, 52, 146, 96, 60 | 9, 10, 97, 106, 112, 161, 185, 200, 253, 2, 246 |
|  | 40% | 4, 14, 27, 28, 29, 30, 31, 32, 37, 49, 55, 56, 57, 58, 74, 133, 137, 139, 142, 143, 144, 146, 147, 148, 251, 254, 255, 257, 260 | 146, 91, 3, 179, 19 | 9, 10, 18, 97, 106, 112, 155, 185, 200, 253, 161, 2 |
|  | 45% | 56, 74, 137, 139, 142, 144, 146, 147, 255, 257, 260 | 212 | 9, 10, 18, 24, 97, 106, 112, 155, 185, 200, 253, 161, 123, 2 |
|  | 50% | 74, 139 | 212, 264, 134, 39, 210, 136, 113 | 9, 18, 24, 97, 106, 112, 155, 161, 200, 203, 246, 253, 71, 2, 185, 160, 10, 105 |
| Dataset2 | 0% | 442, 571, 573, 574, 575, 577, 578, 579, 581, 582, 585, 586, 591, 593, 594, 614, 631, 633, 634, 635, 637, 638, 639, 641, 642, 643, 645, 646, 647, 649, 651, 653, 654, 657, 673, 674, 691, 693, 694, 695, 697, 698, 699, 701, 702, 703, 705, 706, 707, 709, 710, 711, 713, 714, 717, 718, 731, 733, 734, 755, 757, 759, 761, 762, 763, 791, 793, 835, 1052, 1053, 1060, 1067, 1074, 1081, 1265, 1266, 1267, 1852, 1854, 1855, 1856, 1858, 1859, 1860, 1862, 1863, 1864, 1866, 1867, 1870, 1871, 1872, 1874, 1875, 1912, 1914, 1915, 1916, 1918, 1919, 1920, 1922, 1923, 1924, 1926, 1927, 1928, 1930, 1932, 1934, 1935, 1938, 1952, 1954, 1955, 1972, 1974, 1975, 1976, 1978, 1979, 1980, 1982, 1983, 1984, 1986, 1987, 1988, 1990, 1991, 1992, 1994, 1995, 1996, 1998, 2012, 2014, 2015, 2032, 2034, 2035, 2036, 2038, 2039, 2040, 2042, 2043, 2044, 2046, 2048, 2052, 2054, 2055, 2056, 2058, 2072, 2074, 2075, 2100, 2102, 2104, 2108, 2112, 2114, 2116, 2132, 2134, 2334, 2340, 2341, 2348, 2355, 2362, 2546, 2547, 2548, 2549, 2567, 2568, 2569, 2600, 2602, 2603, 2604, 2606, 2607, 2614, 2648, 2650, 2652, 2654, 2662, 2696, 2698, 2699, 2700, 2704, 2706, 2712, 2747, 2748, 2750, 2752, 2754, 2760, 2796, 2798, 2800, 2802, 2804, 2808, 2810, 2835, 2848, 2850, 2852, 2856, 2858, 2883, 2917, 3010, 3011, 3012, 3014, 3018, 3034, 3054, 3074, 3078, 3082, 3086, 3090, 3094, 3098, 3103, 3104, 3114, 3119, 3120, 3123, 3124, 3134, 3138, 3142, 3146, 3148, 3150, 3151, 3152, 3154, 3158, 3162, 3163, 3164, 3174, 3178, 3179, 3180, 3182, 3183, 3184, 3194, 3198, 3202, 3206, 3207, 3208, 3211, 3212, 3214, 3218, 3219, 3220, 3223, 3224, 3236, 3238, 3239, 3240, 3254, 3258, 3262, 3267, 3268, 3271, 3272, 3278, 3279, 3280, 3283, 3284, 3296, 3298, 3299, 3300, 3318, 3328, 3339, 3340, 3434, 3437, 3438, 3459, 3462, 3483, 3485, 3486, 3491, 3492, 3498, 3509, 3510, 3516, 3530, 3553, 3554, 3560, 3566, 3572, 3653, 3657, 3663, 3665, 3671, 3673, 3679, 3704, 3705, 3707, 3826, 3838, 3839, 3840, 3842, 5211, 5218, 5434, 5917, 5919, 5925, 6035, 6036, 6037, 6038, 6039, 6040, 6041, 6042 |  | 77, 5917, 5919 |
|  | 5% | 571, 573, 574, 575, 577, 578, 579, 581, 582, 591, 593, 594, 631, 633, 634, 635, 637, 638, 639, 641, 642, 643, 645, 651, 653, 654, 673, 674, 691, 693, 694, 695, 697, 698, 699, 701, 702, 703, 705, 706, 711, 713, 714, 731, 733, 734, 755, 757, 759, 761, 763, 791, 793, 835, 1060, 1066, 1067, 1074, 1081, 1265, 1266, 1852, 1854, 1855, 1856, 1858, 1859, 1860, 1862, 1872, 1874, 1875, 1912, 1914, 1915, 1916, 1918, 1919, 1920, 1922, 1923, 1924, 1926, 1932, 1934, 1935, 1952, 1954, 1955, 1972, 1974, 1975, 1976, 1978, 1979, 1980, 1982, 1983, 1984, 1986, 1987, 1988, 1992, 1994, 1995, 1996, 1998, 2012, 2014, 2015, 2032, 2034, 2035, 2036, 2038, 2039, 2040, 2042, 2043, 2044, 2046, 2048, 2052, 2054, 2055, 2056, 2058, 2072, 2074, 2075, 2092, 2094, 2095, 2096, 2098, 2099, 2100, 2102, 2104, 2108, 2112, 2114, 2116, 2132, 2134, 2334, 2341, 2347, 2348, 2355, 2362, 2546, 2547, 2548, 2606, 2607, 2614, 2648, 2650, 2760, 2808, 3074, 3094, 3134, 3138, 3142, 3146, 3154, 3158, 3162, 3163, 3164, 3174, 3178, 3179, 3180, 3204, 3207, 3208, 3219, 3220, 3223, 3224, 3233, 3235, 3236, 3238, 3239, 3240, 3268, 3272, 3279, 3280, 3340, 3485, 3492, 3498, 3516, 3653, 3657, 3663, 3665, 3671, 3673, 3679, 3707, 3839, 3840, 3842, 5211, 5218, 5434, 5925, 6035, 6036, 6037, 6038, 6039, 6040, 6041, 6042 |  |  |
|  | 10% | 571, 573, 574, 585, 591, 593, 594, 614, 631, 633, 634, 635, 637, 638, 639, 641, 642, 643, 645, 651, 653, 654, 674, 691, 693, 694, 695, 697, 698, 699, 701, 702, 703, 705, 706, 709, 711, 713, 714, 717, 731, 733, 734, 759, 761, 763, 791, 793, 835, 1060, 1066, 1067, 1074, 1265, 1912, 1914, 1915, 1916, 1918, 1919, 1920, 1922, 1923, 1926, 1927, 1932, 1934, 1935, 1952, 1954, 1955, 1972, 1974, 1975, 1976, 1978, 1979, 1980, 1982, 1983, 1984, 1986, 1987, 1988, 1992, 1994, 1995, 1998, 2012, 2014, 2015, 2032, 2034, 2035, 2036, 2038, 2039, 2040, 2042, 2043, 2044, 2046, 2048, 2052, 2054, 2055, 2056, 2058, 2072, 2074, 2075, 2116, 2132, 2134, 2333, 2341, 2347, 2348, 2355, 2362, 2546, 2547, 2548, 3268, 3279, 3280, 3653, 3657, 3663, 3665, 3671, 3673, 3678, 3679, 3682, 3688, 3690, 3696, 3698, 3705, 3707, 3840, 5211, 5218, 5434, 6035, 6036, 6037, 6038, 6039, 6040, 6041, 6042, 6504 |  |  |
|  | 15% | 442, 634, 635, 637, 638, 639, 641, 643, 691, 693, 694, 695, 697, 698, 699, 701, 702, 711, 713, 714, 717, 733, 734, 763, 791, 793, 1060, 1066, 1074, 1710, 1972, 1974, 1975, 1976, 1978, 1979, 1980, 1982, 1983, 1992, 1994, 1995, 1998, 2012, 2014, 2015, 2040, 2042, 2044, 2048, 2056, 2072, 2074, 2075, 2344, 2346, 2347, 2355, 2362, 2546, 2995, 3154, 3174, 3214, 3219, 3220, 3233, 3235, 3236, 3279, 3280, 3653, 3657, 3663, 3665, 3671, 3673, 3705, 3711, 3717, 3719, 4881, 5211, 5218, 6035, 6036, 6037, 6038, 6039, 6040, 6041, 6042 | 799, 803, 783 |  |
|  | 20% | 425, 442, 674, 691, 693, 694, 698, 711, 713, 714, 717, 733, 734, 839, 1066, 1265, 1710, 1972, 1974, 1975, 1976, 1978, 1979, 1992, 1994, 2012, 2014, 2015, 2056, 2072, 2074, 2116, 2339, 2346, 2347, 2546, 2635, 2995, 2996, 3154, 3214, 3341, 3357, 3653, 3657, 3663, 3665, 3671, 3673, 3678, 3679, 3682, 3688, 3690, 3696, 3698, 3705, 3711, 3840, 4111, 4114, 4207, 4346, 4463, 4641, 4689, 4769, 4833, 4853, 5211, 5218, 5382, 5407, 5409, 5434, 5755, 5801, 5803, 5806, 5807, 5854, 6035, 6036, 6037, 6038, 6039, 6040, 6041, 6042, 6464, 6468, 6472, 6476, 6504, 6564, 6766, 6772 | 5303 | 2335, 4346, 5324, 5303, 5289 |
|  | 25% | 425, 839, 1059, 1063, 1066, 1265, 1706, 1710, 1711, 1720, 1955, 2015, 2044, 2048, 2056, 2072, 2074, 2116, 2331, 2333, 2335, 2337, 2339, 2340, 2342, 2344, 2346, 2347, 2546, 2547, 2686, 2785, 2881, 2941, 2995, 2996, 3194, 3208, 3214, 3219, 3220, 3236, 3279, 3280, 3339, 3340, 3341, 3357, 3653, 3657, 3663, 3665, 3671, 3673, 3678, 3679, 3682, 3688, 3690, 3696, 3698, 3705, 3711, 3717, 3719, 3766, 3840, 3906, 3910, 4105, 4107, 4109, 4111, 4114, 4161, 4162, 4193, 4195, 4197, 4199, 4207, 4209, 4211, 4213, 4215, 4265, 4266, 4267, 4273, 4274, 4346, 4358, 4404, 4425, 4441, 4442, 4443, 4445, 4446, 4447, 4457, 4458, 4461, 4462, 4463, 4466, 4467, 4521, 4522, 4523, 4625, 4641, 4642, 4643, 4645, 4685, 4689, 4701, 4702, 4703, 4709, 4733, 4769, 4833, 4837, 4845, 4853, 4861, 4866, 4867, 4871, 4873, 4881, 4882, 4883, 4887, 4943, 4957, 4958, 4961, 4964, 4967, 4987, 5125, 5211, 5218, 5383, 5407, 5409, 5434, 5753, 5755, 5759, 5761, 5801, 5803, 5804, 5806, 5807, 5814, 5854, 5878, 5894, 6035, 6036, 6037, 6038, 6039, 6040, 6041, 6042, 6337, 6341, 6381, 6397, 6398, 6401, 6402, 6430, 6440, 6441, 6442, 6456, 6458, 6460, 6464, 6468, 6472, 6476, 6484, 6488, 6489, 6490, 6500, 6501, 6502, 6504, 6506, 6518, 6520, 6524, 6528, 6532, 6536, 6544, 6560, 6561, 6562, 6564, 6580, 6730, 6736, 6741, 6742, 6748, 6754, 6760, 6765, 6766, 6771, 6772 | 5314, 5291, 5471, 2347, 5326, 5303, 5289, 5470, 5292, 6511, 2335, 1703, 6555 | 387, 1066, 2335, 2941, 4624, 5299, 5314, 5324, 5585, 7012, 4346, 4449, 5303, 4465, 4445, 4441 |
|  | 30% | 425, 839, 855, 1066, 1077, 1265, 1710, 1715, 1720, 2056, 2060, 2120, 2347, 2358, 2366, 2446, 2546, 2686, 2769, 2785, 2817, 2995, 3341, 3357, 3649, 3653, 3657, 3663, 3665, 3671, 3673, 3705, 3714, 3724, 3766, 3769, 3906, 3910, 3922, 3926, 4109, 4111, 4209, 4211, 4346, 4351, 4358, 4404, 4442, 4443, 4446, 4447, 4461, 4462, 4463, 4465, 4624, 4649, 4689, 4709, 4711, 5211, 5324, 5332, 5407, 6035, 6036, 6037, 6038, 6039, 6040, 6041, 6042 | 5314, 425, 5187, 5291, 5471, 5470, 5201, 5326, 5303, 6207, 5324, 2859, 1281, 1642, 4516, 5289, 3610, 5193, 6850, 2681, 4310, 4306, 6857, 2813 | 425, 2827, 2865, 2941, 3365, 4624, 4876, 5292, 5314, 5316, 5324, 5878, 6027, 6207, 5303, 5307, 5585, 3714, 5332, 7012, 4445, 4449, 5199, 4457, 4465 |
|  | 35% | 424, 425, 434, 775, 839, 855, 1356, 1705, 1706, 1710, 1711, 1715, 1720, 2056, 2060, 2076, 2120, 2136, 2347, 2358, 2769, 2817, 2941, 2952, 2995, 3341, 3652, 3658, 3662, 3664, 3672, 3674, 3677, 3681, 3714, 3719, 3720, 3722, 3724, 3737, 3910, 4094, 4346, 4351, 4358, 4361, 4371, 4376, 4385, 4390, 4624, 5170, 5180, 5326, 5407, 5464, 6020, 6035, 6036, 6037, 6038, 6039, 6040, 6041, 6042 |  | 381, 425, 2120, 2681, 2827, 2941, 3365, 4376, 4465, 4624, 4876, 5201, 5303, 5314, 5326, 6207, 6027, 2683, 4044, 2947 |
|  | 40% | 425, 839, 855, 1066, 1077, 1710, 1720, 2136, 2347, 2769, 2995, 3341, 3357, 3401, 3737, 4351, 4361, 4367, 4371, 4376, 4377, 4385, 6035, 6036, 6037, 6038, 6039, 6040, 6041, 6042 | 77, 5917, 5919 | 381, 1404, 1644, 2827, 2947, 2995, 3365, 4376, 4465, 4624, 5201, 5303, 5326, 6027, 6033, 6207, 6937, 6987, 7012, 7036, 3105, 4387, 2989, 4361 |
|  | 45% | 3737, 4351, 4358, 4361, 4367, 4371, 4376, 4377, 6035, 6036, 6037, 6038, 6039, 6040, 6041, 6042 |  | 1644, 2907, 3708, 3998, 4376, 4624, 5201, 5295, 5326, 5356, 5966, 6033, 6207, 6937, 6984, 5303, 5470, 1780, 2140, 4371, 5314, 5917, 4361 |

|  | 50% | 28, 59, 77, 345, 347, 351, 353, 413, 442, 443, 451, 453, 454, 455, 457, 458, 459, 461, 462, 466, 471, 473, 474, 511, 513, 514, 515, 517, 518, 519, 521, 522, 523, 525, 526, 531, 533, 534, 554, 571, 573, 574, 575, 577, 578, 579, 581, 582, 583, 585, 586, 587, 589, 590, 591, 593, 594, 595, 597, 598, 613, 614, 631, 633, 634, 635, 637, 638, 639, 641, 642, 643, 645, 646, 647, 649, 650, 651, 653, 654, 657, 658, 671, 673, 674, 691, 693, 694, 695, 697, 698, 699, 701, 702, 703, 705, 706, 707, 709, 710, 711, 713, 714, 715, 717, 718, 731, 733, 734, 751, 753, 754, 755, 757, 758, 759, 761, 762, 763, 765, 766, 767, 771, 773, 774, 775, 777, 778, 791, 793, 794, 811, 813, 814, 815, 817, 818, 819, 821, 822, 823, 825, 826, 827, 831, 833, 834, 835, 837, 851, 853, 854, 1039, 1042, 1045, 1046, 1049, 1052, 1053, 1055, 1056, 1060, 1062, 1063, 1064, 1066, 1067, 1074, 1078, 1081, 1091, 1095, 1101, 1103, 1109, 1111, 1145, 1168, 1172, 1264, 1265, 1266, 1267, 1268, 1269, 1270, 1271, 1279, 1673, 1686, 1704, 1710, 1724, 1732, 1734, 1735, 1736, 1738, 1739, 1740, 1742, 1743, 1752, 1754, 1755, 1792, 1794, 1795, 1796, 1798, 1799, 1800, 1802, 1803, 1804, 1806, 1807, 1810, 1811, 1812, 1814, 1815, 1828, 1832, 1834, 1835, 1852, 1854, 1855, 1856, 1858, 1859, 1860, 1862, 1863, 1864, 1866, 1867, 1868, 1870, 1871, 1872, 1874, 1875, 1876, 1878, 1879, 1892, 1894, 1895, 1912, 1914, 1915, 1916, 1918, 1919, 1920, 1922, 1923, 1924, 1926, 1927, 1928, 1930, 1931, 1932, 1934, 1935, 1936, 1938, 1939, 1952, 1954, 1955, 1972, 1974, 1975, 1976, 1978, 1979, 1980, 1982, 1983, 1984, 1986, 1987, 1988, 1990, 1991, 1992, 1994, 1995, 1996, 1998, 1999, 2012, 2014, 2015, 2032, 2034, 2035, 2036, 2038, 2039, 2040, 2042, 2043, 2044, 2046, 2047, 2048, 2050, 2051, 2052, 2054, 2055, 2056, 2058, 2059, 2072, 2074, 2075, 2092, 2094, 2095, 2096, 2098, 2099, 2100, 2102, 2103, 2104, 2106, 2107, 2108, 2112, 2114, 2115, 2116, 2118, 2132, 2134, 2135, 2320, 2323, 2326, 2327, 2330, 2333, 2334, 2336, 2337, 2338, 2340, 2341, 2343, 2344, 2345, 2347, 2348, 2355, 2362, 2372, 2376, 2382, 2384, 2390, 2392, 2424, 2449, 2545, 2546, 2547, 2548, 2549, 2550, 2551, 2560, 2564, 2565, 2566, 2567, 2568, 2569, 2570, 2571, 2572, 2573, 2574, 2575, 2576, 2600, 2601, 2602, 2603, 2604, 2605, 2606, 2607, 2608, 2610, 2611, 2612, 2613, 2614, 2615, 2616, 2622, 2648, 2649, 2650, 2651, 2652, 2653, 2654, 2655, 2656, 2657, 2658, 2660, 2662, 2664, 2666, 2670, 2678, 2696, 2697, 2698, 2699, 2700, 2701, 2702, 2704, 2705, 2706, 2708, 2710, 2712, 2714, 2720, 2744, 2745, 2746, 2747, 2748, 2749, 2750, 2751, 2752, 2753, 2754, 2756, 2758, 2760, 2762, 2764, 2768, 2770, 2771, 2772, 2774, 2784, 2786, 2787, 2788, 2789, 2790, 2792, 2794, 2795, 2796, 2797, 2798, 2799, 2800, 2802, 2804, 2806, 2808, 2810, 2812, 2814, 2816, 2818, 2819, 2821, 2832, 2833, 2834, 2835, 2837, 2844, 2846, 2848, 2850, 2852, 2854, 2856, 2858, 2860, 2862, 2864, 2866, 2868, 2870, 2880, 2881, 2882, 2883, 2884, 2885, 2888, 2889, 2890, 2892, 2893, 2894, 2895, 2896, 2898, 2902, 2904, 2907, 2909, 2912, 2914, 2915, 2917, 2919, 2928, 2931, 2933, 2996, 3009, 3010, 3011, 3012, 3014, 3018, 3022, 3026, 3030, 3032, 3034, 3038, 3042, 3043, 3044, 3047, 3048, 3051, 3052, 3054, 3058, 3059, 3060, 3062, 3063, 3064, 3074, 3078, 3082, 3086, 3090, 3091, 3092, 3094, 3098, 3102, 3103, 3104, 3107, 3108, 3111, 3112, 3114, 3118, 3119, 3120, 3122, 3123, 3124, 3133, 3134, 3135, 3136, 3137, 3138, 3139, 3140, 3141, 3142, 3143, 3144, 3145, 3146, 3147, 3148, 3150, 3151, 3152, 3153, 3154, 3155, 3156, 3158, 3159, 3160, 3162, 3163, 3164, 3167, 3168, 3173, 3174, 3175, 3176, 3178, 3179, 3180, 3182, 3183, 3184, 3193, 3194, 3195, 3196, 3197, 3198, 3199, 3200, 3201, 3202, 3203, 3204, 3205, 3206, 3207, 3208, 3209, 3211, 3212, 3213, 3214, 3215, 3216, 3217, 3218, 3219, 3220, 3222, 3223, 3224, 3227, 3228, 3233, 3234, 3235, 3236, 3238, 3239, 3240, 3243, 3244, 3253, 3254, 3255, 3256, 3257, 3258, 3259, 3260, 3261, 3262, 3263, 3264, 3265, 3267, 3268, 3269, 3271, 3272, 3273, 3274, 3275, 3276, 3277, 3278, 3279, 3280, 3283, 3284, 3293, 3294, 3295, 3296, 3298, 3299, 3300, 3313, 3314, 3315, 3316, 3317, 3318, 3319, 3320, 3321, 3322, 3323, 3324, 3325, 3327, 3328, 3329, 3331, 3332, 3333, 3334, 3335, 3336, 3337, 3338, 3339, 3340, 3353, 3354, 3355, 3356, 3359, 3360, 3373, 3375, 3376, 3377, 3379, 3380, 3381, 3383, 3384, 3385, 3387, 3388, 3389, 3393, 3395, 3396, 3397, 3399, 3400, 3413, 3415, 3416, 3433, 3434, 3435, 3436, 3437, 3438, 3439, 3440, 3441, 3442, 3444, 3445, 3446, 3447, 3448, 3450, 3451, 3453, 3457, 3458, 3459, 3460, 3461, 3462, 3465, 3468, 3471, 3474, 3477, 3480, 3481, 3482, 3483, 3484, 3485, 3486, 3487, 3489, 3490, 3491, 3492, 3493, 3495, 3496, 3497, 3498, 3499, 3501, 3502, 3503, 3504, 3505, 3506, 3507, 3508, 3509, 3510, 3511, 3512, 3513, 3514, 3515, 3516, 3517, 3518, 3519, 3520, 3521, 3522, 3523, 3524, 3527, 3528, 3529, 3530, 3531, 3532, 3533, 3534, 3535, 3536, 3537, 3538, 3539, 3540, 3541, 3542, 3543, 3544, 3545, 3546, 3547, 3548, 3549, 3550, 3551, 3552, 3553, 3554, 3555, 3559, 3560, 3561, 3562, 3563, 3565, 3566, 3567, 3568, 3569, 3571, 3572, 3574, 3575, 3577, 3583, 3584, 3589, 3590, 3595, 3596, 3622, 3629, 3636, 3639, 3643, 3651, 3653, 3654, 3655, 3657, 3659, 3660, 3661, 3663, 3665, 3666, 3667, 3668, 3669, 3671, 3673, 3676, 3678, 3679, 3680, 3682, 3684, 3685, 3686, 3688, 3690, 3691, 3692, 3693, 3694, 3696, 3698, 3704, 3705, 3707, 3709, 3710, 3711, 3716, 3717, 3719, 3731, 3734, 3735, 3740, 3746, 3748, 3760, 3825, 3826, 3835, 3836, 3837, 3838, 3839, 3840, 3842, 3843, 4136, 4153, 4155, 4203, 4205, 4207, 4253, 4255, 4302, 4358, 4833, 4853, 5191, 5198, 5200, 5202, 5205, 5211, 5214, 5215, 5218, 5222, 5225, 5314, 5383, 5384, 5409, 5431, 5434, 5757, 5759, 5765, 5767, 5909, 5911, 5915, 5917, 5919, 5925, 5927, 6444, 6920, 6926, 6934, 7089 | 77, 5917, 5919, 586, 3596 | 77, 351, 1434, 2449, 2917, 3748, 4104, 4255, 4941, 5191, 5200, 5211, 5314, 5315, 5353, 5383, 5533, 5613, 5767, 5917, 5919, 5990, 6484, 717, 3009, 353, 2336, 2546, 2885, 2837, 1265, 733, 2343, 5215, 691, 693, 694, 695, 697, 707, 711, 713, 714, 2712 |
| --- | --- | --- | --- | --- |

Supplementary Figure 1 shows the Jaccard Coefficient Index for different thresholds (a) 5%, (b) 10%, (c) 20%, (d) 30% for RFE, MRMR, and LASSO feature selection for Dataset 1, similarly for Dataset 2 with different threshold such as (e) 5% (f) 10% (g) 20% (h) 30%. Jaccard Coefficient Index has a similar trend across different thresholds.


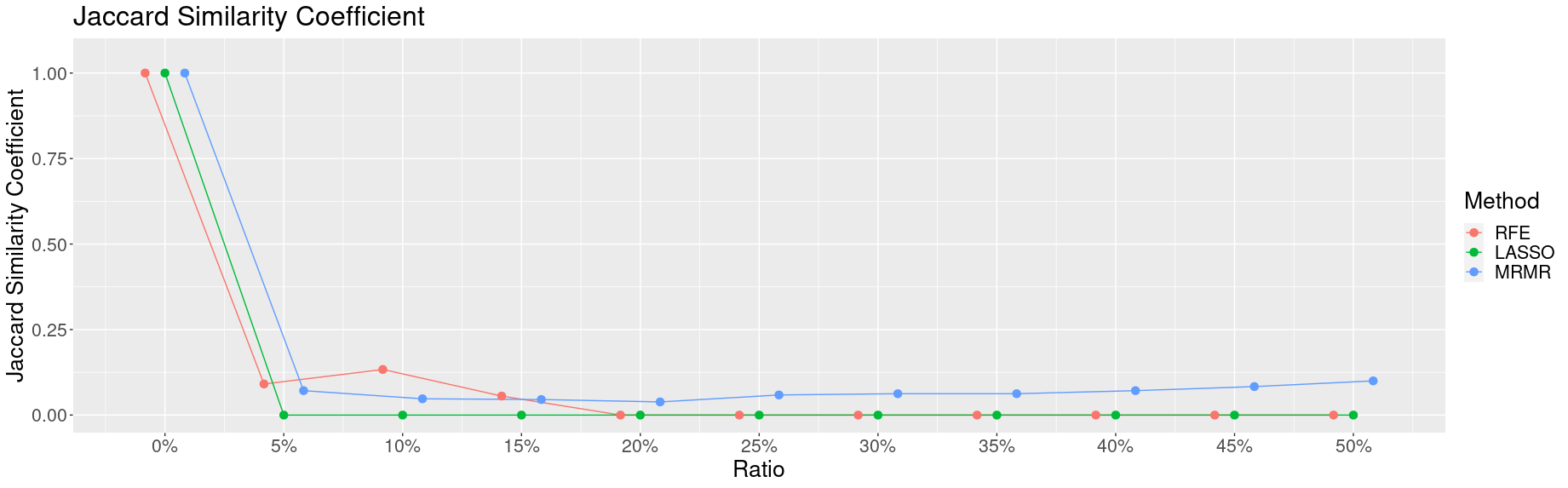


(a)


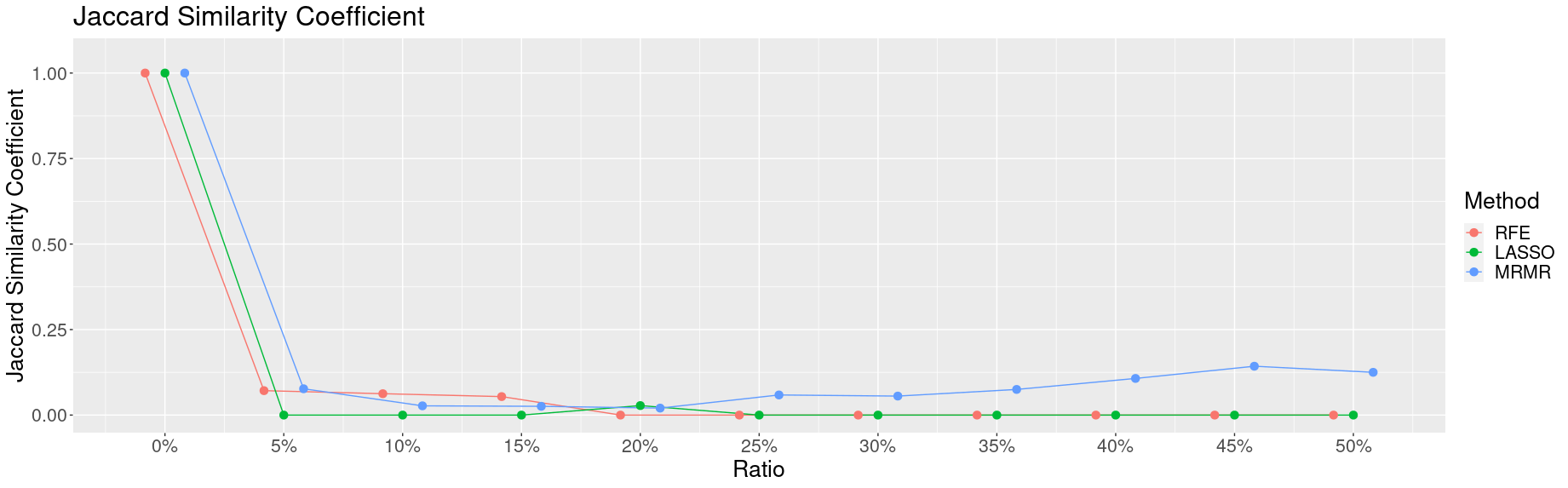


(b)


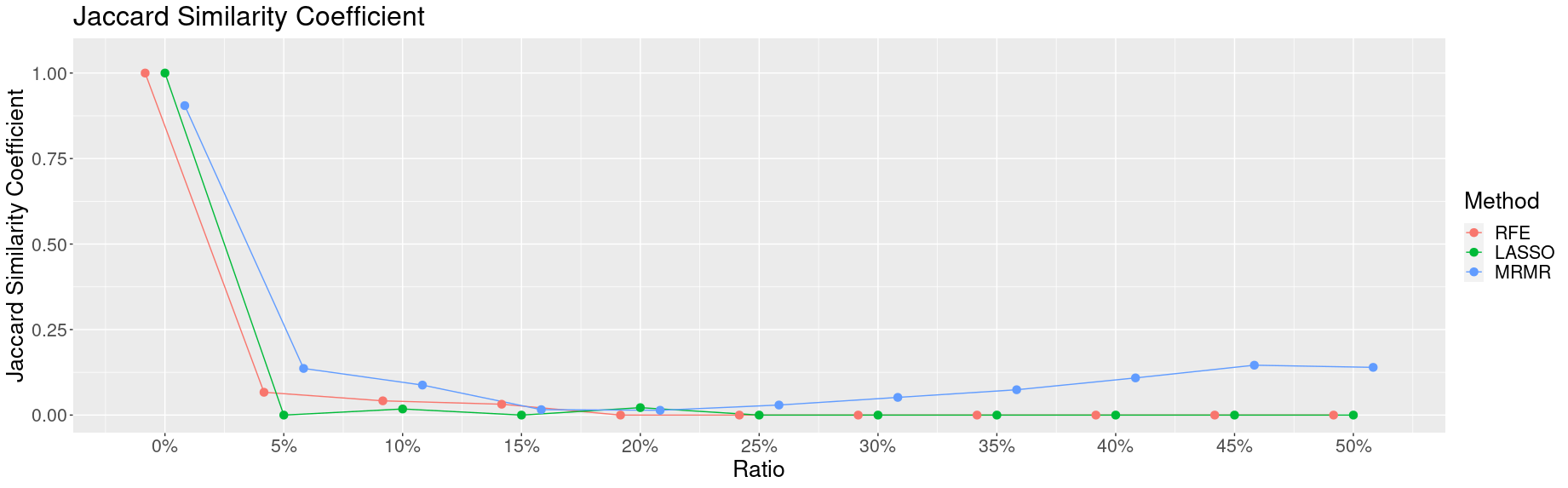


(c)


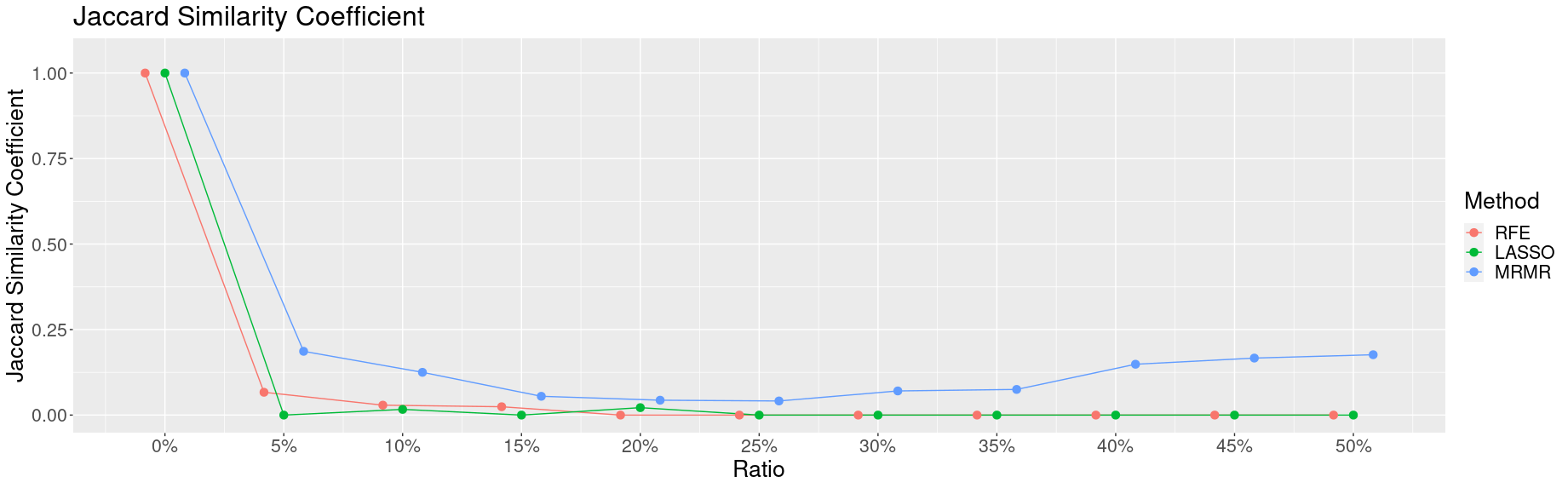


(d)


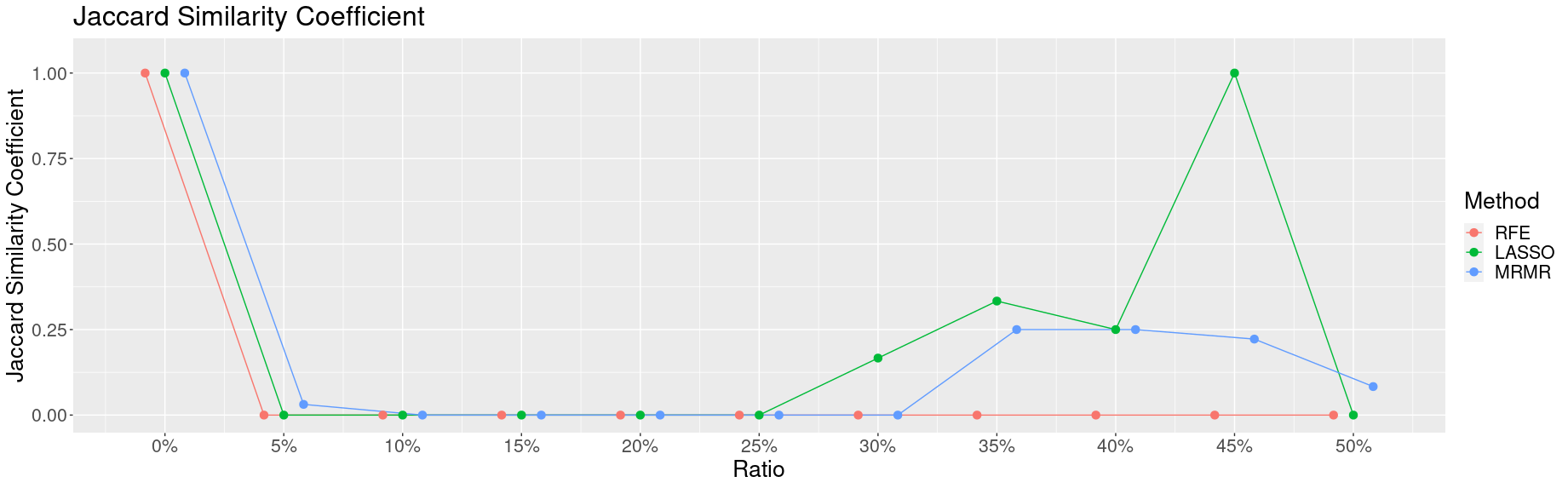


(e)


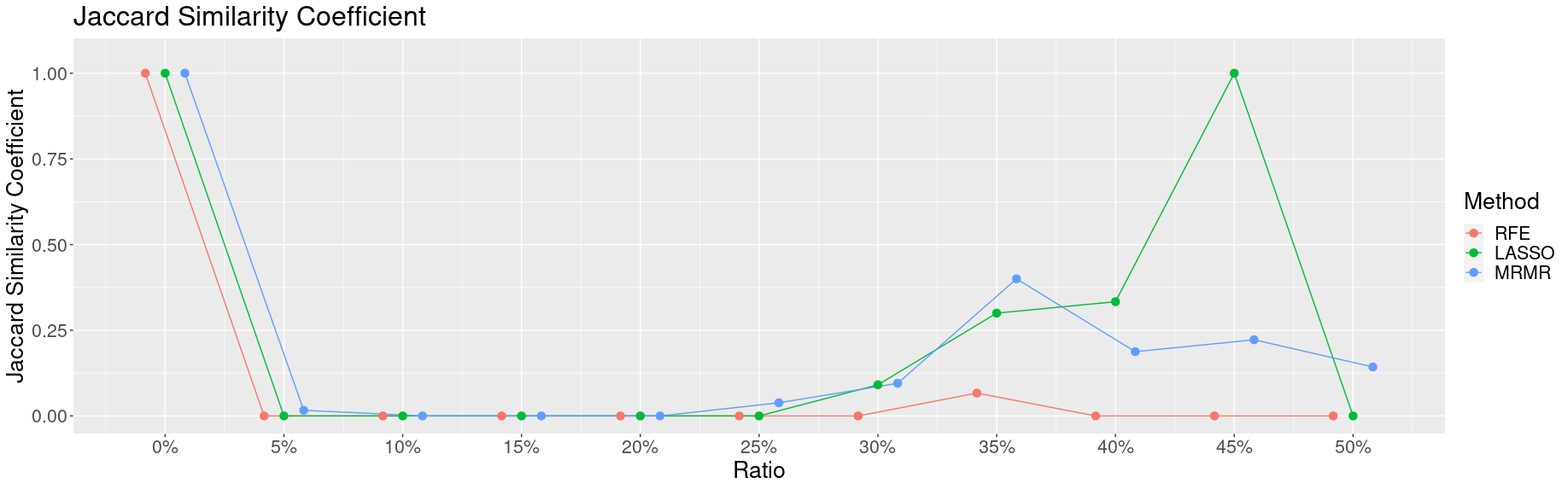


(f)


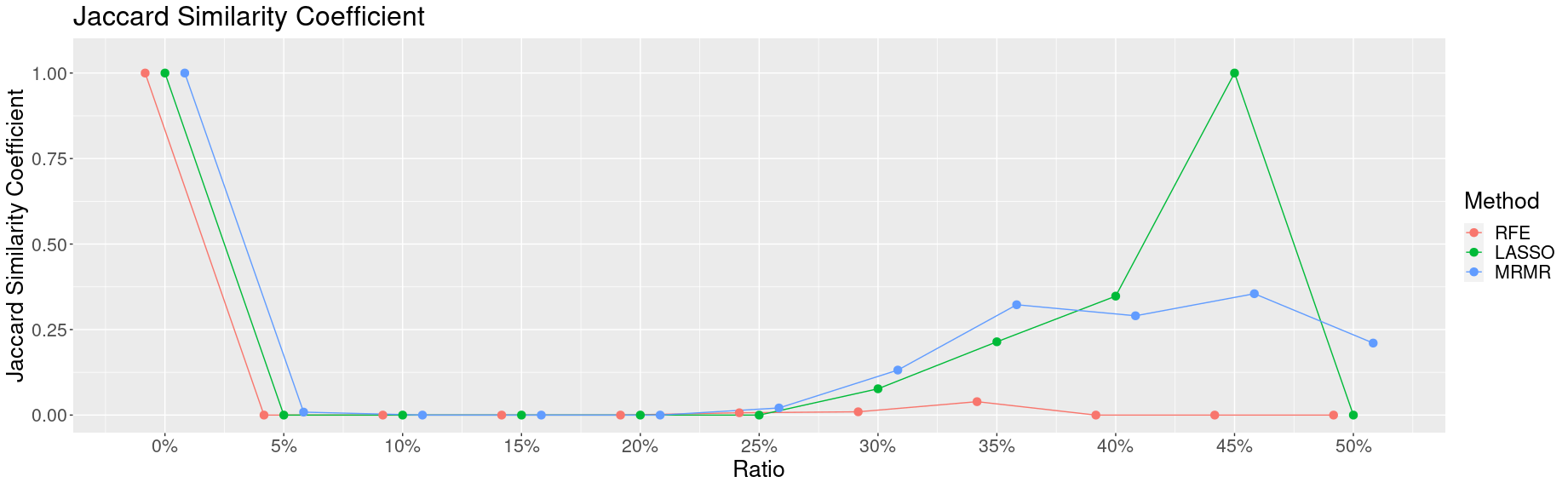


(g)


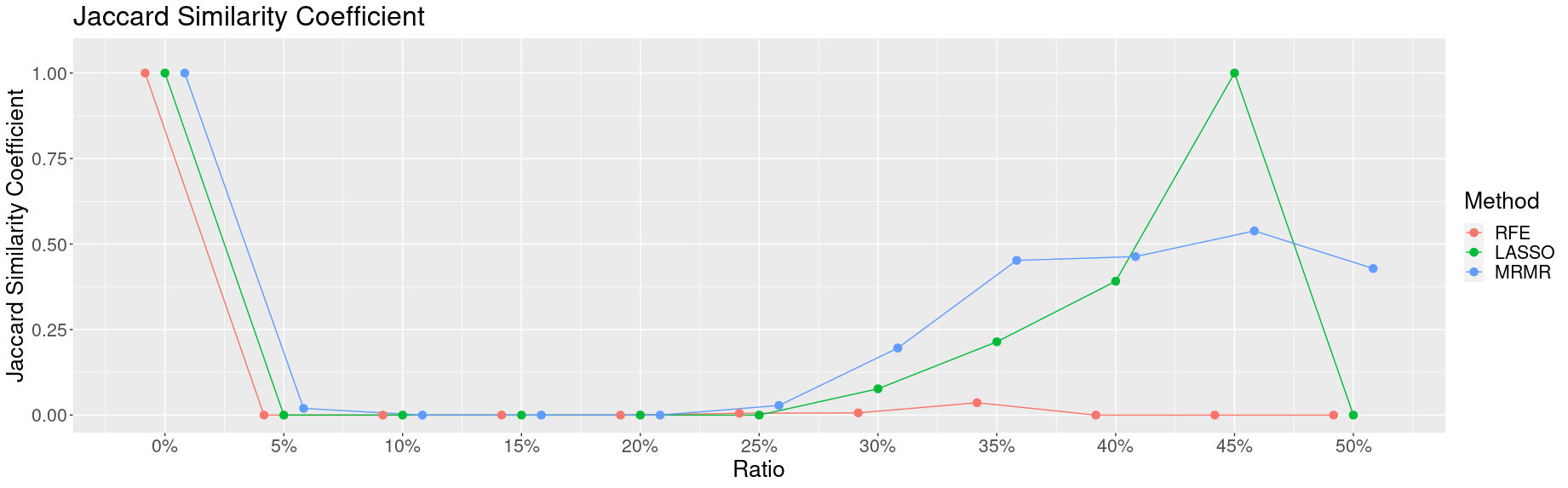


(h)

Supplementary Figure 2 shows the sensitivity of the 8 models calculated for training dataset (with permuted and true clinical reference) and testing dataset in 2 different datasets with different level/ratio of permutation towards the clinical reference.


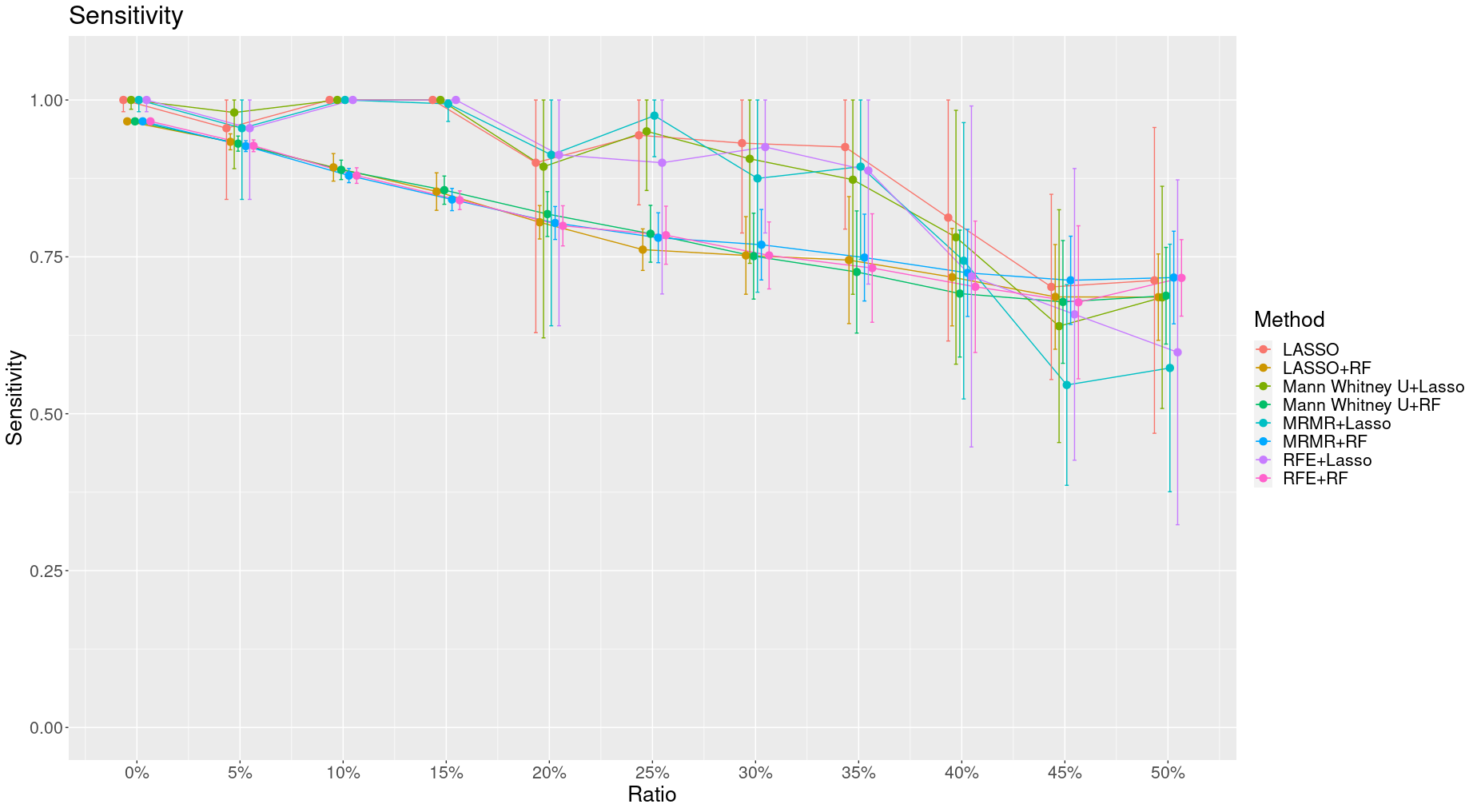

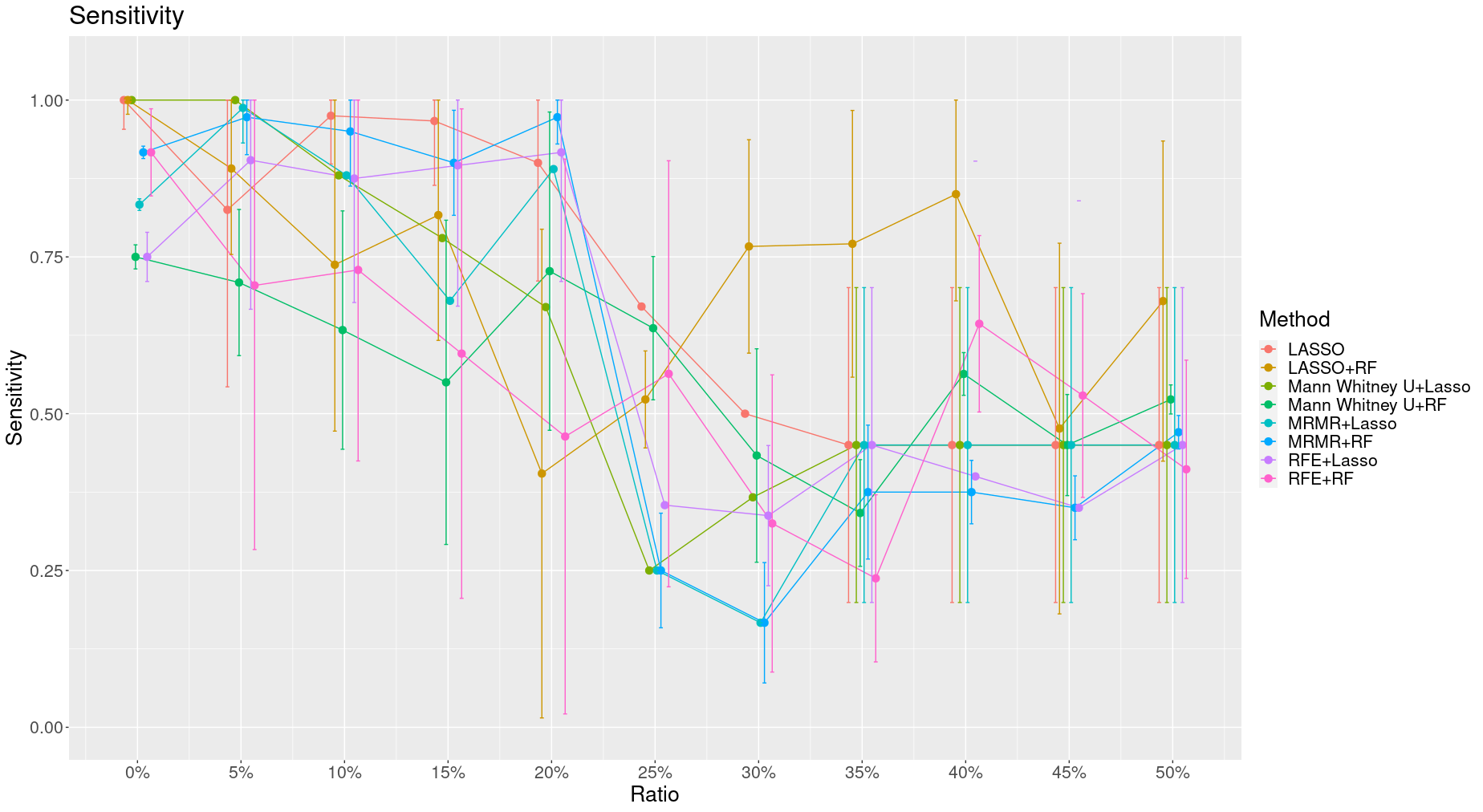


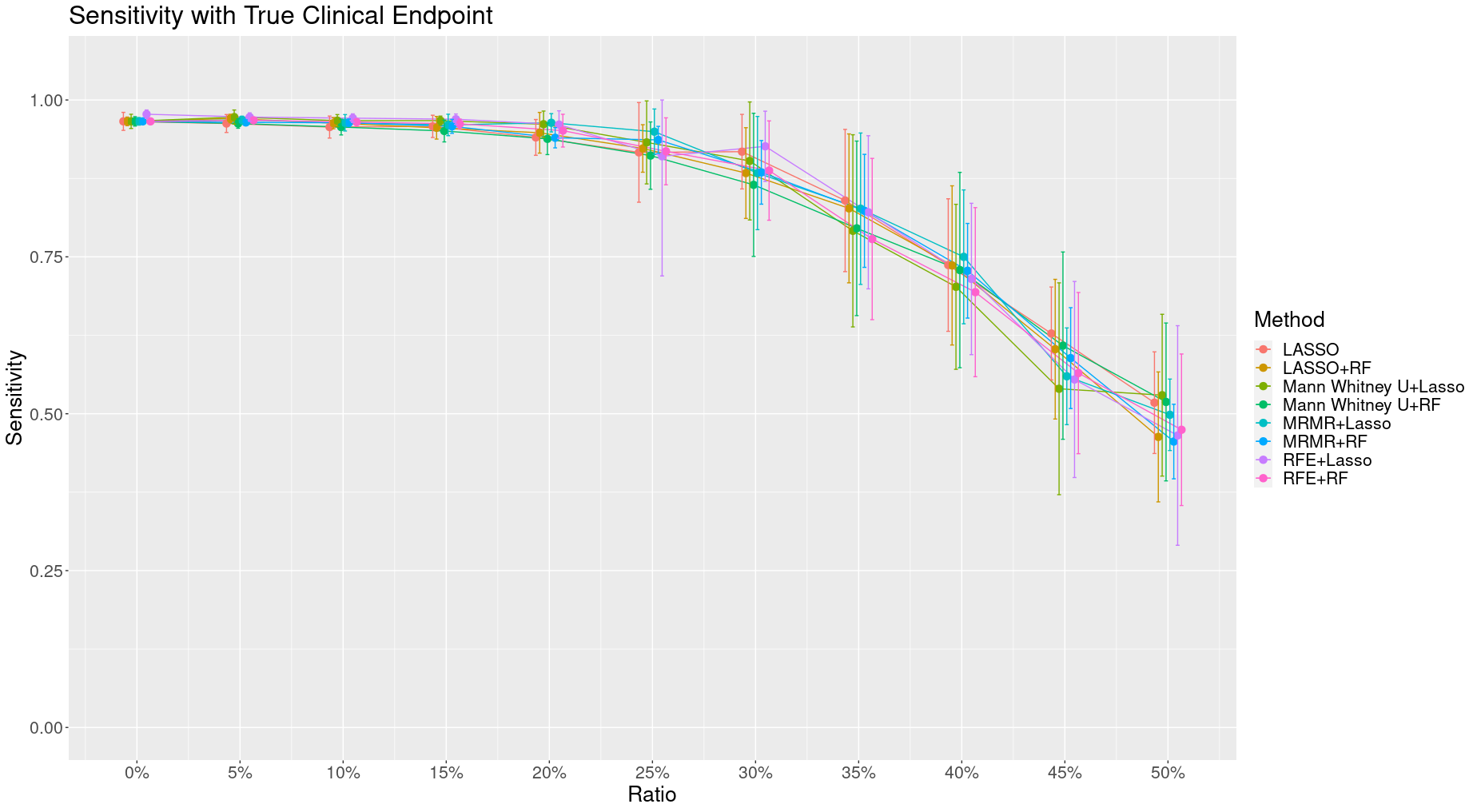

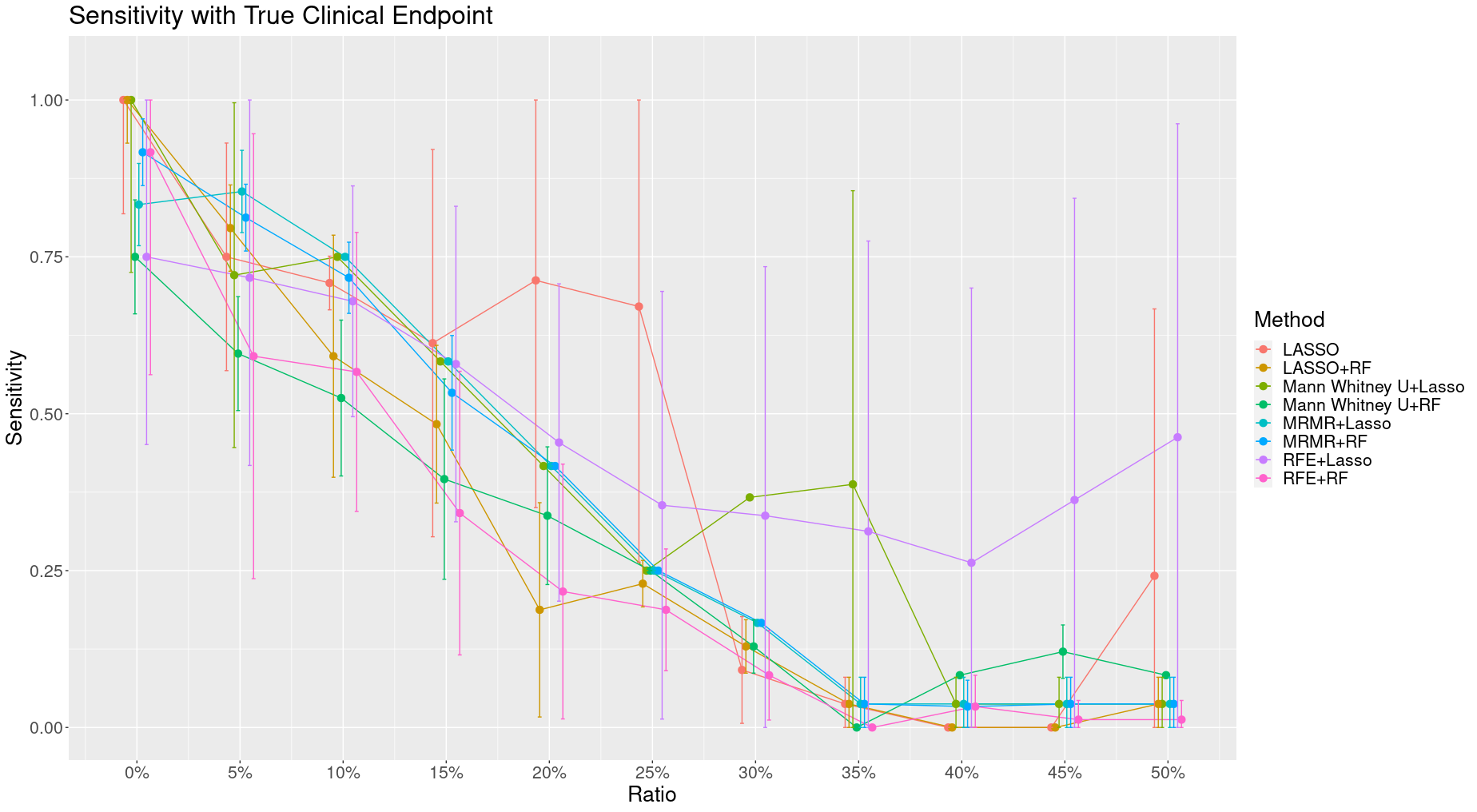


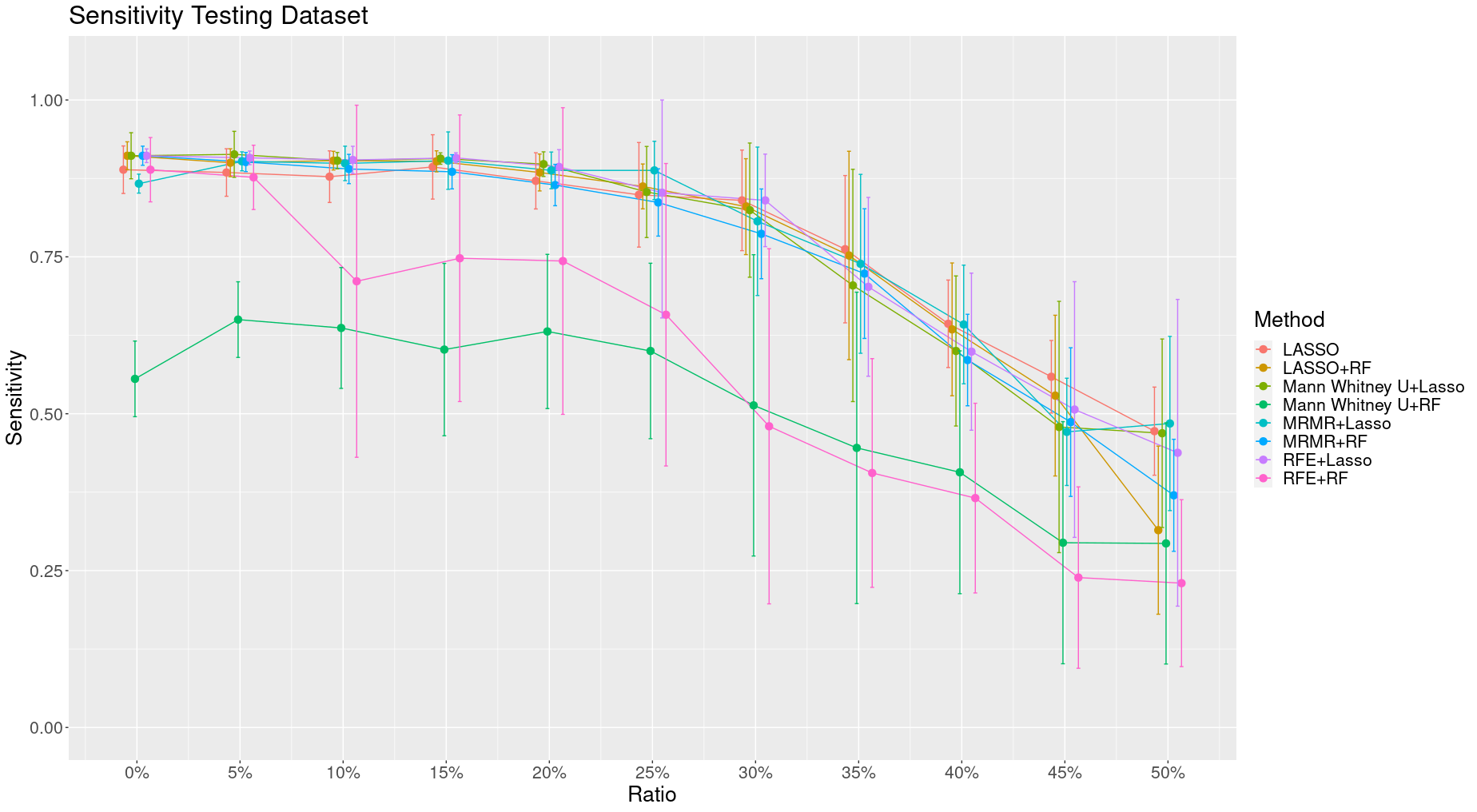

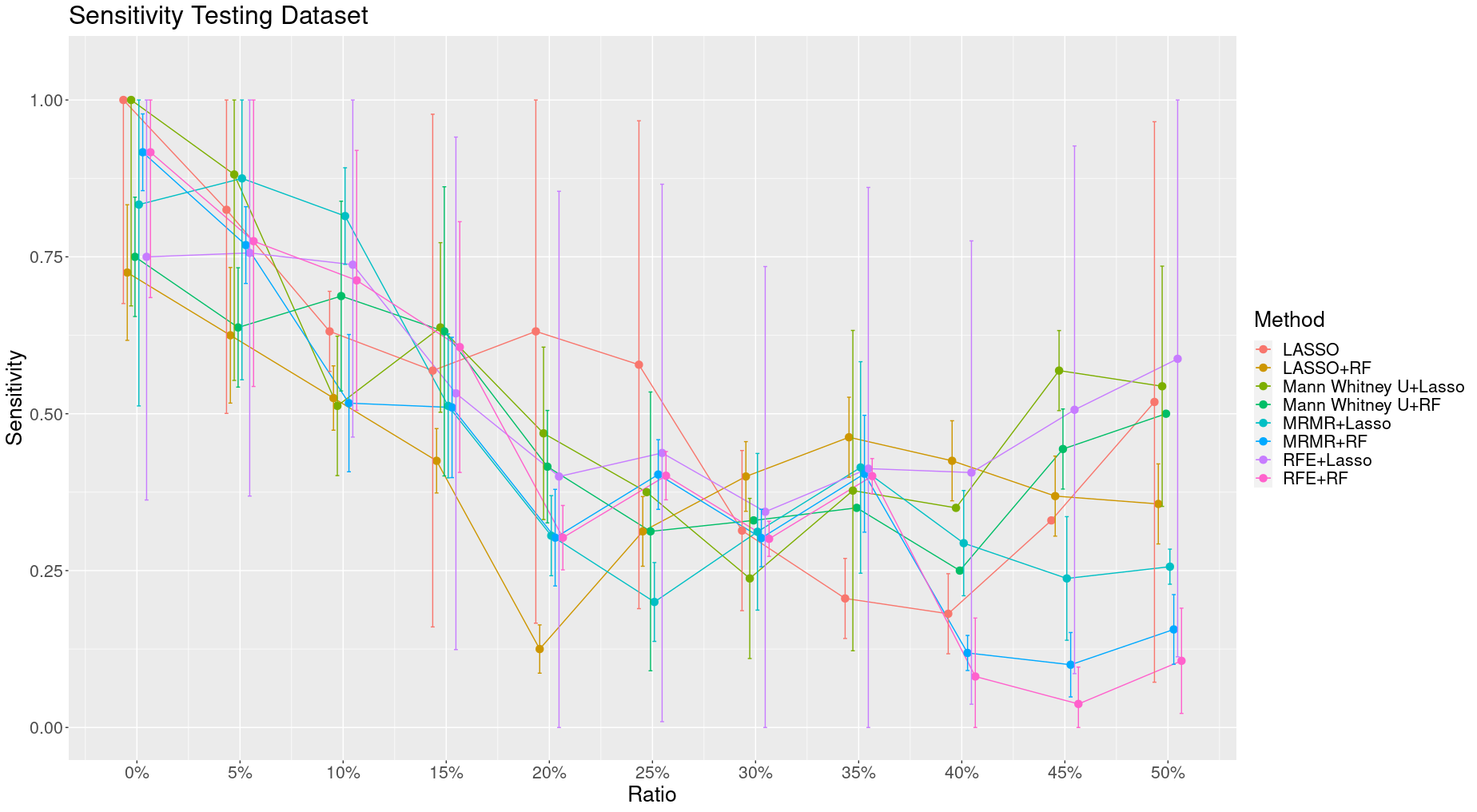
 Dataset 1 Dataset 2

Supplementary Figure 3 shows the specificity of the 8 models calculated for training dataset (with permuted and true clinical reference) and testing dataset in 2 different datasets with different level/ratio of permutation towards the clinical reference.


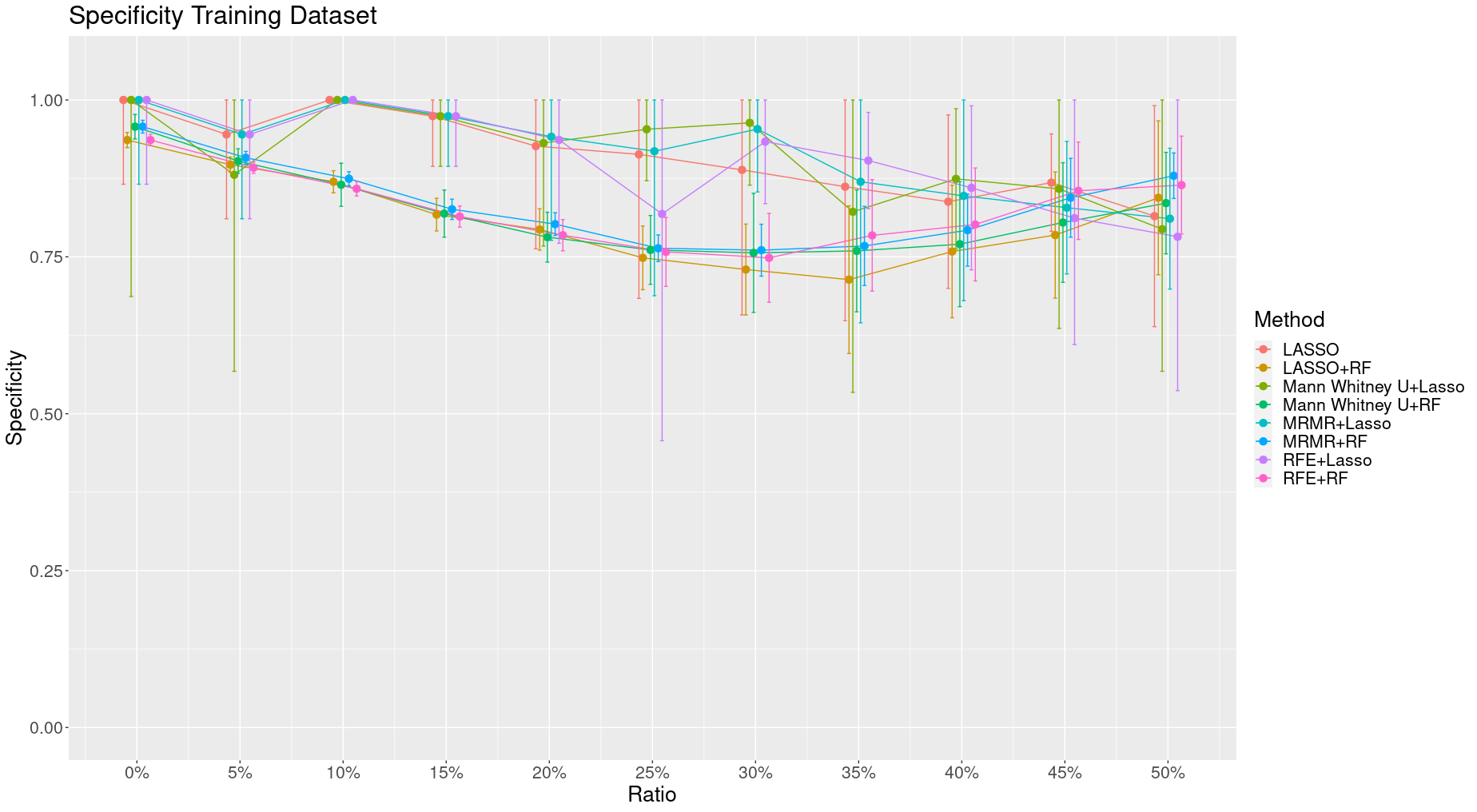

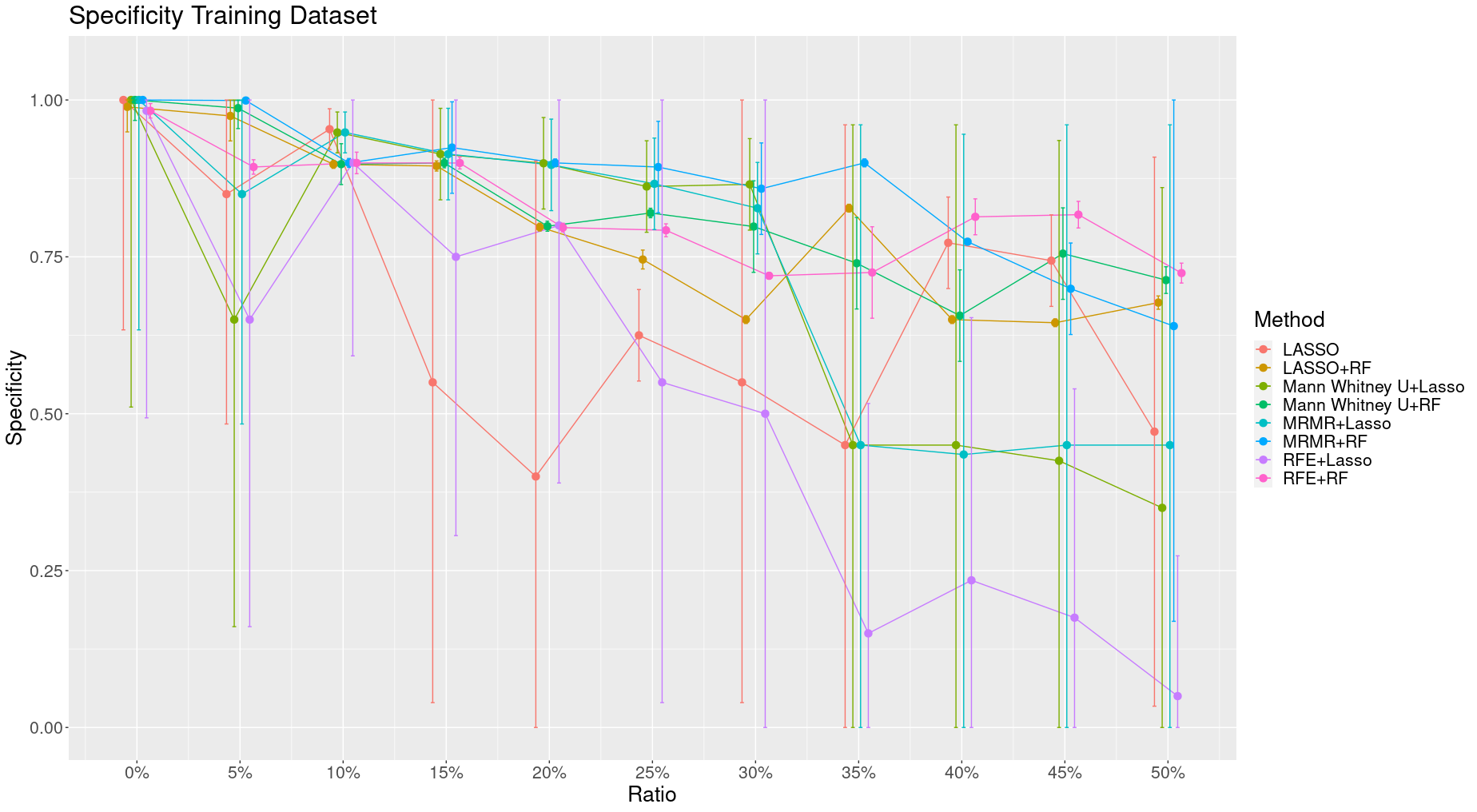


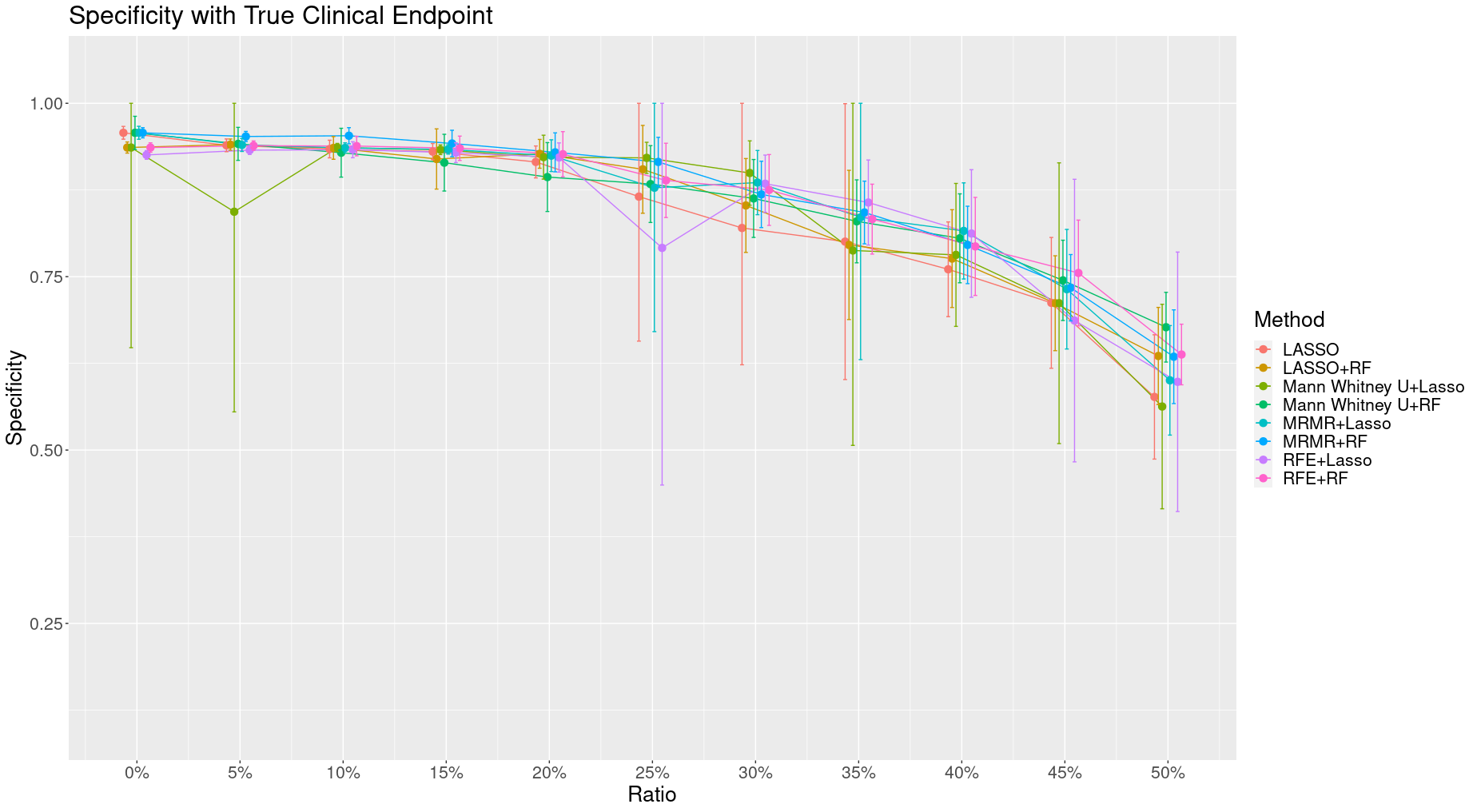

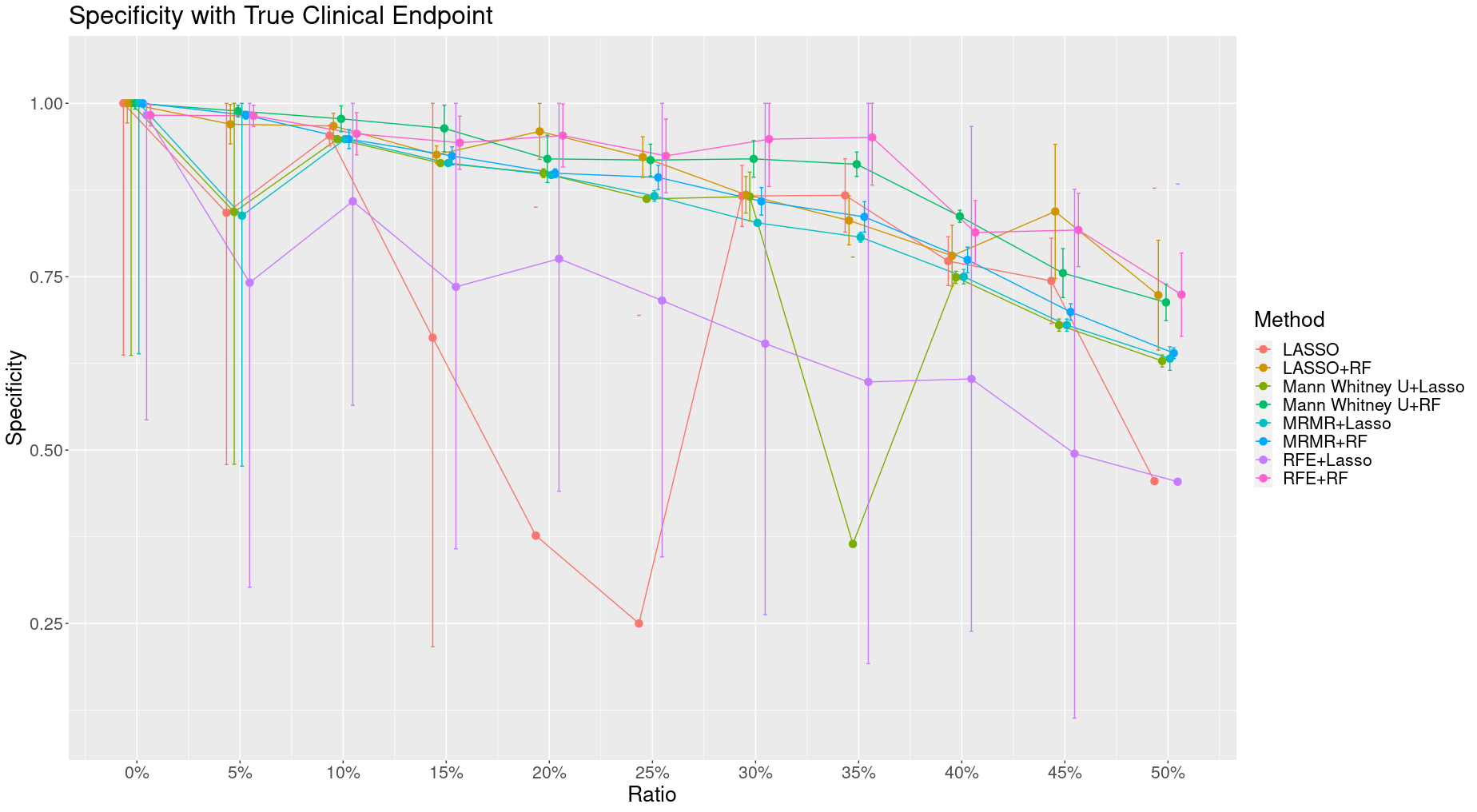


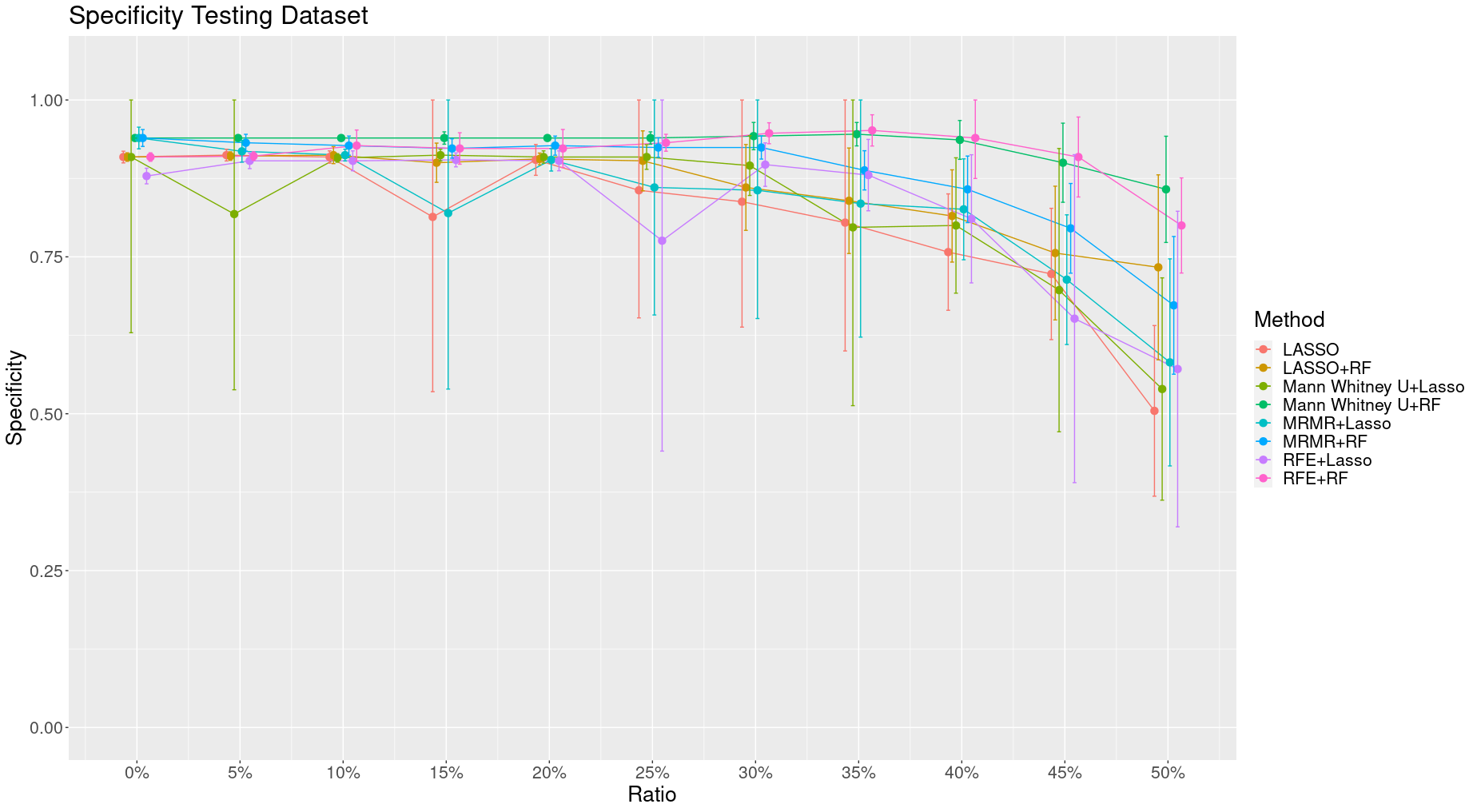

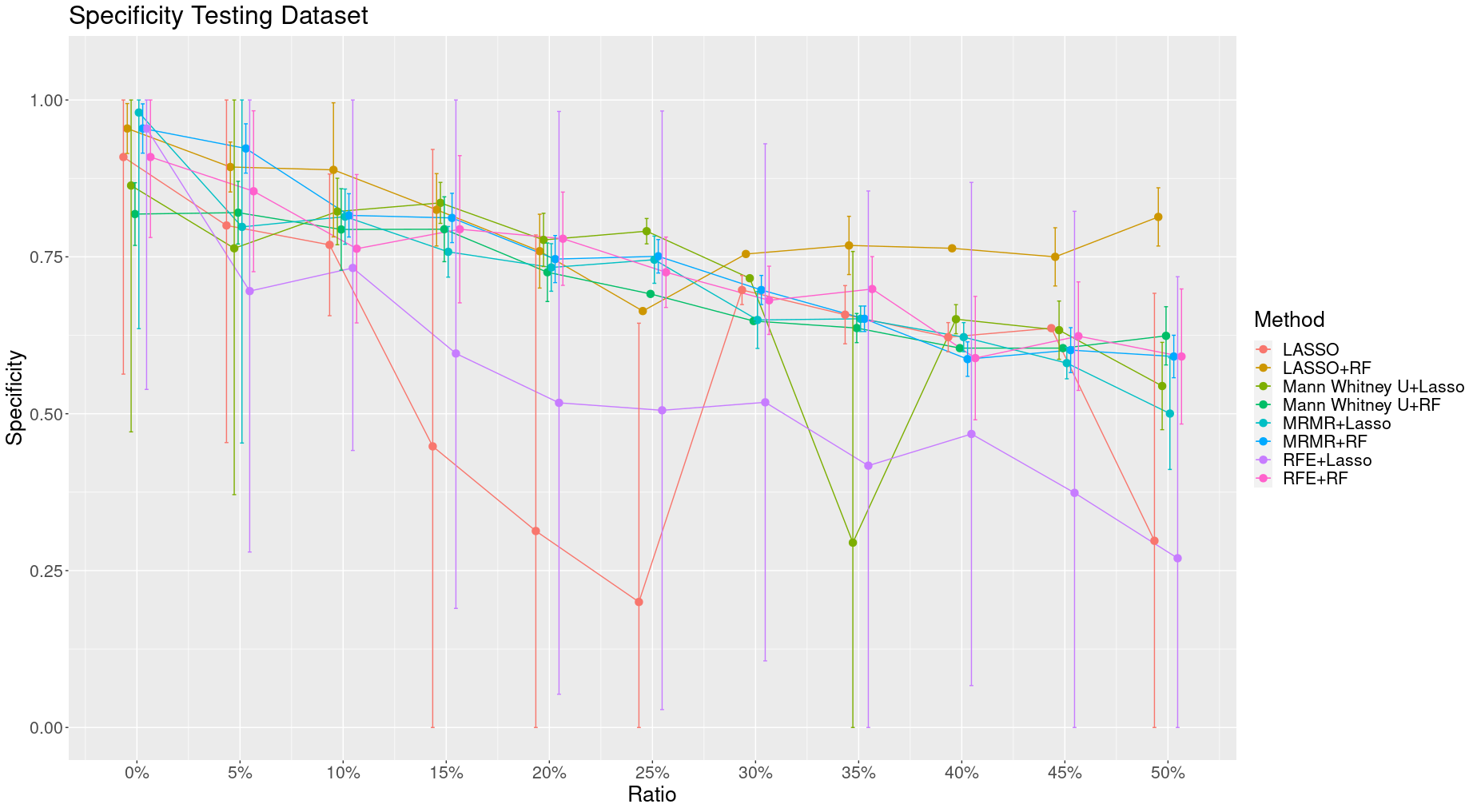


Dataset 1 Dataset 2
